# The development and mechanistic investigation of a palladium-catalyzed 1,3-arylfluorination of chromenes†
†Electronic supplementary information (ESI) available. CCDC 1518394. For ESI and crystallographic data in CIF or other electronic format see DOI: 10.1039/c6sc05102b



**DOI:** 10.1039/c6sc05102b

**Published:** 2017-02-09

**Authors:** Richard T. Thornbury, Vaneet Saini, Talita de A. Fernandes, Celine B. Santiago, Eric P. A. Talbot, Matthew S. Sigman, Jeffrey M. McKenna, F. Dean Toste

**Affiliations:** a Department of Chemistry , University of California , Berkeley , California 94720 , USA . Email: fdtoste@berkeley.edu; b Novartis Institutes for Biomedical Research , Cambridge , Massachusetts 02139 , USA; c Instituto de Química , Universidade de Brasília , Campus Universitário Darcy Ribeiro , Caixa Postal: 04478 , 70904-970 , Brasília , DF , Brazil; d Department of Chemistry , University of Utah , Salt Lake City , Utah 84112 , USA

## Abstract

A mild palladium-catalyzed ligand-controlled regioselective 1,3-arylfluorination of 2[*H*]-chromenes has been developed. The products with a *syn*-1,3 substitution pattern were obtained with high enantiomeric excess using a PyrOx ligand, wherein the utility of these pyranyl-fluorides was further demonstrated through their participation in a diastereoselective C–C bond forming reaction. Ligand dependent divergent formation of both the 1,3- and 1,2- alkene difunctionalization products was observed. The nature of this bifurcation was investigated through experimental studies in combination with computational and statistical analysis tools. Ultimately, the site selectivity was found to rely on ligand denticity and metal electrophilicity, the electronics of the boronic acid, and the donor ability of the directing group in the substrate.

## Abstract 2

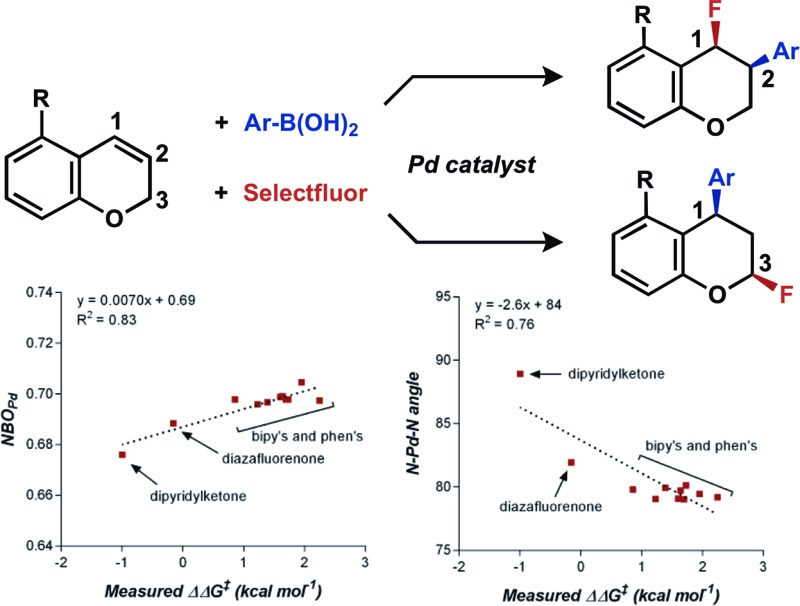
A mild palladium-catalyzed ligand-controlled regioselective 1,3-arylfluorination of 2[*H*]-chromenes has been developed.

## Introduction

**Scheme 1:**
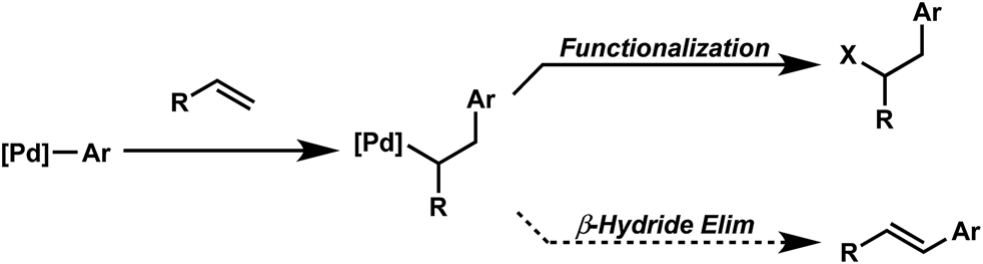
Palladium-catalyzed difunctionalization *via* Interrupted Mizoroki–Heck reaction.

Oxidative difunctionalization of alkenes *via* multi-component reactions is an attractive strategy to rapidly introduce complexity and diversity.^1^ As such, intercepting Mizoroki–Heck intermediates has recently garnered attention as a means to achieve this type of transformation.^2^ In this approach, the σ-alkyl palladium intermediate formed *via* insertion of an olefin into a [Pd]-aryl intermediate is functionalized rather than undergoing β-hydride elimination and alkene dissociation typical of the Heck reaction (Scheme 1). Although some of the earliest reports on the Heck reaction described the formation of alkene difunctionalization products, presumably formed *via* a similar mechanistic scenario, the outlined strategy had been applied sparingly to the development of new synthetic methods.^3^

In particular, this strategy was recently revived in the development of palladium-catalyzed protocols for the 1,2- and 1,1-arylchlorination and bromination of unactivated α-olefins.^2a,c^ In addition, one of our laboratories applied this strategy to the palladium catalyzed 1,2-diarylation of alkenes utilizing arylstannanes as the coupling partner and oxygen as the terminal oxidant.^2*b*^ This technology was further extended to mixed diarylation reactions utilizing aryldiazonium salts and arylboronic acids for 1,1-diarylation of terminal alkenes and the 1,2-diarylations of dienes.^2d,f^ The 1,4-divinylation of isoprene was achieved using vinyl triflates and boronic acids.^2*i*^ The variety of coupling partners used to generate and intercept Mizoroki–Heck intermediates in these reports encouraged us to continue to explore the generality of this 3-component coupling platform.

The question we initially considered was that of oxidative fluorination of the σ-alkyl palladium intermediate on the basis of multiple studies describing sp^3^-C–F reductive elimination from high-valent metal species, including palladium.^4,5^ Specifically in this regard, we successfully integrated alkene difunctionalization reactions initiated *via* a Heck reaction to the enantioselective construction of sp^3^-C–F bonds,^6^ in our reported Pd-catalyzed directed enantioselective 1,2-arylfluorination of styrenes.^2*e*^ This initial report was followed by the development of methods for enantioselective 1,1-arylfluorination of protected allylamines^2*h*^ and β,β-arylfluorination of α,β-unsaturated carbonyls.^2*j*^

Although these reactions apply similar conceptual strategies, the regiochemical outcome of the transformations is dependent on substrate and conditions. The ultimate outcome of these reactions is determined by both the regioselectivity of the initial migratory insertion event, and the propensity of the palladium to migrate *via* successive β-hydride elimination and insertion events prior to oxidative functionalization. The latter suggests that oxidative alkene difunctionalization beyond the reported 1,1- and 1,2- regioselectivity should be accessible though pathways analogous to those proposed for the redox-relay Heck reaction reported by one of our laboratories.^7^ Herein, we present the development of the first Pd-catalyzed 1,3-arylfluorination reaction, including a catalytic enantioselective variant, as well as an integrated experimental, computational, and statistical analysis of the site selectivity as a function of substrate and ligand. The results of these studies shed light on the factors that govern site selectivity in the migratory insertion step, which should inform future applications of the strategies described.^8^

## Discovery and development of 1,3-arylfluorination

**Scheme 2:**
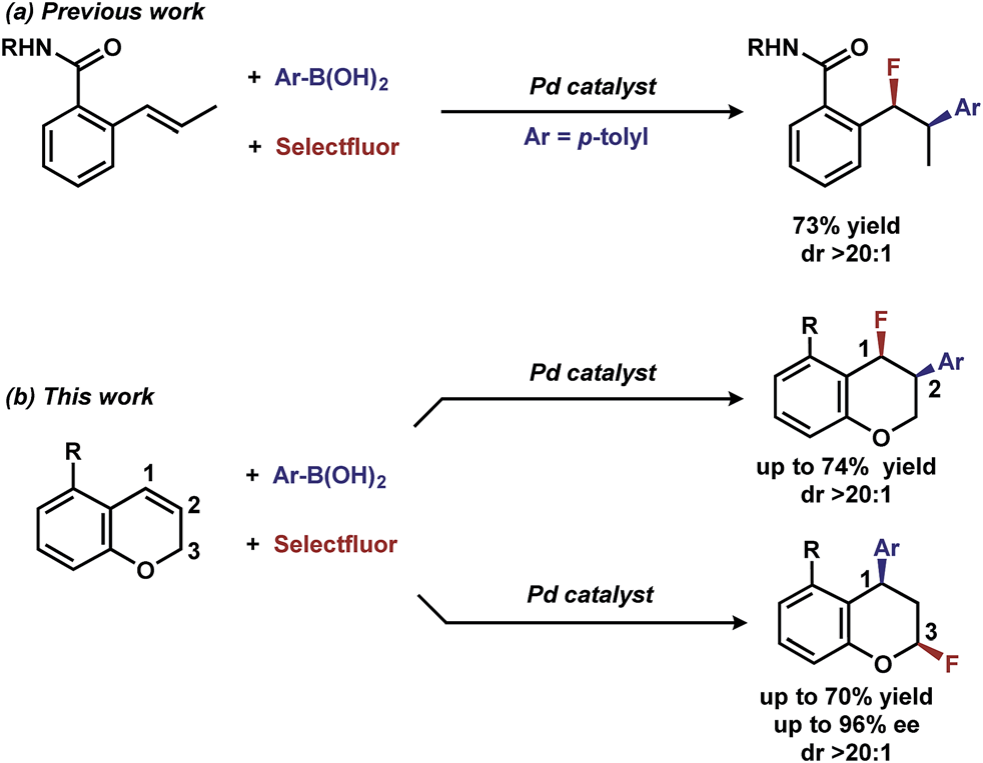
Palladium-catalyzed fluoroarylation reactions.

In our previous studies, we noted that the alkene was arylated at a single site resulting in a 2,1-fluoroarylation of a *trans*-di-substituted styrene (Scheme 2a). In light of this result, the degree of substitution and geometry of alkenes that were competent to participate in this reaction was further examined. During these studies, our attention turned to the *ortho*-carboxamide of [2*H*]-chromene (Scheme 2b, R = CONHPh) as a substrate. Under the previously optimal conditions to achieve a directed 2,1-arylfluorination process, the resulting product was observed only in a trace amount. Surprisingly, the 1,3-arylfluorination product was the major product (Scheme 2b), thereby overcoming the expected directing group bias.^2e,9^ Given this unprecedented site selectivity of the initial migratory insertion event and the impact of ligand on such selectivity (*vide infra*), we posited that this transformation would be an ideal platform to investigate the subtle factors that contribute to regioselectivity in migratory insertion step of interrupted Mizoroki–Heck reactions.

Intrigued by the 1,3-disposition of the introduced substituents, we sought to initially probe the scope of this transformation (Table 1). Using 4,4′-di-*tert*-butyl-2,2′-bipyridine **L1** as the ligand, a wide variety of boronic acids (bearing electron withdrawing and donating groups) were evaluated. Under these conditions, the 1,3 display of the introduced functional groups was conserved with a range of boronic acids (**2a–h**). Interestingly, [2*H*]-chromenes bearing different substitution patterns were either less efficient (**3–5a**) or ineffective (**5b–5d**) under these reaction conditions.

**Table 1:** 1,3-Arylfluorination of chromenes^*a*^ ^,^ ^*b*^ ^,^ ^*c*^

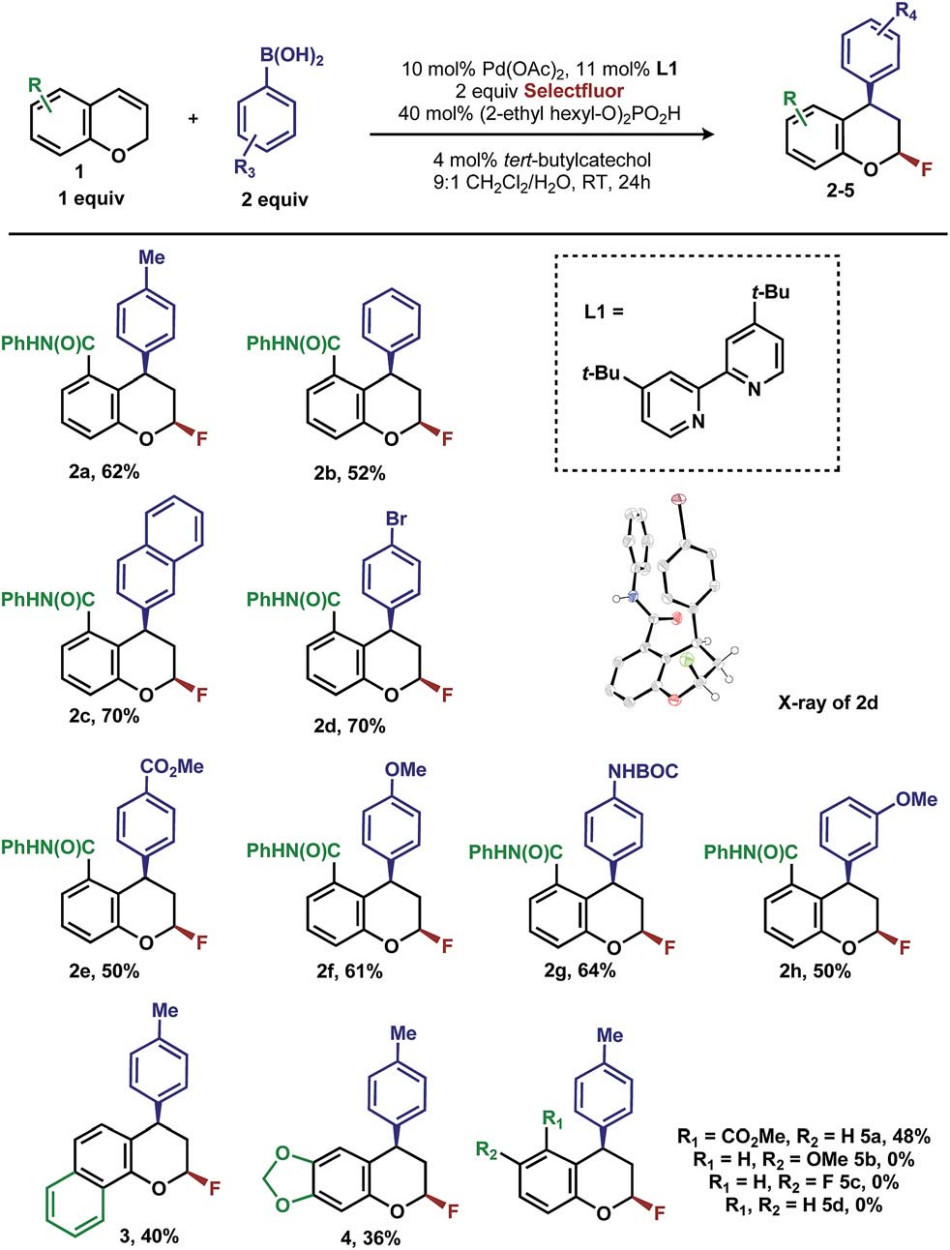

^*a*^Reactions performed on 0.10 mmol scale and 2.0 mL total volume of solvent.

^*b*^Yields refer to isolated yields.

^*c*^All samples are racemic.

Having already achieved an enantioselective 2,1-arylfluorination utilizing styrene as a substrate,^2*e*^ we anticipated that the novel 1,3-arylfluorination manifold could also be rendered enantioselective. Several commercially available and readily accessible chiral *N*,*N* ligands were evaluated (see ESI†); however, only trace amounts of the target product were observed in nearly all cases. In a similar fashion to our previous study, the most promising class of ligand for this transformation were the PyrOx class, of which (*S*)-4-*tert*-butyl-2-(2-pyridyl)oxazoline led to the formation of **2a** with the highest enantioselectivity (see ESI†). After extensive optimization (see ESI†), we identified that the addition of 1.5 equivalents of sodium fluoride^10^ and a DCE/H_2_O solvent mixture rendered the reaction selective for the 1,3-product (5 : 1 relative to the 2,1-arylfluorination product), while also maintaining a high enantioselectivity. Using these modified reaction conditions, the scope of the enantioselective reaction was explored. It should be noted that the preformed palladium(ii) complex **6** was utilized in this investigation as this aided the reproducibility.^11^ The 1,3-difunctionalized products were obtained in moderate yields and high enantiomeric excess (Table 2, **2a–2h**), when chromenes with an *ortho*-amide substituent were employed. When the corresponding ester substituent was employed in the *ortho* position, the product **5a** was formed in both lower yield and reduced enantiomeric excess. The synthetic utility of these pyranyl fluorides was further demonstrated by treating **2a** with potassium trifluoroborate salt **7** in the presence of BF_3_·etherate to form alkyne **8** in good yield and high diastereoselectivity, without erosion of the enantiomeric purity.^12^

**Table 2:** Asymmetric 1,3-arylfluorination of chromenes and diastereoselective derivatization of pyranyl fluoride^*a*^ ^,^ ^*b*^ ^,^ ^*c*^

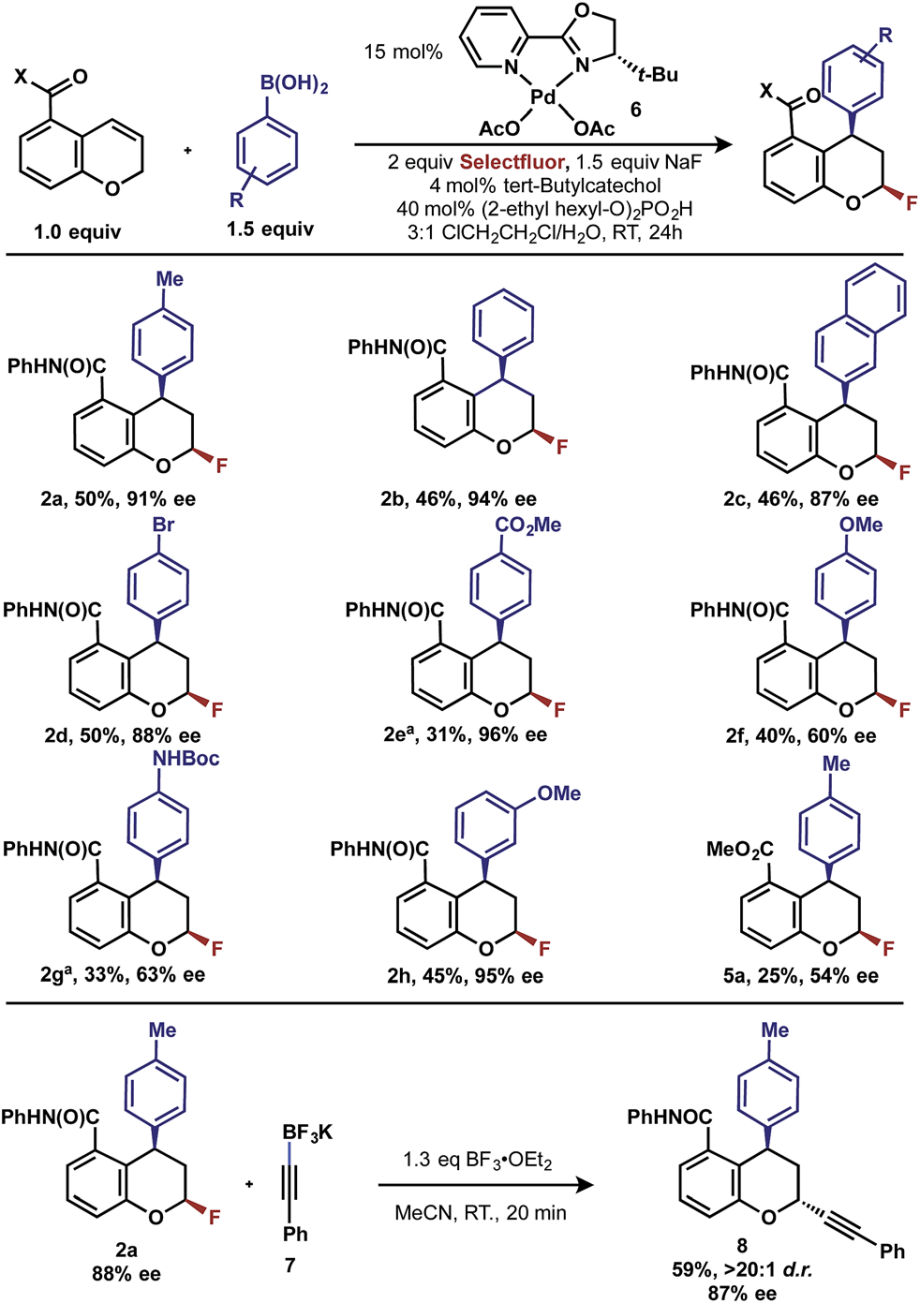

^*a*^Reactions performed on 0.10 mmol scale and 2.0 mL total volume of solvent.

^*b*^Yields refer to isolated yields.

^*c*^Enantiomeric excess determined by chiral HPLC.

During the development of the enantioselective variant of this reaction, we observed the formation of the 2,1-arylfluorination product **9** (Table 3) in significant quantities. Given the unique nature of the 1,3-fluoroarylation reaction and the apparent ligand effect on regioselectivity, we sought to better understand the origin of this divergence. We set out to determine what experimental parameters affect the site selectivity of migratory insertion, the results of which are summarized in Table 3. We have identified three variables that significantly influence the observed ratio of products **2** and **9**: choice of ligand, aryl boronic acid coupling partner, and directing group. The data was utilized in the following computational and statistical analysis to develop our mechanistic hypothesis and better our understanding the factors governing reaction performance and site selectivity.

**Table 3:** Ratio of 1,3- and 2,1-arylfluorination products (**2**/**9**) as a function of ligand, boronic acid and directing group^*a*^ ^,^ ^*b*^ ^,^ ^*c*^ ^,^ ^*d*^

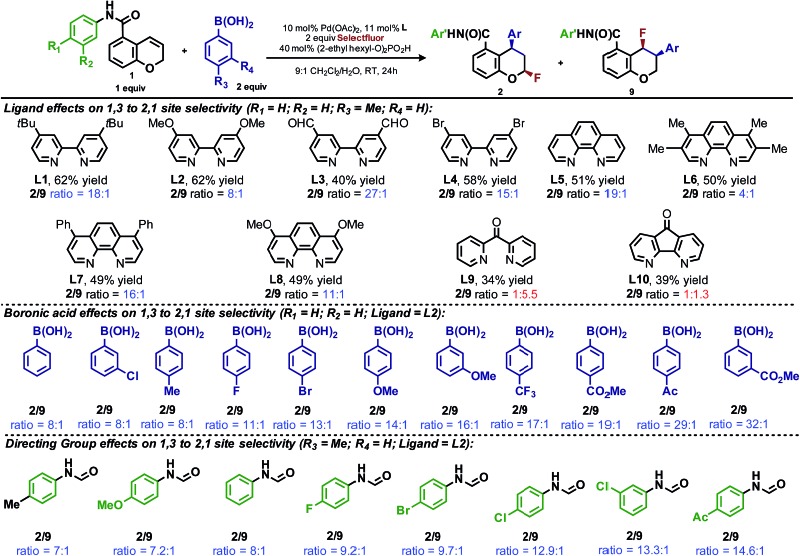

^*a*^Reactions performed on 0.10 mmol scale and 2.0 mL total volume of solvent.

^*b*^Yields and product ratios determined by ^19^F-NMR by comparison to an internal standard (4-fluorobenzoic acid).

^*c*^Yields and product ratios are the average of two runs.

^*d*^All samples are racemic.

## Ligand effects on product distribution

To elucidate the ligand features that influence the site selectivity in the arylfluorination reaction of chromenes, various ligands were evaluated in the palladium-catalyzed arylfluorination reaction of [2*H*]-chromene **1a** and *para*-tolyl boronic acid (Table 3). The results illustrate a wide variation of reaction outputs from an undiscriminating (1 : 1.3; Table 3, **L10**) to a highly selective (27 : 1; Table 3, **L3**) 1,3-arylfluorination process. Following these experiments the ground state structure of various ligated palladium complexes, PdLCl_2_, were calculated using DFT.

**Fig. 1:**
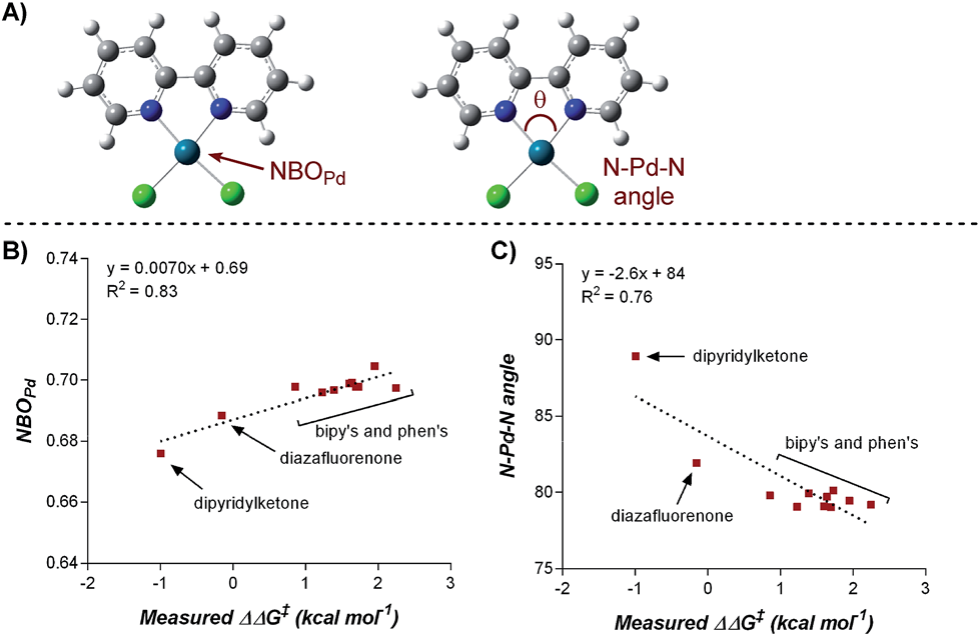
Correlation of Pd-NBO charge and ligand bite angle with regioselectivity.

Numerous parameters were gathered from the geometry optimized structures of the palladium complexes including Natural Bond Orbital (NBO) charges, N–Pd–N bite angle, Sterimol values, and IR frequencies and intensities.^13^ Through linear regression analysis, Pd NBO charge was found to correlate well with the difference in transition state energies of the two regioisomers (ΔΔ*G* ^‡^), which can be related to the log of the product ratios (Fig. 1b).^14^ As the Pd NBO charge becomes more positive, the ΔΔ*G* ^‡^ increases, which is directly correlated to greater selectivity for the 1,3-product. We conclude from this correlation that enhanced electrophilicity of the palladium center results in greater selectivity for the 1,3-arylfluorination product.

Additionally, the dipyridylketone and diazafluorenone were computed to have wider bite angles as compared to the other ligands tested, which correlates to the formation of more of the 2,1-product (Fig. 1c). This structural distortion has previously been shown to result in complex co-ordination chemistry for diazafluorenone with palladium(ii) acetate.^15*a*^ As a result, these ligands have hemi-labile behavior, suggesting they can act as both monodentate and bidentate ligands.^15*b*^ We hypothesized that this ability to act in some cases as a monodentate ligand was critical for the preferred formation of the 2,1-product. This hypothesis was further supported by the fact that when monodentate ligands, such as oxazoles and pyridines, were employed with **1a** and a variety of boronic acids exclusive formation of the 1,2-product was observed (see ESI†).

## Effect of aryl coupling partner

**Fig. 2:**
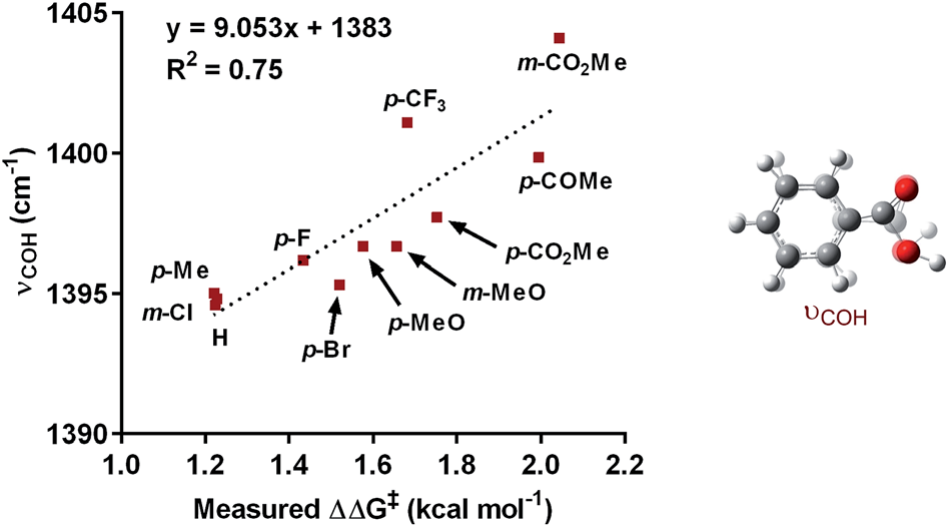
Correlation of IR stretch with regioselectivity.

The impact of the aryl boronic acid on site selectivity was explored in order to further understand the features contributing to the site of migratory insertion in these reactions. For this study, the standard reaction conditions were applied with substrate **1a** in conjunction with a range of arylboronic acids; 4,4′-dimethoxy-2-2′-bipyridine **L2** was chosen as the ligand since its use demonstrated moderate selectivity in the initial ligand screen (8 : 1; Table 3). Under the standard reaction conditions, the site selectivity observed in the arylfluorination was significantly impacted as a result of changes to the boronic acid coupling partner (Table 3). To understand what factors drive these changes, a similar correlation analysis was applied for this collection of arylboronic acids. Through the use of univariate linear regression analysis, it was found that the IR COH bending frequency of the corresponding benzoic acid correlated to the site selectivity in migratory insertion for this diverse set of boronic acids (Fig. 2).^16^ A more electron-withdrawing group (EWG) enhanced the formation of the 1,3-product. This correlation is consistent with our previous hypothesis that an increase in the cationic character of palladium complex favors formation of the 1,3-product.

## Directing group influence on regioselectivity

**Fig. 3:**
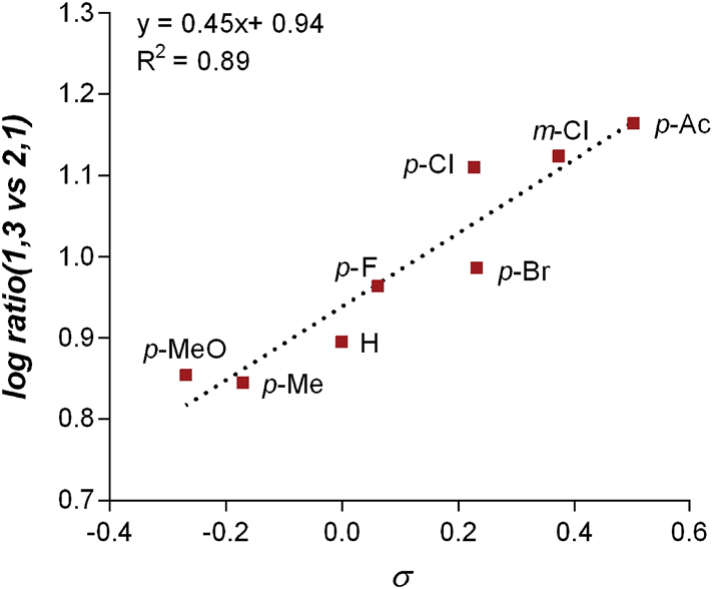
Plot of Hammett *σ*-values *versus* Measured Gibbs free energy change.

The final point for manipulation and analysis concerned the effect that the “directing group” has on the reaction outcome. To investigate this consequence, the standard reaction conditions were once again employed with 4,4′-dimethoxy-2-2′-bipyridine **L2** as the ligand. The arylfluorination reaction site selectivity was impacted through alteration of the aryl-amide substituent, suggesting that the initial olefin insertion process is also controlled by the electronics on the arylamide of the chromene substrate (Table 3). In fact, a linear correlation was identified between the Hammett *σ*-values of various aryl substituents on the amide *versus* differential transition state energies for the formation of two constitutional isomers yielded in the reaction (Fig. 3).^17^

A positive slope in the Hammett plot suggests that the electronics on the arylamide is impacting the orientation of [Pd]–Ar species *via* coordination to the metal center, thus influencing the migratory insertion pathway. In general, electron donating groups (EDG) on the arylamide decreases the selectivity for 1,3-products. We rationalized that with the use of EDG on the arylamide, coordination to the [Pd]–Ar species is more favorable, and thus the increased 2,1-product formation is a result of enhanced efficiency of coordination of the directing group.

To further probe the mechanistic divergence, we sought validation of our developing hypothesis that bifurcation occurs from the initial migratory insertion event. We assumed that the 2,1-arylfluorination product was formed in an analogous fashion to our previously reported reaction with styrenes, namely by a migratory insertion that places the palladium in the benzylic position, and the aryl-group in the homo-benzylic position, followed by C–F bond formation. For the 1,3-arylfluorination reaction, we anticipated an oxidative Heck-type mechanism would also be operative, although the possibility of alternative mechanisms, including allylic C–H palladation,^18^ was considered. We hypothesized that the 1,3-product was formed by a migratory insertion with the opposite sense of selectivity, followed by palladium migration, and C–F bond formation.

## Deuterium labeling experiments

**Scheme 3:**
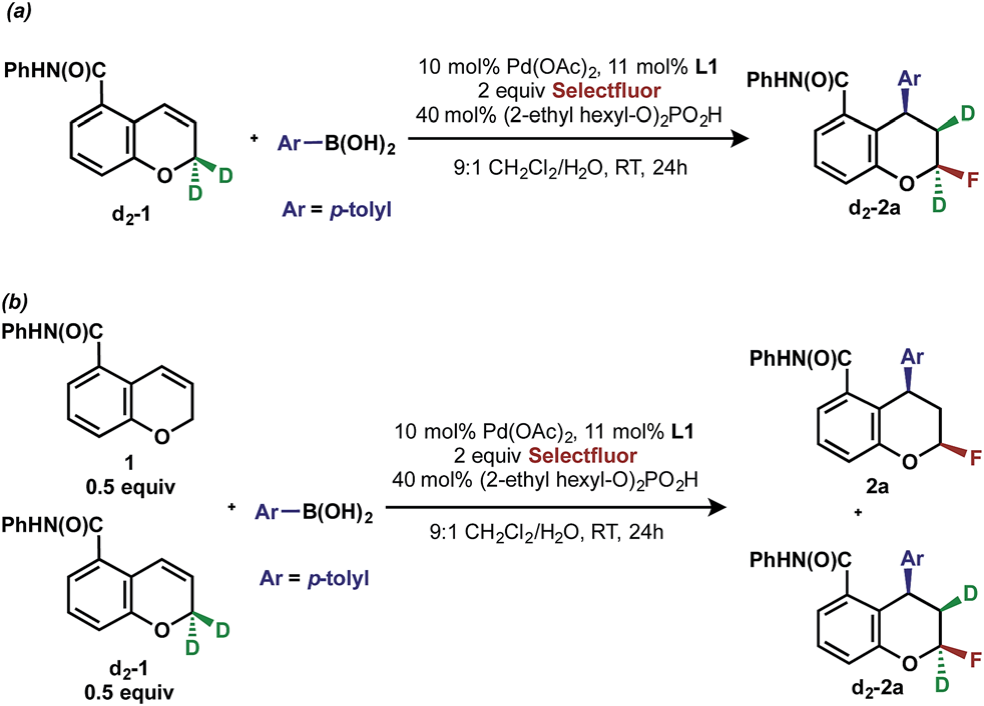
Deuterium labeling experiment with chromene **d_2_-1**.

To gather further support for this latter hypothesis, we performed a deuterium labeling experiment with chromene **d_2_-1**, (Scheme 3a). The resulting product **d_2_-2a**, in which one deuterium migrated to the adjacent carbon, was the exclusive arylfluorination product. This is possible if a [Pd]-alkyl intermediate undergoes β-hydride elimination and reinsertion events on the carbon α to oxygen, which is suggestive of an oxidative Heck-type mechanism. In a second experiment designed to understand the palladium chain walking and probe the stereochemistry of the C–F bond-forming step, a cross-over experiment with chromene **1** and deuterated substrate **d_2_-1** was performed (Scheme 3b). The observation of a 1 : 1 mixture of product **2a** with no deuterium and product **d_2_-2a** with two deuterium atoms indicated that dissociation of an intermediate olefin from a palladium hydride species and subsequent isotopic scrambling likely did not occur,^19^ thereby supporting an inner-sphere C–F bond forming reductive elimination.^4a,20^

## Mechanistic proposal

**Fig. 4:**
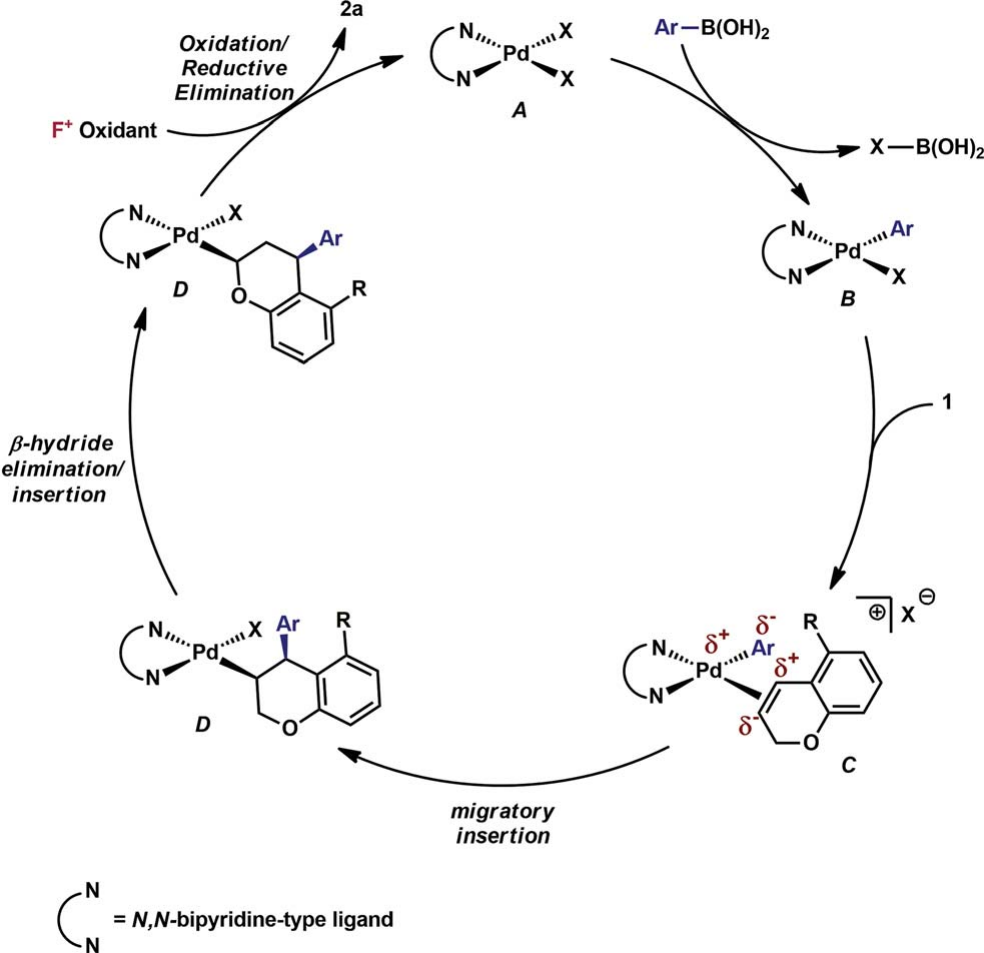
Proposed mechanisms for the formation of the 1,3-arylfluorination product.

**Fig. 5:**
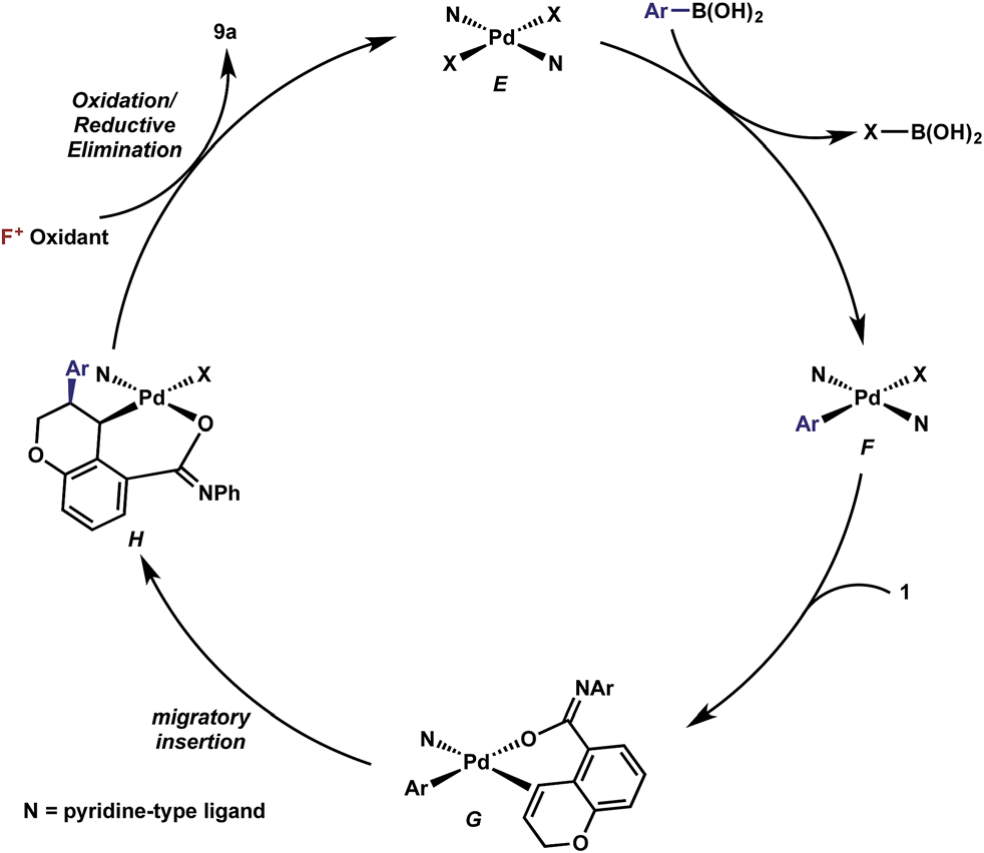
Proposed mechanisms for the formation of the 2,1-arylfluorination product.

Having established that both products likely arise from oxidative Heck-like mechanisms, we propose the following two pathways to rationalize the divergence in site selectivity outlined in Fig. 4 and 5. In the presence of a strong bidentate ligand such as 4,4′-di-*tert*-butyl-2,2′-bipyridine **L1** (Fig. 4), transmetallation with an arylboronic acid, followed by displacement of an anionic ligand with a chromene olefin, results in the formation of cationic palladium species, **C**. The site of migratory insertion is then controlled by the polarity of the alkene. When considering the chromene as a vinylogous enol-ether, insertion of the aryl group at the position α to the aromatic ring and the palladium at the β-position gives rise to the expected regiochemical outcome for an electron rich olefin and a cationic palladium species.^21^ Subsequent migration and oxidation results in the formation of the observed 1,3-product. In this case, the polarity bias of the olefin outcompetes the influence of the directing group. The correlations we found between the increased cationic character on palladium to greater selectivity for the 1,3-product corroborates this hypothesis. Additionally, when considering the pre-migratory insertion intermediate **C**, the lack of readily available coordination site in the square planar complex, the directing group would be expected to have little influence on the regioselectivity of the migratory insertion. In the presence of either hemi-labile bidentate or monodentate ligands,^22^ we alternatively propose the mechanism outlined in Fig. 5. After transmetallation of an arylboronic acid to form intermediate **F**, a labile ligand may allow for the formation of an intermediate such as **G**, in which the substrate is ligated by both the olefin and the *ortho*-carboxamide directing group. We propose that subsequently a directed migratory insertion occurs, placing the palladium α to the aromatic ring, and proximal to the *ortho*-amide directing group. Finally, oxidation and reductive elimination would afford the 2,1-arylfluorination product. In this case, the open coordination site for the directing group to occupy would be more accessible, thus enhancing the influence of the directing group. In addition, our studies also indicate that the electrophilicity of the [Pd]–Ar also has an influence on the location of the migratory insertion. The proposed intermediate **G**, is a neutral palladium species and as a result is less-electrophilic than the corresponding intermediate in the mechanism in Fig. 4; thus, the polarity of the olefin has significantly less influence in the selectivity determining step. It should be noted that similar results where significant shifts in the site of migratory insertion as a function of ligand structure in Heck reactions is precedented.^23^

**Scheme 4:**
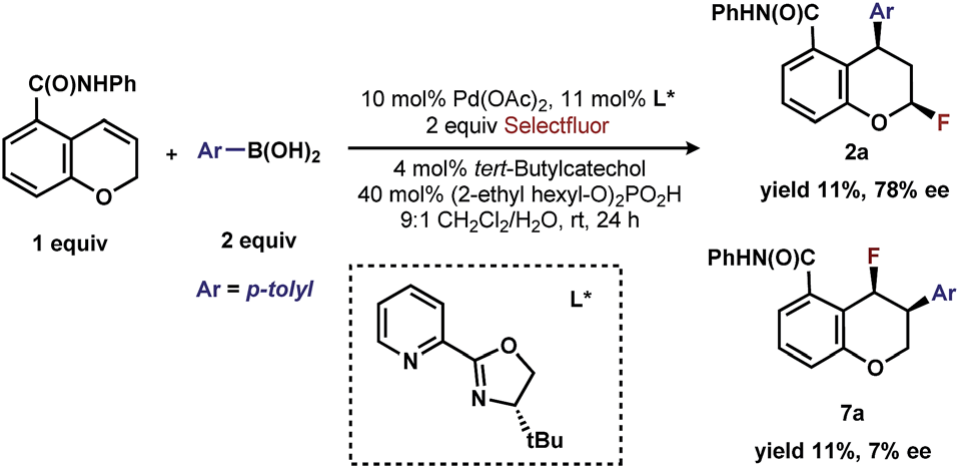
Comparison of enantioselective 1,3-arylfluorination to 2,1-arylfluorination.

Finally, the low enantioselectivity observed (7% ee; Scheme 4) for 2,1-arylfluorination products when bidentate chiral ligands are employed may also be attributed to the partial dissociation of ligand allowing directing group ligation. In contrast, high enantioselectivity was observed with the chiral bidentate PyrOx ligand L* for the formation of 1,3-arylfluorination product (Table 2). This observation is consistent with a mechanistic hypothesis discussed above in which the 1,3-arylfluorination occurs through a palladium species wherein the chiral ligands maintain their bidentate coordination.^24^

## Conclusion

We have developed a 1,3-arylfluorination of [2*H*]-chromenes; the first of the palladium-catalyzed arylhalogenation reactions that results in 1,3-relationship of the introduced substituents. In addition, we have developed an enantioselective variant of the 1,3-arylfluorination of [2*H*]-chromenes and demonstrated the utility of the enantioenriched pyranyl fluorides by further diastereoselective C–C bond formation. We have established that both the 1,3- and 2,1-products likely arise from oxidative Heck-type mechanisms that diverge at the initial migratory insertion event. Our integrated experimental, computational, and statistical analysis revealed that the identity of the ligand, the arylboronic acid coupling partner, and the directing group all affect the site of migratory insertion. The vinylogous enol ether selectivity leading to formation of the 1,3-product is enhanced by increased electrophilic character at palladium, either by the bipyridine/phenanthroline ligand or the electronics of the aryl coupling partner. Selectivity for the 1,2-product is enhanced by increased electron donating character of the amide directing group and decreased denticity of the supporting ligand. These results should help inform the design of future arylfluorination reactions, and more broadly shed light on the subtle factors, which influence the site of functionalization in interrupted Mizoroki–Heck reactions and the role of directing groups in high-valent palladium catalyzed reactions.
