# Supplementary material for: The development and mechanistic investigation of a palladium-catalyzed 1,3-arylfluorination of chromenes
Source: Chem Sci. 2017 Feb 9;8(4):2890–7. doi: 10.1039/c6sc05102b (PMC5376715; doi:10.1039/c6sc05102b)
Supplement: Supplementary file 1 [file SC-008-C6SC05102B-s001.pdf]

Electronic Supplementary Material (ESI) for Chemical Science.
This journal is © The Royal Society of Chemistry 2017

Supporting Information

For

The Development and Mechanistic Investigation of a Palladium-Catalyzed 1,3-Arylfluorination of Chromenes

Richard T. Thornbury,[a] Vaneet Saini,[b] Talita de A. Fernandes,[a,c] Celine B. Santiago,[d] Eric P. A. Talbot,[a,b] Matthew S. Sigman,[d] Jeffrey M. McKenna,[b] F. Dean Toste*[a]

[a] *Department of Chemistry, University of California, Berkeley, California 94720, United States Address here.*
[b] *Novartis Institutes for Biomedical Research, Cambridge, Massachusetts 02139, United States*
[c] *Instituto de Química, Universidade de Brasília, Campus Universitário Darcy Ribeiro, Caixa postal: 04478, 70904-970, Brasília, DF, Brazil*
[d] *Department of Chemistry, University of Utah, Salt Lake City, Utah 84112, United States*

Table of Contents

1. **General Information** **S2**
2. **Preparation and characterization of chromene substrate** **S3**
3. **Optimization, preparation characterization of arylfluorination products** **S10**
4. **Preparation and characterization of deuterated substrate** **S22**
5. **Preparation and characterization of 2-alkynyl derivative** **S24**
6. **Experimental Data Set for Statistical Analysis** **S25**
7. **Spectral data** **S28**
8. **Copies of Chiral HPLC Analysis** **S61**
9. **X-ray crystallographic data for *rac*-2d** **S73**
10. **DFT of *cis*- and *trans*- 1,3-arylfluorination Products** **S83**
11. **DFT for Statistical Analysis** **S86**
12. **References** **S102**

1. General Information

Unless otherwise noted, reagents were obtained from commercial sources and used without further purification. All reactions were carried out without rigorous exclusion of water and air and at room temperature (23 °C) except where otherwise indicated. All reactions were magnetically stirred and monitored by analytical thin layer chromatography (TLC) using Merck 60 pre-coated silica gel plates with F254 indicator. Visualization was accomplished by UV light (254 nm). Flash column chromatography was performed using ICN SiliTech 32-63 D 60Å silica gel. Commercial grade solvents were used without further purification except as indicated below. Tetrahydrofuran (THF), diethyl ether (Et_2O), dichloromethane (CH_2Cl_2), and, *N,N'*-dimethylformamide (DMF) were dried by passing commercially available pre-dried, oxygen-free formulations through activated alumina columns. ^1H NMR, ^{13}C NMR, and ^{19}F spectra were recorded on Bruker AMX-300, AVQ-400, AVB-400, DRX-500 and AV-600 spectrometers and referenced to $CDCl_3$. Tetramethylsilane was used as an internal standard for ^1H NMR (δ: 0.0 ppm), and $CDCl_3$ for ^{13}C NMR (δ: 77.23 ppm). Multiplicities are indicated by s (singlet), d (doublet), t (triplet), q (quartet), and m (multiplet). Enantiomeric excess was determined on a Shimadzu VP Series Chiral HPLC with a variable wavelength detector using chiral stationary columns. Mass spectral data were obtained from the QB3/Chemistry Mass Spectrometry Facility at the University of California, Berkeley.

2.Preparation and characterization of chromene substrates

Synthesis of methyl 3-(prop-2-yn-1-yloxy)benzoate:

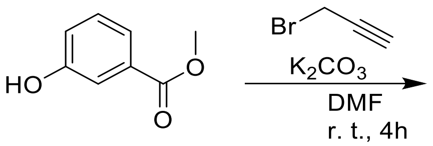

To a round bottom flask equipped with a stir bar and under positive pressure of N_2, methyl 3-hydroxybenzoate (3.04 g, 20.0 mmol 1.00 eq) and potassium carbonate (4.18 g, 30.0 mmol 1.50 eq) were added as solids and suspended in 20 ml of DMF. To the reaction mixture was added propargyl bromide (80% weight solution in toluene, 3.12 ml 28.0 mmol, 1.40 eq) was added via syringe. A slight yellow solution resulted. After stirring for 2.5 hours the reaction mixture was diluted with ethyl acetate and H2O. The organic layer was separated and washed with 1M NaOH. The organics were again separated, dried over Na2SO4, filtered, and concentrated. Residual DMF was removed in vacuo over 12 hours. The resulting yellow liquid was used crude.

^{1}H NMR (300 MHz, CDCl3) δ 7.73-7.59 (m, 2H), 7.37 (t, J = 8.0 Hz, 1H), 7.18 (ddd, J = 8.2, 2.7, 1.0 Hz, 1H), 4.74 (d, J = 2.4 Hz, 2H), 3.91 (s, 3H), 2.54 (t, J = 2.4 Hz, 1H). Spectrum is in agreement with literature report.[1]

Synthesis of methyl 2H-chromene-5-carboxylate **3**:

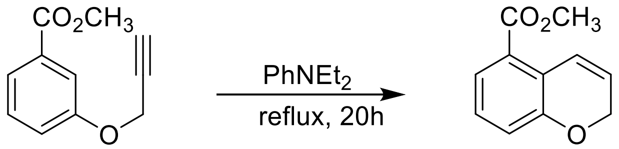

A round bottomed flask containing a stir bar and the crude methyl 3-(prop-2-yn-1- yloxy)benzoate was placed under N2 and equipped with a reflux condenser. To the flask, 20 mL of ,-diethyl aniline was added and the reaction mixture heated to 215 °C. While heating, the solution began to turn dark brown in color beyond 150 °C. The reaction mixture was heated for 24 hours, after which it was cooled to room temperature and diluted with ethyl acetate. The combined organics were washed with two times with 1 M HCl, separated, dried over Na2SO4, and concentrated to afford a brown residue. The residue was purified by silica chromatography S4 (1.5%-2.5% ethyl acetate in hexanes) affording the product (1.31 g, 34% over 2 steps) as a yellow oil.

^{1}H NMR (300 MHz, CDCl3) δ 7.48 (dd, J = 7.8, 1.3 Hz, 1H), 7.31 (dtd, J = 10.2, 1.9, 0.8 Hz, 2H), 7.14 (t, J = 8.0 Hz, 1H), 7.02 – 6.92 (m, 1H), 5.94 (dt, J = 10.2, 3.8 Hz, 1H), 4.78 (dd, J = 3.8, 1.9 Hz, 2H) 3.89 (s, 3H). Spectrum is in agreement with literature report.[1]

Synthesis of 2H-chromene-5-carboxylic acid

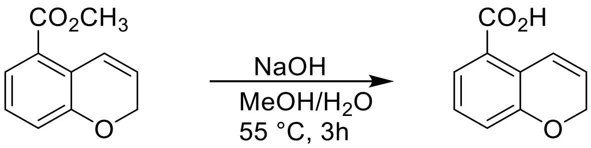

To a round bottomed flask equipped with a stir bar was added sodium hydroxide (412 mg, 10.3 mmol, 1.50 eq) was added and dissolved in 37 mL of H_2O. To the stirred solution methyl 2Hchromene-5-carboxylate (1.31 g, 1.0 eq, 6.87 mmol) was added in 17 mL of MeOH resulting in a cloudy mixture. The reaction mixture was heated to 55 °C at which point a clear yellow solution formed. After heating for the 3 hours, the contents of the flask were cooled to room temperature and the MeOH was removed by a rotary evaporator. To the remaining aqueous solution, 15 mL of 1 M HCl was added forming a colorless precipitate. The precipitate was filtered and rinsed with water on a Büchner funnel and dried in vacuo for 12 hours affording the product (846 mg, 70% yield) as a colorless powder.
^{1}H NMR (300 MHz, CDCl3) δ 7.63 (dd, J = 7.8, 1.3 Hz, 1H), 7.42 (d, J = 10.2 Hz, 1H), 7.18 (t, J = 8.0 Hz, 1H), 7.03 (d, J = 8.0 Hz, 1H), 5.97 (dt, J = 10.3, 3.8 Hz, 1H), 4.80 (dd, J = 3.8, 1.9 Hz, 2H). Spectrum is in agreement with literature report.[2]

Synthesis of N-phenyl-2H-chromene-5-carboxamide **1a**:

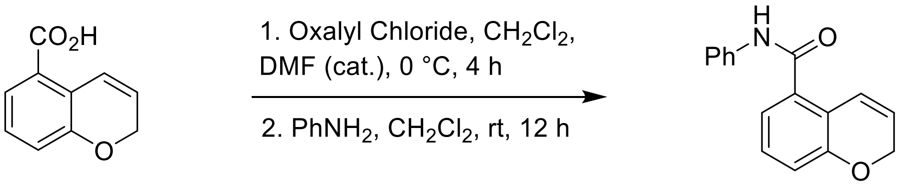

1a

To a solution of the 2-H-chromene-5-carboxylic acid (264 mg, 1.5 mmol, 1.00 equiv) in dcm (10 mL) at 0 °C was added dropwise oxalyl chloride (381 mg, 3.0 mmol, 2.0 equiv) followed by a catalytic amount of dry dmf (2 drops). The reaction was allowed to stir at rt until completion (typically 4 h). The solvent was then removed under reduced pressure to afford the corresponding crude acid chloride. Aniline (168 mg, 1.8 mmol, 1.2 equiv) was added to solution of acid chloride in dcm (20 mL). Reaction was stirred

for 12 h at rt followed by quenching with sat. $NaHCO_3$ (20 mL) solution. Organic layers were dried over $MgSO_4$, filtered, evaporated and purified by silica chromatography (10-40% EtOAc:hexanes) to give the compound **1a** as a white powder (300 mg, 80%) .

^{1}H NMR (400 MHz, $CDCl_3$) δ 7.61 (m, 3H), 7.37 (t, J = 7.9 Hz, 2H), 7.21 – 7.06 (m, 3H), 6.94 – 6.83 (m, 2H), 5.88 (dt, J = 10.1, 3.7 Hz, 1H), 4.81 (dd, J = 3.8, 1.9 Hz, 2H).

^{13}C NMR (101 MHz, $CDCl_3$) δ 166.6, 155.0, 138.1, 133.8, 129.3, 129.1, 124.9, 123.5, 122.2, 120.9, 120.1, 119.9, 118.7, 65.3.

HRMS (ESI) m/z $(M+H)^+$ calculated for $C_{16}H_{14}NO_2$: 252.1019 observed 252.1019.

Synthesis of N-(*p*-tolyl)-2H-chromene-5-carboxamide **1b**:

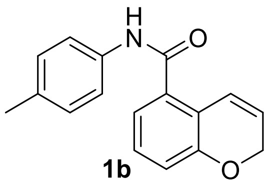

1b

The same general procedure as that for the synthesis of **1a** was followed using 2-H-chromene-5-carboxylic acid (264 mg, 1.5 mmol, 1.00 equiv), oxalyl chloride (381 mg, 3.0 mmol, 2.0 equiv), *p*-toluidine (193 mg, 1.8 mmol, 1.2 equiv), and dmf (2 drops). The compound was purified by silica chromatography (10-40% EtOAc:hexanes) to give the compound **1b** as a white powder (334 mg, 84%) .

^{1}H NMR (400 MHz, $CDCl_3$) δ 7.50 (d, *J* = 8.3 Hz, 3H), 7.21 – 7.05 (m, 4H), 6.90 (t, *J* = 8.2 Hz, 2H), 5.89 (dt, *J* = 10.1, 3.7 Hz, 1H), 4.82 (dd, *J* = 3.7, 1.8 Hz, 2H), 2.34 (s, 3H).

^{13}C NMR (101 MHz, $CDCl_3$) δ 166.5, 155.1, 135.5, 134.6, 133.9, 129.8, 129.1, 123.5, 122.3, 120.9, 120.1, 119.9, 118.6, 65.3, 21.1.

HRMS (ESI) m/z $(M+H)^+$ calculated for $C_{17}H_{15}NO_2$: 266.1176 observed: 266.1190.

Synthesis of N-(4-methoxyphenyl)-2H-chromene-5-carboxamide **1c**:

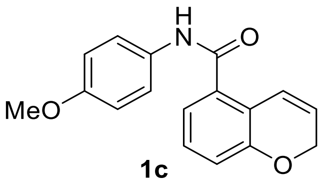

1c

The same general procedure as that for the synthesis of **1a** was followed using 2-H-chromene-5-carboxylic acid (264 mg, 1.5 mmol, 1.00 equiv), oxalyl chloride (381 mg, 3.0 mmol, 2.0 equiv), *p*-

anisidine (222 mg, 1.8 mmol, 1.2 equiv), and dmf (2 drops). The compound was purified by silica chromatography (10-40% EtOAc:hexanes) to give the compound **1c** as a white powder (312 mg, 74%) .

^{1}H NMR (400 MHz, $CDCl_3$) δ 7.52 (d, *J* = 8.9 Hz, 2H), 7.41 (s, 1H), 7.13 (dd, *J* = 18.5, 7.1 Hz, 2H), 6.91 (d, *J* = 8.8 Hz, 4H), 5.89 (d, *J* = 10.1 Hz, 1H), 4.82 (dd, *J* = 3.7, 1.8 Hz, 2H), 3.82 (s, 3H).

^{13}C NMR (101 MHz, $CDCl_3$) δ 166.5, 156.9, 155.1, 133.9, 131.1, 129.1, 123.5, 122.3, 121.9, 121.0, 119.9, 118.6, 114.5, 65.3, 55.7.

HRMS (ESI) m/z $(M+H)^+$ calculated for $C_{17}H_{16}NO_3$: 282.1125 observed: 282.1125.

Synthesis of N-(4-acetylphenyl)-2H-chromene-5-carboxamide **1d**:

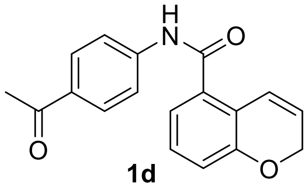

1d

The same general procedure as that for the synthesis of **1a** was followed using 2-H-chromene-5-carboxylic acid (264 mg, 1.5 mmol, 1.00 equiv), oxalyl chloride (381 mg, 3.0 mmol, 2.0 equiv), *p*-acetylaniline (243 mg, 1.8 mmol, 1.2 equiv), and dmf (2 drops). The compound was purified by silica chromatography (10-40% EtOAc:hexanes) to give the compound **1d** as a white powder (264 mg, 60%) .

^{1}H NMR (400 MHz, $CDCl_3$) δ 8.01 (d, *J* = 8.7 Hz, 2H), 7.74 (s, 2H), 7.72 (bs, 1H), 7.21 (t, *J* = 7.8 Hz, 1H), 7.17 – 7.11 (m, 1H), 6.97 (d, *J* = 8.0 Hz, 1H), 6.90 (d, *J* = 10.1 Hz, 1H), 5.94 (dt, *J* = 10.1, 3.7 Hz, 1H), 4.86 (dd, *J* = 3.7, 1.9 Hz, 2H), 2.62 (s, 3H).

^{13}C NMR (101 MHz, $CDCl_3$) δ 197.1, 166.7, 155.2, 142.3, 133.4, 133.1, 130.1, 129.2, 123.9, 122.0, 121.1, 119.9, 119.2, 119.1, 65.3, 26.7.

HRMS (ESI) m/z $(M+H)^+$ calculated for $C_{18}H_{16}NO_3$: 294.1125 observed: 294.1130.

Synthesis of N-(4-fluorophenyl)-2H-chromene-5-carboxamide **1e**:

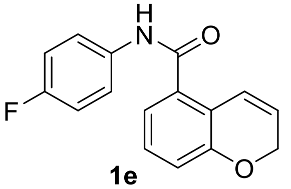

1e

The same general procedure as that for the synthesis of **1a** was followed using 2-H-chromene-5-carboxylic acid (264 mg, 1.5 mmol, 1.00 equiv), oxalyl chloride (381 mg, 3.0 mmol, 2.0 equiv), *p*-

fluoroaniline (200 mg, 1.8 mmol, 1.2 equiv), and dmf (2 drops). The compound was purified by silica chromatography (10-40% EtOAc:hexanes) to give the compound **1e** as a white powder (238 mg, 59%) .

^{1}H NMR (400 MHz, $CDCl_3$) δ 7.62 – 7.55 (m, 2H), 7.15 (t, *J* = 7.8 Hz, 1H), 7.11 – 7.03 (m, 3H), 6.97 – 6.83 (m, 3H), 5.89 (dt, *J* = 10.1, 3.7 Hz, 1H), 4.81 (dd, *J* = 3.7, 1.8 Hz, 2H).

^{13}C NMR (101 MHz, $CDCl_3$) δ 166.6, 159.7 (d, *J* = 244.0 Hz) , 155.1, 134.0 (d, *J* = 2.8 Hz), 133.5, 129.1, 123.6, 122.1, 121.9 (d, *J* = 7.9 Hz), 119.9, 118.8, 116.0 (d, *J* = 22.5 Hz), 115.9, 65.2.

HRMS (ESI) m/z $(M+H)^+$ calculated for $C_{16}H_{13}FNO_2$: 270.0925 observed: 270.0926.

Synthesis of N-(4-bromophenyl)-2H-chromene-5-carboxamide **1f**:

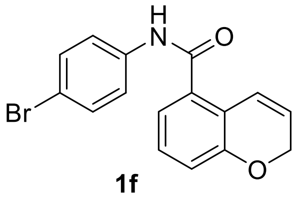

1f

The same general procedure as that for the synthesis of **1a** was followed using 2-H-chromene-5-carboxylic acid (264 mg, 1.5 mmol, 1.00 equiv), oxalyl chloride (381 mg, 3.0 mmol, 2.0 equiv), *p*-bromoaniline (310 mg, 1.8 mmol, 1.2 equiv), and dmf (2 drops). The compound was purified by silica chromatography (10-40% EtOAc:hexanes) to give the compound **1f** as a white powder (205 mg, 62%) .

^{1}H NMR (400 MHz, $CDCl_3$) δ 7.50 (q, *J* = 8.9, 8.5 Hz, 5H), 7.18 (t, *J* = 7.8 Hz, 1H), 7.10 (d, *J* = 6.8 Hz, 1H), 6.93 (d, *J* = 8.0 Hz, 1H), 6.87 (d, *J* = 10.1 Hz, 1H), 5.91 (dt, *J* = 10.1, 3.7 Hz, 1H), 4.83 (dd, *J* = 3.7, 1.9 Hz, 2H).

^{13}C NMR (101 MHz, $CDCl_3$) δ 166.5, 155.1, 137.1, 132.3, 129.1, 123.8, 122.1, 121.6, 121.0, 120.3, 119.8, 118.9, 117.5, 65.3.

HRMS (ESI) m/z $(M+H)^+$ calculated for $C_{16}H_{13}NO_2Br$: 330.0124 observed: 330.0133.

Synthesis of N-(4-chlorophenyl)-2H-chromene-5-carboxamide **1g**:

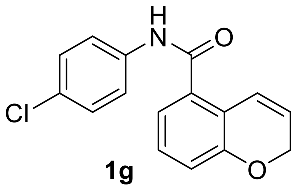

1g

The same general procedure as that for the synthesis of **1a** was followed using 2-H-chromene-5-carboxylic acid (264 mg, 1.5 mmol, 1.00 equiv), oxalyl chloride (381 mg, 3.0 mmol, 2.0 equiv), *p*-

chloroaniline (230 mg, 1.8 mmol, 1.2 equiv), and dmf (2 drops). The compound was purified by silica chromatography (10-40% EtOAc:hexanes) to give the compound **1g** as a white powder (236 mg, 55%) .

^{1}H NMR (400 MHz, $CDCl_3$) δ 7.57 (d, *J* = 8.7 Hz, 3H), 7.33 (d, *J* = 8.8 Hz, 2H), 7.16 (t, *J* = 7.8 Hz, 1H), 7.09 (d, *J* = 7.0 Hz, 1H), 6.92 (d, *J* = 8.0 Hz, 1H), 6.85 (d, *J* = 10.1 Hz, 1H), 5.89 (dt, *J* = 10.1, 3.7 Hz, 1H), 4.82 (dd, *J* = 3.7, 1.8 Hz, 2H).

^{13}C NMR (101 MHz, $CDCl_3$) δ 166.6, 155.1, 136.6, 133.4, 129.8, 129.3, 129.1, 123.7, 122.1, 121.3, 121.0, 119.9, 118.9, 65.2.

HRMS (ESI) m/z $(M+H)^+$ calculated for $C_{16}H_{13}NO_2Cl$: 286.0629 observed: 286.0633.

<u>Synthesis of N-(3-chlorophenyl)-2H-chromene-5-carboxamide **1h**:</u>

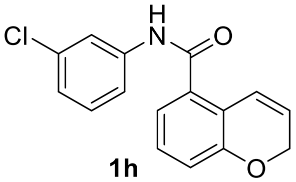

1h

The same general procedure as that for the synthesis of **1a** was followed using 2-H-chromene-5-carboxylic acid (264 mg, 1.5 mmol, 1.00 equiv), oxalyl chloride (381 mg, 3.0 mmol, 2.0 equiv), *m*-chloroaniline (230 mg, 1.8 mmol, 1.2 equiv), and dmf (2 drops). The compound was purified by silica chromatography (10-40% EtOAc:hexanes) to give the compound **1h** as a white powder (194 mg, 50%) .

^{1}H NMR (400 MHz, CDCl3) δ 7.76 (s, 1H), 7.58 (s, 1H), 7.44 (d, *J* = 7.9 Hz, 1H), 7.28 (d, *J* = 8.1 Hz, 1H), 7.20 – 7.11 (m, 2H), 7.08 (dd, *J* = 7.6, 1.1 Hz, 1H), 6.97 – 6.89 (m, 1H), 6.86 (d, *J* = 10.1 Hz, 1H), 5.90 (dt, *J* = 10.1, 3.7 Hz, 1H), 4.82 (dd, *J* = 3.7, 1.9 Hz, 2H).

^{13}C NMR (101 MHz, CDCl3) δ 166.6, 155.1, 139.1, 135.0, 133.3, 130.3, 129.1, 124.9, 123.8, 122.0, 121.0, 120.1, 119.9, 119.0, 118.0, 65.3.

HRMS (ESI) m/z (M+H)+ calculated for C16H13NO2Cl: 286.0629 observed: 286.0635**.**

<u>Synthesis of 2H-chromene **1i**:</u>

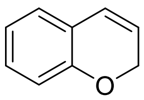

1i

Compound was prepared according to literature procedure.[4] Analytical data matches the literature.[3]

<u>Synthesis of 6-fluoro-2H-chromene **1j**:</u>

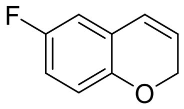

1j

Compound was prepared according to literature procedure.[4] Analytical data matches the literature.[4]

Synthesis of 6-methoxy-2H-chromene **1k**:

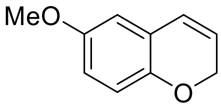

1k

Compound was prepared according to literature procedure.[4] Analytical data matches the literature.[4]

Synthesis of 6H-[1,3]dioxolo[4,5-g]chromene **1l**:

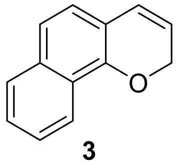

3

Compound **1l** was prepared according to literature procedure.[5]

Synthesis of 2H-benzo[h]chromene **1m**:

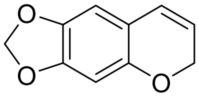

4

Compound **1m** was prepared according to literature procedure.[4]

3. Optimization, preparation characterization of arylfluorination products

Procedure A: Synthesis of racemic 1,3-arylfluorination products

Preparation of the catalyst (10 mol %):

$Pd(OAc)_2$ (2.2 mg, 0. 010 mmol, 0.10 eq) was added to a solution of 4,4'-di-tert-butyl-2,2'-bipyridine (2.9 mg, 0.011 mmol, 0.11 eq) in CH_2Cl_2 (1 ml) and the reaction mixture was stirred for 30 min.

The catalyst solution was then added to a solution of chromene **1** (25.0 mg, 0.100 mmol, 1.00 eq), aryl boronic acid (0.200 mmol, 2.00 eq), bis(2-ethylhexyl) hydrogen phosphate (12.8 mg, 0.040 mmol, 0.40 eq), Selectfluor (71.0 mg, 0.200 mmol, 2.00 eq) and *tert*-butyl catechol (0.6 mg, 0.004 mmol, 0.04 eq) in CH_2Cl_2 (0.8 ml)/water (0.2 ml). The reaction mixture was vigorously stirred for 24 h. The reaction mixture was diluted with CH_2Cl_2, dried with Na_2SO_4, filtered through celite, and concentrated under reduce pressure. The residue was purified by column chromatography to give the fluorinated products **2-5**.

Optimization of 1,3- asymmetric reaction conditions and 2,1- reaction conditions

Figure 1. Chiral ligand classes surveyed for enantioselective 1,3-fluoroarylation

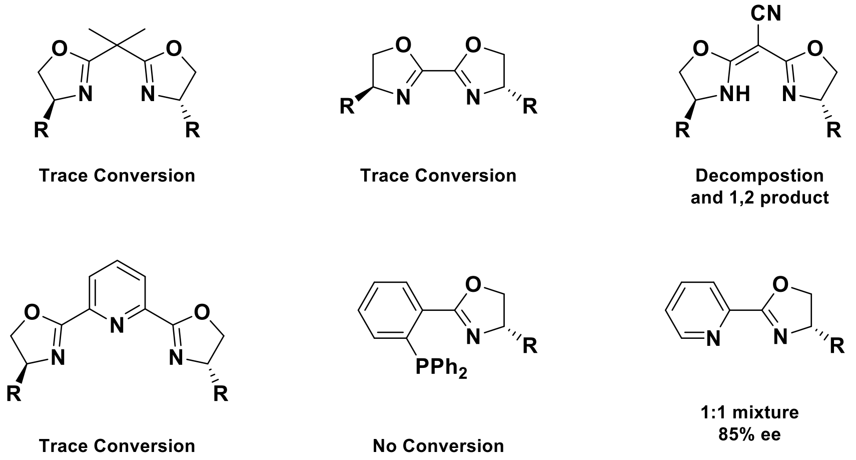

Figure 2. Ligands surveyed that afforded 1,2-fluoroarylation product

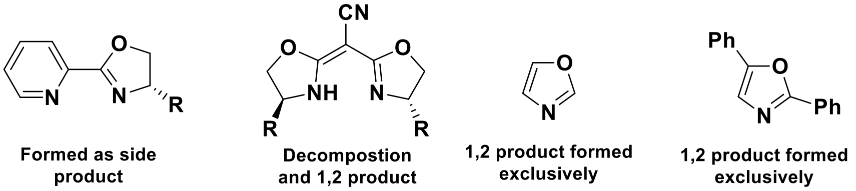

Table 1: Screening of pyridine based ligands

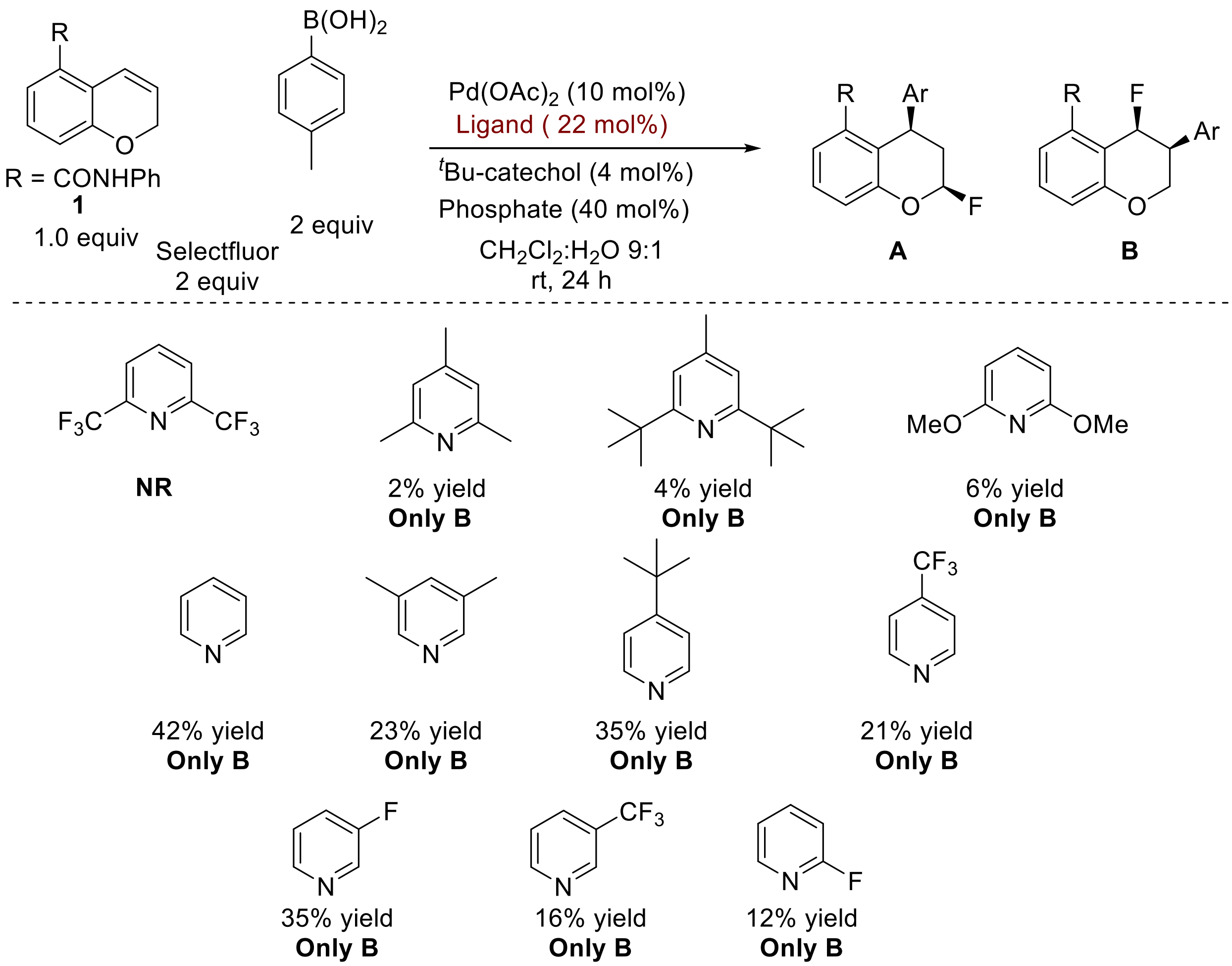

Procedure B: Synthesis of racemic 2,1-arylfluorination products

Preparation of the catalyst (10 mol %):

$Pd(OAc)_2$ (2.2 mg, 0. 010 mmol, 0.10 eq) was added to a solution of of 2,5-diphenyloxazole (4.9 mg, 0.022 mmol)in CH_2Cl_2 (1 ml) and the reaction mixture was stirred for 30 min.

The catalyst solution was then added to a solution of chromene **1** (25.0 mg, 0.100 mmol, 1.00 eq), aryl boronic acid (0.200 mmol, 2.00 eq), bis(2-ethylhexyl) hydrogen phosphate (12.8 mg, 0.040 mmol, 0.40 eq), Selectfluor (71.0 mg, 0.200 mmol, 2.00 eq) and *tert*-butyl catechol (0.6 mg, 0.004 mmol, 0.04 eq) in CH_2Cl_2 (0.8 ml)/water (0.2 ml). The reaction mixture was vigorously stirred for 24 h. The reaction mixture was diluted with CH_2Cl_2, dried with Na_2SO_4, filtered through celite, and concentrated under reduce pressure. The residue was purified by column chromatography to give the fluorinated products **9**.

Table 2. Scope of 2,1-arylfluorination

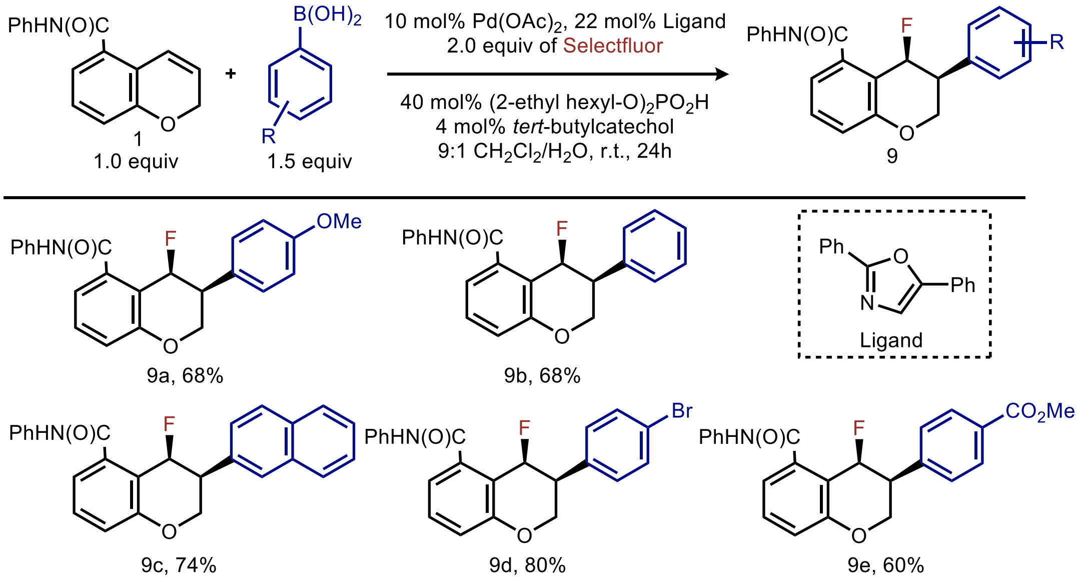

Procedure C: synthesis of enantioenriched 1,3-arylfluorination products

The palladium complex **6** was prepared as previously reported in the literature.[6]

To a round bottom flask, chromene **1** (25.0 mg, 0.100 mmol, 1.00 eq), aryl boronic acid (0.150 mmol, 1.50 eq), bis(2-ethylhexyl) hydrogen phosphate (12.8 mg, 0.040 mmol, 0.40 eq), Selectfluor (71.0 mg, 0.200 mmol, 2.00 eq), *tert*-butyl catechol (0.6 mg, 0.004 mmol, 0.04 eq), and palladium complex **5** (6.8 mg, 0.0150 mmol, 0.15 eq) were added and placed under N_2. To the flask, 1,2-dichloroethane (1.5 ml)/water (0.5 ml) was added. The reaction mixture was vigorously stirred for 24 h. The reaction mixture was diluted with CH_2Cl_2, dried with Na_2SO_4, filtered through celite, and concentrated under

reduce pressure. The residue was purified by column chromatography to give the fluorinated product. The enantiomeric excess was determined by chiral HPLC ananlysis.

TABLE 3. Optimization of Asymmetric 1,3-arylfluorination

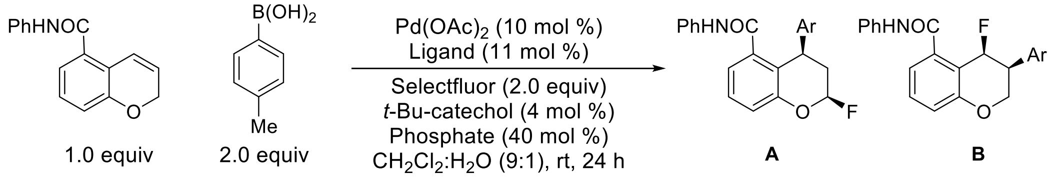

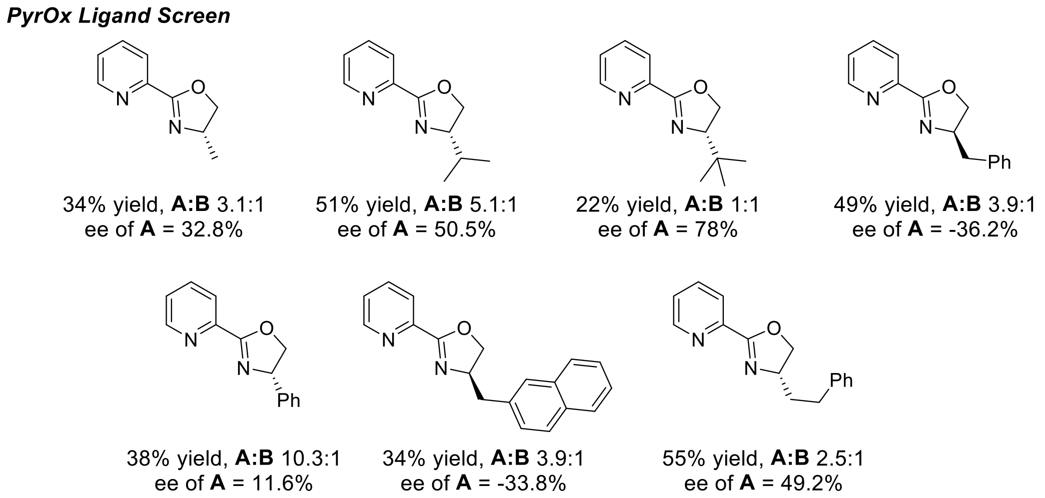

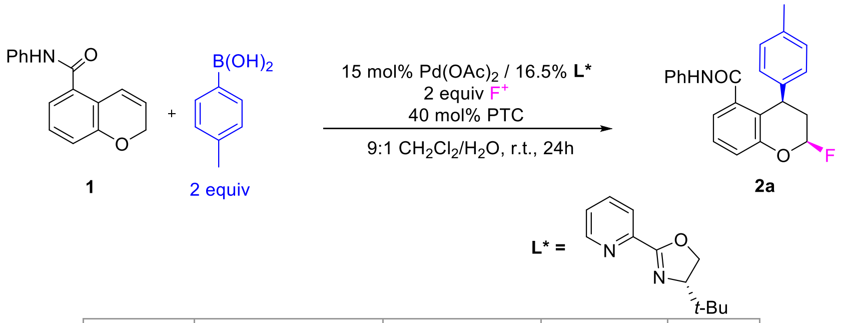

Entry	Solvent	Additive	1,3:1,2 ratio[ab]	% ee[c]
1	DCM/H_2O 9:1	-	1:1	85 %
2	DCM/H_2O 9:1	2 eq KF	5:1	80 %
3	DCM/H_2O 9:1	1 eq KF	5:1	86 %
4	DCM/H_2	0.5 eq	2:1	-

	O 9:1	KF		
**5**	DCM/H_2O 9:1	1 eq CsF	3:1[d]	-
**6**	DCM/H_2O 9:1	1 eq NaF	4:1	86 %
**7**	DCM/H_2O 9:1	1 eq NaF	>10:1[e]	-
**8**	DCE/H_2O 9:1	1 eq NaF	4:1	89 %
**9**	$CHCl_3$/H_2O 9:1	1 eq NaF	1:1	-
**10**	DCE/H_2O 3:1	1 eq NaF	5:1	89 %
**11**	**DCE/H_2O 3:1**	**1.5 eq NaF**	**5:1**[f]	**90 %**

Standard conditions: 1 equiv Chromene, 2 equiv boronic acid, 15 mol% Pd(OAc)$_2$, 16.5 mol% PyOx, 2 equiv Selectfluor, 40 mol% bis(2-ethylhexyl)hydrogenphosphate, 4 mol% *t*-butyl catechol, CH_2Cl_2/H_2O 9:1, N_2 atmosphere, 23^0 C, 24h.; (a) Reactions reach full conversion of the starting material unless otherwise noted. (b) Determined by ^{1}H and ^{19}F NMR; (c) Determined by Chiral HPLC; (d)Large amount of side product formation; (e) Reaction run at 0^0 C, trace conversion. (f) 1.5 equiv boronic acid, less side product formation.

2-fluoro-N-phenyl-4-(p-tolyl)chroman-5-carboxamide **2a**:

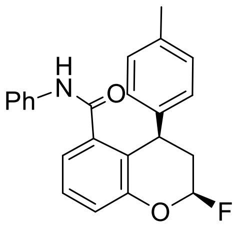

• **^{1}H NMR** (400 MHz, $CDCl_3$) δ 7.36 (t, *J* = 7.8 Hz, 1H), 7.25 - 7.14 (m, 4H), 7.08 – 6.91 (m, 7H), 6.06 (d, *J* = 55.8 Hz, 1H), 4.75 – 4.67 (m, 1H), 2.58 – 2.33 (m, 2H), 2.26 (s, 3H).

• **^{19}F NMR** (377 MHz, $CDCl_3$): -118.4

• **^{13}C NMR** (101 MHz, $CDCl_3$) δ 166.9, 151.4, 141.4, 138.3, 137.1, 136.3, 129.3, 128.7, 128.7, 128.1, 128.1, 124.5, 121.7, 120.9, 120.0, 119.7, 104.9 (d, *J*=221.5), 34.0, 33.8, 21.0.

• **HRMS** (ESI): M+H$^+$ found 362.1550; $C_{23}H_{21}FNO_2$ requires 362.1551

2-fluoro-N,4-diphenylchroman-5-carboxamide **2b**:

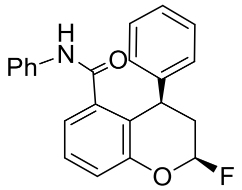

• **^{1}H NMR** (400 MHz, $CDCl_3$) δ 7.37 (t, *J* = 7.8 Hz, 1H), 7.26 – 7.00 (m, 10H), 6.99 – 6.91 (m, 2H), 6.06 (d, *J* = 55.7 Hz, 1H), 4.78 (d, *J* = 5.6 Hz, 1H), 2.60 – 2.37 (m, 2H).

• **^{19}F NMR** (377 MHz, $CDCl_3$): -118.2

• **^{13}C NMR** (101 MHz, $CDCl_3$) δ 167.0, 151.6, 144.6, 138.3, 137.1, 128.9, 128.8, 128.8, 128.3, 126.8, 124.7, 121.8, 120.8, 120.3, 119.9, 104.9 (d, *J*=221.7), 34.2, 34.0, 33.8.

• **HRMS** (ESI): $M+H^+$ found 348.1392; $C_{22}H_{19}FNO_2$ requires 348.1394

2-fluoro-4-(naphthalen-2-yl)-N-phenylchroman-5-carboxamide **2c**:

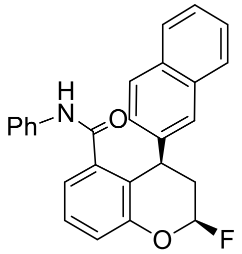

• **^{1}H NMR** (600 MHz, $CDCl_3$) δ 7.75 (d, *J* = 7.7 Hz, 1H), 7.72 (d, *J* = 8.5 Hz, 1H), 7.63 (d, *J* = 7.7 Hz, 1H), 7.51 (s, 1H), 7.44 – 7.37 (m, 3H), 7.29 – 7.27 (m, 1H), 7.25 – 7.21 (m, 2H), 6.98 (t, *J* = 7.6 Hz, 2H), 6.94 – 6.88 (m, 2H), 6.66 (d, *J* = 7.8 Hz, 2H), 6.09 (d, *J* = 55.6 Hz, 1H), 4.94 (d, *J* = 6.8 Hz, 1H), 2.67 – 2.48 (m, 2H).

• **^{19}F NMR** (377 MHz, $CDCl_3$): -118.3

• **^{13}C NMR** (151 MHz, $CDCl_3$) δ 167.1, 151.6, 141.9, 138.4, 136.8, 133.5, 132.4, 129.0 128.6, 128.4, 127.8, 127.6, 127.1, 126.6, 126.3, 125.9, 124.6, 121.7, 121.1, 120.2, 119.9, 104.9 (d, *J*=221.6), 34.4, 33.9, 33.7.

• **HRMS** (ESI): $M+H^+$ found 398.1549; $C_{26}H_{21}FNO_2$ requires 398.1551

4-(4-bromophenyl)-2-fluoro-N-phenylchroman-5-carboxamide **2d**:

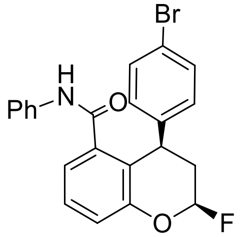

• ^{1}**H NMR** (400 MHz, CD_2Cl_2) δ 7.38 (t, J = 7.9 Hz, 1H), 7.33 – 7.15 (m, 6H), 7.14 – 7.02 (m, 3H), 6.96 (d, J = 8.2 Hz, 2H), 6.06 (d, J = 55.3 Hz, 1H), 4.85 (d, J = 6.1 Hz, 1H), 2.58 – 2.42 (m, 2H).

• ^{19}**F NMR** (377 MHz, $CDCl_3$): -118.2

• ^{13}**C NMR** (101 MHz, CD_2Cl_2) δ 166.7, 151.8, 144.1, 138.1, 137.6, 131.5, 130.5, 129.2, 129.1, 125.1, 121.4, 121.3, 120.7, 120.4, 120.2, 105.3 (d, J = 221.2 Hz), 33.7 (d, J = 21.2 Hz), 33.3.

• **HRMS** (ESI): M+H$^+$ found 426.0500; $C_{22}H_{18}BrFNO_2$ requires 426.0499

<u>methyl 4-(2-fluoro-5-(phenylcarbamoyl)chroman-4-yl)benzoate **2e**:</u>

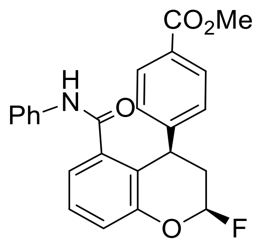

• ^{1}**H NMR** (400 MHz, CD_2Cl_2) δ 7.87 (d, J = 8.4 Hz, 2H), 7.39 (t, J = 7.8 Hz, 1H), 7.26 – 7.13 (m, 6H), 7.08 – 7.01 (m, 4H), 6.96 (s, 1H), 6.06 (dt, J = 55.2, 2.3 Hz, 1H), 4.98 (dd, J = 7.1, 2.8 Hz, 1H), 3.82 (s, 3H), 2.60 – 2.47 (m, 2H).

• ^{19}**F NMR** (377 MHz, $CDCl_3$): -118.2

• ^{13}**C NMR** (101 MHz, $CDCl_3$) 167.0, 166.9, 151.7, 149.9, 138.0, 137.0, 129.9, 129.2, 129.0, 128.6, 128.5, 125.0, 121.4, 120.8, 120.4, 120.2, 104.8 (d, J = 223.2 Hz), 52.2, 33.8, 33.6 (d, J = 21.2 Hz).

• **HRMS** (ESI): M+H$^+$ found 406.1446 ; $C_{24}H_{21}FNO_4$ requires 406.1449.

<u>2-fluoro-4-(4-methoxyphenyl)-N-phenylchromane-5-carboxamide **2f**:</u>

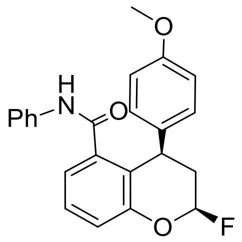

• ^{1}**H NMR** (500 MHz, $CDCl_3$)δ 7.34 (t, J = 7.8 Hz, 1H), 7.24 – 7.18 (m, 3H), 7.17 – 7.13 (m, 1H), 7.09 – 6.97 (m, 5H), 6.92 (s, 1H), 6.73 (d, J = 8.7 Hz, 2H), 6.06 (dt, J = 55.8, 2.4 Hz, 1H), 4.70 (t, J = 4.7 Hz, 1H), 3.69 (s, 3H), 2.53 – 2.36 (m, 2H).

• ^{19}**F NMR** (377 MHz, $CDCl_3$): -118.0

• ^{13}**C NMR** (101 MHz, CD_2Cl_2) δ 167.0, 158.6, 151.8, 138.5, 137.8, 136.9, 129.6, 129.0, 128.9, 124.8, 121.8, 121.7, 120.4, 119.9, 114.0, 105.5 (d, J = 221.2 Hz), 55.5, 34.2 (d, J = 20.2 Hz), 33.4.

• **HRMS**(ESI): M+H$^+$ found 378.1497; $C_{23}H_{21}FNO_3$ requires 378.1500.

tert-butyl (4-(2-fluoro-5-(phenylcarbamoyl)chroman-4-yl)phenyl)carbamate **2g**:

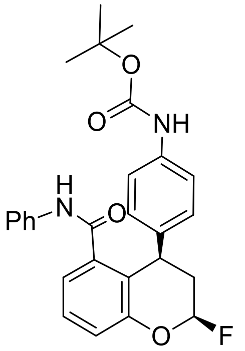

• **^{1}H NMR** (500 MHz, $CDCl_3$) δ 7.34 (t, *J* = 7.9 Hz, 1H), 7.23 – 7.12 (m, 6H), 7.08 – 7.03 (m, 3H), 7.00 (d, *J* = 8.2 Hz, 2H), 6.93 (s, 1H), 6.35 (s, 1H), 6.04 (d, *J* = 55.5 Hz, 1H), 4.72 (t, *J* = 4.8 Hz, 1H), 2.54 – 2.34 (m, 2H), 1.50 (s, 9H).

• **^{19}F NMR** (377 MHz, $CDCl_3$): -118.1

• **^{13}C NMR** (126 MHz, $CDCl_3$) δ 167.1, 152.8, 151.6, 139.2, 138.3, 137.2, 137.1, 129.1, 129.1, 128.9, 124.8, 121.7, 121.3, 120.4, 119.9, 118.8, 105.0 (d, *J* = 221.8 Hz), 34.0 (d, *J* = 20.2 Hz), 33.6, 28.5.

• **HRMS** (ESI): M+H+ found 463.2033; $C_{27}H_{28}FN_2O_4$ requires 463.2028.

2-fluoro-4-(3-methoxyphenyl)-N-phenylchromane-5-carboxamide **2h**:

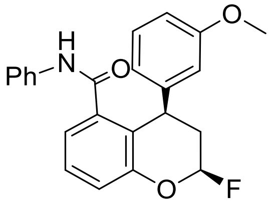

• **^{1}H NMR** (600 MHz, $CDCl_3$) δ 7.35 (t, *J* = 7.9 Hz, 1H), 7.24 – 7.18 (m, 3H), 7.15 (ddd, *J* = 7.9, 4.4, 3.2 Hz, 2H), 7.07 – 7.03 (m, 1H), 7.01 (d, *J* = 7.6 Hz, 1H), 6.98 (s, 1H), 6.05 (dt, *J* = 55.9, 2.3 Hz, 1H), 4.71 (d, *J* = 6.7 Hz, 1H), 3.66 (s, 3H), 2.61 – 2.34 (m, 2H).

• **^{19}F NMR** (377 MHz, $CDCl_3$): -117.3

• **^{13}C NMR** (151 MHz, $CDCl_3$): δ 167.1, 160.0, 151.6, 146.3, 138.5, 137.3, 129.8, 129.0, 128.9, 124.8, 121.9, 120.8, 120.2, 119.9, 114.8, 111.9, 104.9 (d, *J* = 222.0 Hz), 55.4, 34.3, 33.9 (d, *J* = 19.6 Hz).

• **HRMS** (ESI): M+H^{+} found 378.1492; $C_{23}H_{21}FNO_3$ requires 378.1500.

methyl 2-fluoro-4-(p-tolyl)chromane-5-carboxylate **3**:

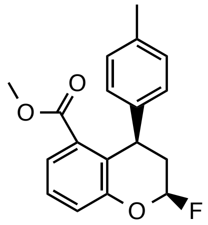

• ^{1}**H NMR** (600 MHz, $CDCl_3$) δ 7.54 (d, *J* = 7.6 Hz, 1H), 7.32 (t, *J* = 7.9 Hz, 1H), 7.20 (d, *J* = 8.3 Hz, 1H), 7.01 (d, *J* = 7.7 Hz, 2H), 6.88 (d, *J* = 7.7 Hz, 2H), 6.03 (dt, *J* = 55.6, 2.1 Hz, 1H), 5.08 (d, *J* = 7.5 Hz, 1H), 3.54 (s, 3H), 2.59 – 2.38 (m, 3H), 2.27 (s, 3H).

• ^{19}**F NMR** (377 MHz, $CDCl_3$): -119.2

• ^{13}**C NMR** (151 MHz, $CDCl_3$) δ 167.6, 151.8, 142.1, 135.6, 131.7, 129.0, 128.2, 127.8, 125.0, 124.2, 121.7, 104.9 (d, *J* = 222.0 Hz), 52.11, 34.3 (d, *J* = 19.6 Hz), 33.9, 21.25.

• **HRMS** (ESI): M+Na+ found 323.1051 ; $C_{18}H_{17}FO_3Na$ requires 323.1054.

<u>6-fluoro-8-(p-tolyl)-7,8-dihydro-6H-[1,3]dioxolo[4,5-g]chromene **4**:</u>

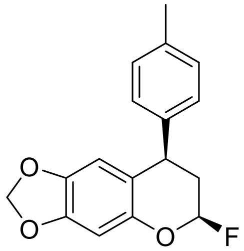

• ^{1}**H NMR** (500 MHz, CD_2Cl_2) δ 7.10 (d, *J* = 7.8 Hz, 2H), 7.02 (d, *J* = 7.9 Hz, 2H), 6.51 (s, 1H), 6.31 (d, *J* = 0.8 Hz, 1H), 5.99 (ddd, *J* = 56.1, 4.1, 2.7 Hz, 1H), 5.89 (d, *J* = 1.4 Hz, 2H), 4.07 (t, *J* = 6.2 Hz, 1H), 2.53 – 2.32 (m, 2H), 2.31 (s, 3H).

• ^{19}**F NMR** (377 MHz, CD_2Cl_2): -120.5

• ^{13}**C NMR** (126 MHz, CD_2Cl_2) δ 147.7, 146.1, 143.2, 141.7, 136.6, 129.4, 128.51 (d, *J* = 2.5 Hz), 116.5, 108.8, 106.1 (d, *J* = 220.5 Hz), 101.8, 99.0, 37.2, 35.2 (d, *J* = 21.4 Hz), 21.1.

• **HRMS** (EI): M+ found 286.1007 ; $C_{17}H_{15}FO_3$ requires 286.1005.

<u>2-fluoro-4-(p-tolyl)-3,4-dihydro-2H-benzo[h]chromene **5**:</u>

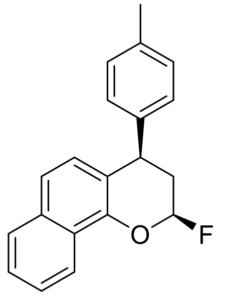

• **^{1}H NMR** (500 MHz, CD_2Cl_2) δ 8.31 – 8.21 (m, 1H), 7.81 (dd, *J* = 7.2, 2.1 Hz, 1H), 7.53 (ddt, *J* = 10.7, 7.0, 3.5 Hz, 2H), 7.44 (d, *J* = 8.5 Hz, 1H), 7.10 (d, *J* = 7.8 Hz, 2H), 7.02 (m, 3H), 6.36 – 6.17 (m, 1H), 4.34 (dd, *J* = 7.4, 4.3 Hz, 1H), 2.73 – 2.50 (m, 2H), 2.31 (s, 3H).

• **^{19}F NMR** (377 MHz, CD_2Cl_2): -121.4

• **^{13}C NMR** (126 MHz, CD_2Cl_2) δ 146.4, 141.8, 136.5, 134.0, 129.3, 128.7, 128.0, 127.9, 126.7, 126.3, 125.2, 121.9, 121.7, 118.2, 106.1 (d, *J* = 219.2 Hz), 37.3, 35.1 (d, *J* = 20.2 Hz), 21.1.

• **HRMS** (EI): M+ found 292.1264 ; $C_{20}H_{17}FO$ requires 292.1263.

<u>4-fluoro-N-phenyl-3-(p-tolyl)chroman-5-carboxamide **9a**:</u>

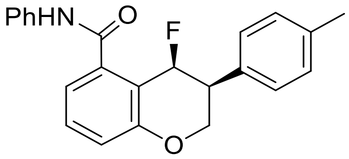

• **^{1}H NMR** (500 MHz, $CDCl_3$) δ 8.01 (br d, *J* = 5.6 Hz, 1H), 7.60 (d, *J* = 7.6 Hz, 2H), 7.44 (td, *J* = 8.2, 2.1 Hz, 1H), 7.36 – 7.31 (m, 3H), 7.22 – 7.16 (m, 4H), 7.14 (dd, *J* = 10.6, 4.2 Hz, 1H), 7.09 (d, *J* = 8.3 Hz, 1H), 5.96 (d, *J* = 50.6 Hz, 1H), 4.63 (ddd, *J* = 12.8, 10.7, 1.9 Hz, 1H), 4.40 (dd, *J* = 10.6, 4.1 Hz, 1H), 3.34 (dddd, *J* = 33.2, 12.9, 3.9, 2.1 Hz, 1H), 2.36 (s, 3H).

• **^{19}F NMR** (377 MHz, $CDCl_3$): -158.1

• **^{13}C NMR** (126 MHz, $CDCl_3$) δ 166.1, 139.1, 137.7, 137.6, 133.0, 131.8, 129.5, 129.4, 129.2, 129.1, 128.4, 128.4, 125.1, 124.7, 121.0, 120.9, 120.0, 120.0, 119.9, 84.7 (d, J=172.0), 64.1 (d, J=3.5), 42.6 (d, J=19.6), 21.1.

• **HRMS** (EI): M+ found 361.1480, $C_{23}H_{20}FNO_2$ requires 361.1481

4-fluoro-N,3-diphenylchroman-5-carboxamide **9b**:

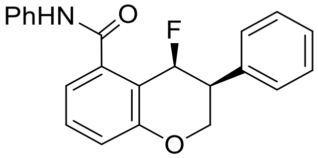

• [1]**H NMR** (600 MHz, $CDCl_3$) δ 8.03 (br s, 1H), 7.61 (d, *J* = 7.8 Hz, 2H), 7.45 (td, *J* = 8.2, 1.7 Hz, 1H), 7.40 – 7.30 (m, 8H), 7.14 (t, *J* = 7.4 Hz, 1H), 7.10 (d, *J* = 8.3 Hz, 1H), 6.00 (d, *J* = 50.6 Hz, 1H), 4.70 – 4.62 (m, 1H), 4.43 (dd, *J* = 10.6, 4.0 Hz, 1H), 3.37 (dd, *J* = 33.1, 11.5 Hz, 1H).

• [19]**F NMR** (377 MHz, $CDCl_3$): -158.3

• [13]**C NMR** (151 MHz, $CDCl_3$) δ 166.2, 155.1, 155.1, 139.2, 137.8, 136.2, 131.9, 131.8, 129.2, 128.9, 128.6, 128.6, 128.0, 124.8, 121.0, 121.0, 120.1, 84.6 (d, J=171.8), 64.1 (d, J=2.7), 43.1 (d, J=19.3).

• **HRMS** (EI): M+ found 347.1319, $C_{22}H_{18}FNO_2$ requires 347.1322

4-fluoro-3-(naphthalen-2-yl)-N-phenylchroman-5-carboxamide **9c**:

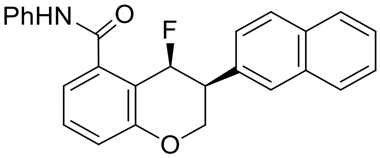

• [1]**H NMR** (400 MHz, $CDCl_3$) δ 8.06 (br s, 1H), 7.89 – 7.81 (m, 3H), 7.77 (s, 1H), 7.60 (d, *J* = 7.9 Hz, 2H), 7.53 – 7.40 (m, 4H), 7.32 (dd, *J* = 15.2, 7.5 Hz, 3H), 7.17 – 7.08 (m, 2H), 6.09 (d, *J* = 50.6 Hz, 1H), 4.77 (t, *J* = 11.7 Hz, 1H), 4.53 (dd, *J* = 10.6, 3.7 Hz, 1H), 3.53 (dd, *J* = 33.0, 11.8 Hz, 1H).

• [19]**F NMR** (377 MHz, $CDCl_3$): -157.8

• [13]**C NMR** (151 MHz, $CDCl_3$) δ 166.2, 155.1, 155.1, 139.2, 137.8, 133.6, 133.5, 132.9, 131.9, 131.9, 129.2, 128.6, 128.0, 127.8, 127.5, 127.5, 126.5, 126.3, 124.8, 121.1, 120.1, 116.5, 116.4, 84.6 (d, J=171.9), 64.2, 43.2 (d, J=19.4).

• **HRMS** (ESI): $M+H^+$ found 398.1549; $C_{26}H_{21}FNO_2$ requires 398.1551

3-(4-bromophenyl)-4-fluoro-N-phenylchroman-5-carboxamide **9d**:

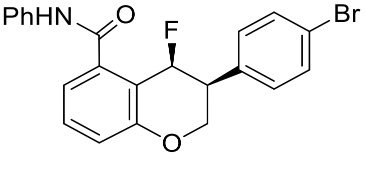

• **^{1}H NMR** (400 MHz, CD_2Cl_2) δ 7.93 (s, 1H), 7.58 (d, *J* = 7.5 Hz, 2H), 7.53 – 7.42 (m, 3H), 7.40 – 7.27 (m, 3H), 7.22 (d, *J* = 8.2 Hz, 2H), 7.17 – 7.09 (m, 2H), 6.00 (d, *J* = 50.7 Hz, 1H), 4.58 (t, *J* = 11.5 Hz, 1H), 4.39 (d, *J* = 9.7 Hz, 1H), 3.40 (dd, *J* = 32.4, 11.8 Hz, 1H).

• **^{19}F NMR** (377 MHz, $CDCl_3$): -158.7

• **^{13}C NMR** (101 MHz, CD_2Cl_2) δ 166.2, 155.3, 139.3, 138.2, 135.8, 132.1, 132.0, 131.7, 130.7, 129.4, 125.0, 122.0, 120.9, 120.2, 116.7 (d, *J* = 19.2 Hz), 84.3 (d, *J* = 172.7 Hz), 64.1, 42.8 (d, *J* = 20.2 Hz).

• **HRMS** (ESI): $M+H^+$ found 426.0500; $C_{22}H_{18}BrFNO_2$ requires 426.0499

methyl 4-(4-fluoro-5-(phenylcarbamoyl)chroman-3-yl)benzoate **9e**:

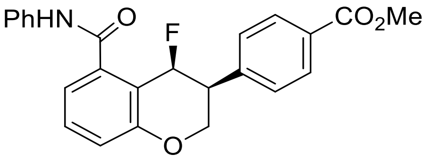

• **^{1}H NMR** (400 MHz, CD_2Cl_2) δ 8.01 (d, *J* = 8.4 Hz, 2H), 7.93 (bs, 1H), 7.58 (d, *J* = 7.8 Hz, 2H) 7.50 – 7.39 (m, 3H), 7.35 – 7.26 (m, 3H), 7.12 (dd, *J* = 18.8, 7.9 Hz, 2H), 6.07 (d, *J* = 50.7 Hz, 1H), 4.64 (t, *J* = 11.8 Hz, 1H), 4.44 (dd, *J* = 10.6, 3.4 Hz, 1H), 3.88 (s, 3H), 3.47 (dd, *J* = 32.3, 12.3 Hz, 1H).

• **^{19}F NMR** (377 MHz, $CDCl_3$): -158.8

• **^{13}C NMR** (101 MHz, CD_2Cl_2) δ 166.9, 166.2, 155.4, 141.8, 139.3, 138.2, 132.1, 130.1, 129.4, 129.0, 125.0, 120.9, 120.2, 120.1, 116.7 (d, *J* = 19.2 Hz), 84.2 (d, *J* = 172.7 Hz), 64.1, 52.4, 43.3 (d, *J* = 19.2 Hz).

• **HRMS** (ESI): $M+H^+$ found 406.1445 ; $C_{24}H_{21}FNO_4$ requires 406.1449.

4. Preparation and characterization of deuterated substrates

<u>Synthesis of (1,1-2H$_2$)-1-bromoprop-2-yne</u>

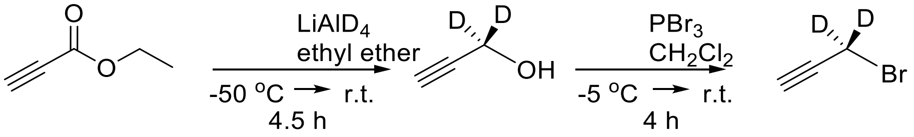

The deuteride alkyne was synthesized as described by Nag *et al.*[7]

<u>Synthesis of (2,2-2H$_2$)-N-phenyl-2H-chromene-5-carboxamide</u>

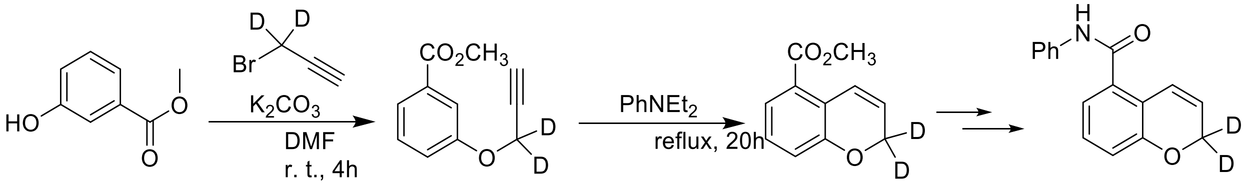

The deuterated chromene was synthesized via an analogous procedure as the non-deuterated substrate using the d$_2$-propargyl bromide.

<u>N-phenyl-2H-chromene-2,2-d2-5-carboxamide **d$_2$-1**:</u>

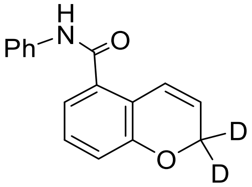

• **^{1}H NMR** (400 MHz, CHCl$_3$) δ 7.61 (d, *J* = 8.1 Hz, 3H), 7.37 (t, *J* = 7.8 Hz, 2H), 7.21 – 7.06 (m, 3H), 6.89 (dd, *J* = 12.1, 9.0 Hz, 2H), 5.87 (d, *J* = 10.1 Hz, 1H).

• **^{13}C NMR** (101 MHz, CDCl$_3$) δ 166.6, 155.0, 138.1, 133.8, 129.3, 129.0, 124.9, 123.3, 122.3, 120.9, 120.1, 119.9, 118.7.

• **HRMS** (ESI): M+H$^+$ found 254.1146; $C_{16}H_{12}{}^2H_2NO_2$ requires 254.1145.

<u>2-fluoro-N-phenyl-4-(p-tolyl)chromane-2,3-d2-5-carboxamide **d$_2$-2a**:</u>

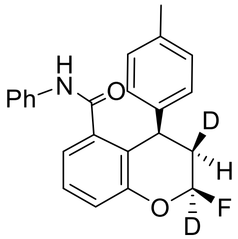

• **^{1}H NMR** (500 MHz, $CDCl_3$) δ 7.35 (t, *J* = 7.9 Hz, 1H), 7.19 (m, 4H), 7.04 (m, 3H), 7.00 – 6.94 (m, 4H), 6.92 (s, 1H), 4.69 (d, *J* = 7.5 Hz, 1H), 2.42 (dd, *J* = 38.4, 7.6 Hz, 1H), 2.25 (s, 3H).

• **^{19}F NMR** (377 MHz, $CDCl_3$): -118.5

• **^{13}C NMR** (126 MHz, $CDCl_3$) δ 167.1, 151.6, 141.6, 138.4, 137.3, 136.5, 129.5, 128.9, 128.8, 128.3, 124.7, 121.9, 121.1, 120.2, 119.8, 33.9, 21.2.

• **HRMS** (ESI): $M+H^+$ found 364.1675; $C_{23}H_{19}{}^{2}H_2FNO_2$ requires 364.1676.

5. Preparation and characterization of 2-alkynyl derivatives

N-phenyl-2-(phenylethynyl)-4-(p-tolyl)chromane-5-carboxamide **8**:

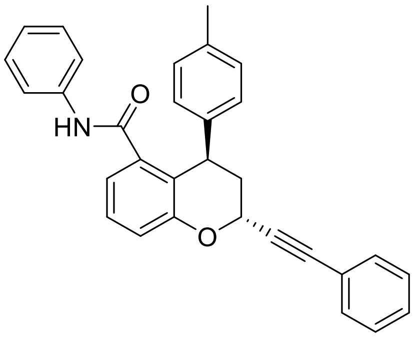

Procedure:

An adapted procedure from the literature was used.[7] 2-pyranyl fluoride **2a** (17.8 mg, 0.050 mmol, 1.0 eq) and trifluoroborate salt **7** (12.5 mg, 0.060 mmol, 1.2 eq) were added to a 1 dram vial with a septum cap and placed under an atmosphere of N_2. The solids were dissolved in 0.3 mL of dry acetonitrile and stirred. To the vial, boron trifluoride etherate (8 μl, 0.065 mmol, 1.3 eq) was added via syringe. A yellow solution resulted. The solution was stirred for 20 minutes. The contents of the vial were diluted with CH_2Cl_2 and washed 2 times with a saturated solution of $NaHCO_3$. The organic layer was separated, dried over Na_2SO_4, and concentrated. The crude solid was purified by silica chromatography (15:1 pentane/ ethyl acetate) to afford **8** as a colorless powder (13 mg, 59%).

• **^{1}H NMR** (600 MHz, $CDCl_3$) δ 7.44 (dd, J = 7.9, 1.7 Hz, 2H), 7.35 – 7.27 (m, 4H), 7.20 (dd, J = 8.4, 7.3 Hz, 2H), 7.14 (d, J = 8.0 Hz, 2H), 7.10 (d, J = 7.8 Hz, 2H), 7.08 – 7.03 (m, 1H), 7.03 – 6.96 (m, 4H), 6.92 (s, 1H), 5.04 (dd, J = 11.0, 2.5 Hz, 1H), 4.74 (dd, J = 5.3, 3.3 Hz, 1H), 2.52 (ddd, J = 13.8, 11.0, 5.3 Hz, 1H), 2.32 (t, J = 2.9 Hz, 1H), 2.30 (s, 3H).

• **^{13}C NMR** (151 MHz, $CDCl_3$) δ 167.3, 154.8, 142.3, 138.3, 137.5, 136.9, 132.1, 129.8, 128.9, 128.9, 128.7, 128.6, 128.5, 124.6, 122.3, 120.6, 120.6, 120.0, 119.7, 86.8, 86.1, 63.0, 37.6, 37.0, 21.2.

• **HRMS** (ESI): M+Na+ found 466.1778; $C_{31}H_{25}NO_2Na$ requires 466.1785.

6. Experimental Data Set for Statistical Analysis

Table 4: Screening of sp^2-Nitrogen based bidentate ligands

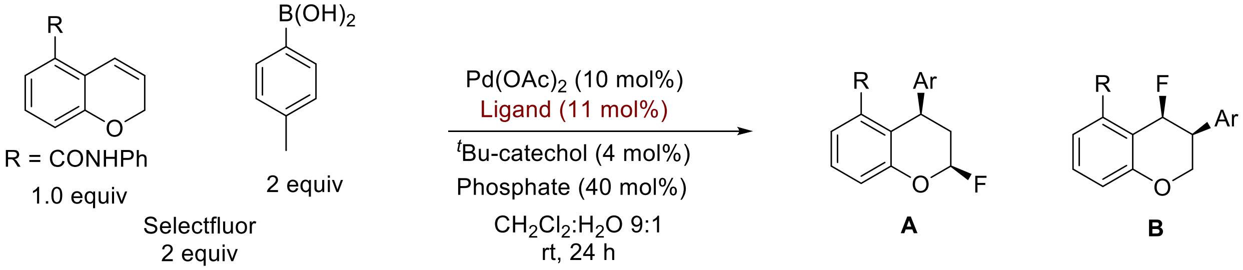

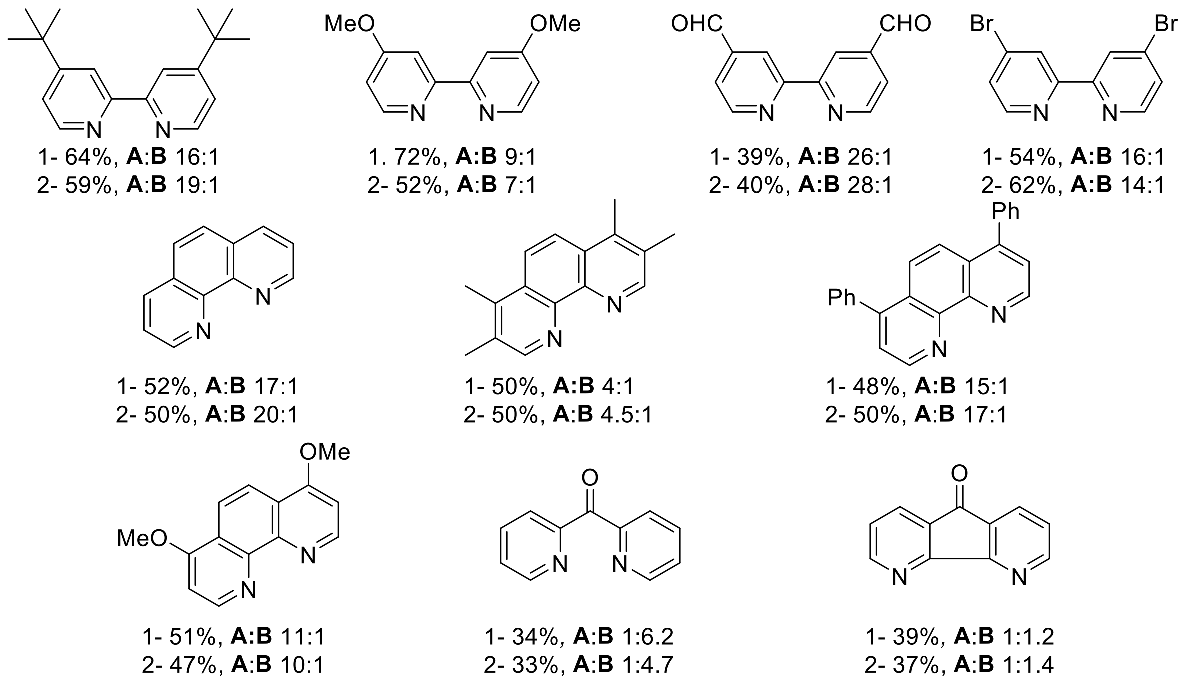

Procedure D: $Pd(OAc)_2$ (2.2 mg, 0. 010 mmol, 0.10 equiv) was added to a solution of Ligand (0.011 mmol, 0.11 equiv) in CH_2Cl_2 (1 ml) and the reaction mixture was stirred for 30 min. The catalyst solution was then added to a solution of chromene **1** (25.0 mg, 0.100 mmol, 1.00 equiv), *p*-tolyl phenylboronic acid (27 mg, 0.200 mmol, 2.00 equiv), bis(2-ethylhexyl) hydrogen phosphate (12.8 mg, 0.040 mmol, 0.40 equiv), Selectfluor (71.0 mg, 0.200 mmol, 2.00 equiv) and *tert*-butyl catechol (0.6 mg, 0.004 mmol, 0.04 equiv) in CH_2Cl_2 (0.8 ml)/water (0.2 ml). The reaction mixture was vigorously stirred for 24 h. The reaction mixture was diluted with CH_2Cl_2, dried with Na_2SO_4, filtered through celite, and concentrated under reduce pressure. Then, a known amount of 4-fluorobenzoic acid as an internal standard was added to the crude extract. Yield and regioselectivity was determined using ^{19}F NMR (CD_2Cl_2, 400 MHz, d1=10). The reaction was repeated twice and results obtained are described in Table

2. *Note:* In the above Table 1- and 2-represents first and second run, respectively. The percentage figure represents total yield (A+B) of the reaction.

Table 5: Screening of various boronic acids

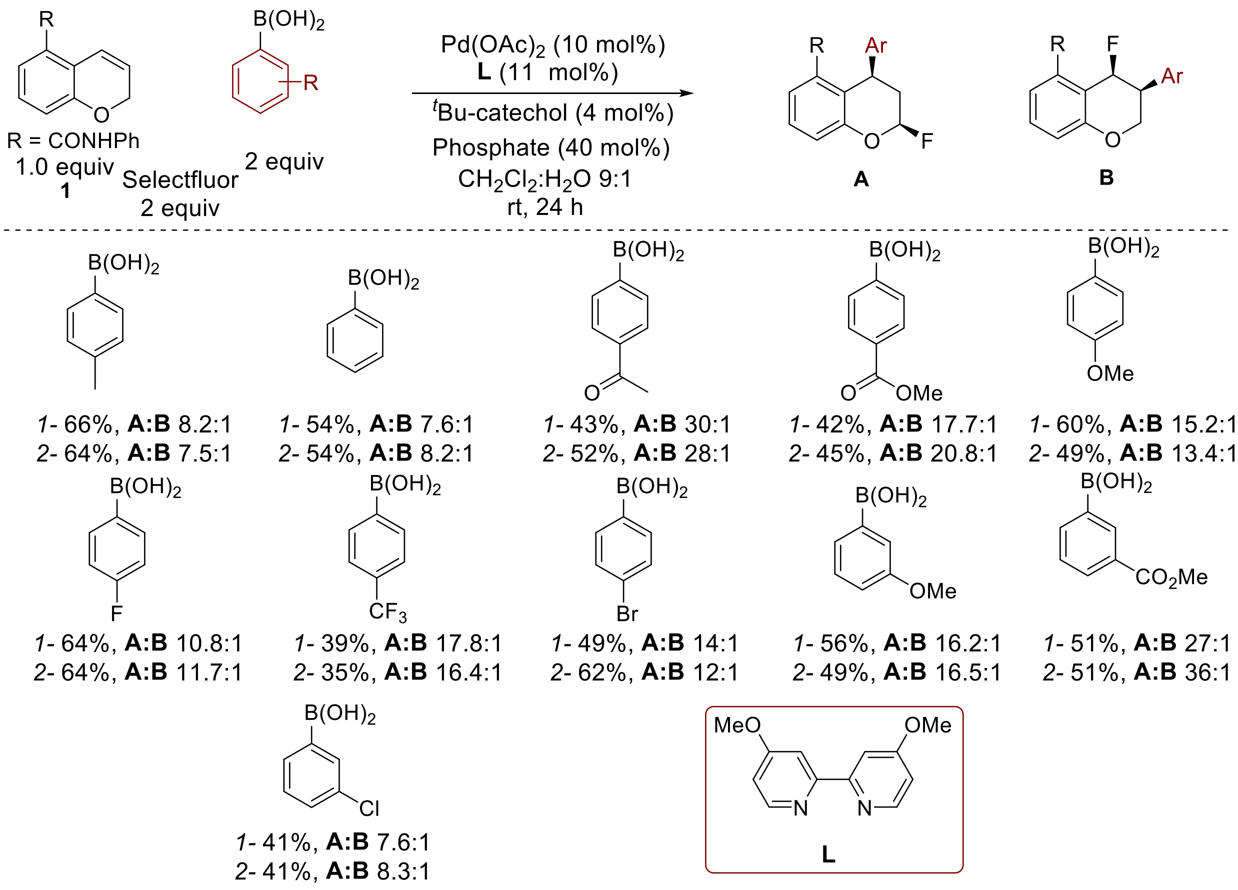

Procedure: The general **procedure D** was followed with the following modification. Ligand **L** (0.011 mmol, 0.11 equiv) and different boronic acids (0.022 mmol, 0.22 equiv) as described in the Table 4 were used. The yields and selectivities were obtained by ^{19}F NMR using 4-fluorobenzoic acid as an internal standard and are described below each substrate in Table 4.

Table 6: Screening of different chromene substrates for Hammett plot

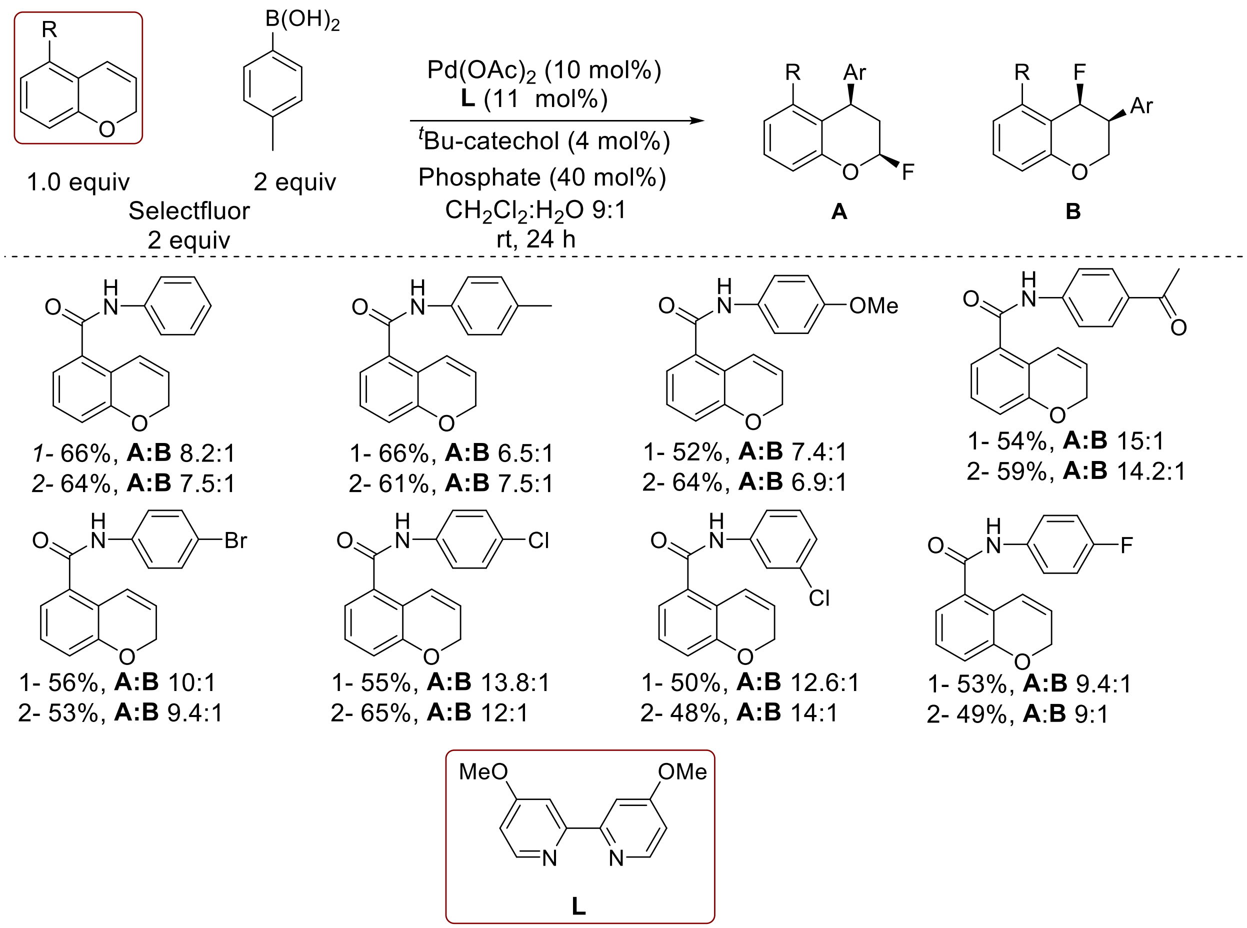

Procedure: The general **procedure D** was followed with the following modification. Ligand **L** and the chromene substrates as shown in the table above were used and the corresponding yield and selectivity obtained is described below each substrate. The yields and selectivities were obtained by ^{19}F NMR using 4-fluorobenzoic acid as an internal standard. The product formation was later confirmed by LCMS.

7. Spectral data

^{1}H NMR (400 MHz, $CDCl_3$) of methyl 3-(prop-2-yn-1-yloxy)benzoate

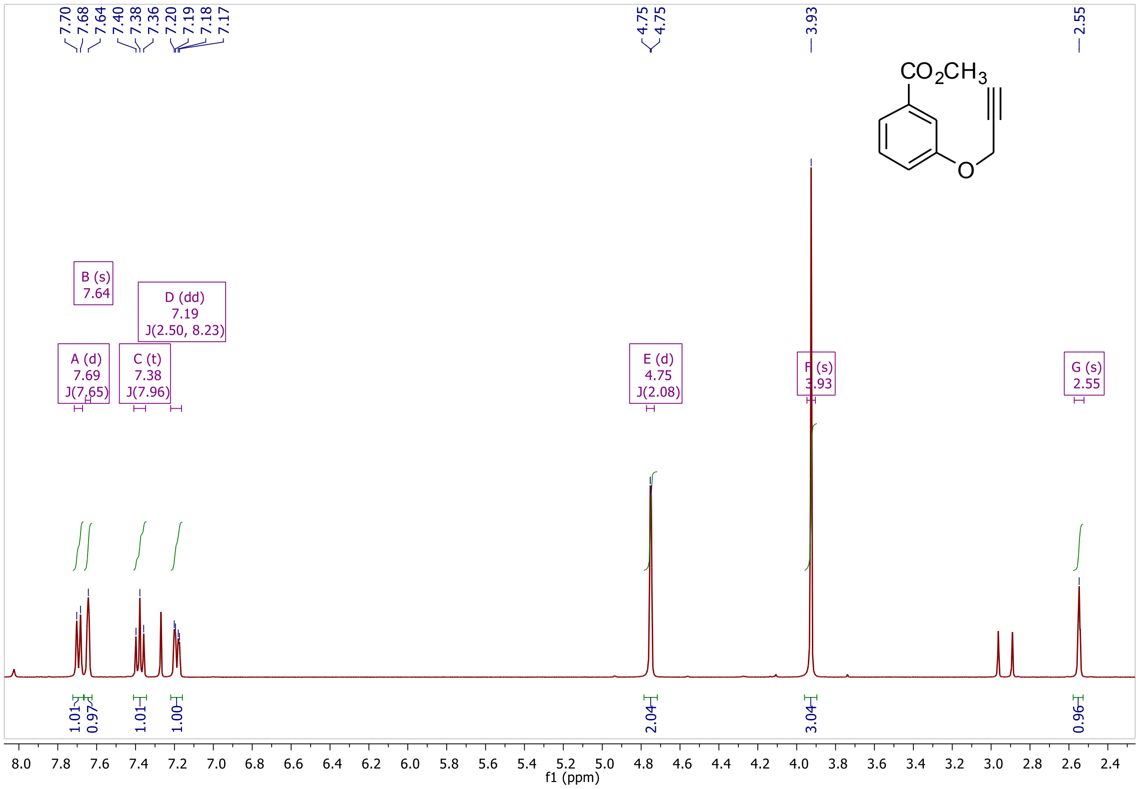

^{1}H NMR (400 MHz, $CDCl_3$) of methyl 2H-chromene-5-carboxylate **3**

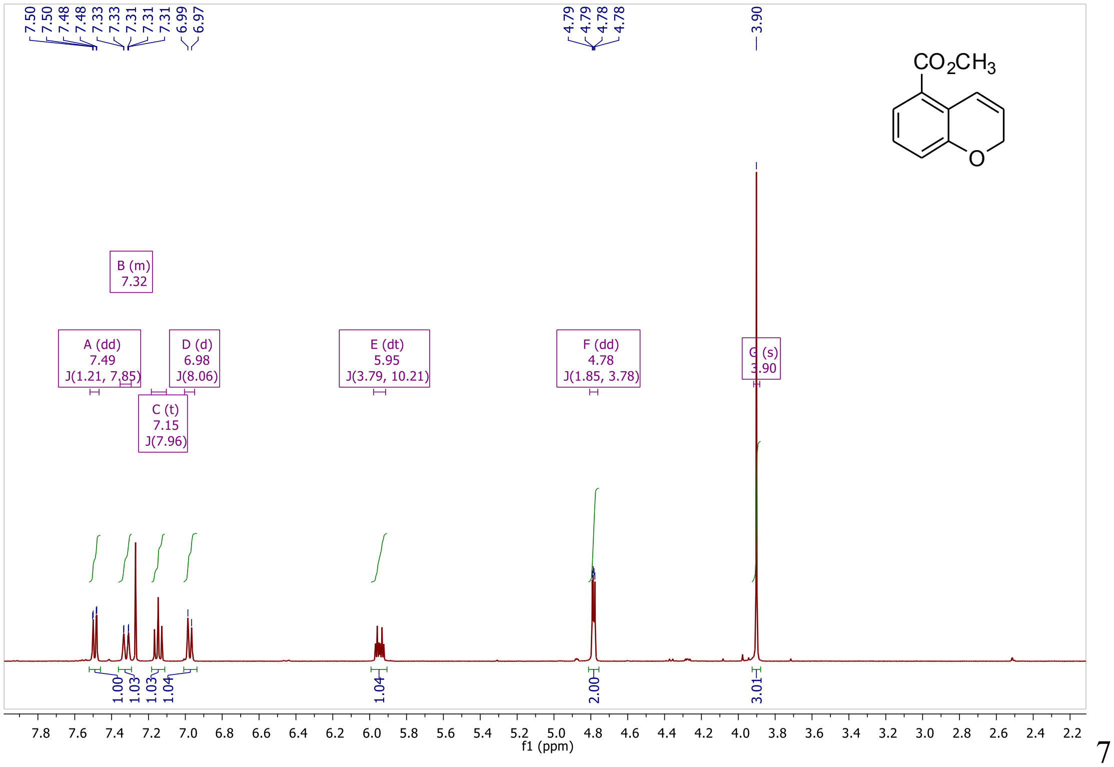

^{1}H NMR (400 MHz, $CDCl_3$) of 2H-chromene-5-carboxylic acid

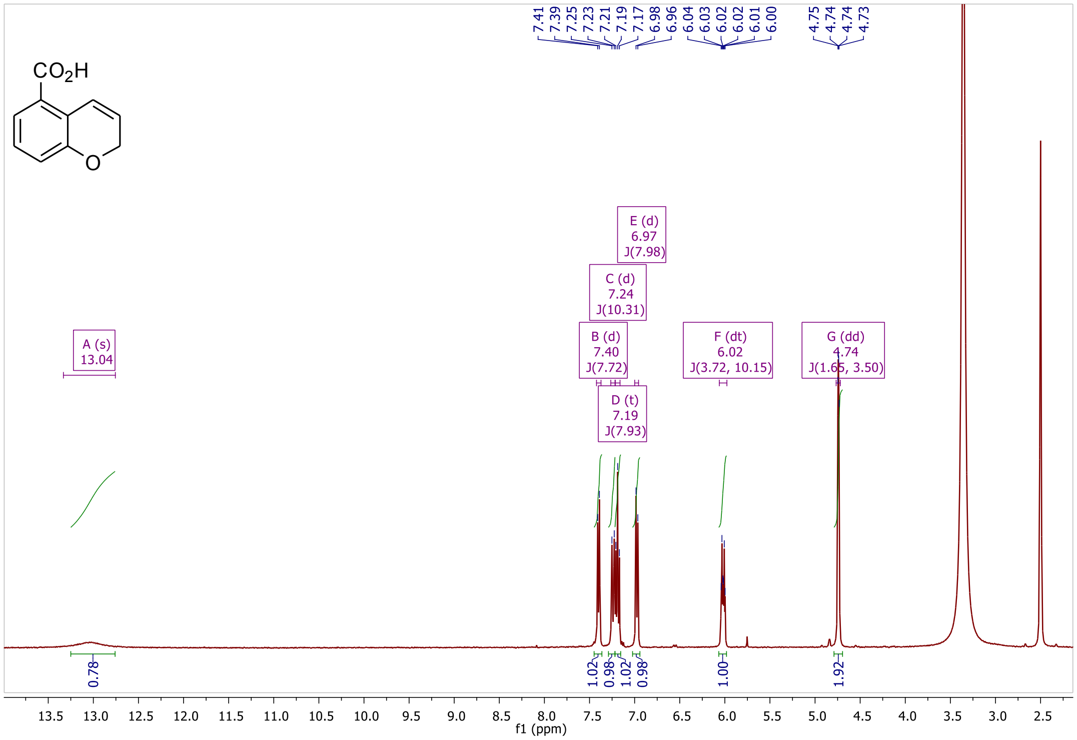

^{1}H NMR (300 MHz, $CDCl_3$) of N-phenyl-2H-chromene-5-carboxamide **1**

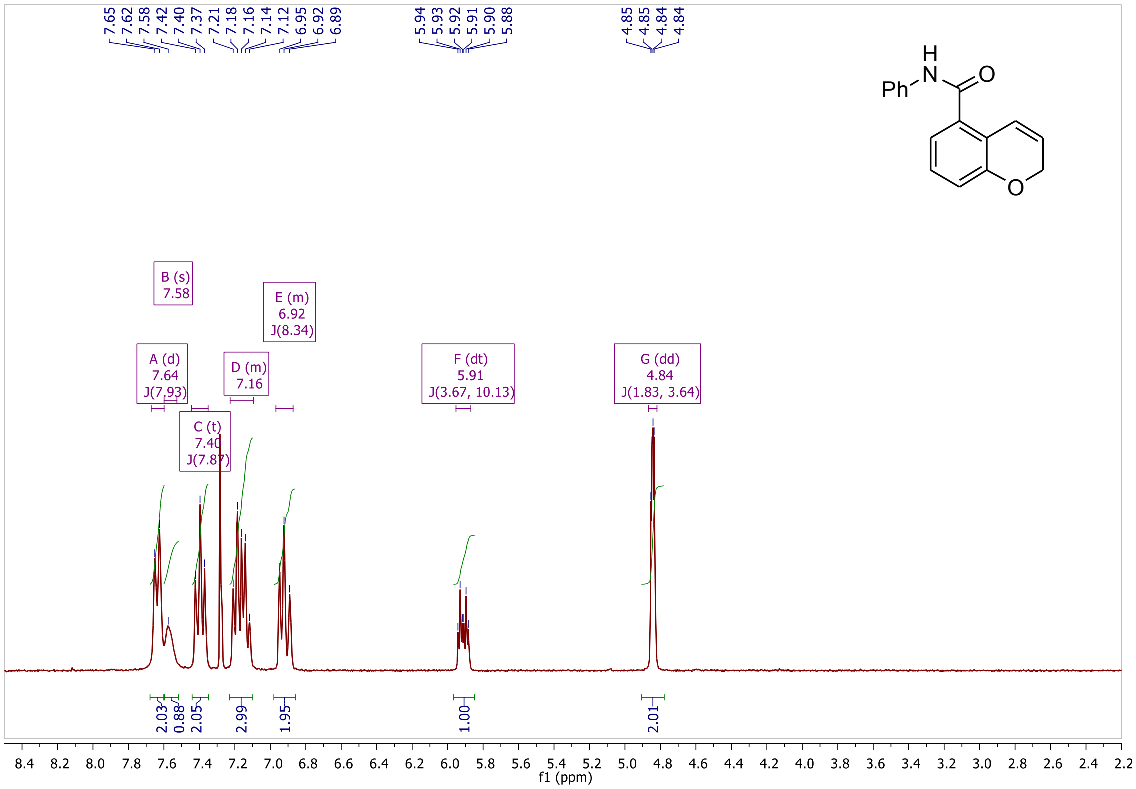

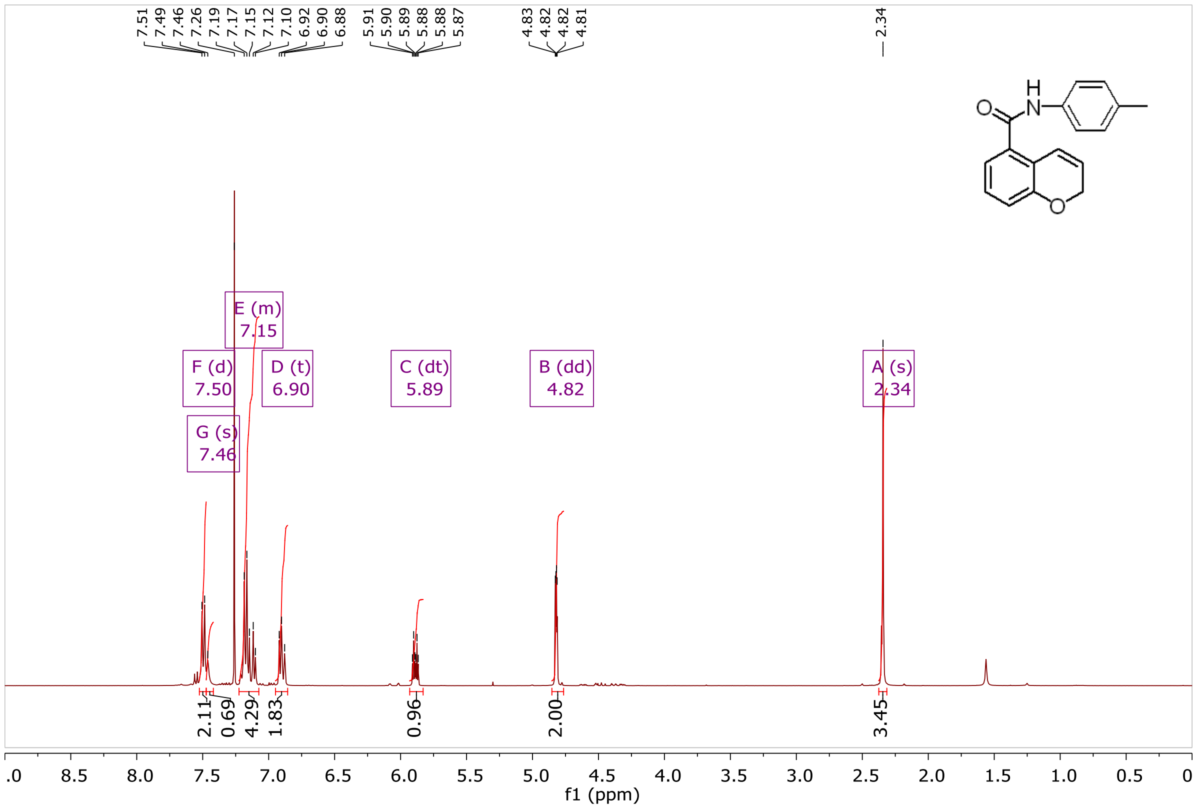

^{1}H NMR (400 MHz, $CDCl_3$) and ^{13}C NMR (101 MHz, $CDCl_3$) of **2a:**

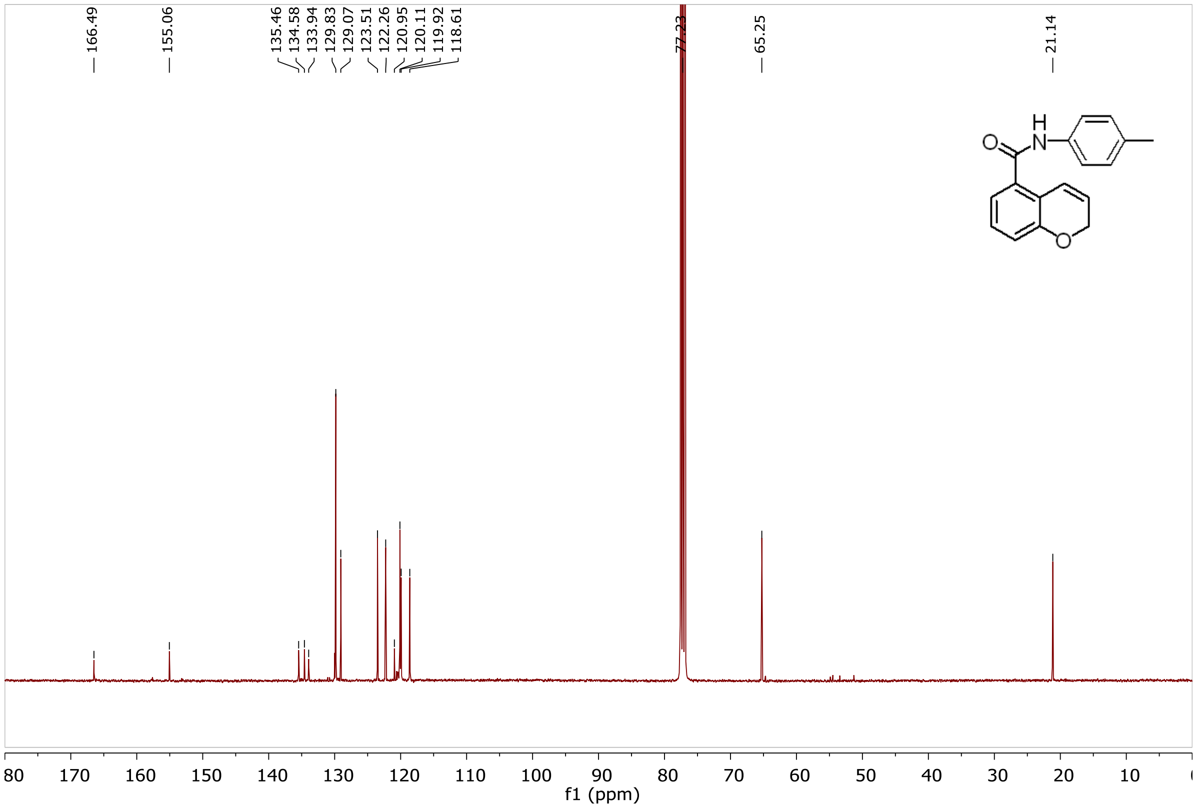

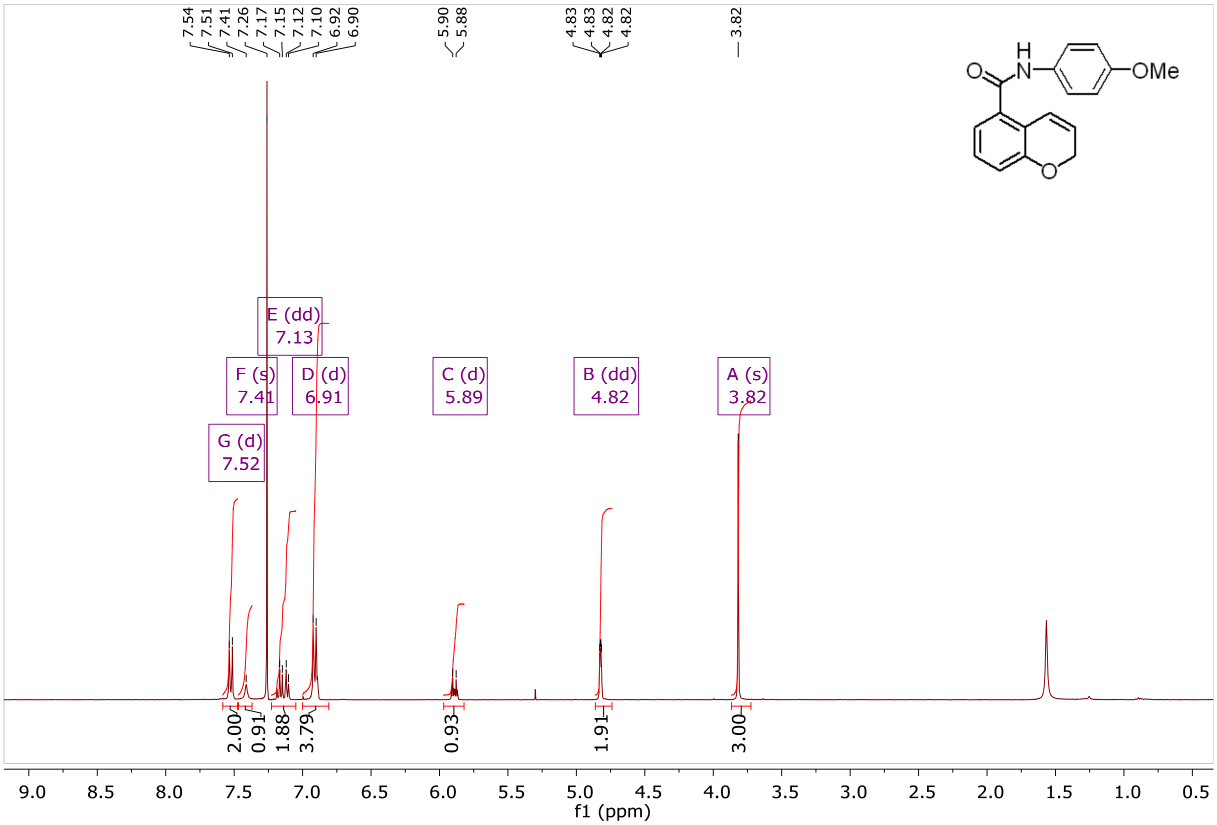
7.54
7.51
7.41
7.26
7.17
7.15
7.12
7.10
6.92
6.90
5.90
5.88
4.83
4.83
4.82
4.82
3.82
H
N
O
OMe
O
E (dd)
7.13
F (s)
7.41
D (d)
6.91
C (d)
5.89
B (dd)
4.82
A (s)
3.82
G (d)
7.52
2.00
0.91
1.88
3.79
0.93
1.91
3.00
9.0
8.5
8.0
7.5
7.0
6.5
6.0
5.5
5.0
4.5
4.0
3.5
3.0
2.5
2.0
1.5
1.0
0.5
f1 (ppm)

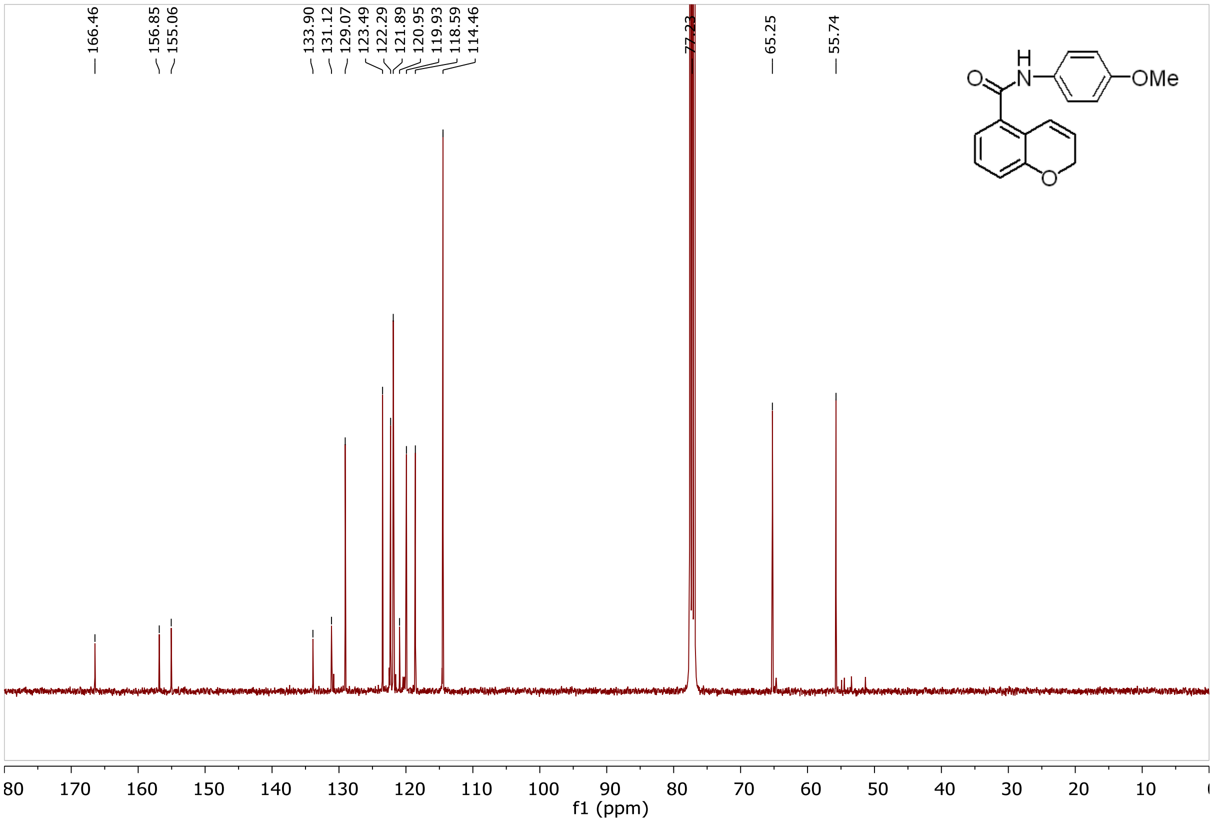
166.46
156.85
155.06
133.90
131.12
129.07
123.49
122.29
121.89
120.95
119.93
118.59
114.46
65.25
55.74
H
N
O
OMe
f1 (ppm)

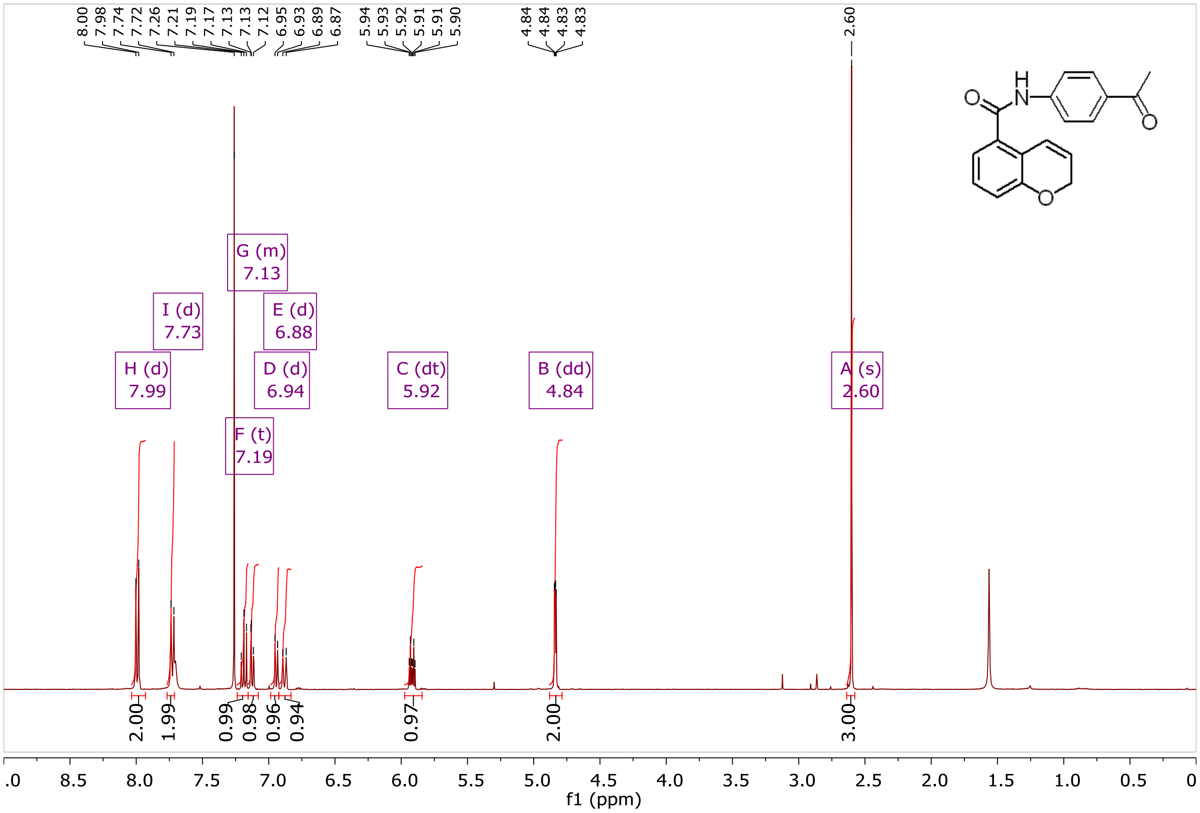
8.00
7.98
7.74
7.72
7.26
7.21
7.19
7.17
7.13
7.13
7.12
6.95
6.93
6.89
6.87
5.94
5.93
5.92
5.91
5.91
5.90
4.84
4.84
4.83
4.83
2.60
H
N
O
G (m) 7.13
I (d) 7.73
E (d) 6.88
H (d) 7.99
D (d) 6.94
C (dt) 5.92
B (dd) 4.84
A (s) 2.60
F (t) 7.19
2.00
1.99
0.99
0.98
0.96
0.94
0.97
2.00
3.00
f1 (ppm)

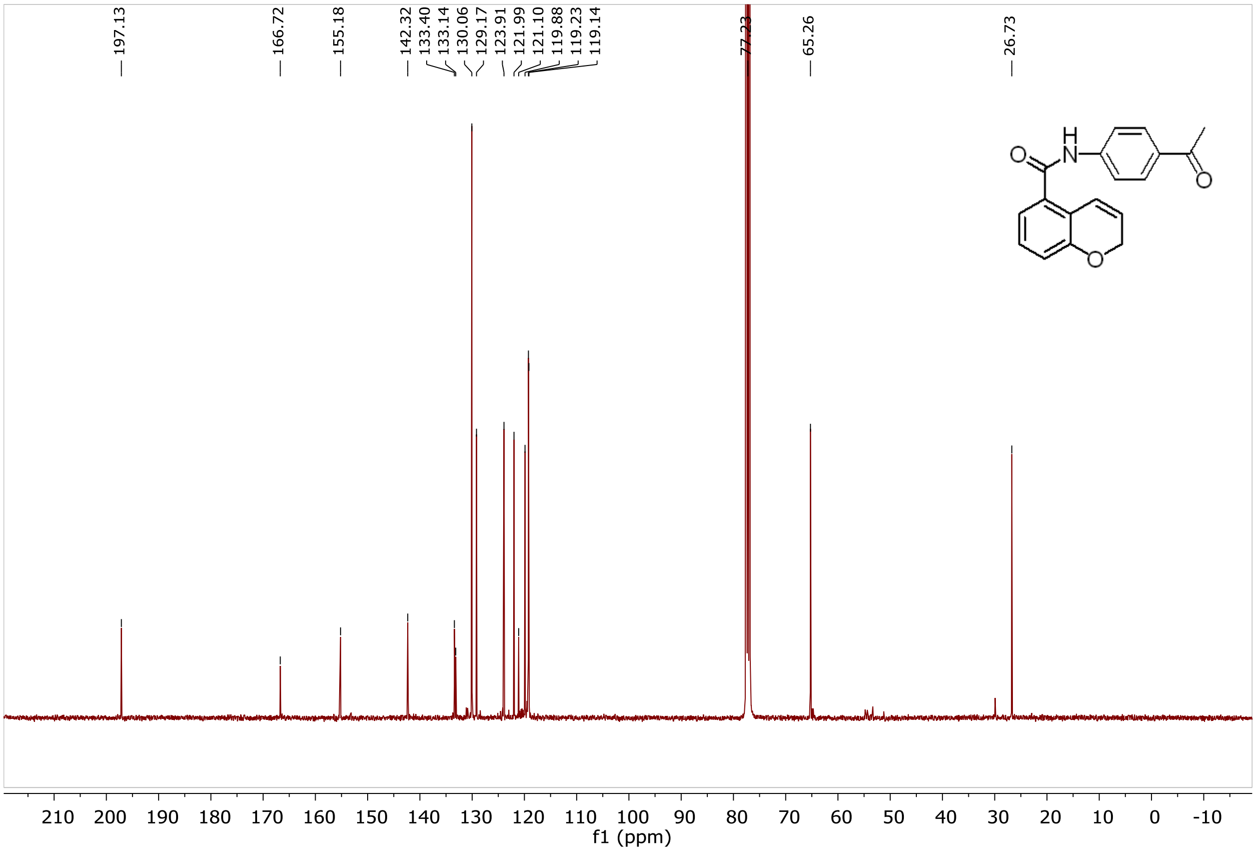
197.13
166.72
155.18
142.32
133.40
133.14
130.06
129.17
123.91
121.99
121.10
119.88
119.23
119.14
77.23
65.26
26.73
210 200 190 180 170 160 150 140 130 120 110 100 90 80 70 60 50 40 30 20 10 0 -10
f1 (ppm)

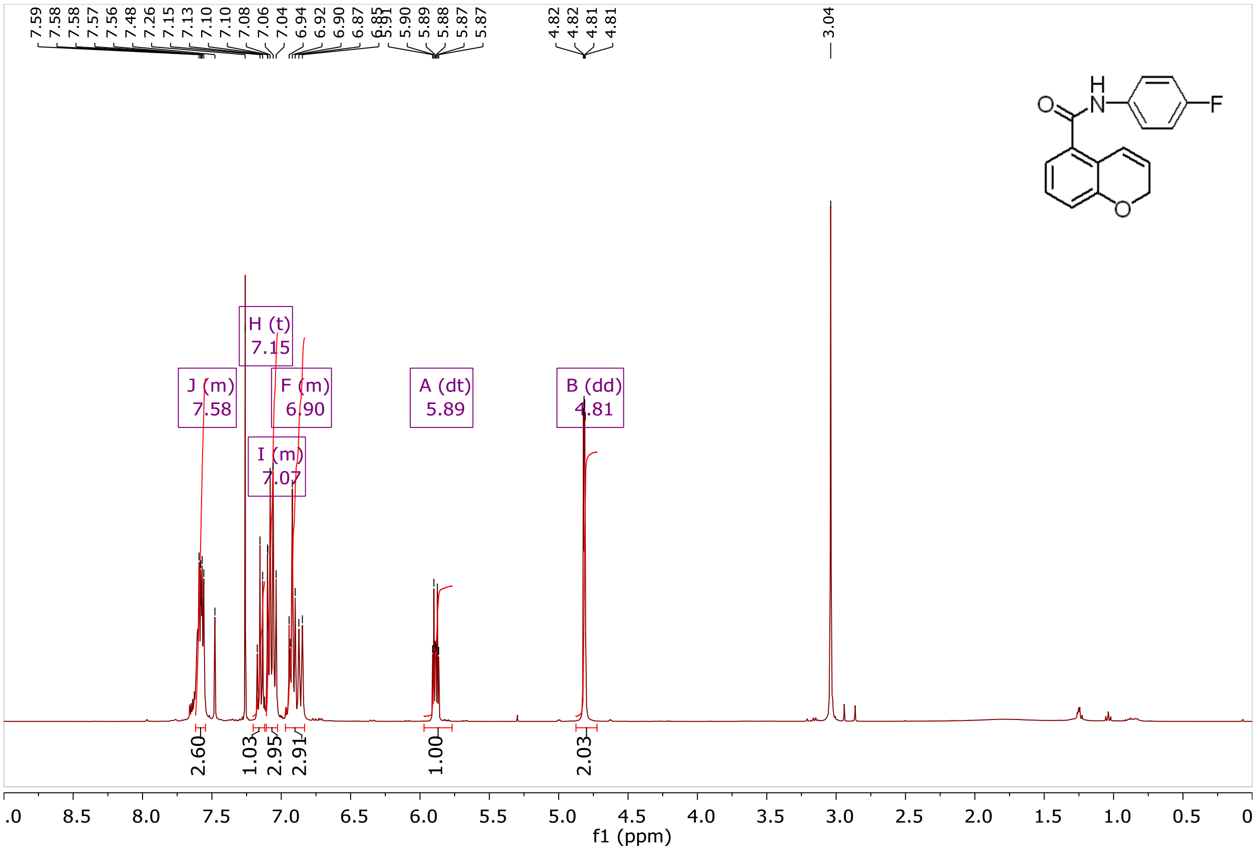
7.59
7.58
7.58
7.57
7.56
7.48
7.26
7.15
7.13
7.10
7.10
7.08
7.06
7.04
6.94
6.92
6.90
6.87
6.85
5.91
5.90
5.89
5.88
5.87
5.87
4.82
4.82
4.81
4.81
3.04
H (t) 7.15
J (m) 7.58
F (m) 6.90
A (dt) 5.89
B (dd) 4.81
I (m) 7.07
2.60
1.03
2.95
2.91
1.00
2.03
.0 8.5 8.0 7.5 7.0 6.5 6.0 5.5 5.0 4.5 4.0 3.5 3.0 2.5 2.0 1.5 1.0 0.5 0
f1 (ppm)

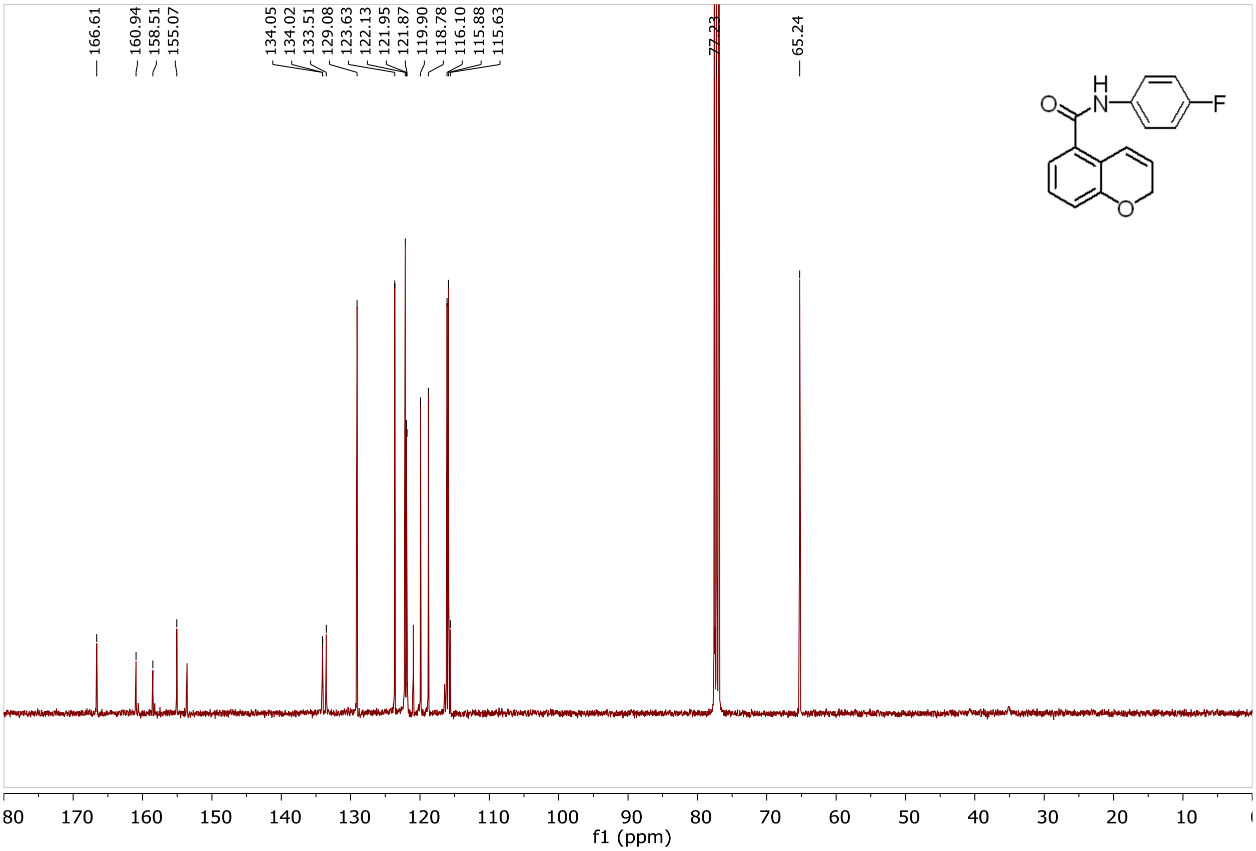
166.61
160.94
158.51
155.07
134.05
134.02
133.51
129.08
123.63
122.13
121.95
121.87
119.90
118.78
116.10
115.88
115.63
77.23
65.24
O
H
N
F
O
80 170 160 150 140 130 120 110 100 90 80 70 60 50 40 30 20 10
f1 (ppm)

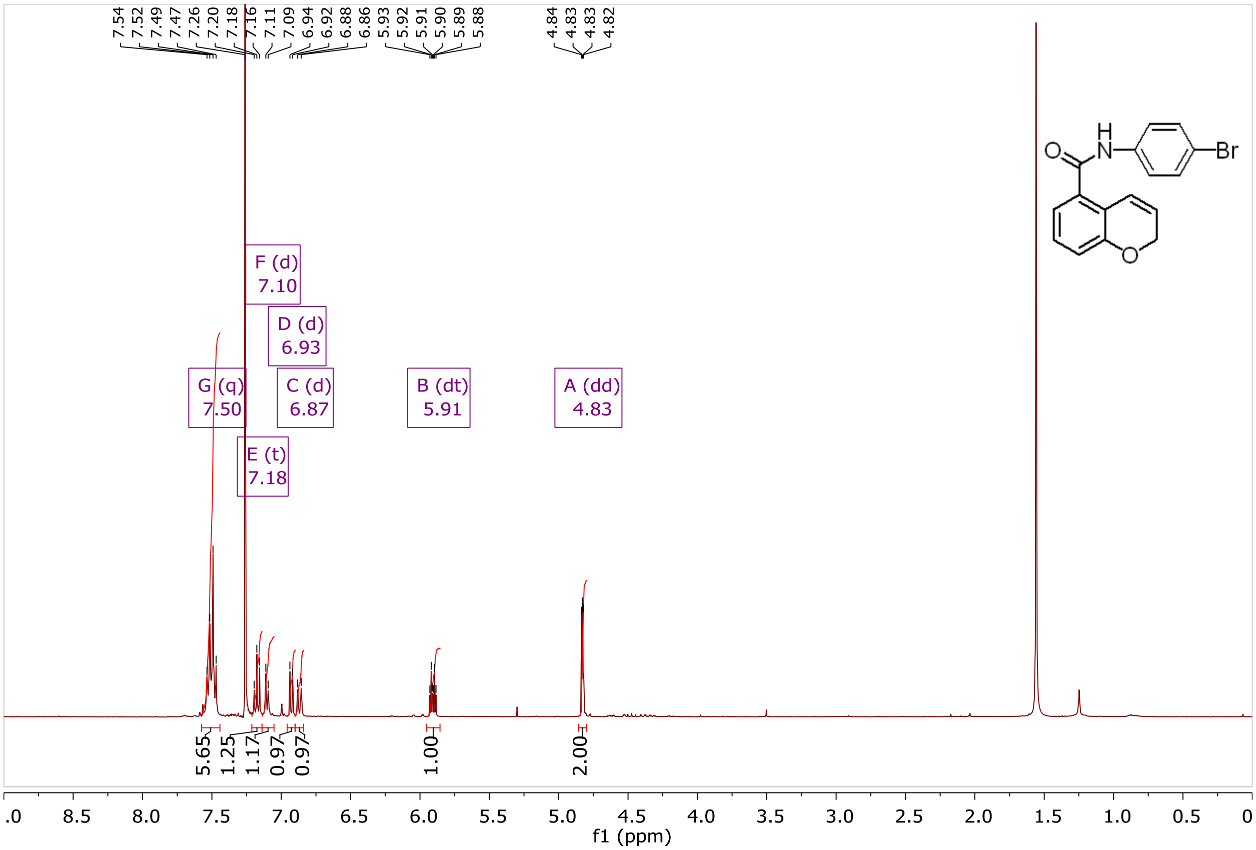
7.54
7.52
7.49
7.47
7.26
7.20
7.18
7.16
7.11
7.09
6.94
6.92
6.88
6.86
5.93
5.92
5.91
5.90
5.89
5.88
4.84
4.83
4.83
4.82
O
H
N
Br
O
F (d)
7.10
D (d)
6.93
G (q)
7.50
C (d)
6.87
B (dt)
5.91
A (dd)
4.83
E (t)
7.18
5.65
1.25
1.17
0.97
0.97
1.00
2.00
.0 8.5 8.0 7.5 7.0 6.5 6.0 5.5 5.0 4.5 4.0 3.5 3.0 2.5 2.0 1.5 1.0 0.5 0
f1 (ppm)

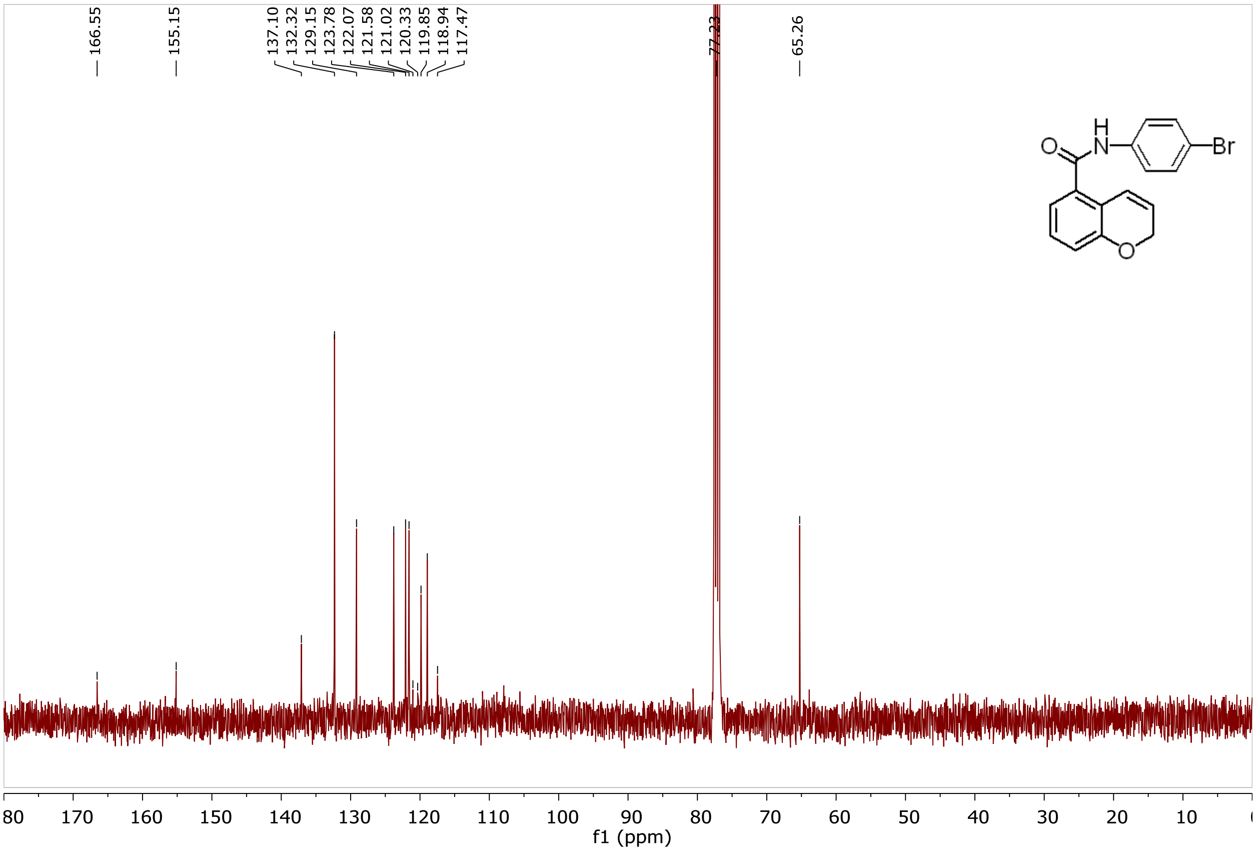
166.55
155.15
137.10
132.32
129.15
123.78
122.07
121.58
121.02
120.33
119.85
118.94
117.47
77.23
65.26
f1 (ppm)
Br

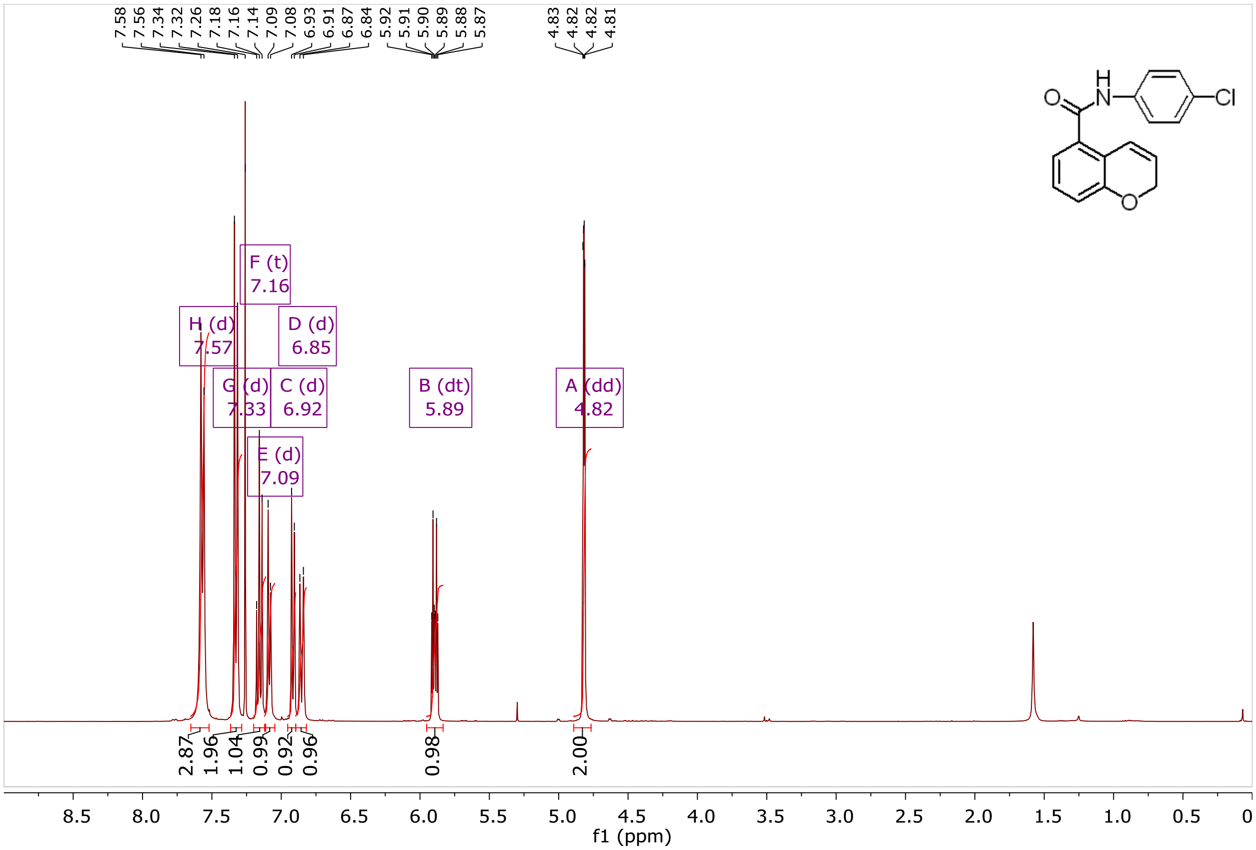
7.58
7.56
7.34
7.32
7.26
7.18
7.16
7.14
7.09
7.08
6.93
6.91
6.87
6.84
5.92
5.91
5.90
5.89
5.88
5.87
4.83
4.82
4.82
4.81
F (t) 7.16
H (d) 7.57
D (d) 6.85
G (d) 7.33
C (d) 6.92
B (dt) 5.89
A (dd) 4.82
E (d) 7.09
2.87
1.96
1.04
0.99
0.92
0.96
0.98
2.00
f1 (ppm)
Cl

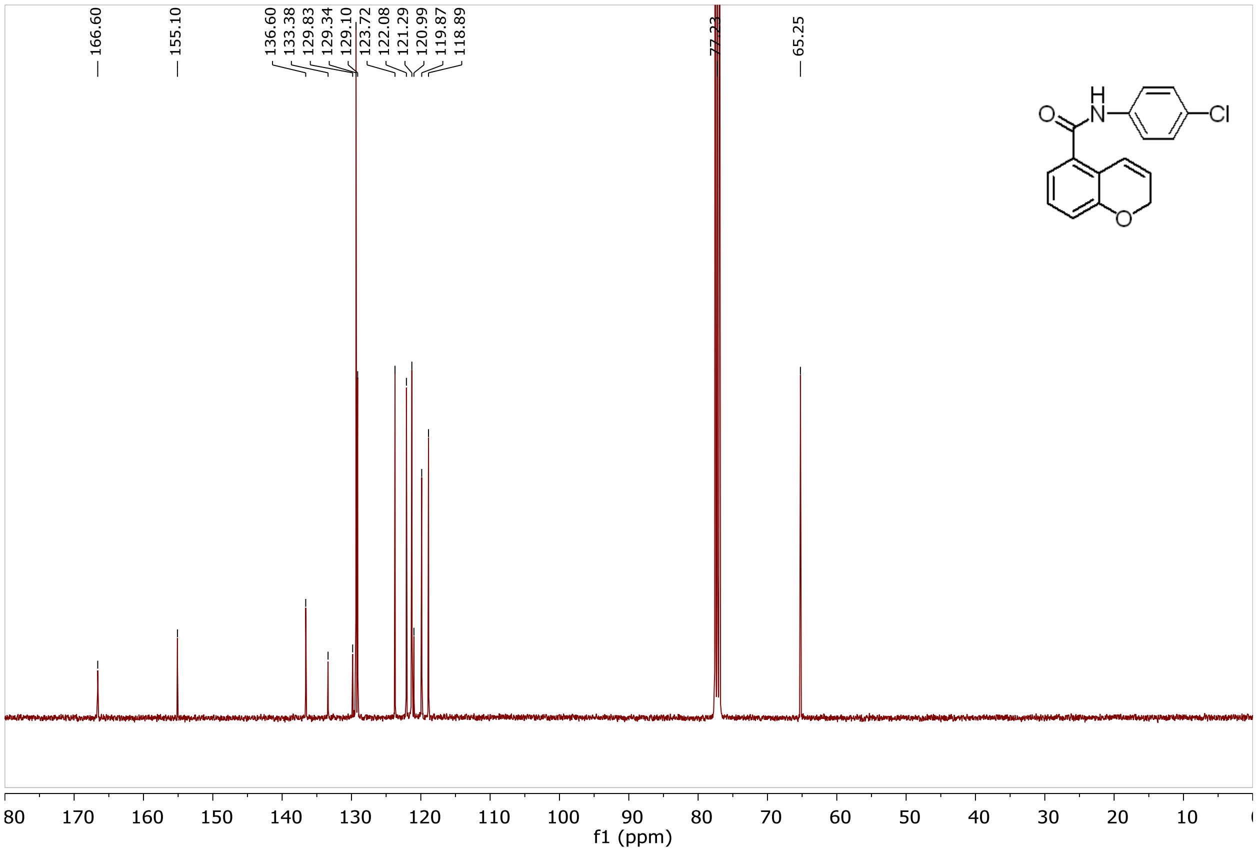
166.60
155.10
136.60
133.38
129.83
129.34
129.10
123.72
122.08
121.29
120.99
119.87
118.89
77.23
65.25
O
H
N
Cl
f1 (ppm)

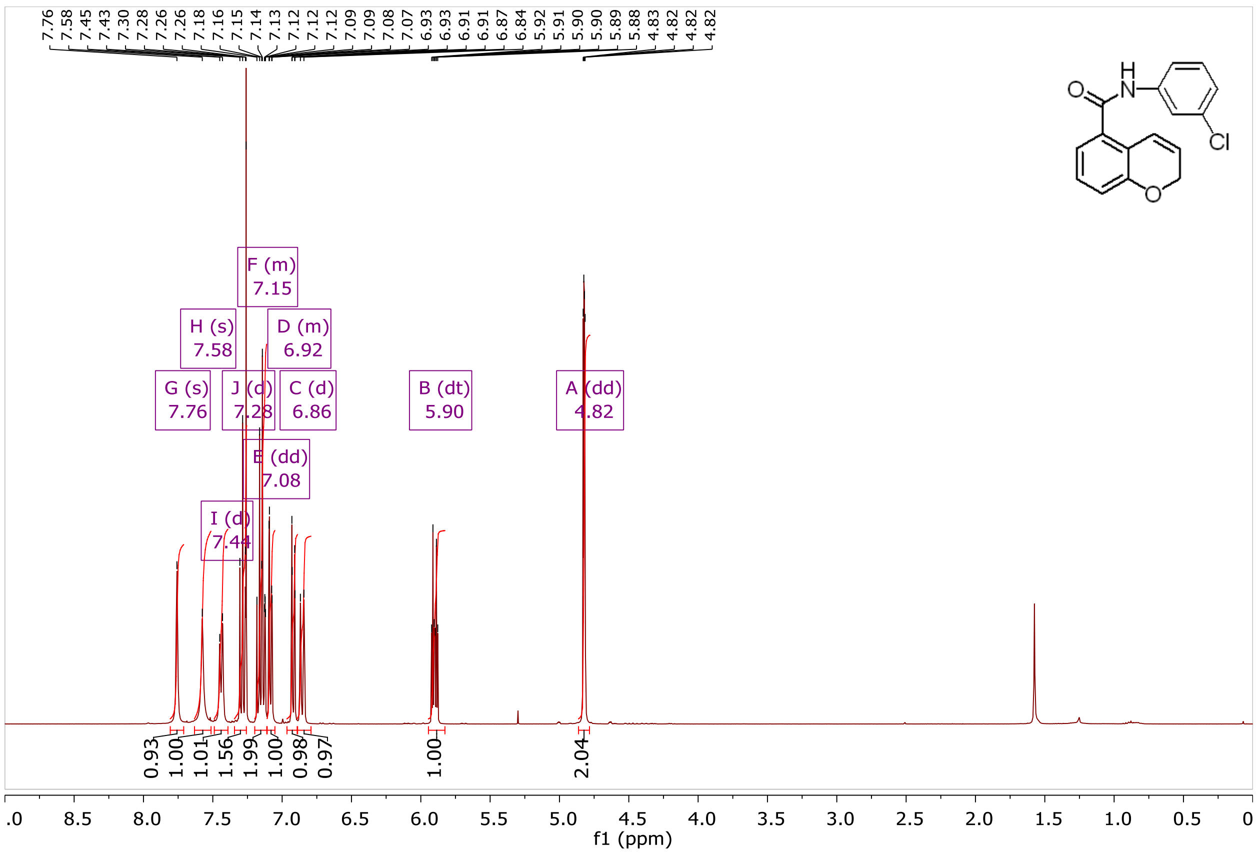
F (m)
7.15
H (s)
7.58
D (m)
6.92
G (s)
7.76
J (d)
7.28
C (d)
6.86
B (dt)
5.90
A (dd)
4.82
E (dd)
7.08
I (d)
7.44
0.93
1.00
1.01
1.56
1.99
1.00
0.98
0.97
1.00
2.04
O
H
N
Cl
f1 (ppm)

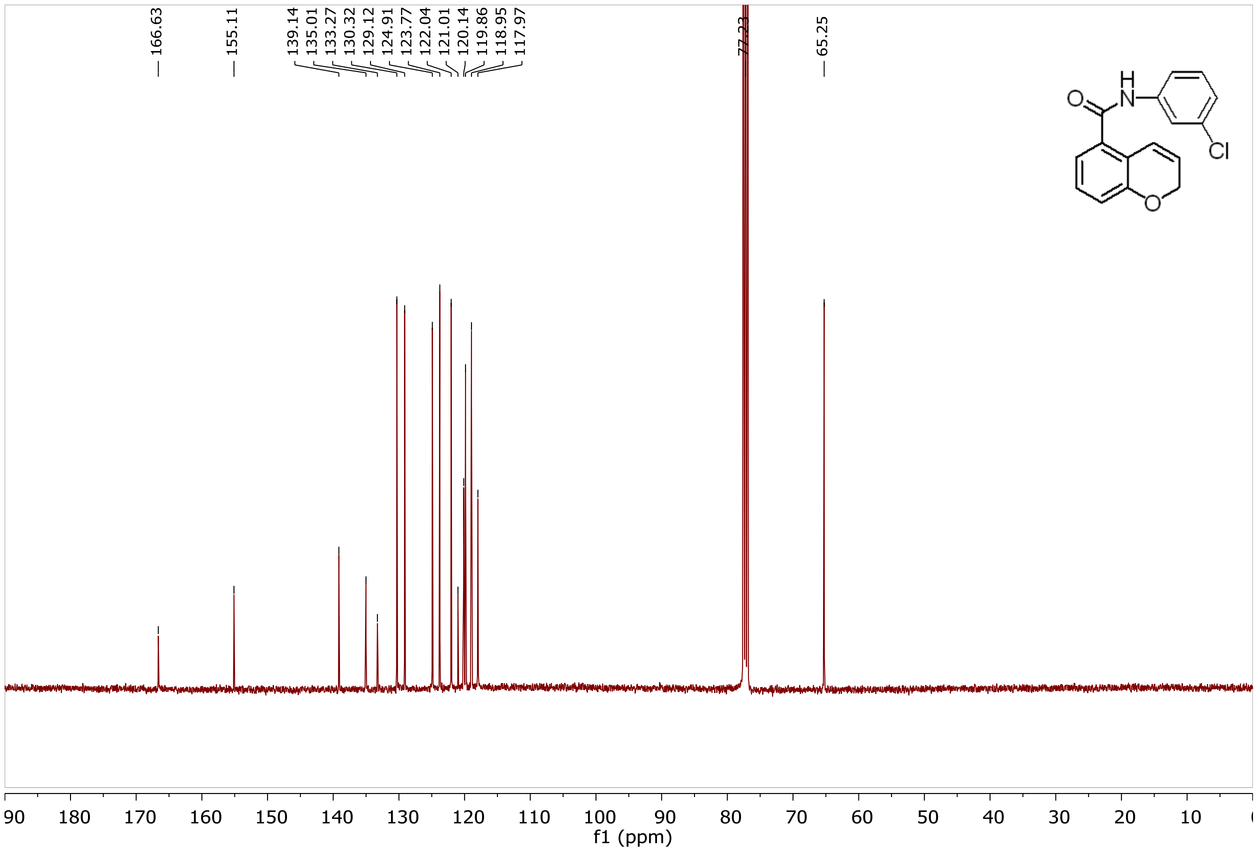
166.63
155.11
139.14
135.01
133.27
130.32
129.12
124.91
123.77
122.04
121.01
120.14
119.86
118.95
117.97
77.23
65.25
O
H
N
Cl
O
90
180
170
160
150
140
130
120
110
100
90
80
70
60
50
40
30
20
10
f1 (ppm)

^{1}H NMR (400 MHz, $CDCl_3$) and ^{13}C NMR (101 MHz, $CDCl_3$) of **2a:**

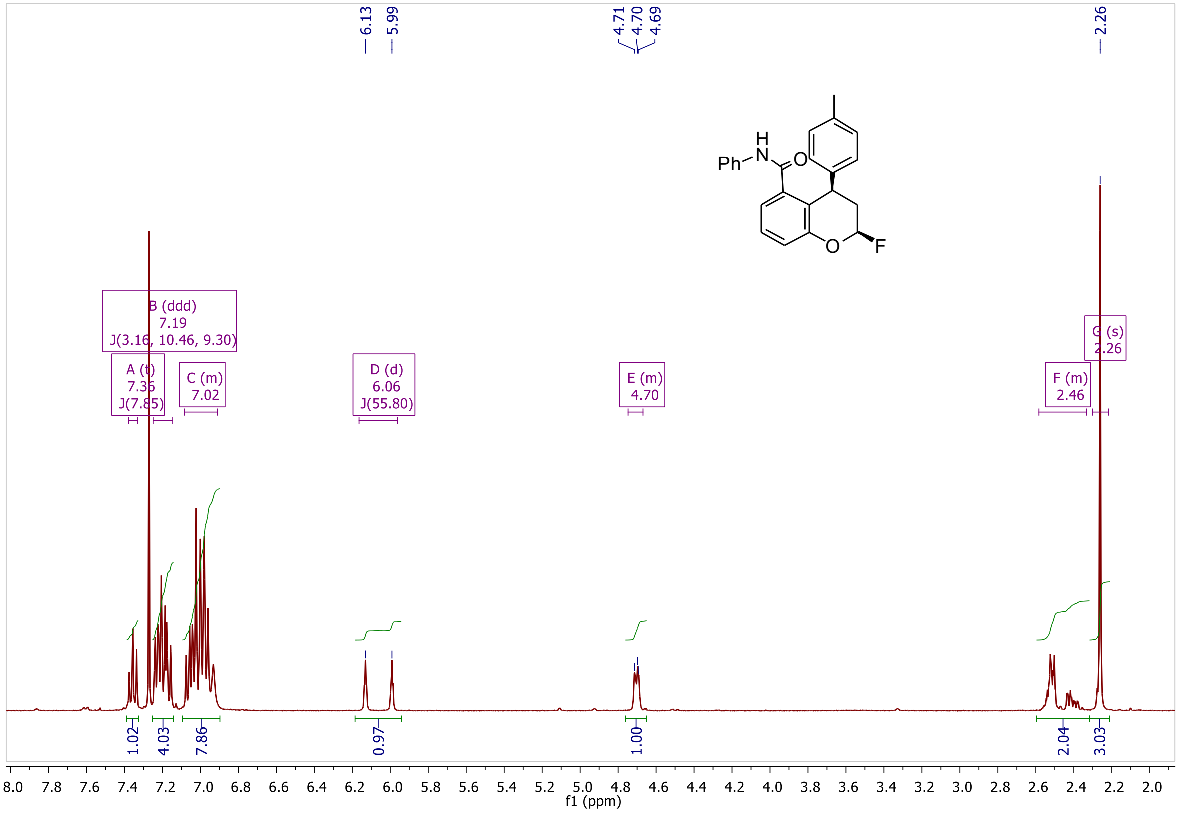

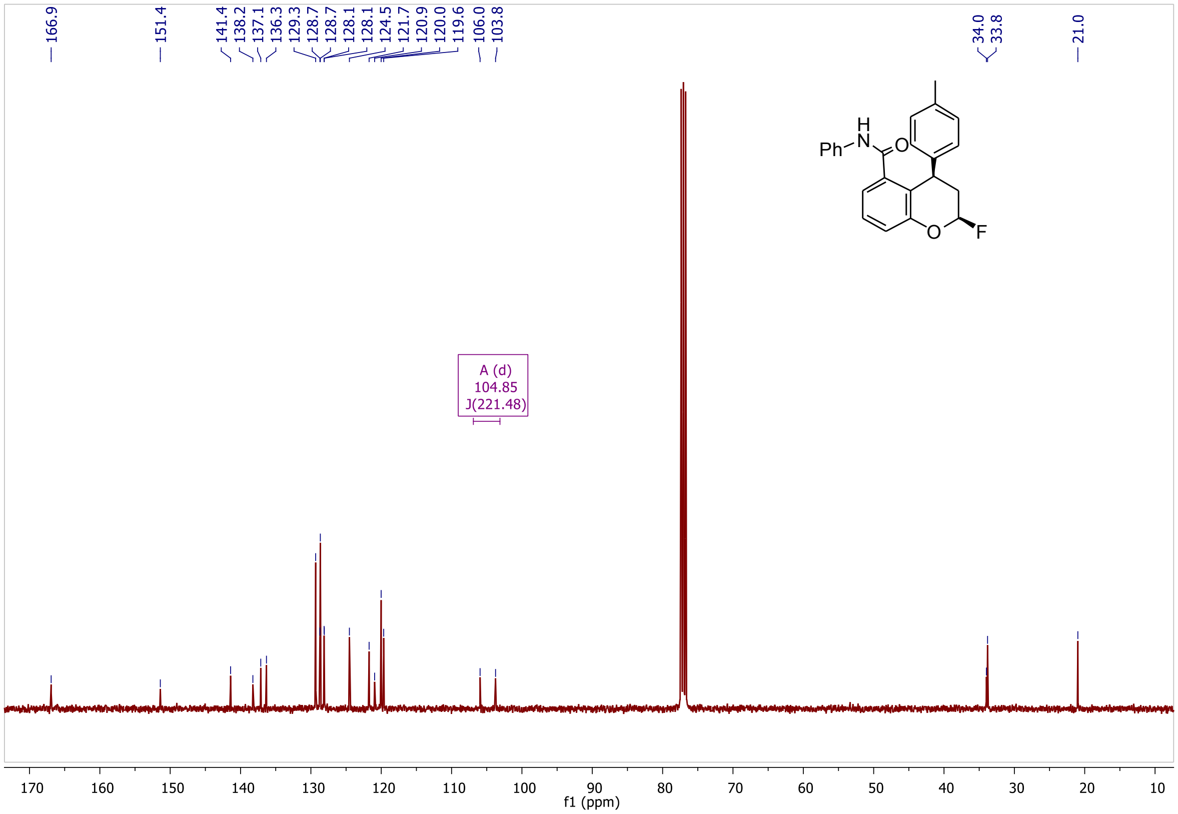

^{1}H NMR (400 MHz, $CDCl_3$) and ^{13}C NMR (101 MHz, $CDCl_3$) of **2b:**

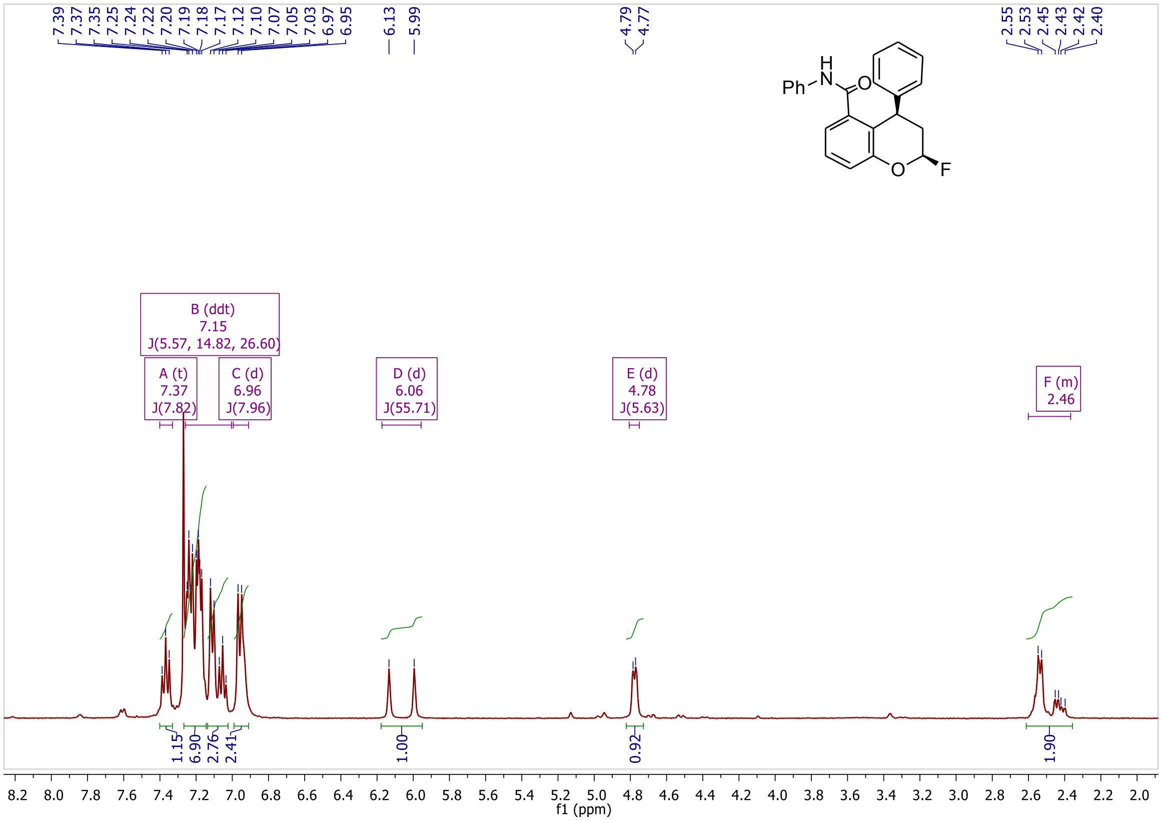

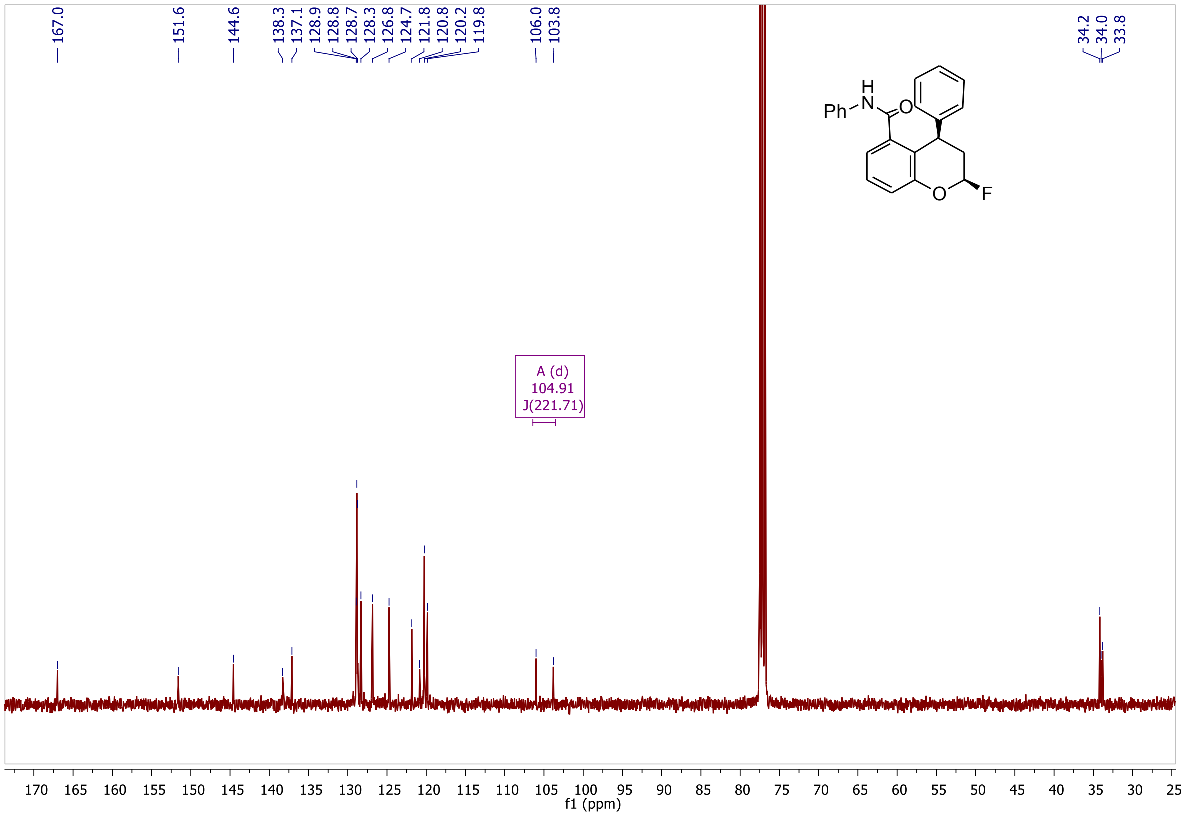

^{1}H NMR (600 MHz, $CDCl_3$) and ^{13}C NMR (151 MHz, $CDCl_3$) of **2c:**

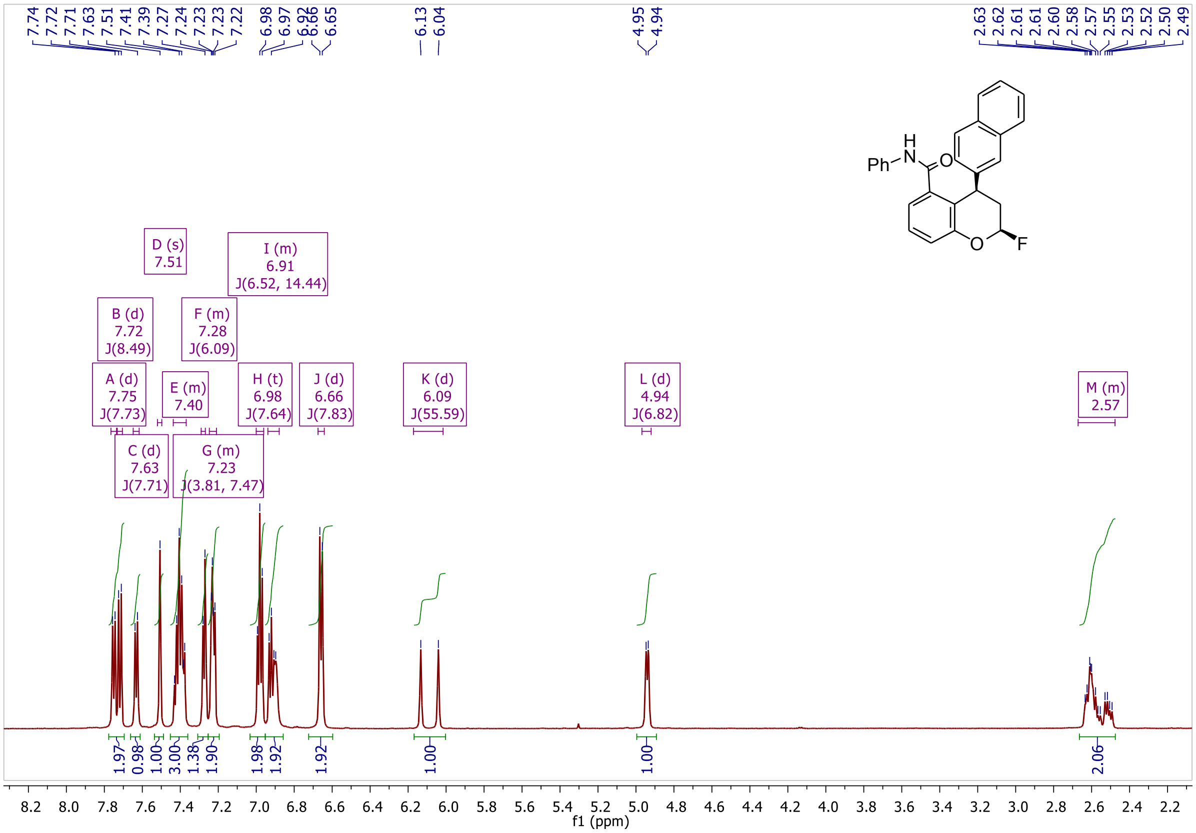

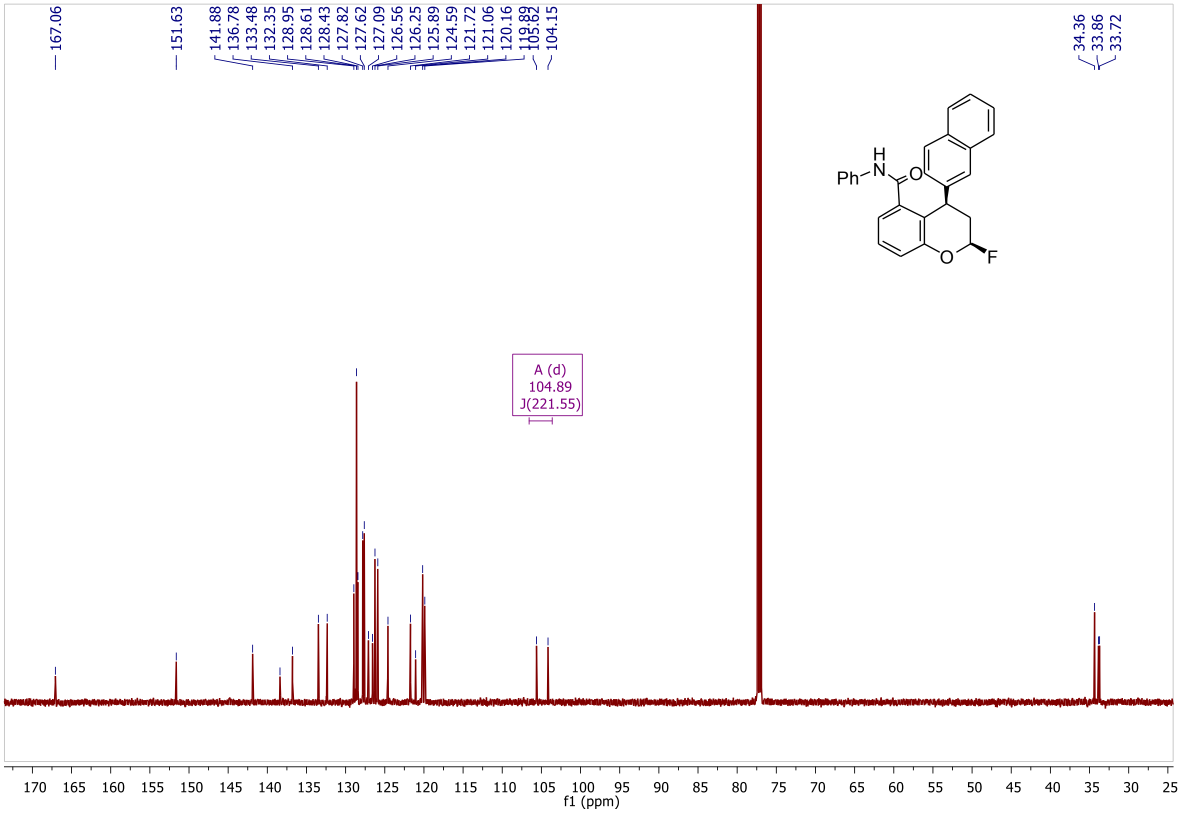

^{1}H NMR (400 MHz, CD_2Cl_2) and ^{13}C NMR (101 MHz, CD_2Cl_2) of **2d:**

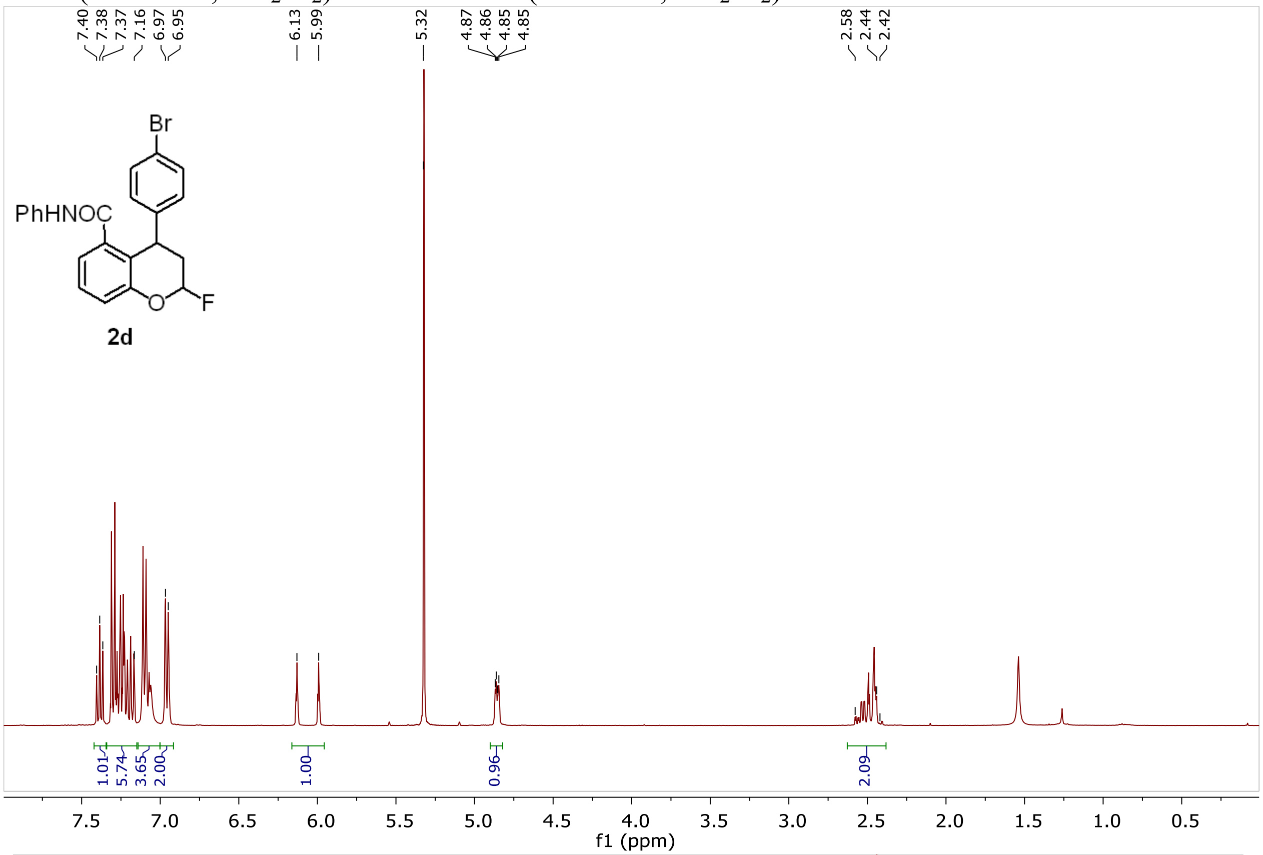

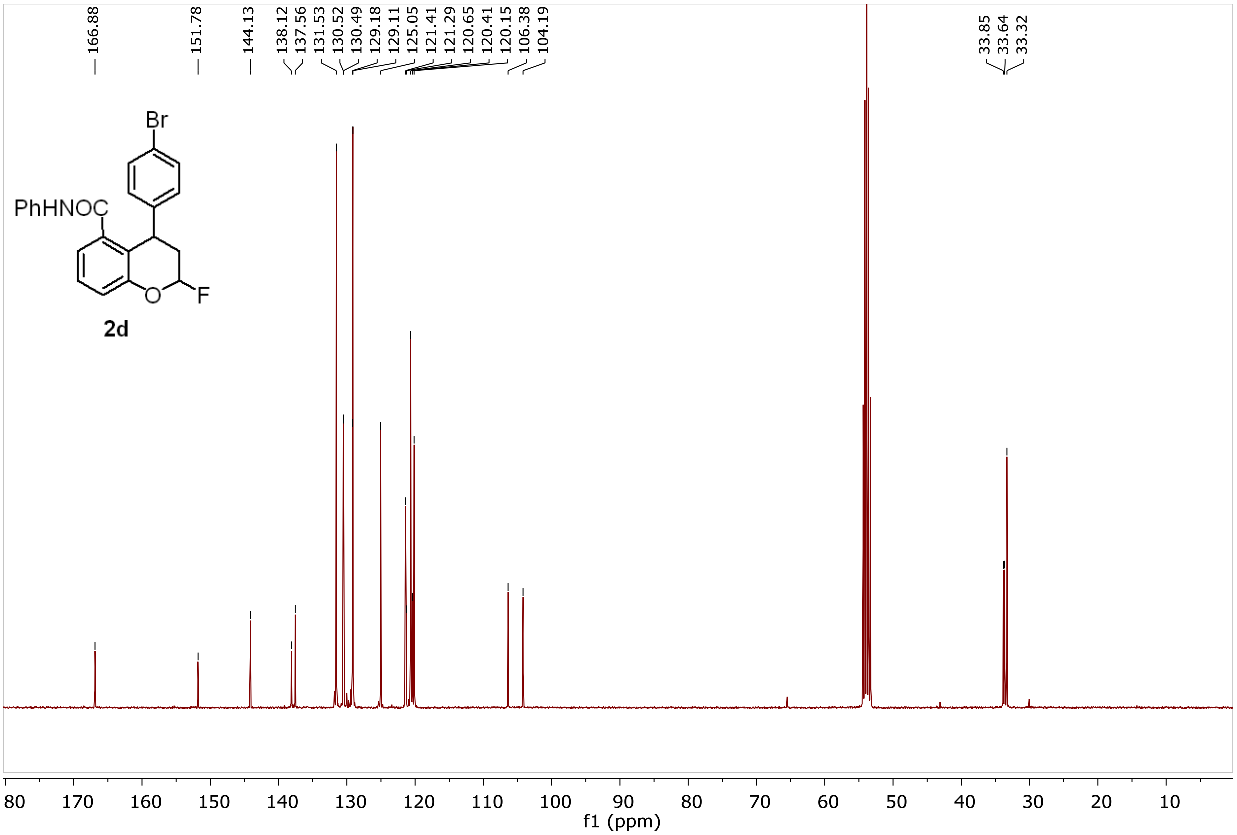

^{1}H NMR (400 MHz, CD_2Cl_2) and ^{13}C NMR (101 MHz, $CDCl_3$) of **2e**:

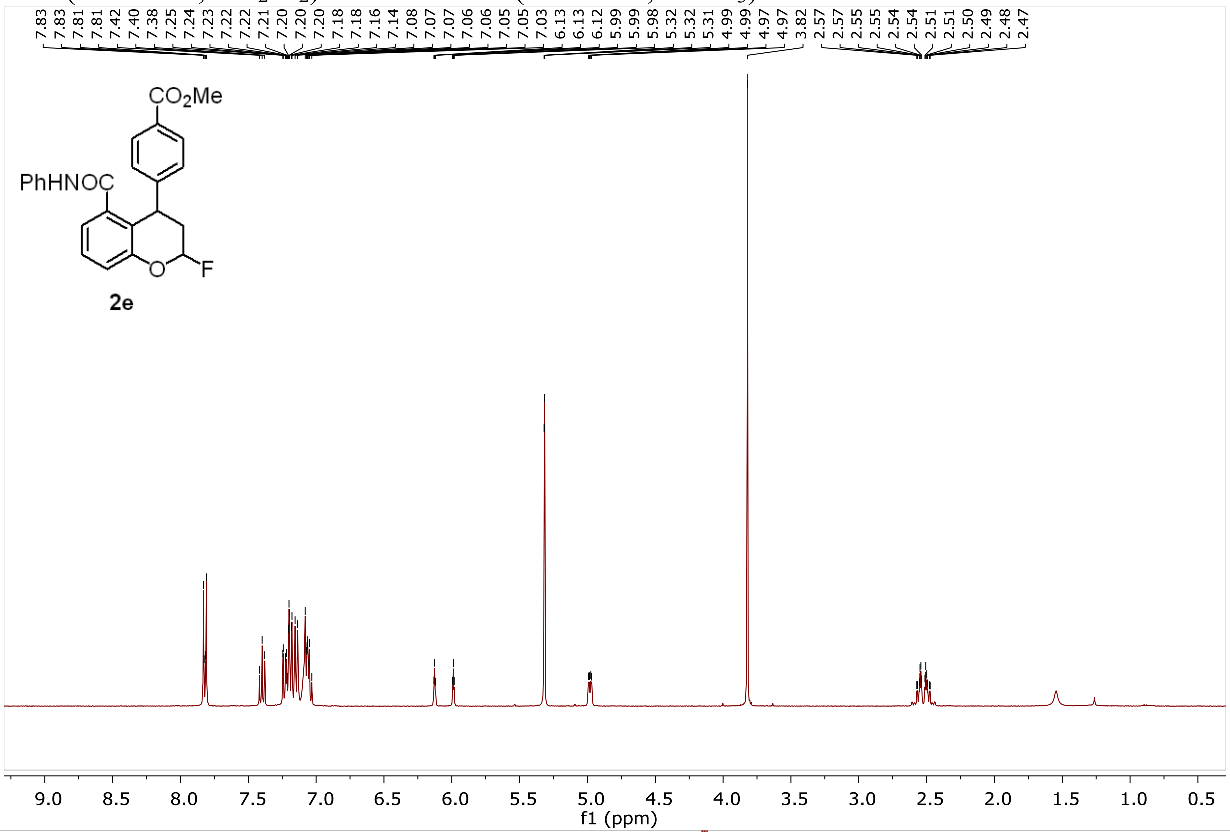

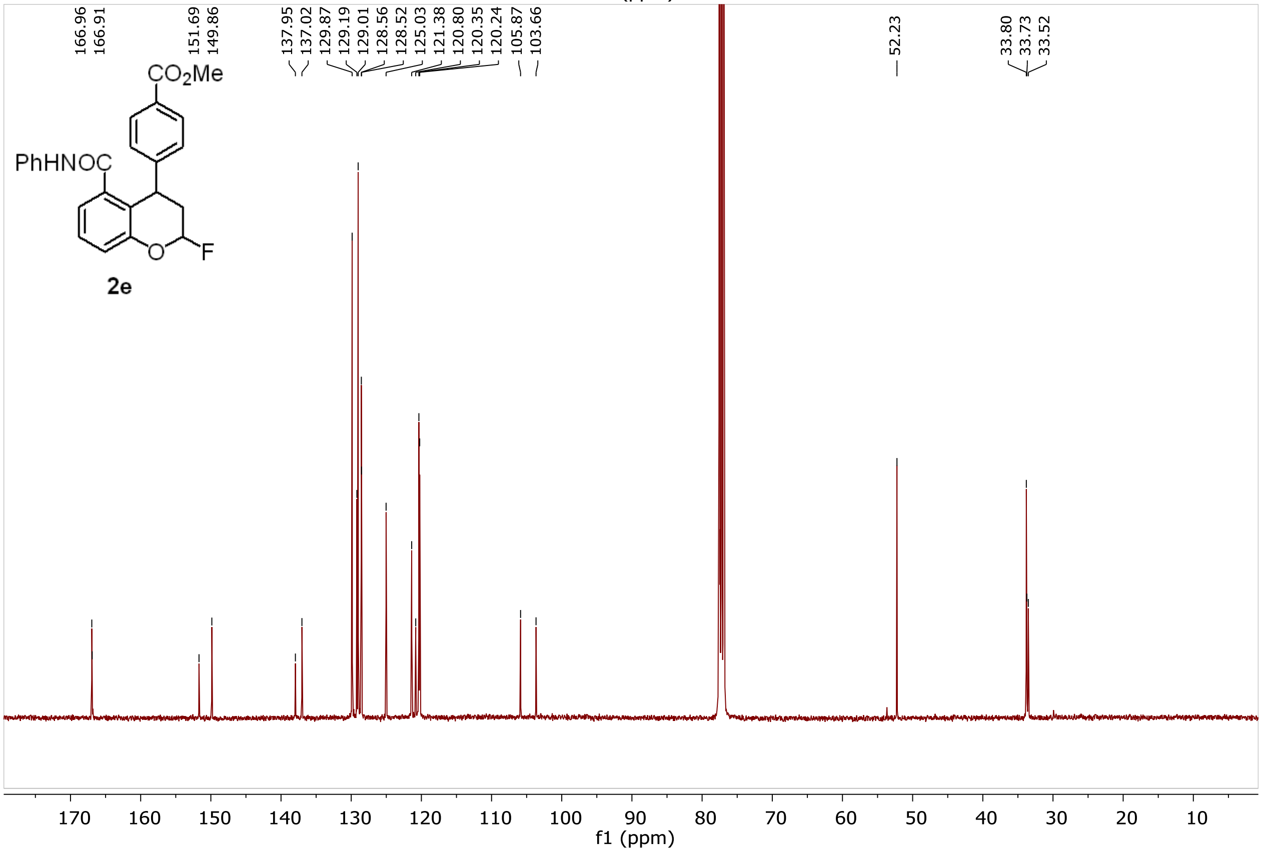

^{1}H NMR (500 MHz, $CDCl_3$) and ^{13}C NMR (101 MHz, CD_2Cl_2) of **2f** :

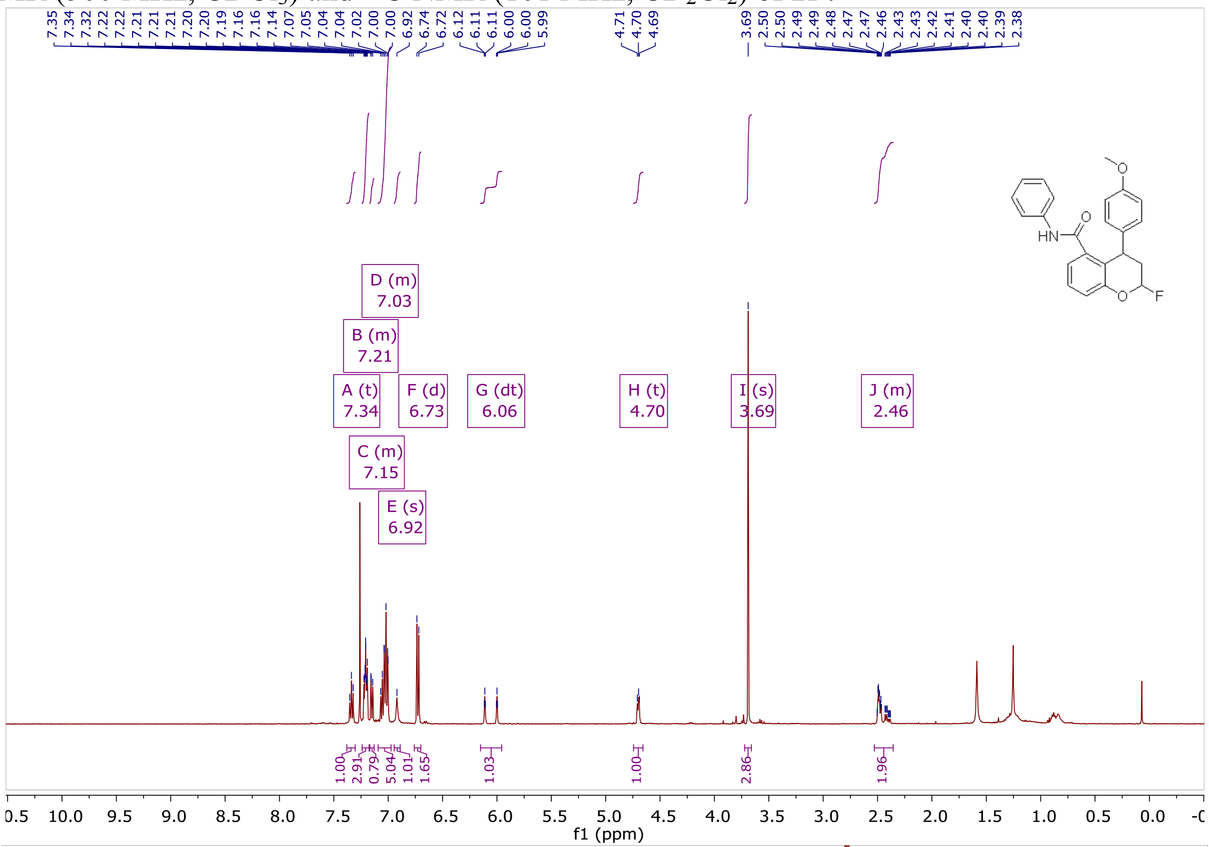

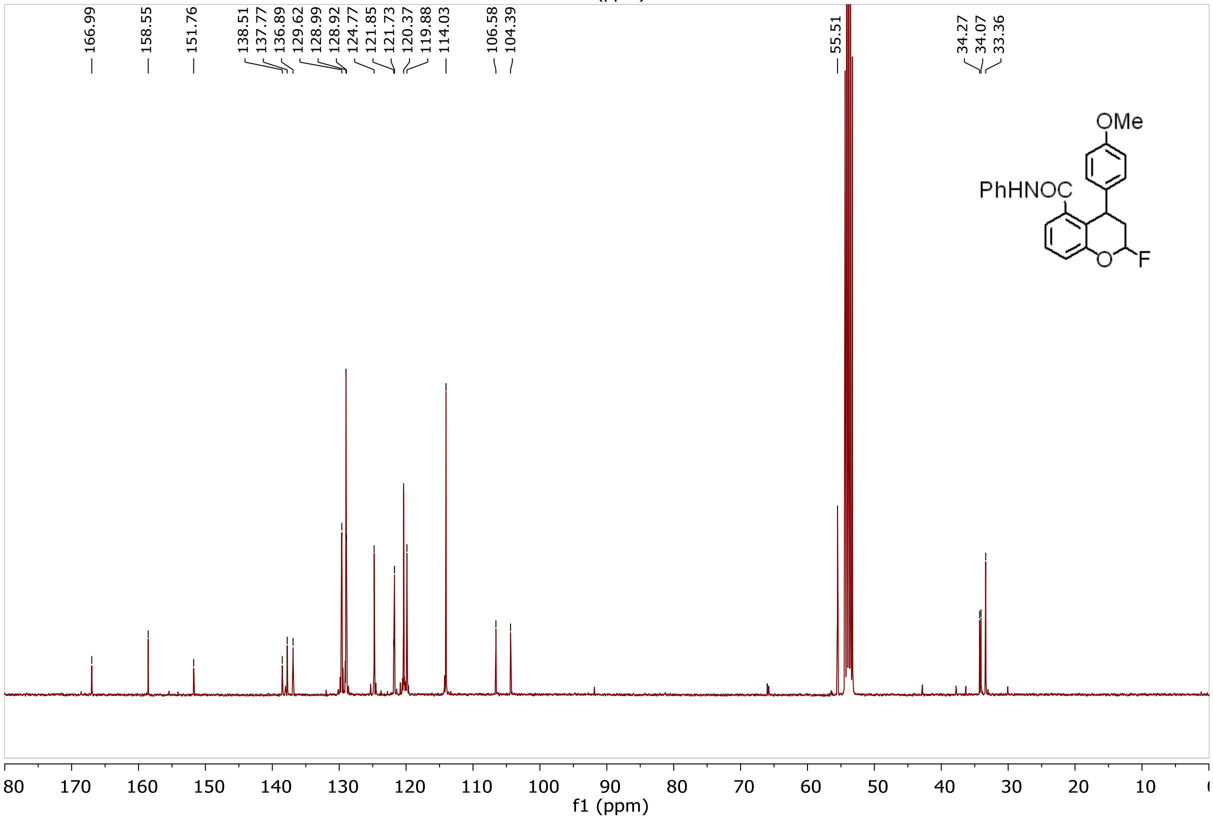

^{1}H NMR (600 MHz, CDCl3) and ^{13}C NMR (151 MHz, CDCl3) of **2g :**

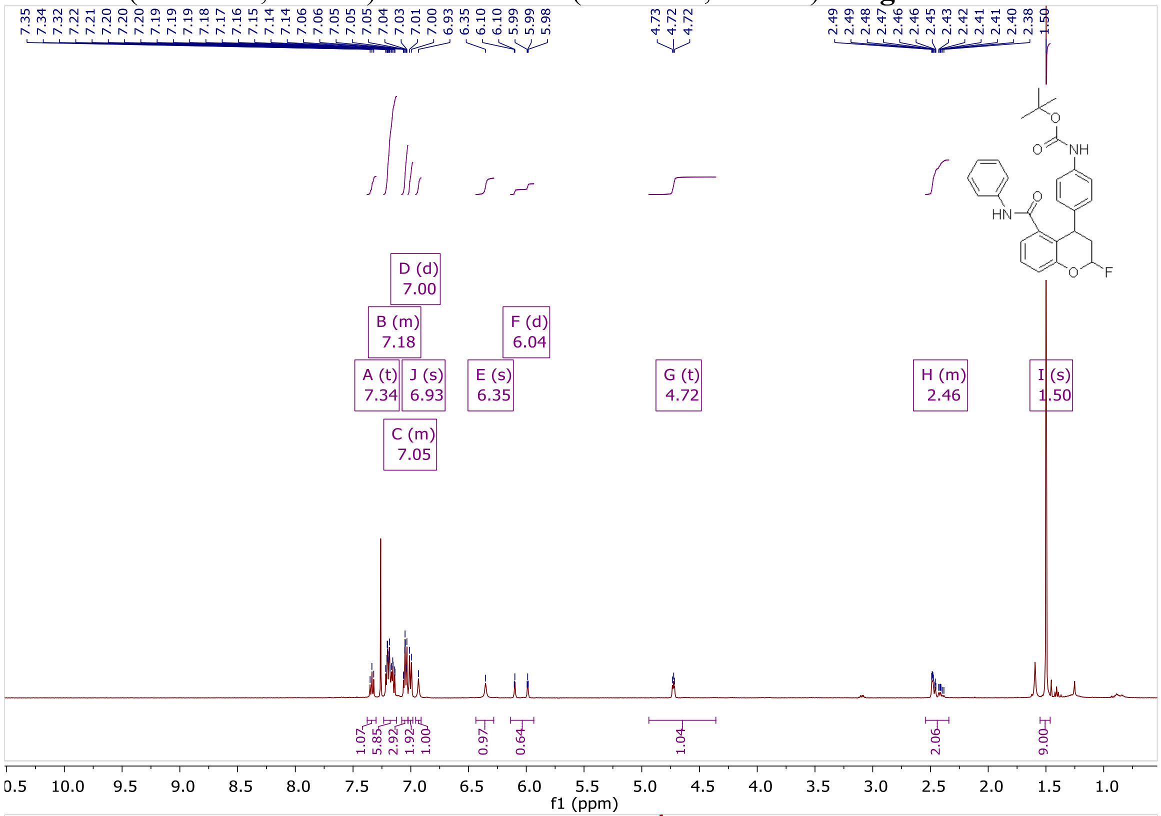

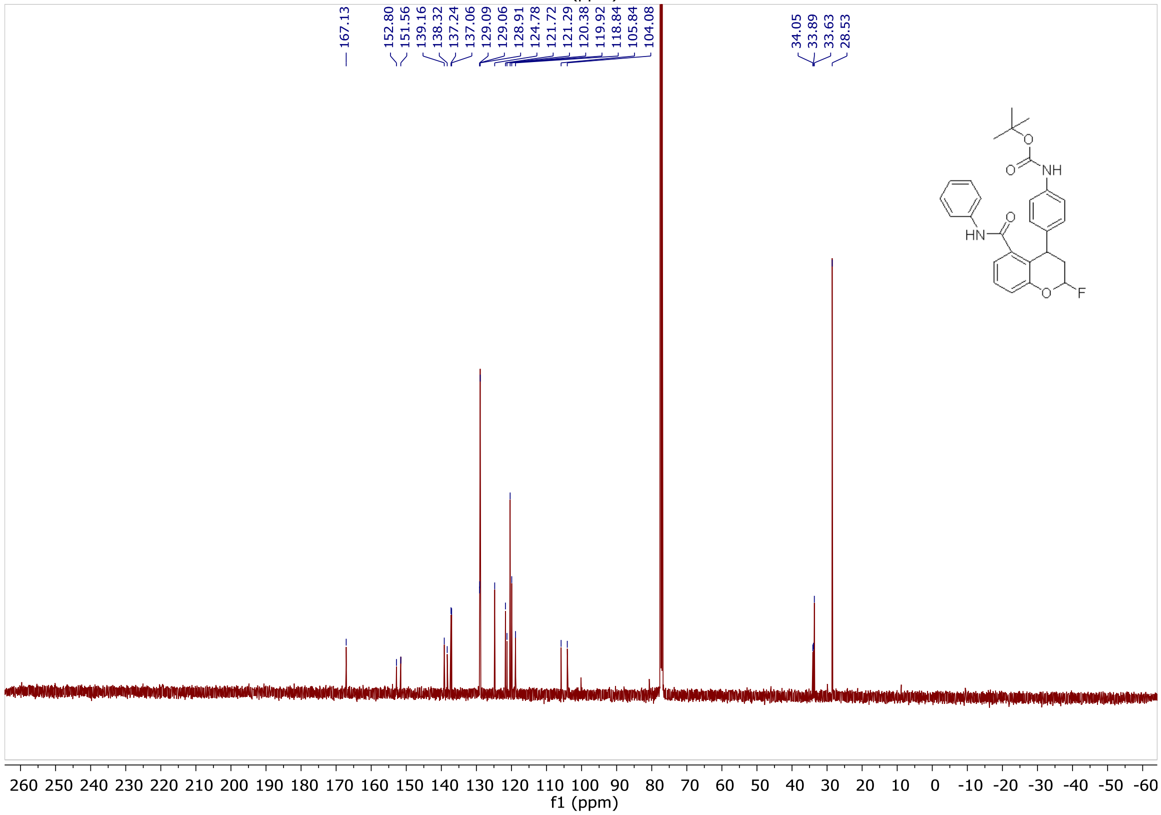

^{1}H NMR (600 MHz, CDCl3) and ^{13}C NMR (151 MHz, CDCl3) of **2f :**

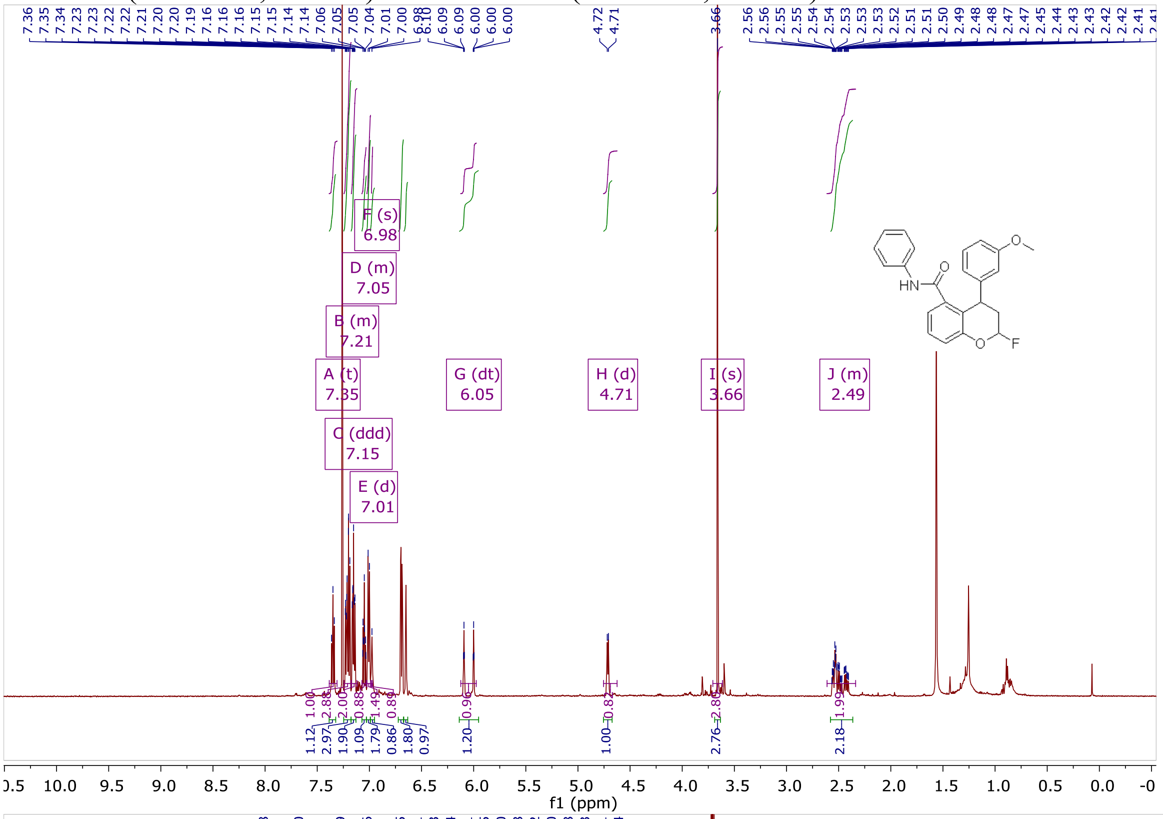

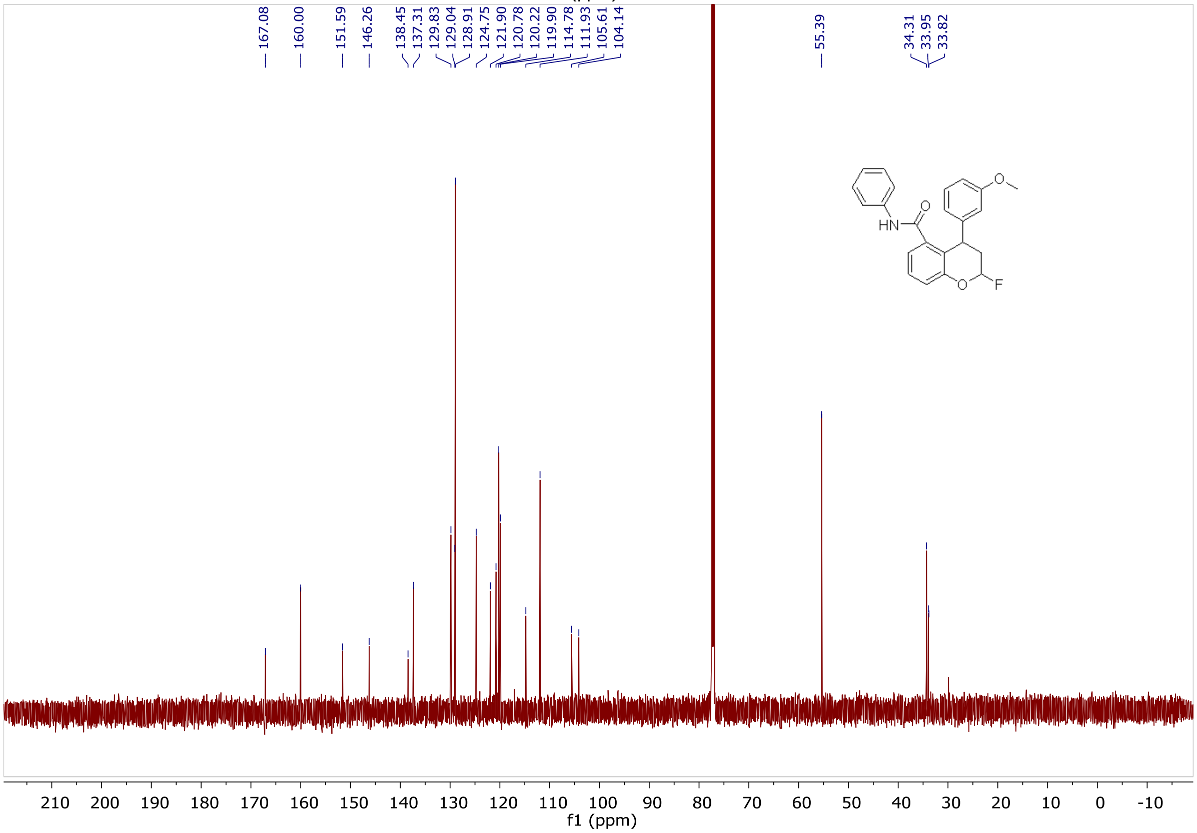

^{1}H NMR (500 MHz, CDCl3) and ^{13}C NMR (126 MHz, CDCl3) of **3**:

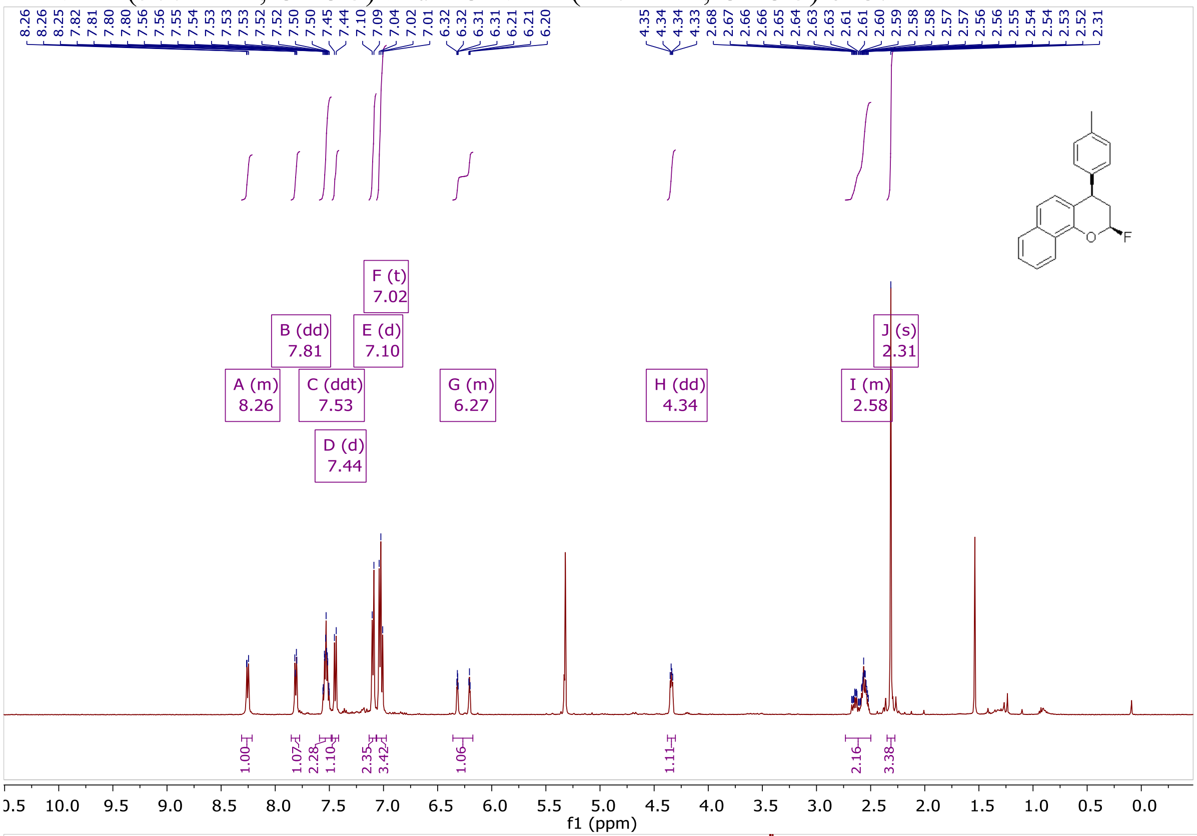

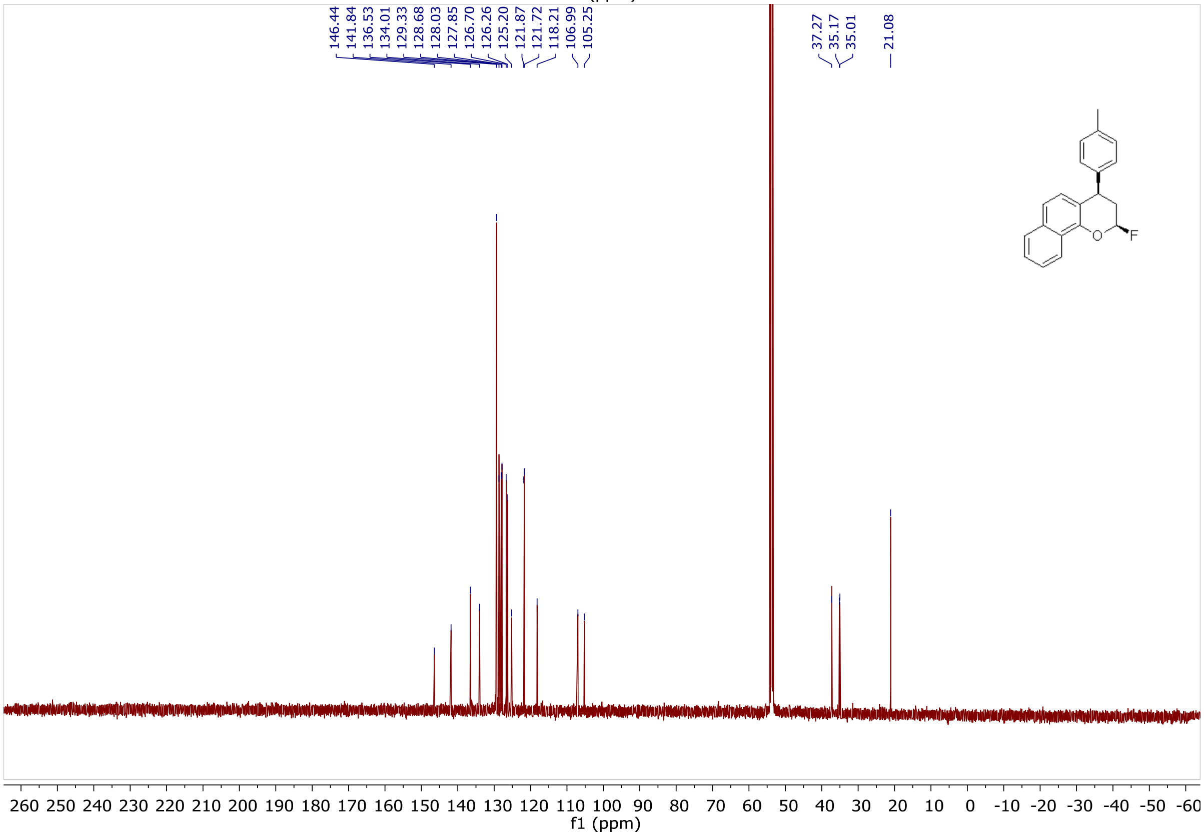

^{1}H NMR (500 MHz, CDCl3) and ^{13}C NMR (126 MHz, CDCl3) of **4**:

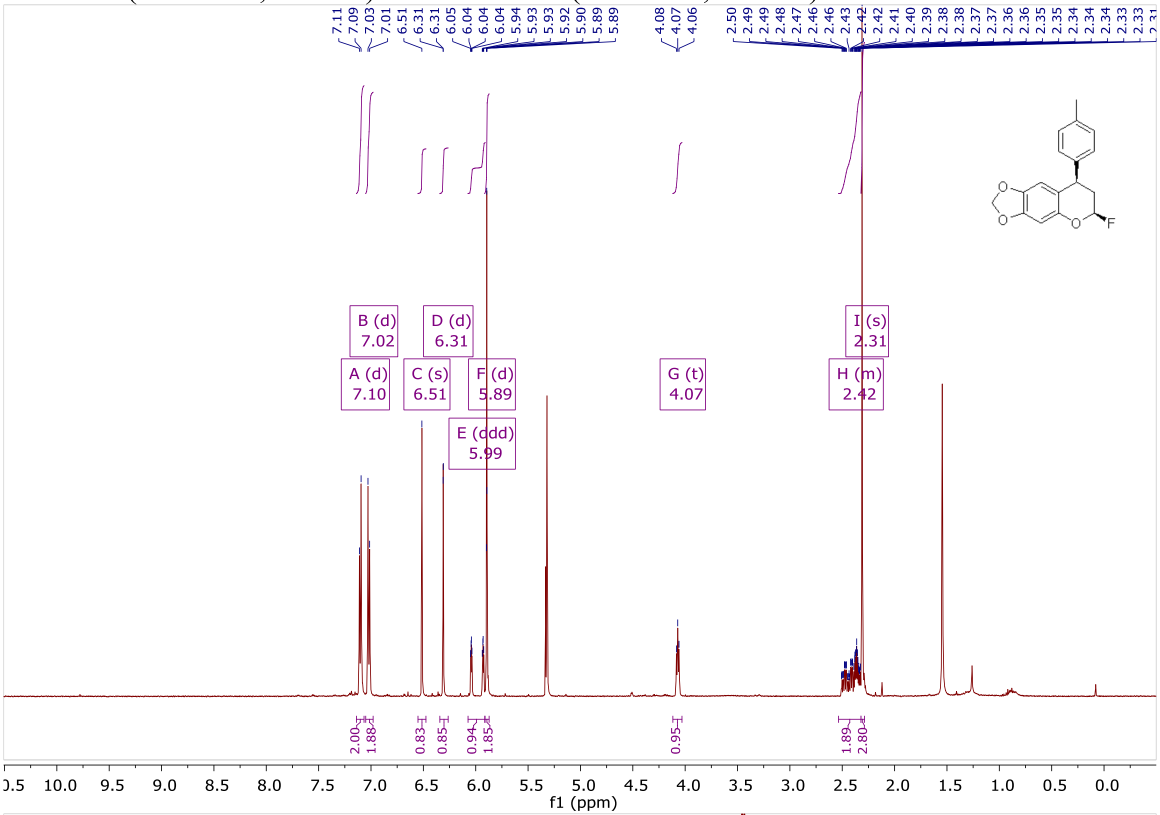

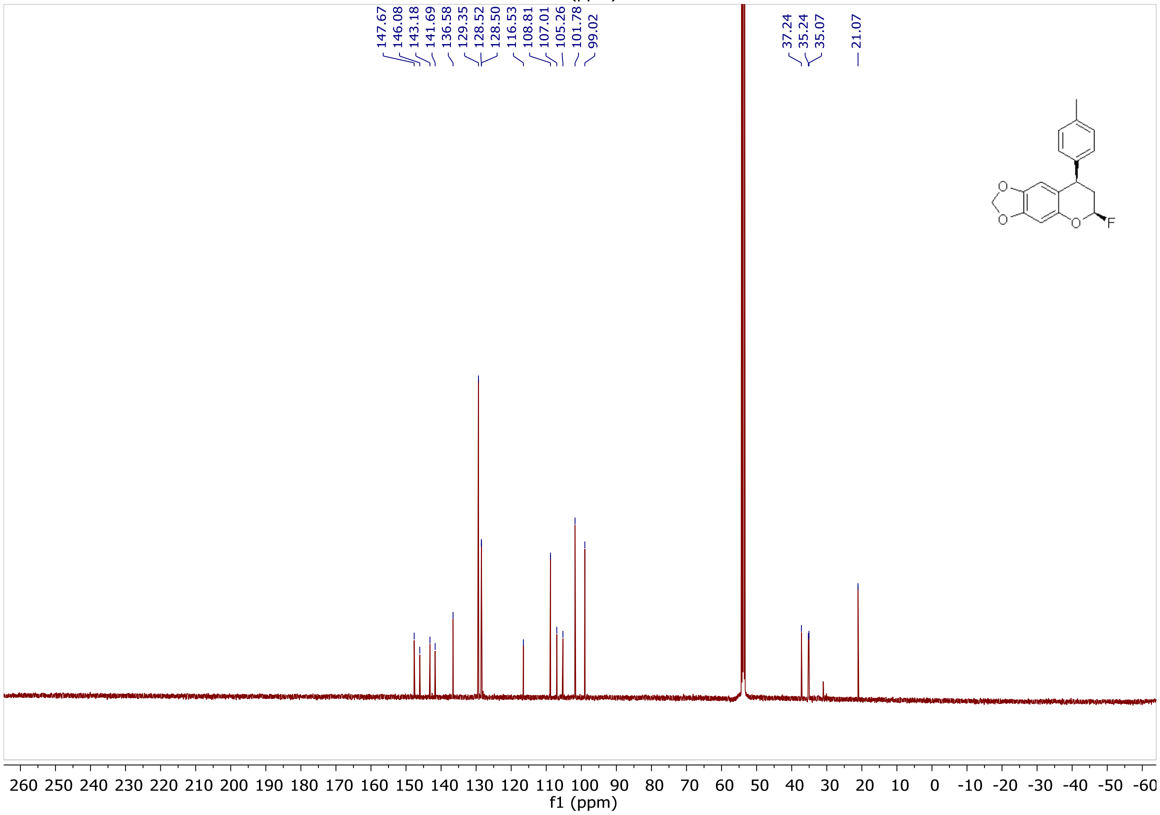

^{1}H NMR (600 MHz, CDCl3) and ^{13}C NMR (151 MHz, CDCl3) of **5**:

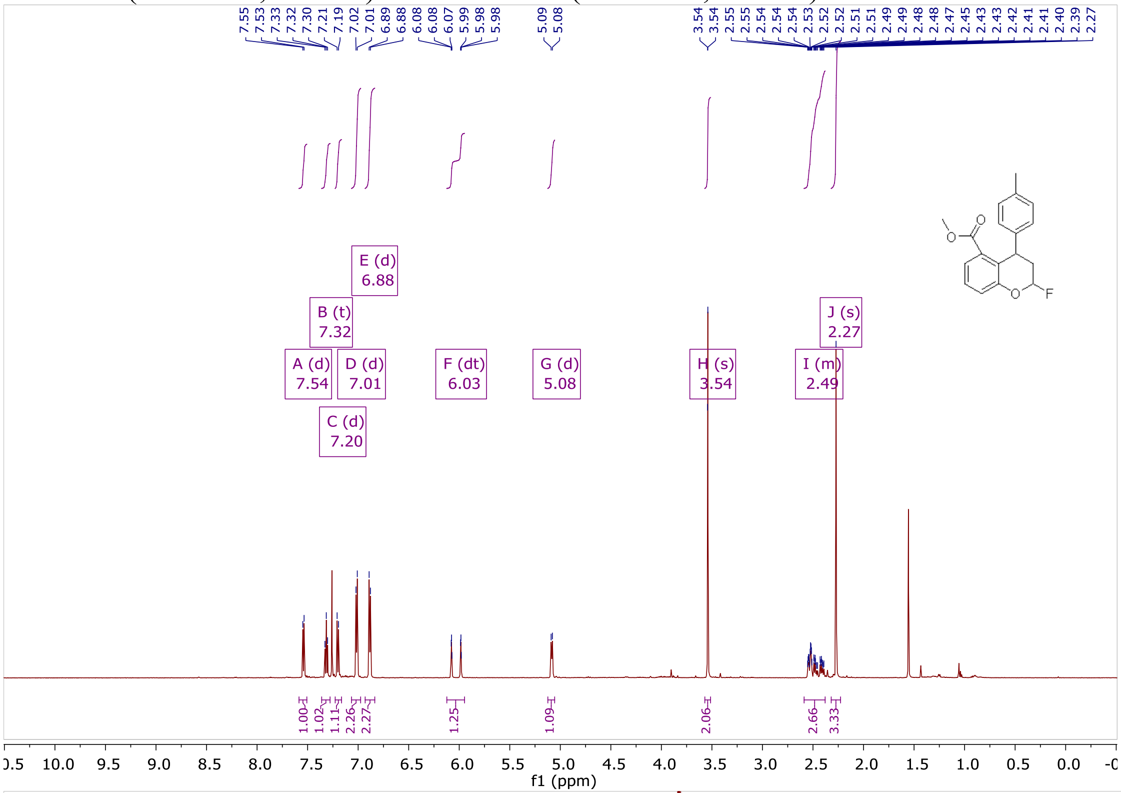

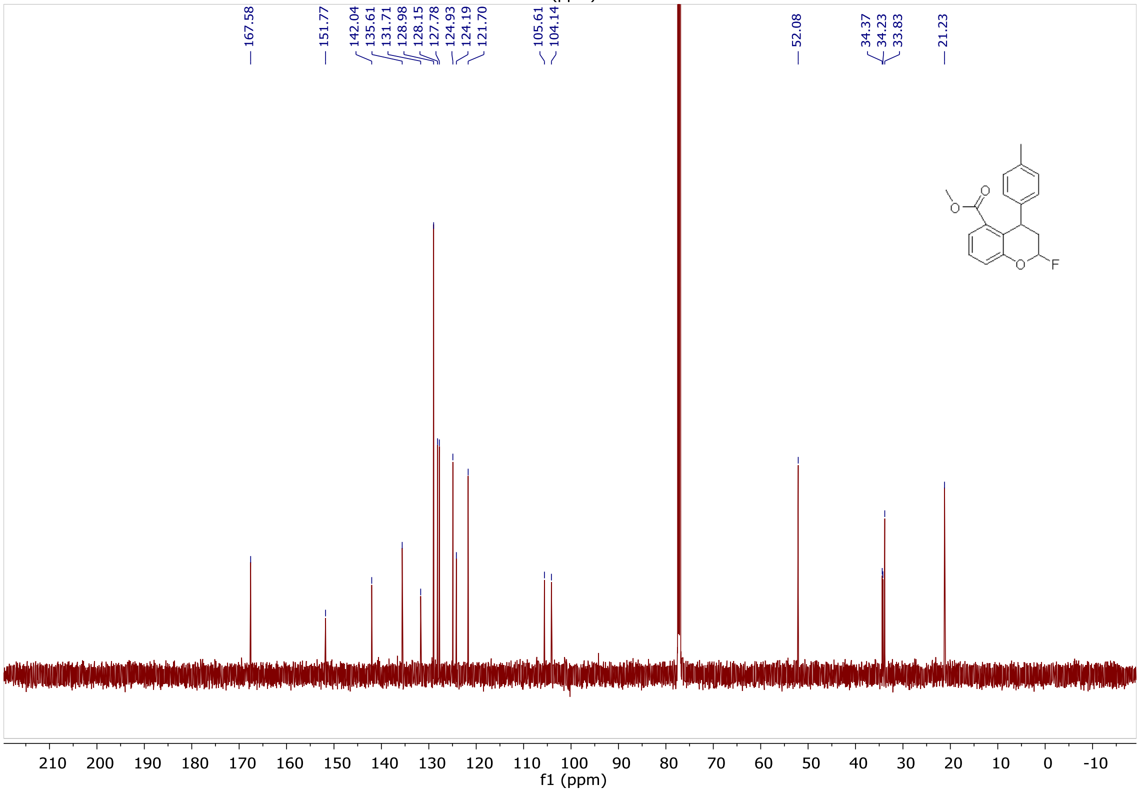

^{1}H NMR (400 MHz, CDCl3) and ^{13}C NMR (101 MHz, CDCl3) of $\mathbf{d_2}$**-1**

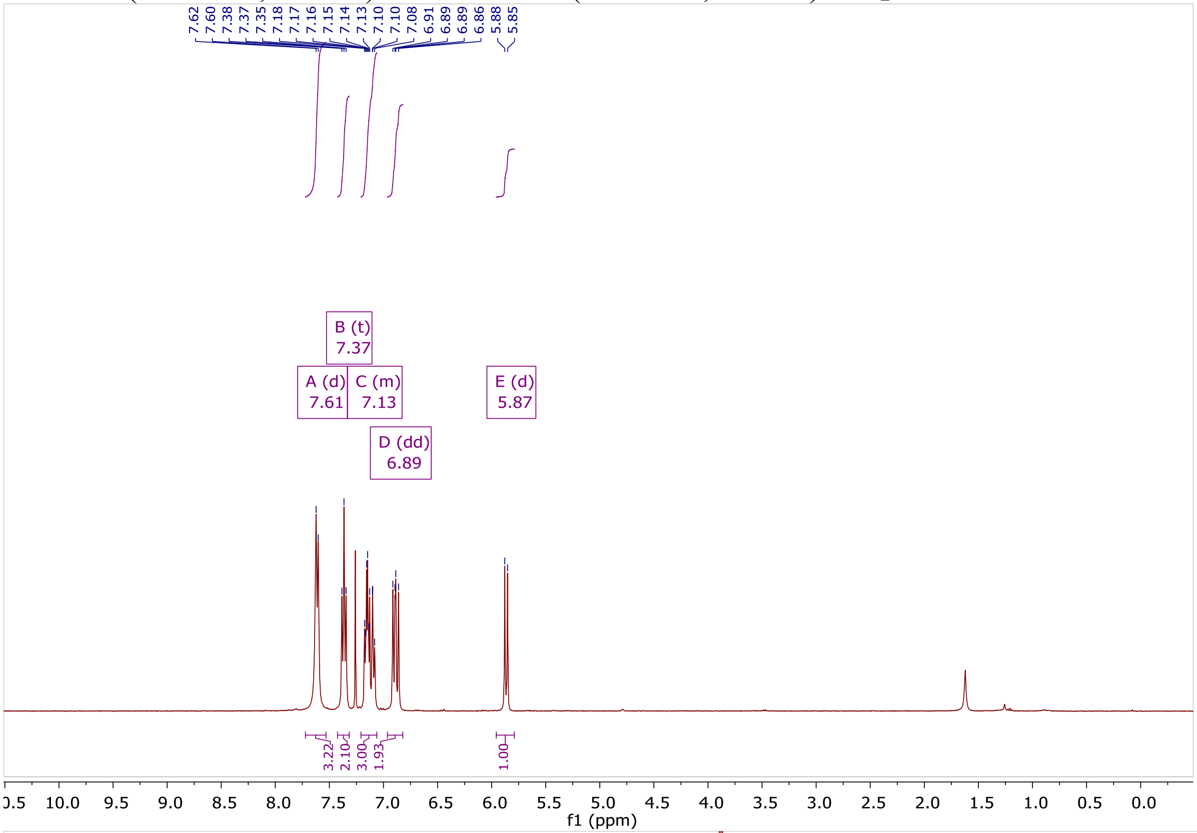

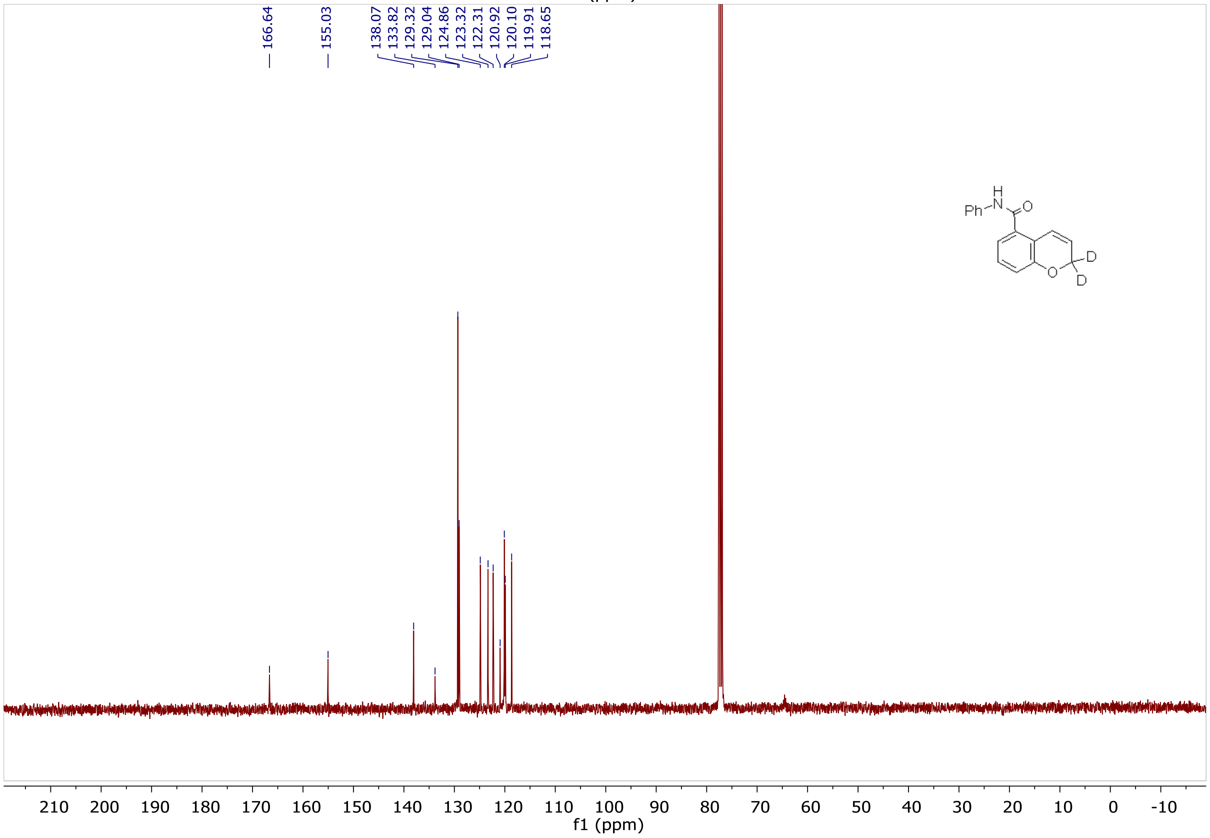

^{1}H NMR (500 MHz, CDCl3) and ^{13}C NMR (126 MHz, CDCl3) of **d$_2$-2a**

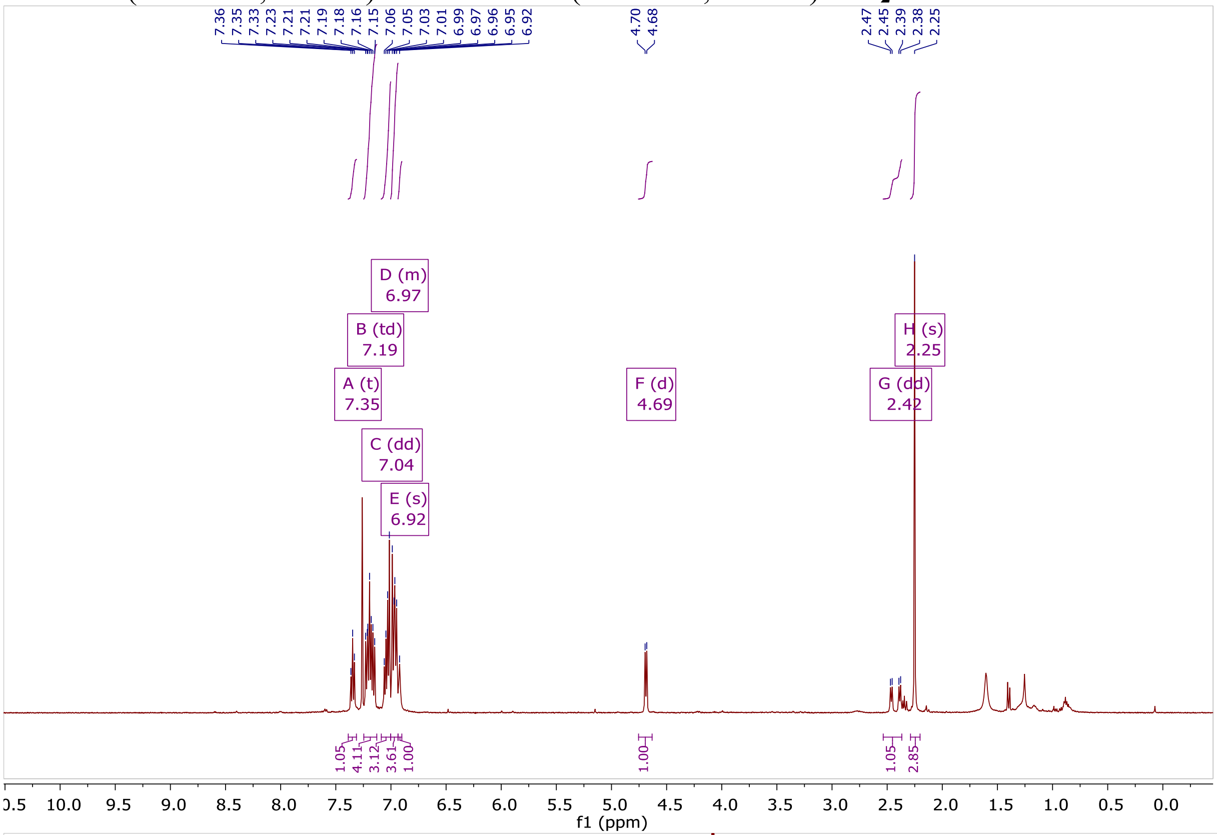

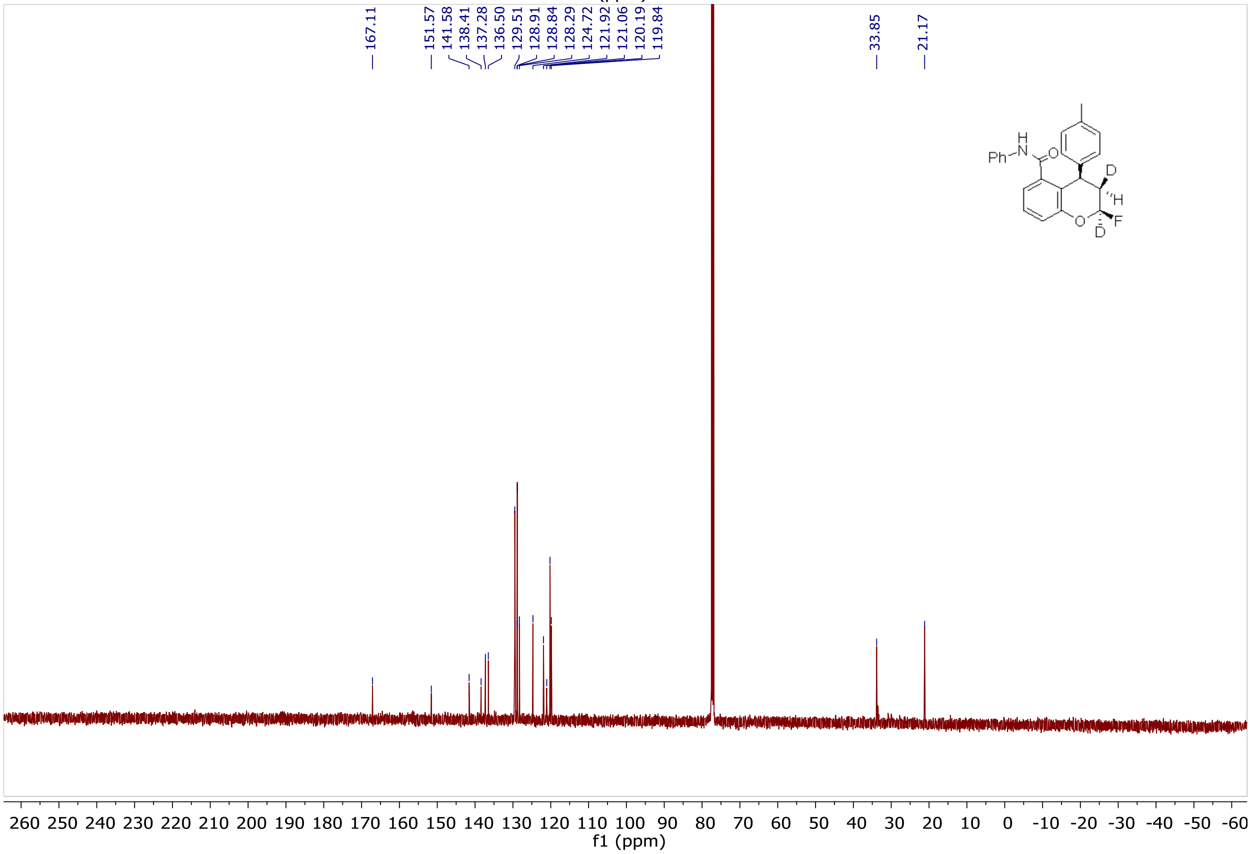

^{1}H 2D NOESY spectrum of **d_2-2a** :

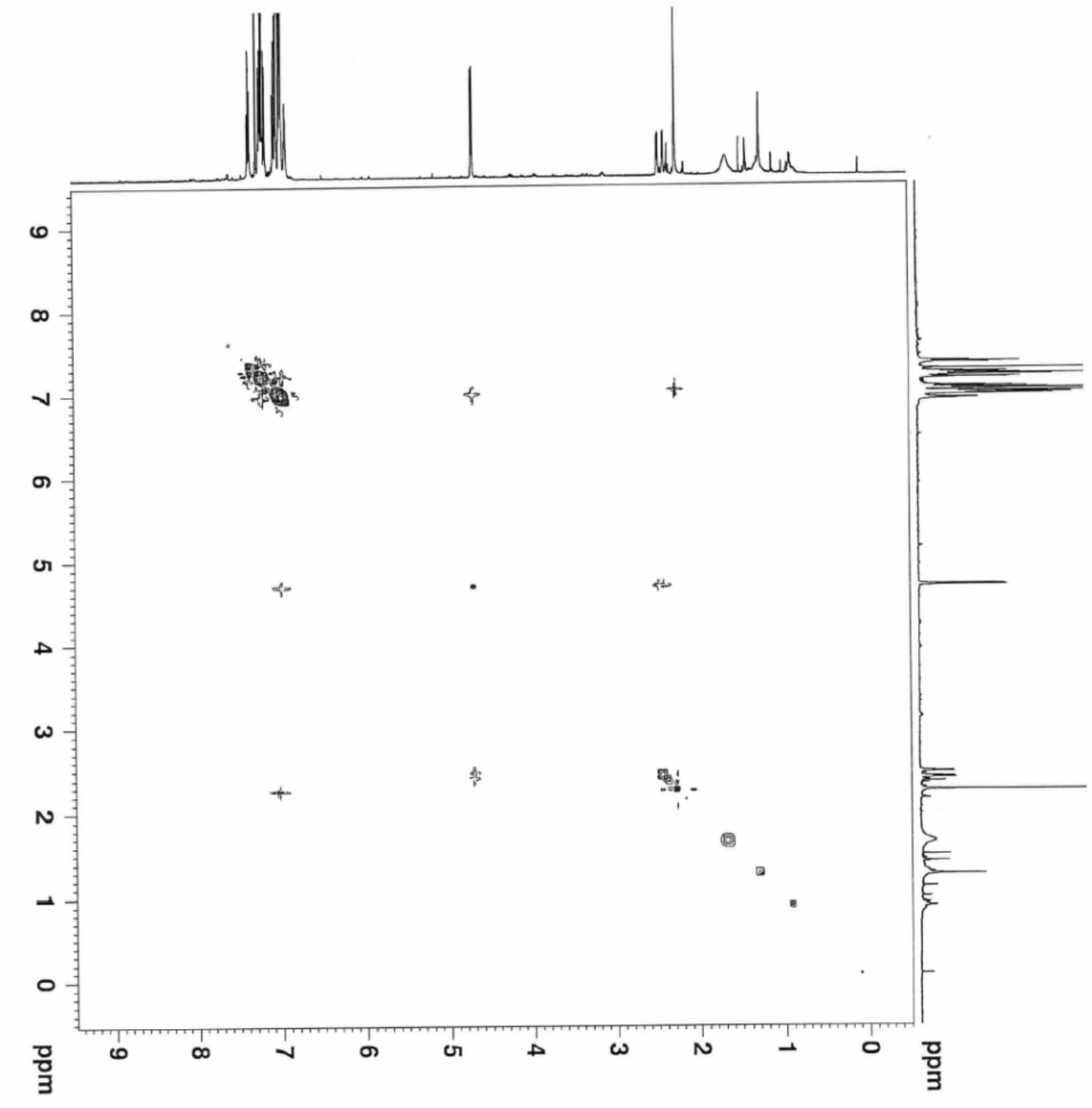

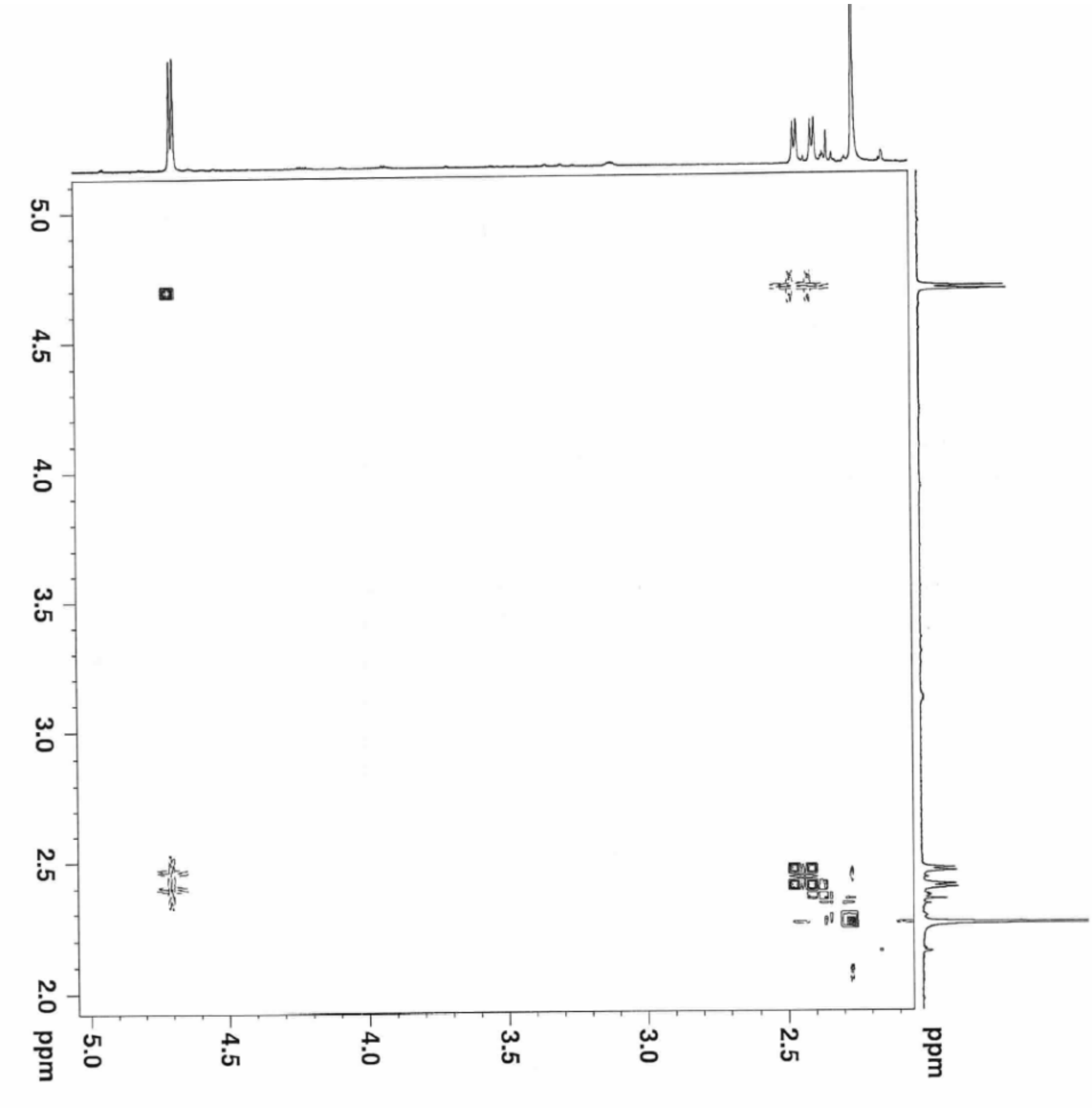
5.0
4.5
4.0
3.5
3.0
2.5
2.0
ppm
5.0
4.5
4.0
3.5
3.0
2.5
ppm

^{1}H NMR (500 MHz, $CDCl_3$) and ^{13}C NMR (126 MHz, $CDCl_3$) of **9a:**

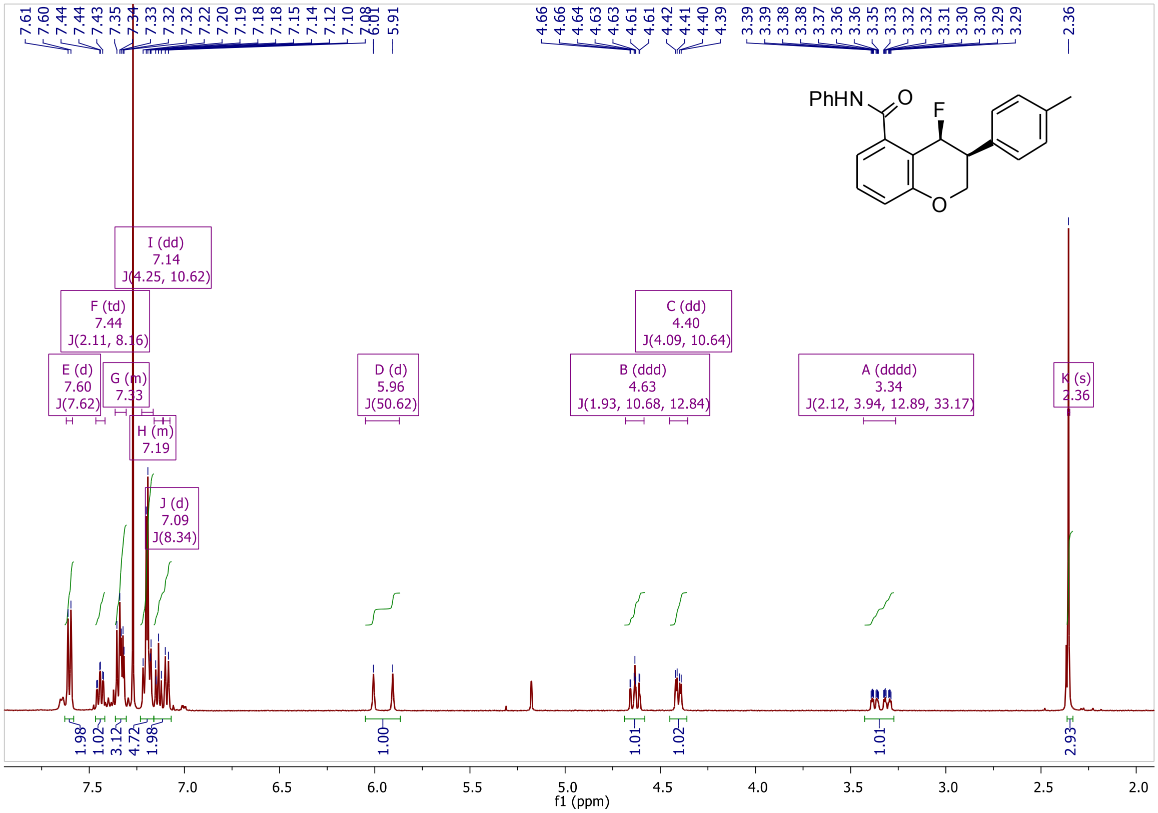

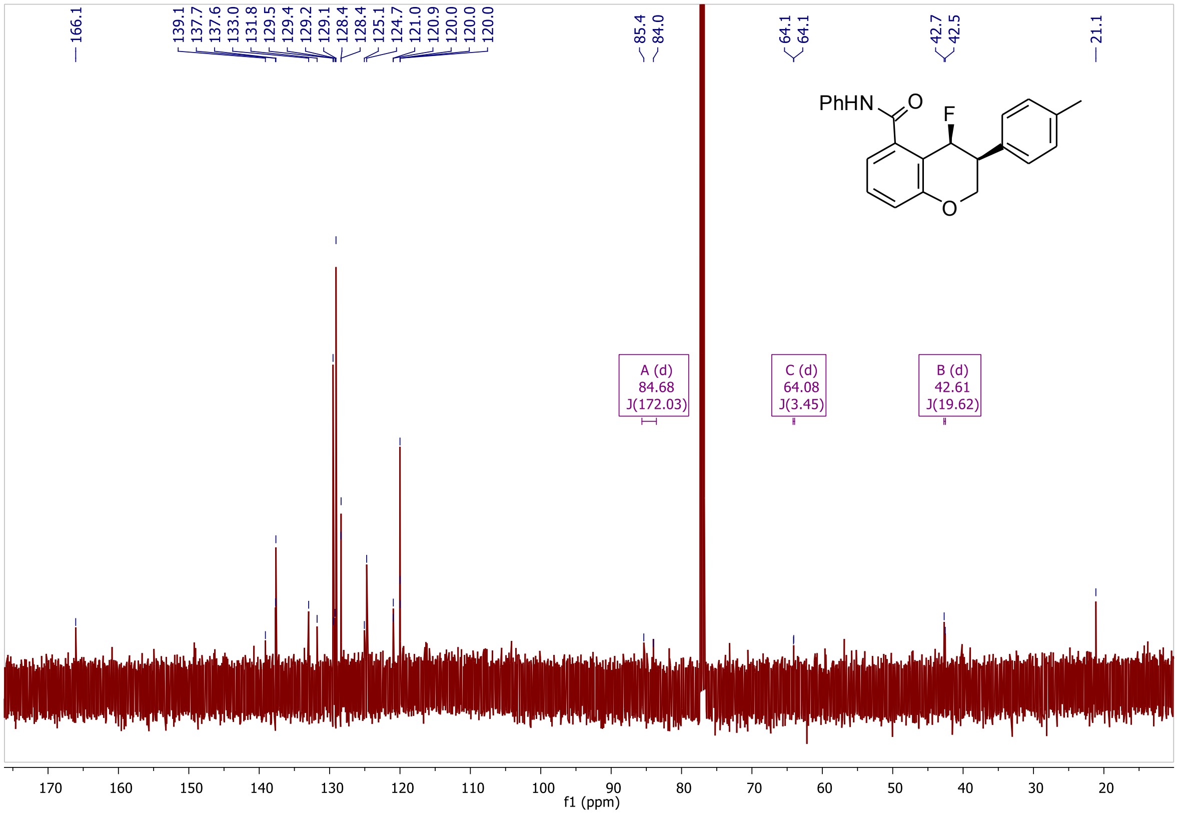

^{1}H NMR (600 MHz, $CDCl_3$) and ^{13}C NMR (151 MHz, $CDCl_3$) of **9b:**

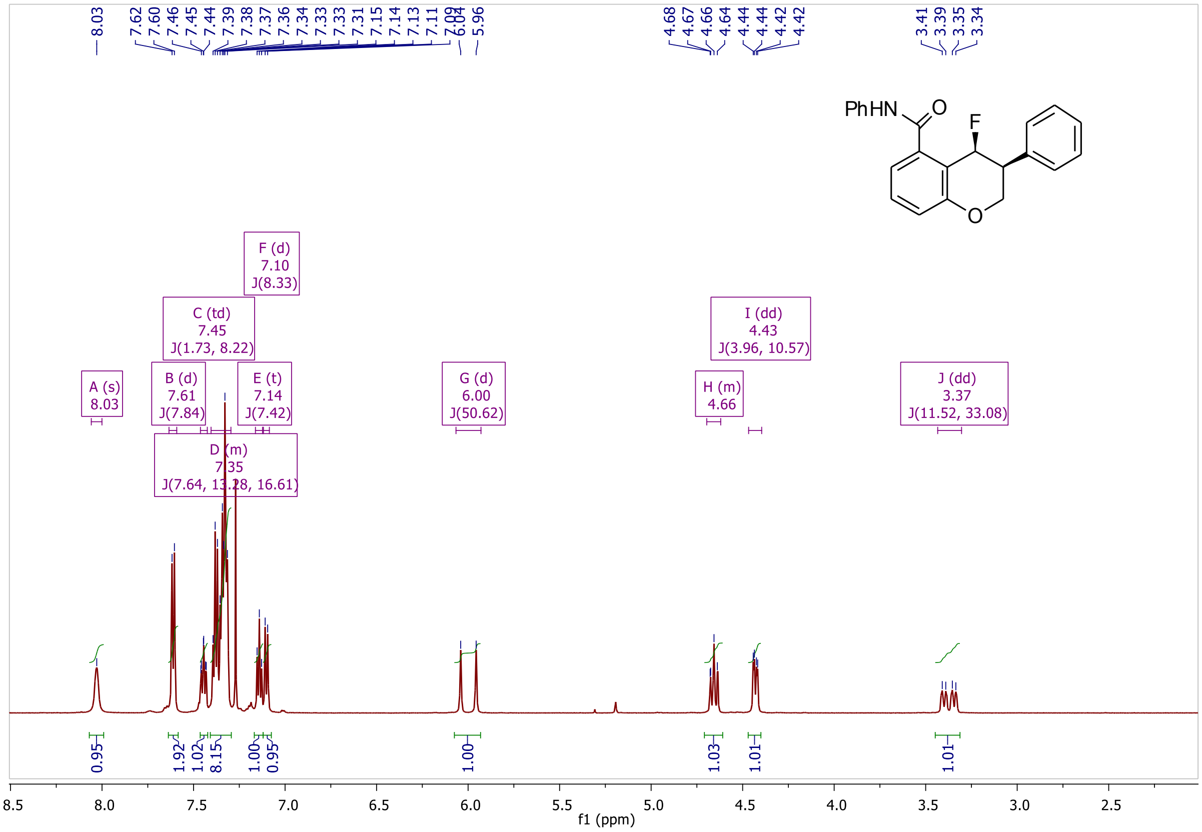

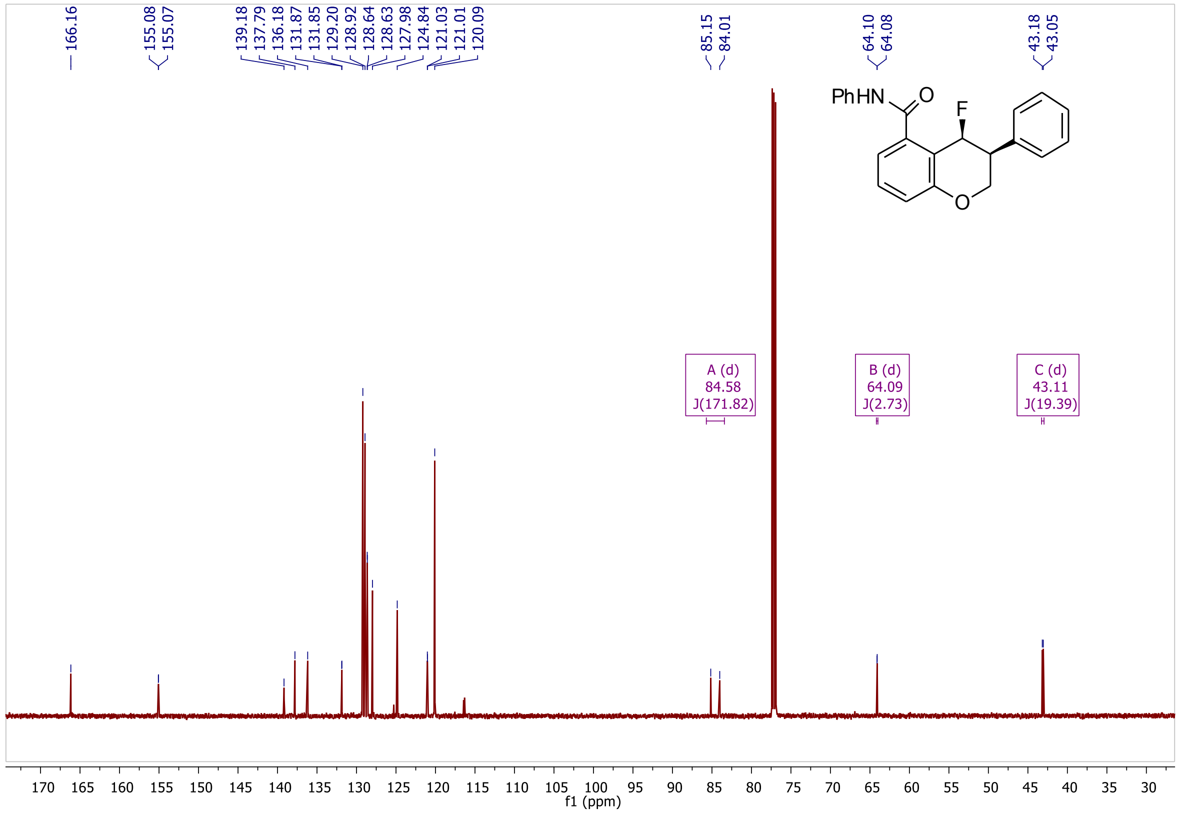

^{1}H NMR (400 MHz, $CDCl_3$) and ^{13}C NMR (151 MHz, $CDCl_3$) of **9c:**

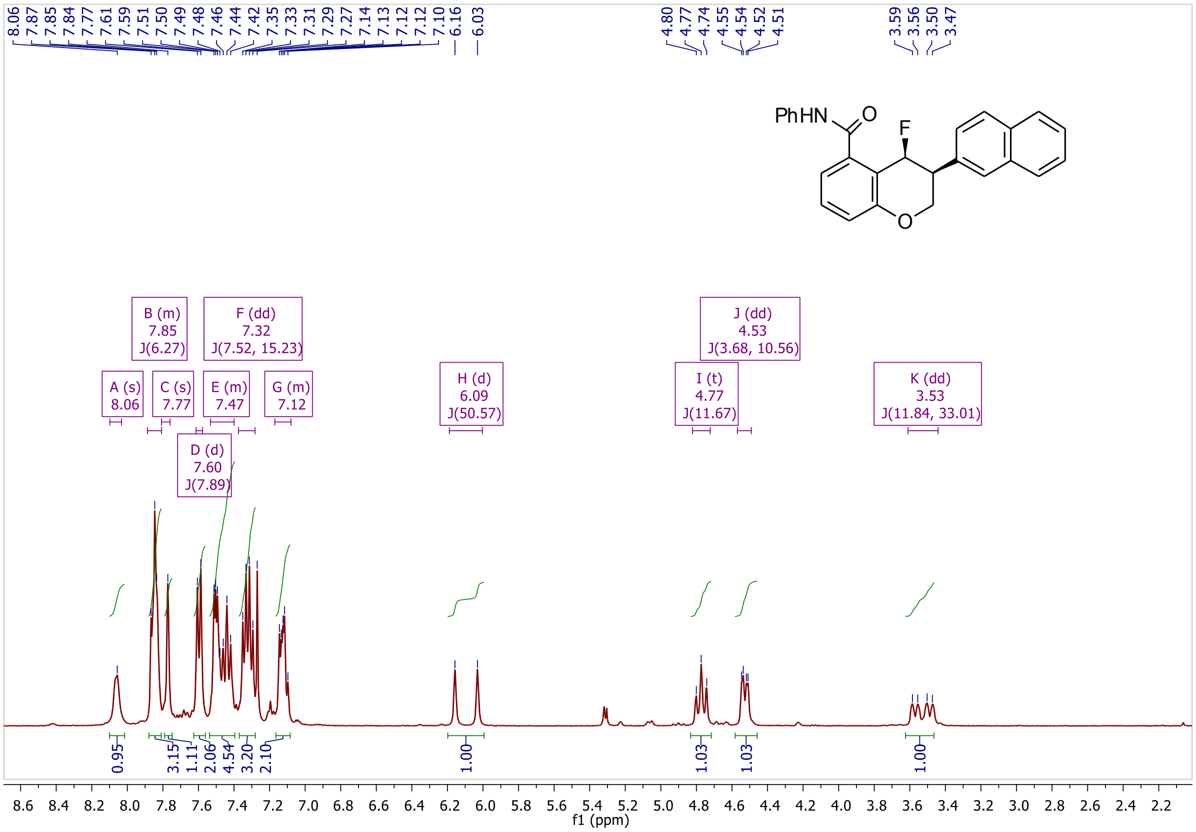

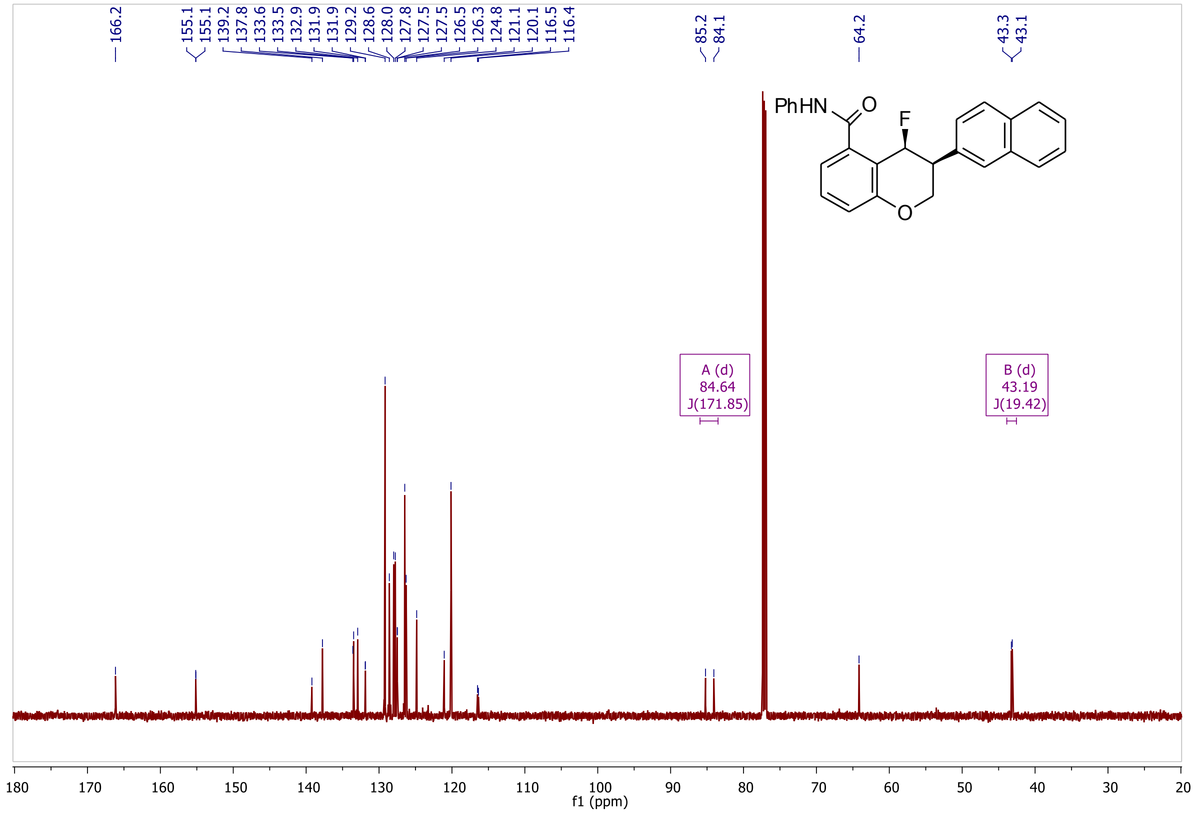

^{1}H NMR (400 MHz, CD_2Cl_2) and ^{13}C NMR (101 MHz, CD_2Cl_2) of **9d:**

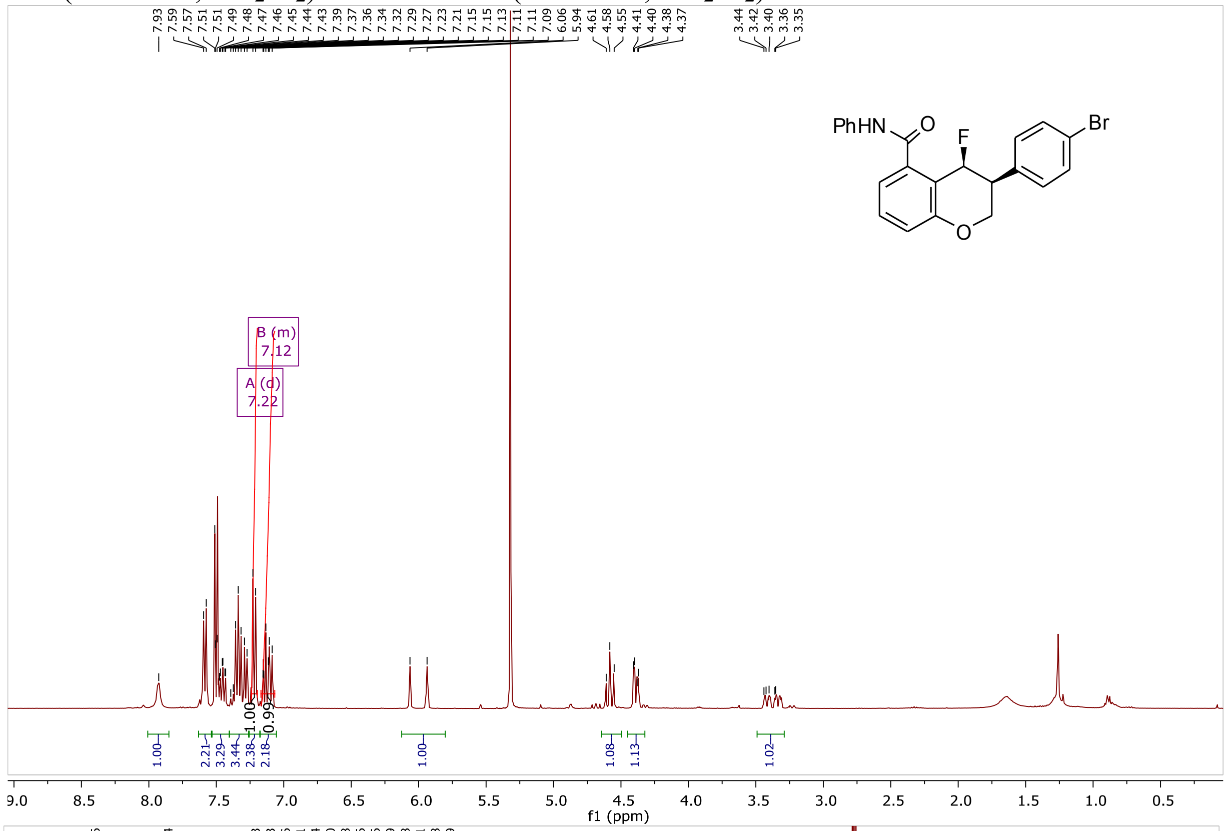

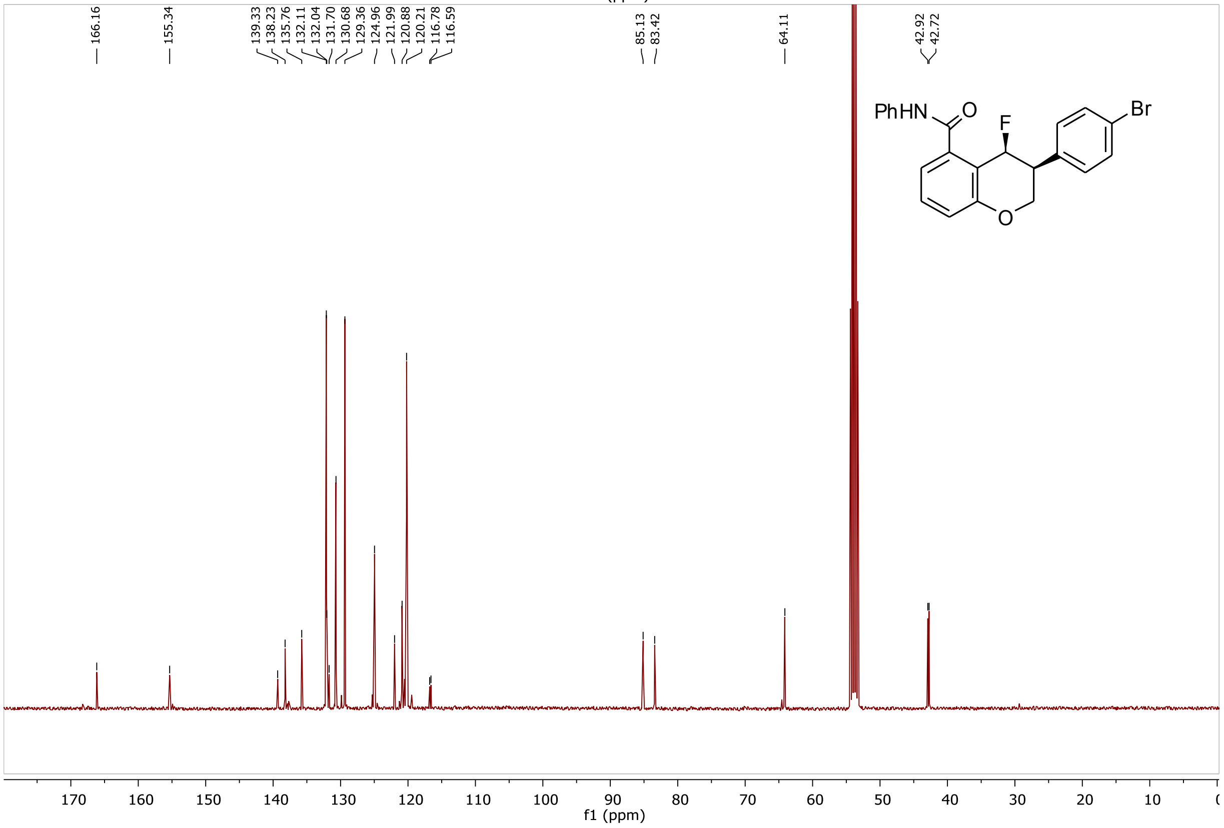

^{1}H NMR (400 MHz, CD_2Cl_2) and ^{13}C NMR (101 MHz, CD_2Cl_2) of **9e:**

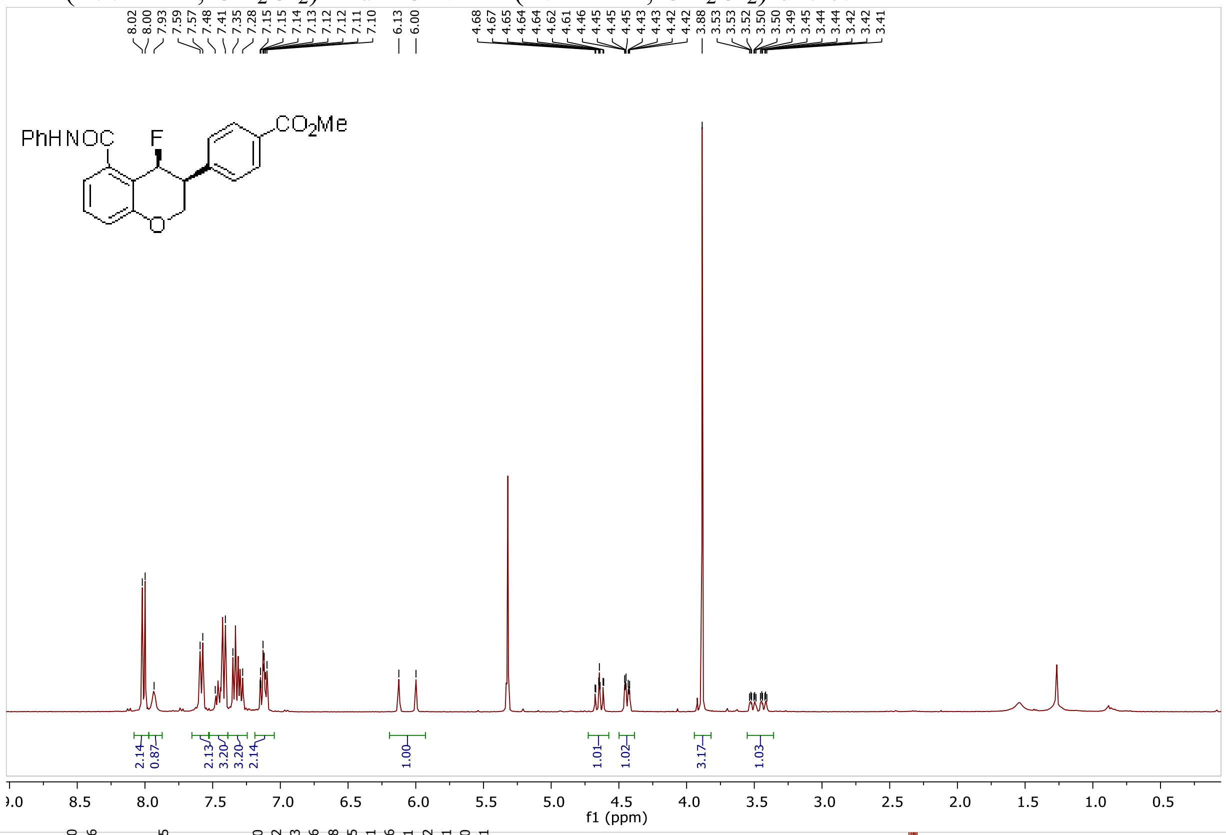

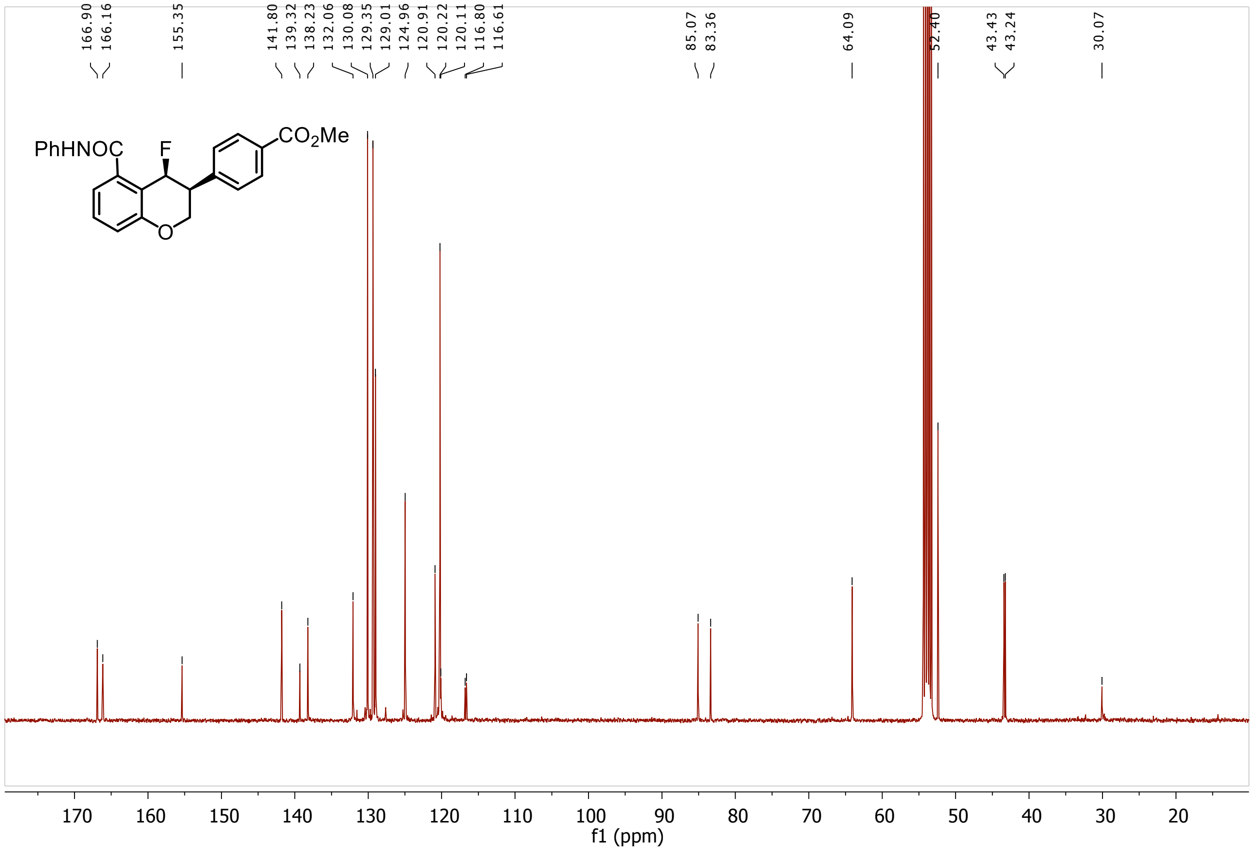

^{1}H NMR (600 MHz, CDCl3) and ^{13}C NMR (151 MHz, CDCl3) of **8**

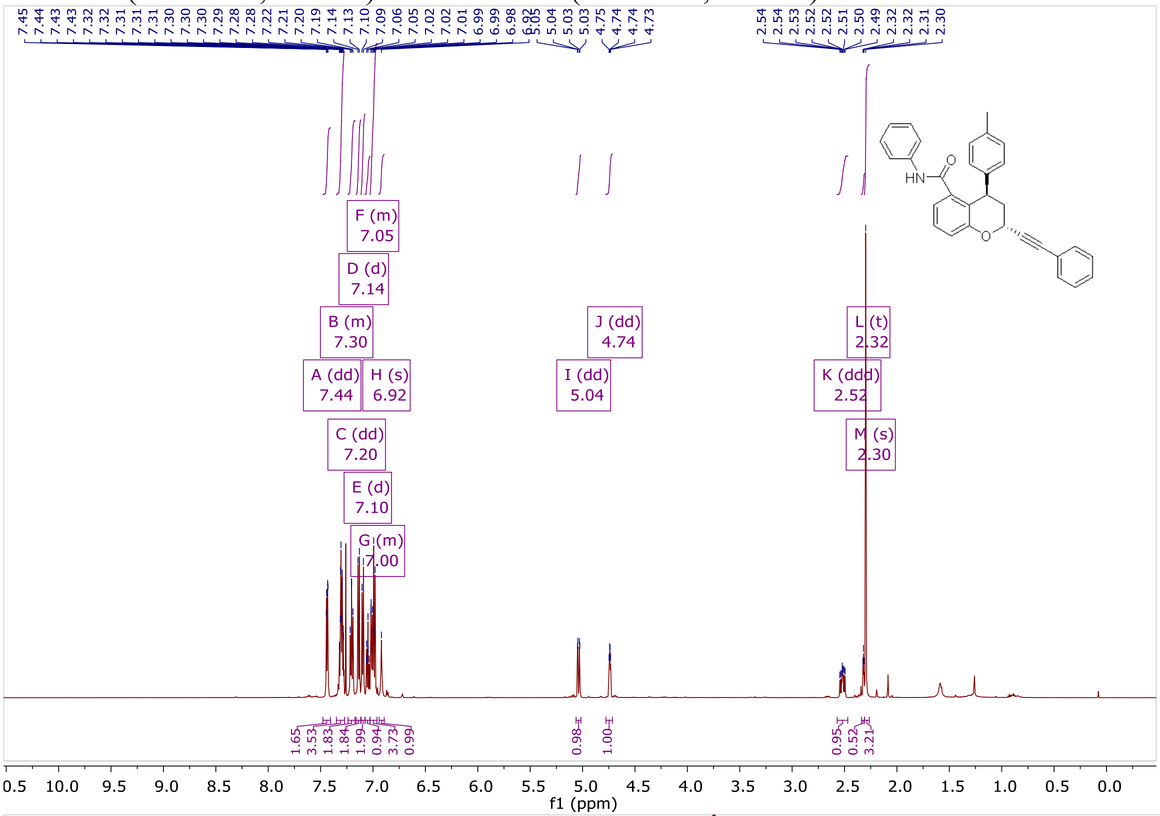

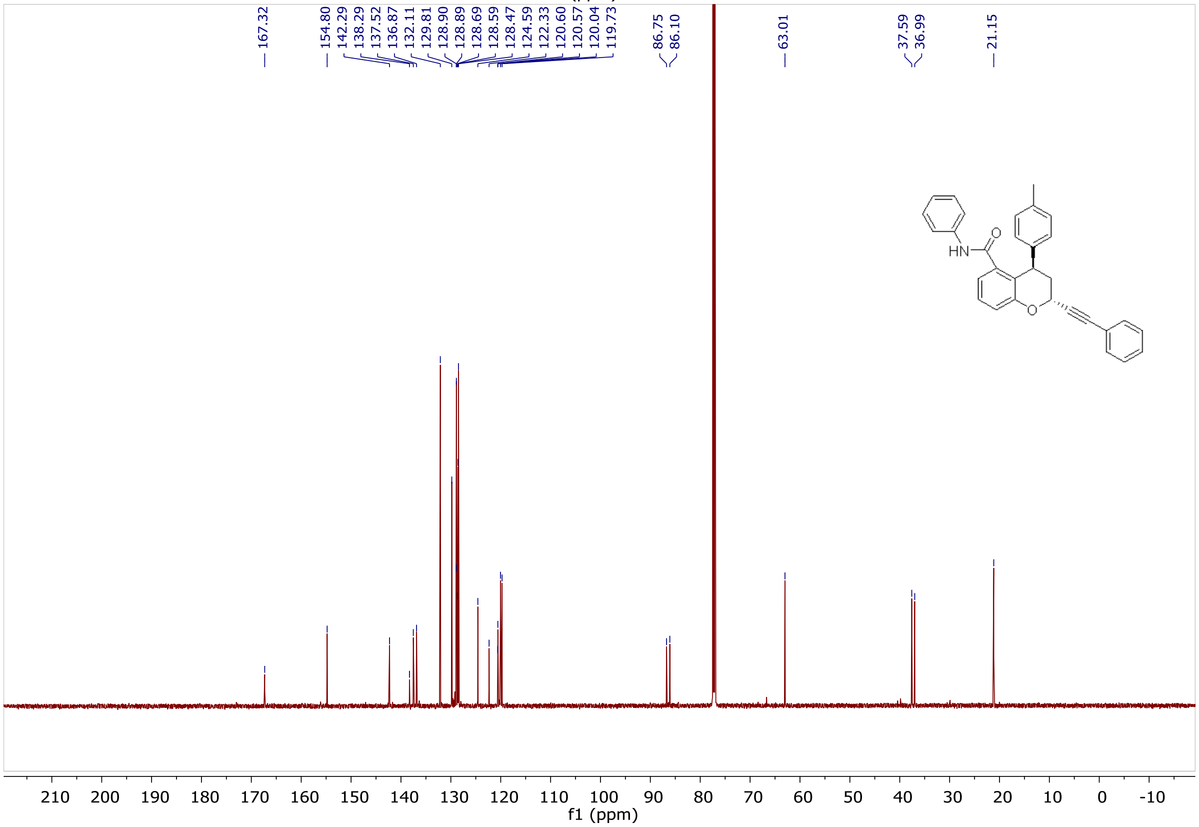

^{1}H 2D NOESY spectrum of **8**:

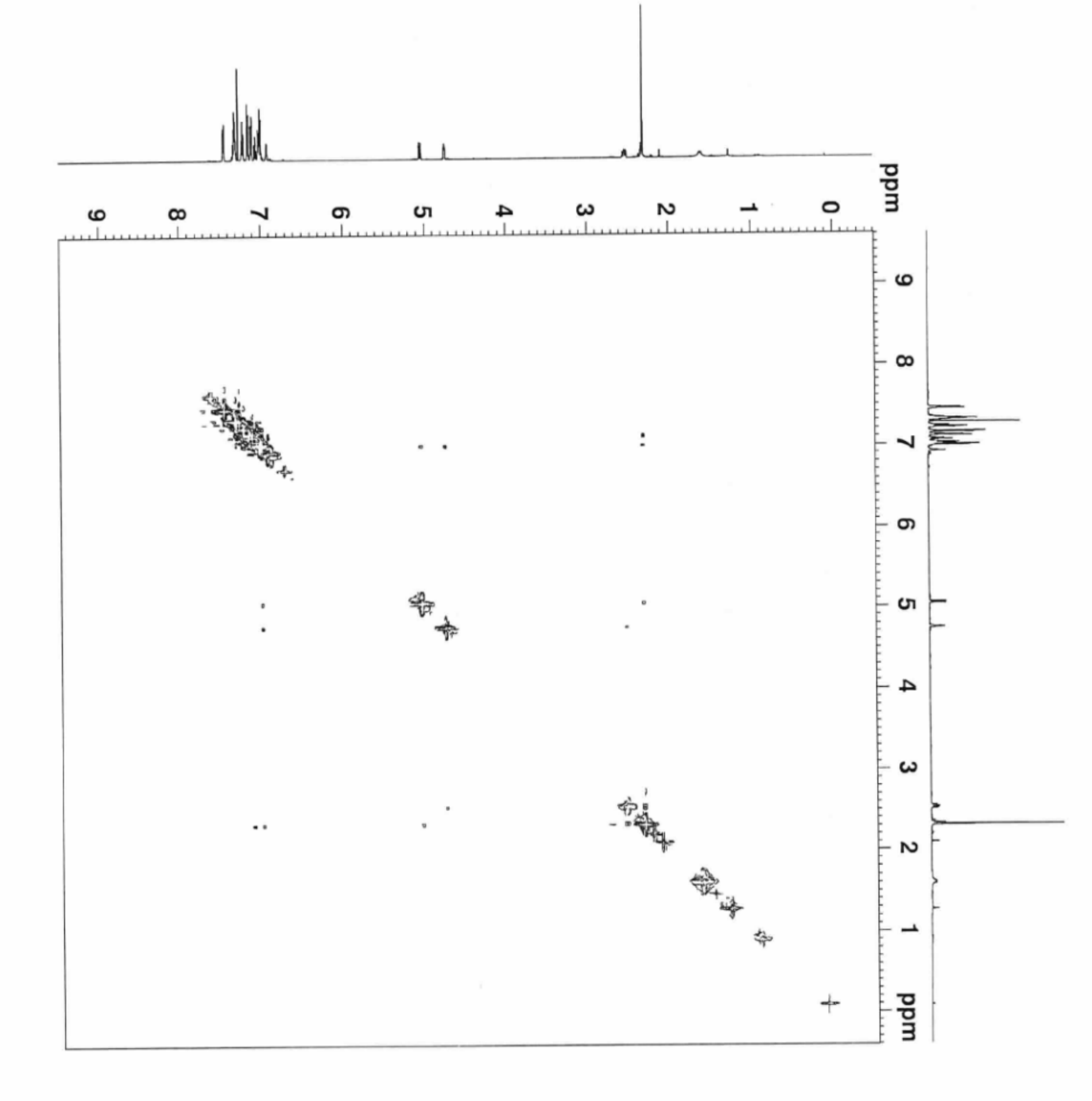

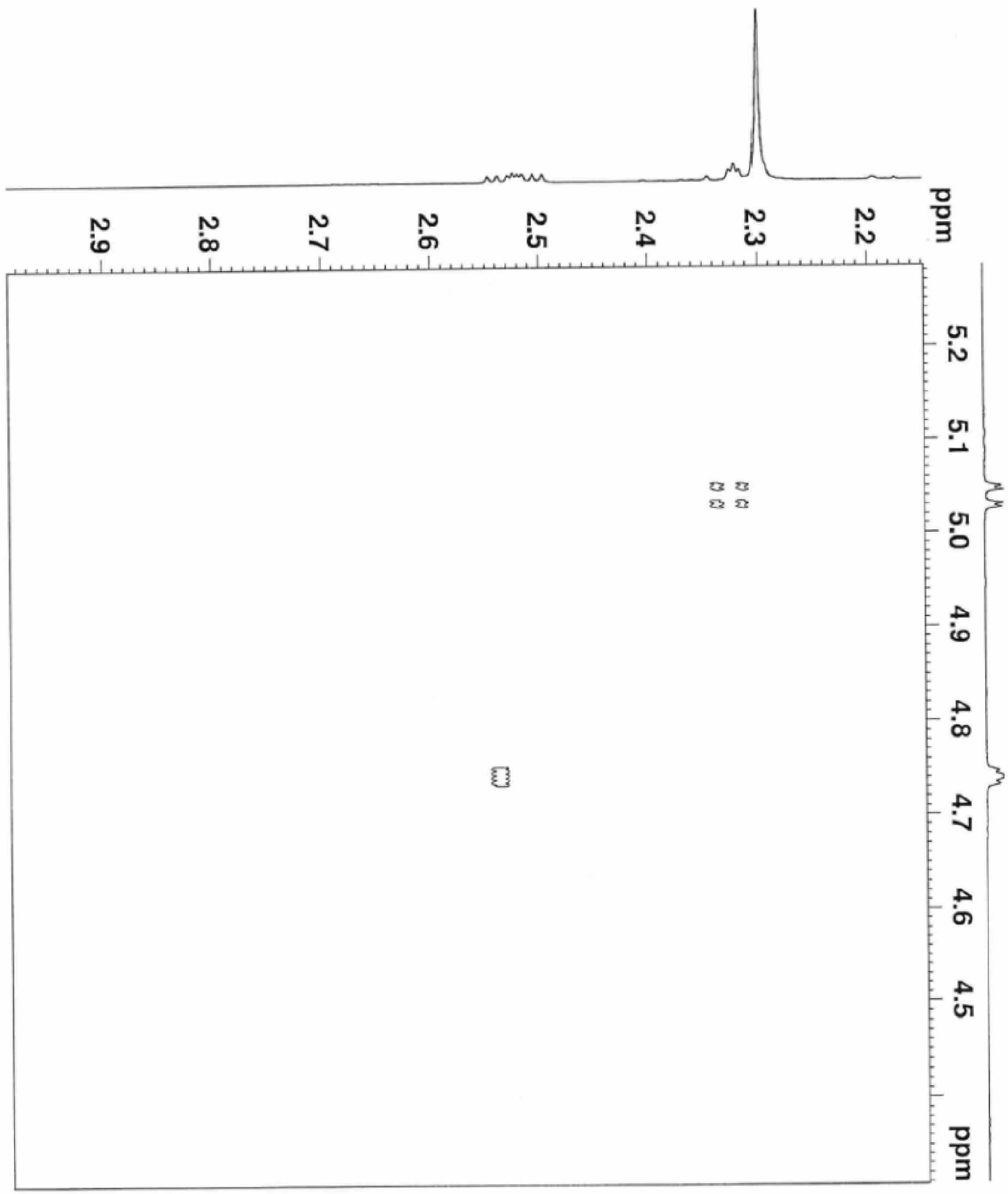
ppm
2.9
2.8
2.7
2.6
2.5
2.4
2.3
2.2
5.2
5.1
5.0
4.9
4.8
4.7
4.6
4.5
ppm

6. Copies of Chiral HPLC Analysis

HPLC Analysis of **2a**

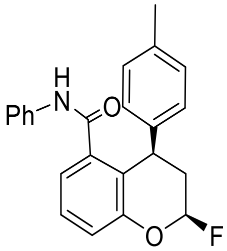

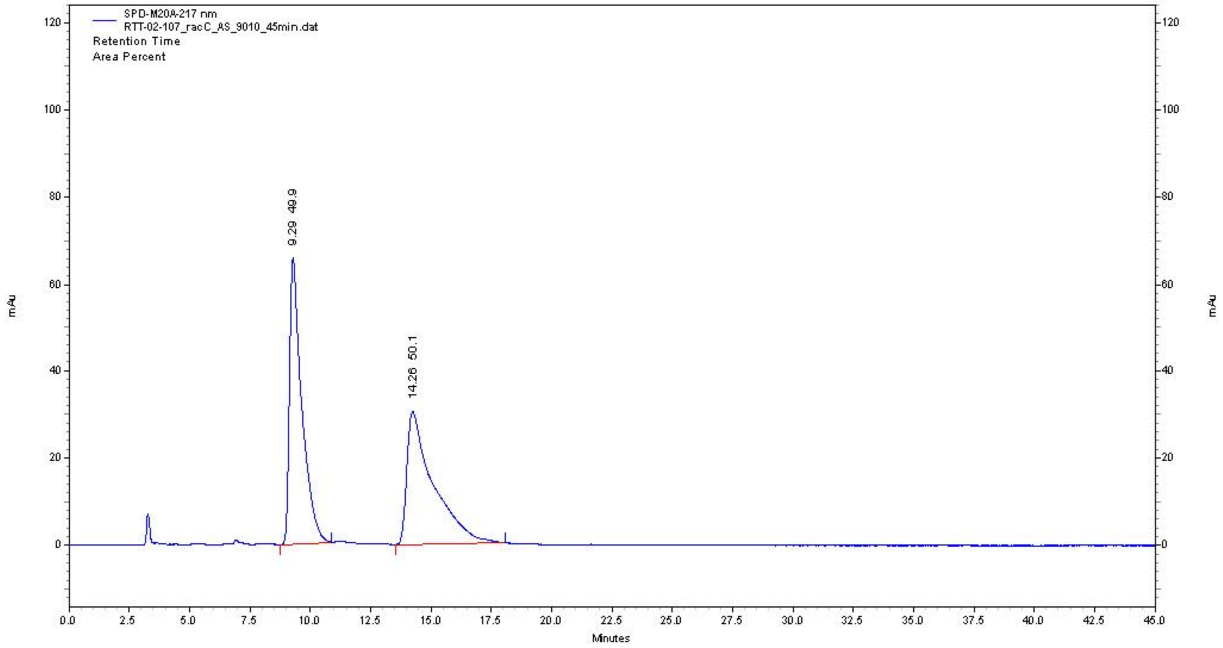

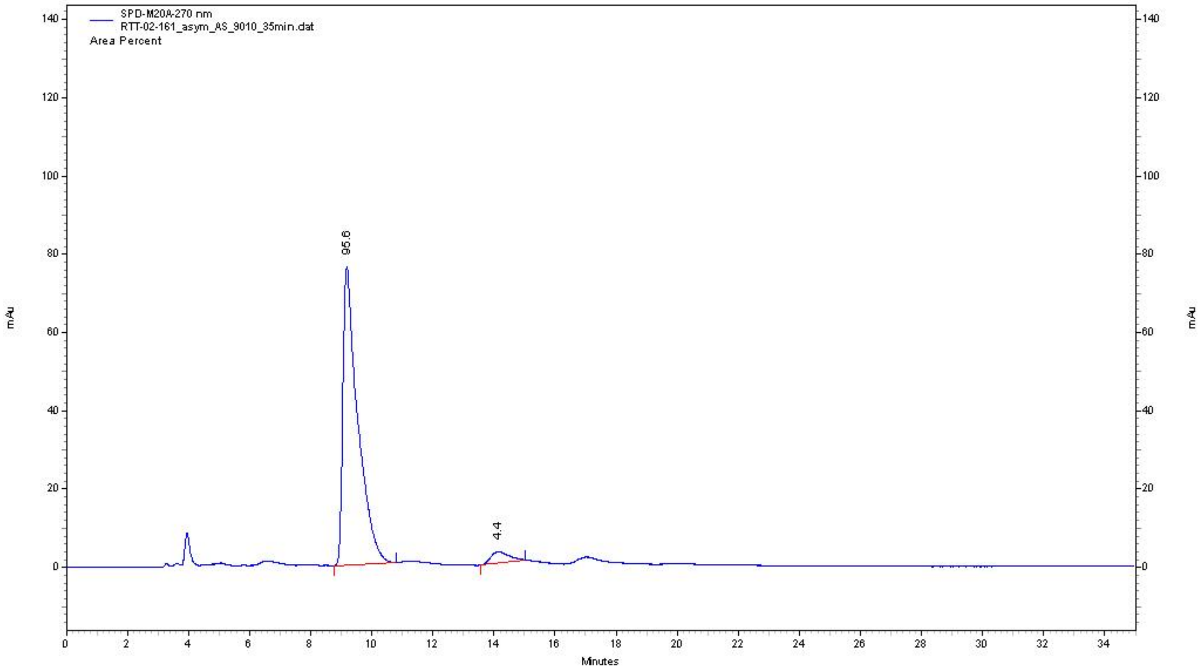

HPLC Analysis of **2b**

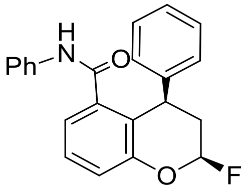

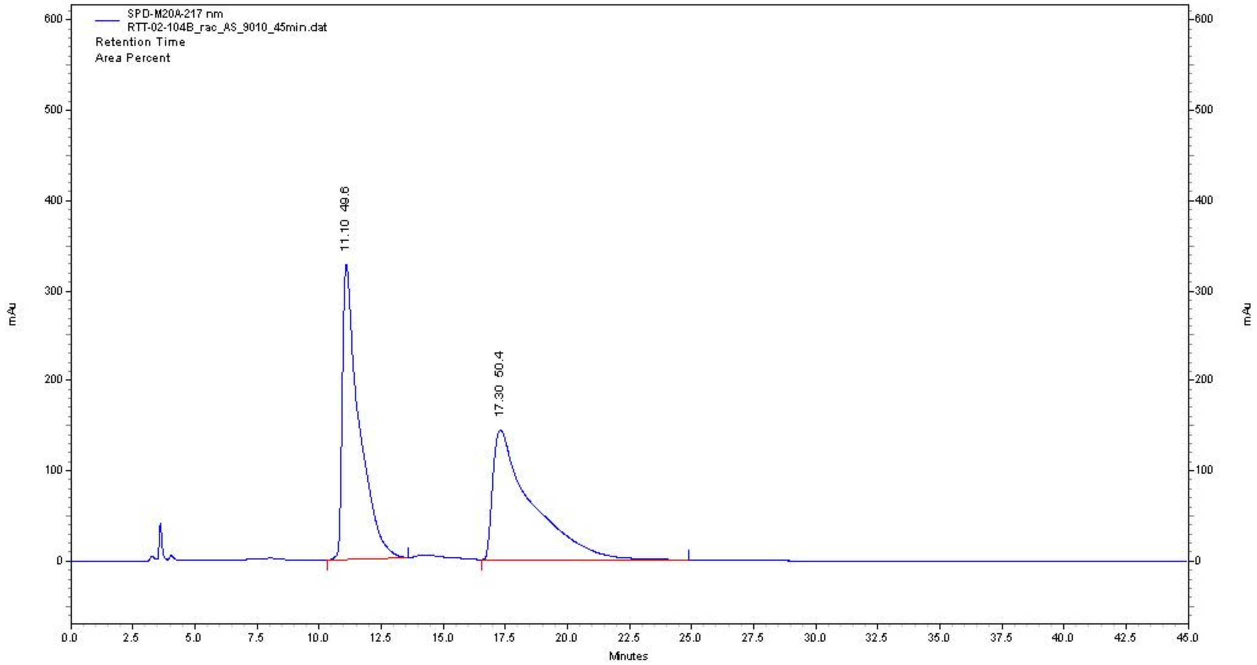

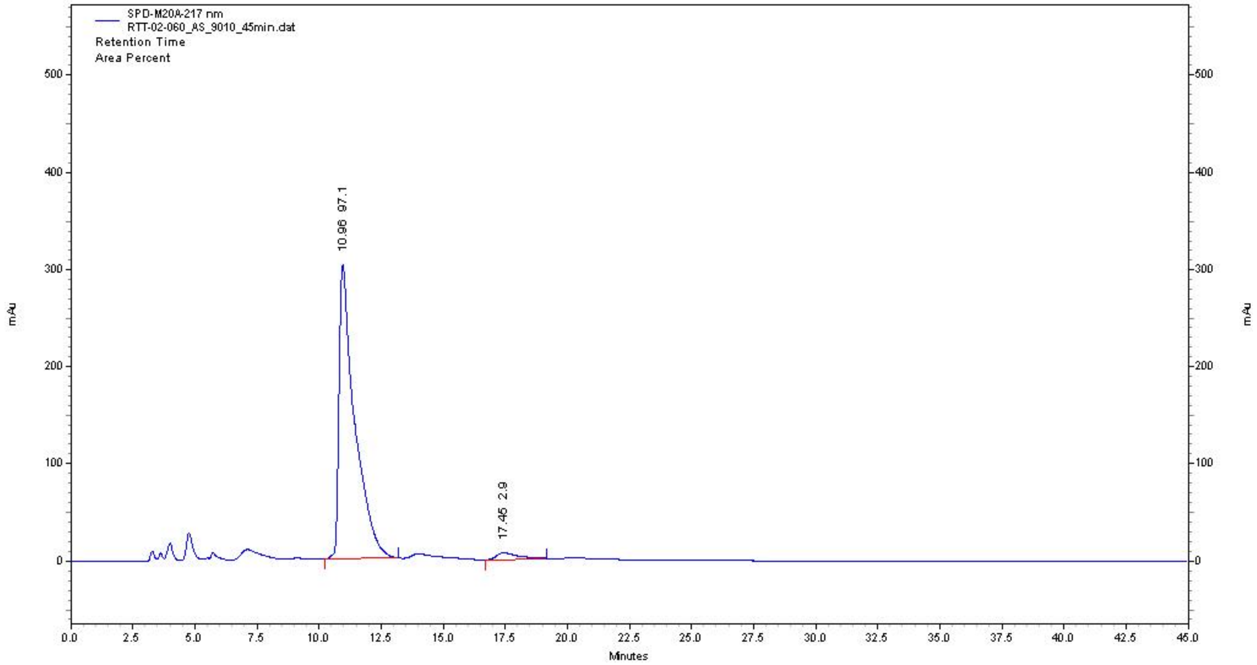

HPLC Analysis of **2c**

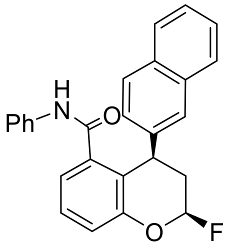

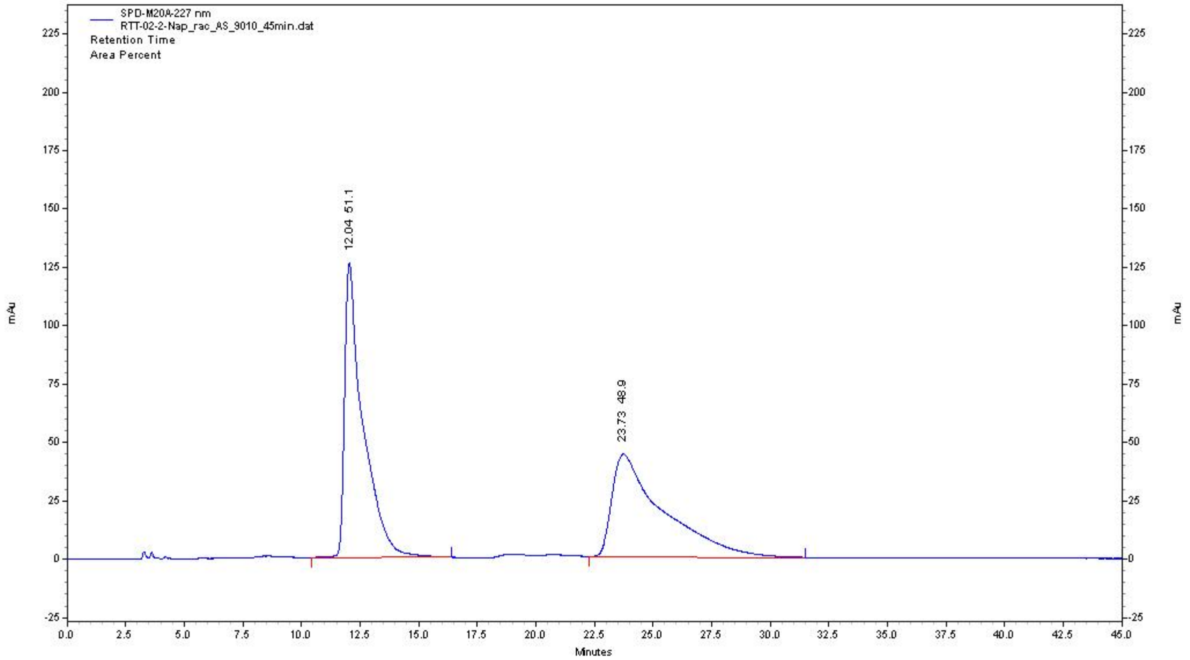

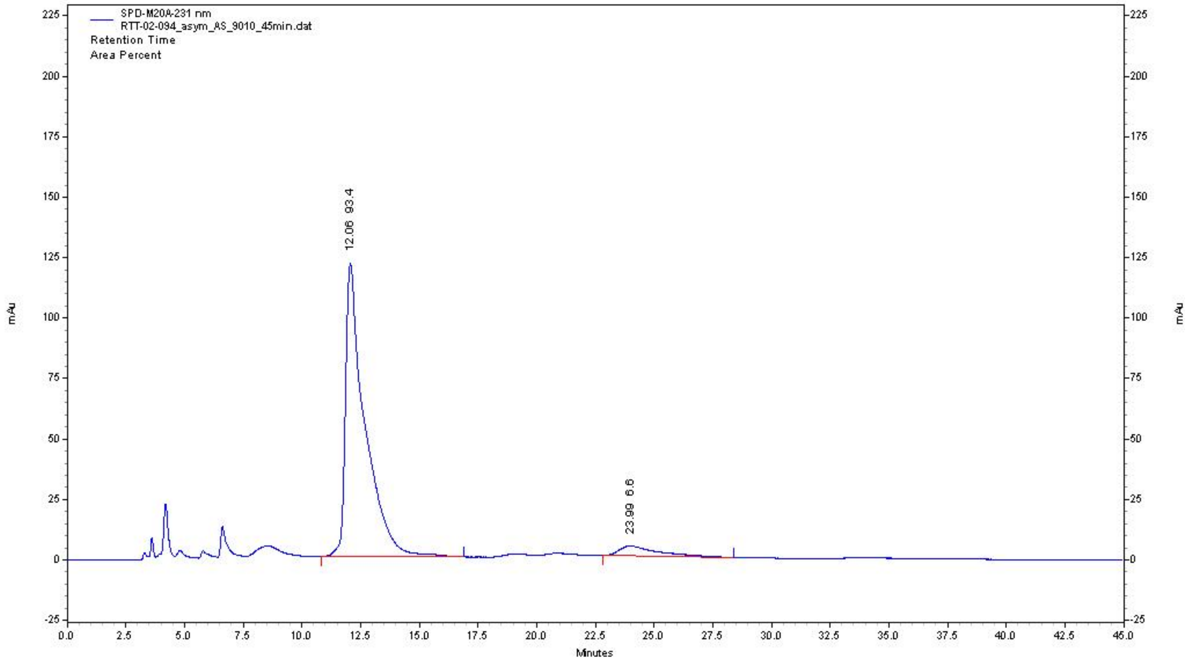

HPLC Analysis of **2d**

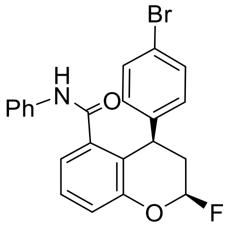

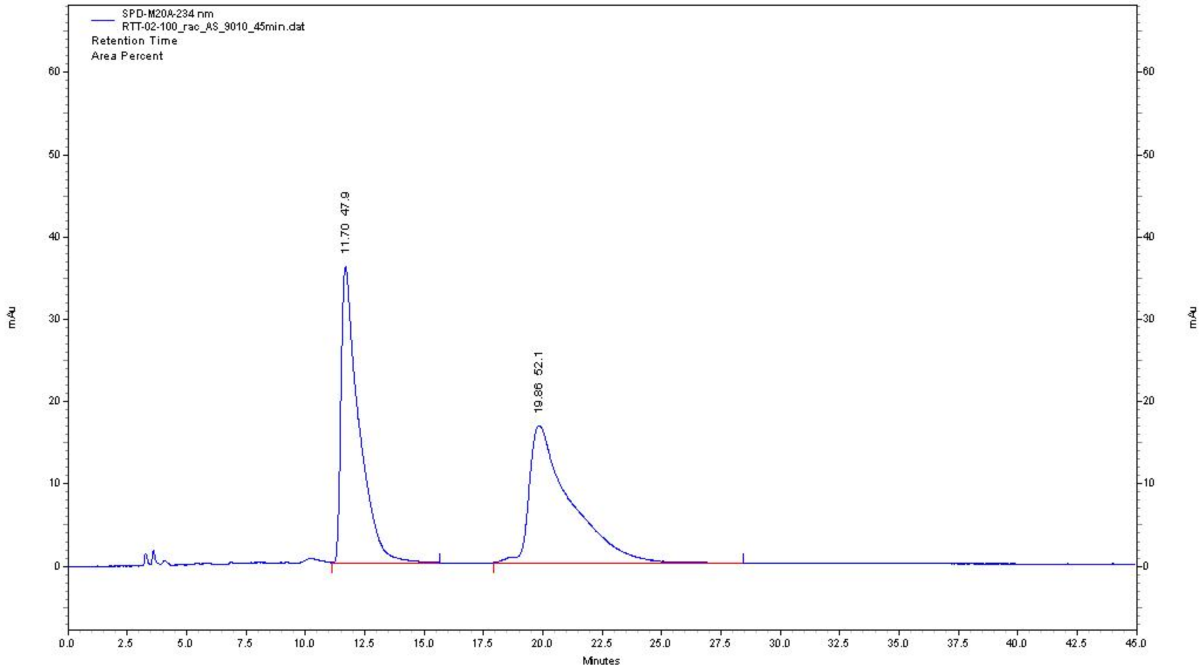

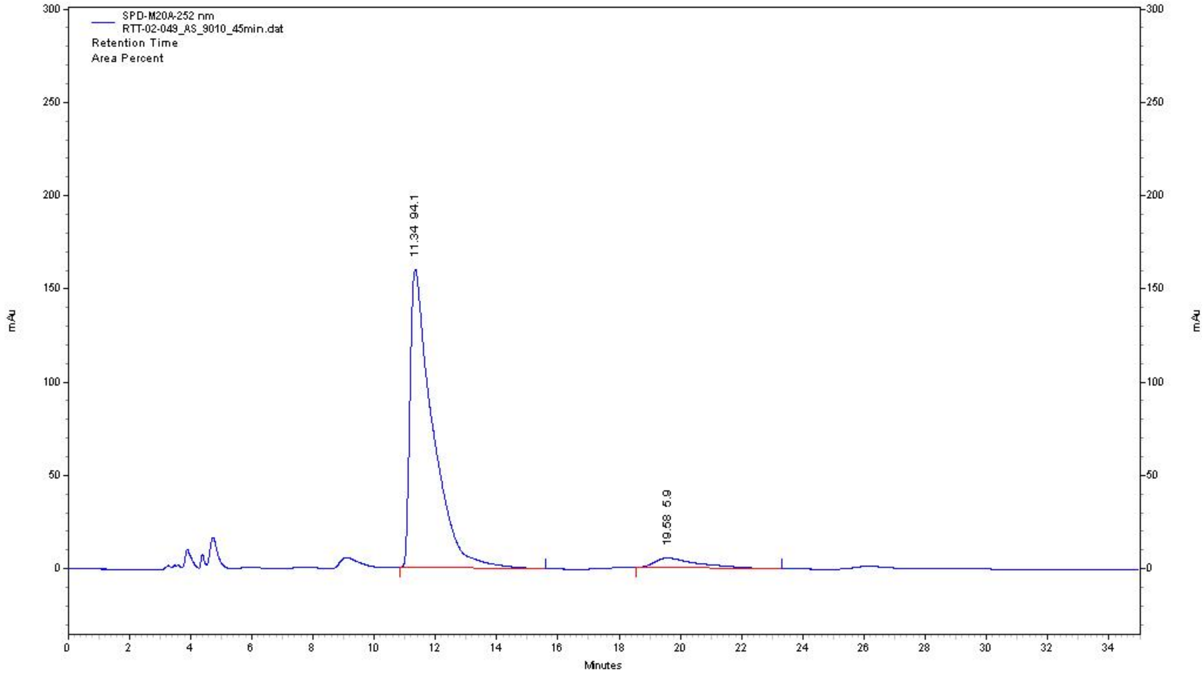

HPLC Analysis of **2e**

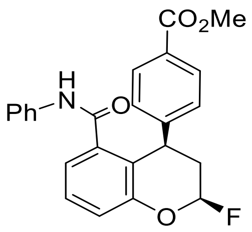

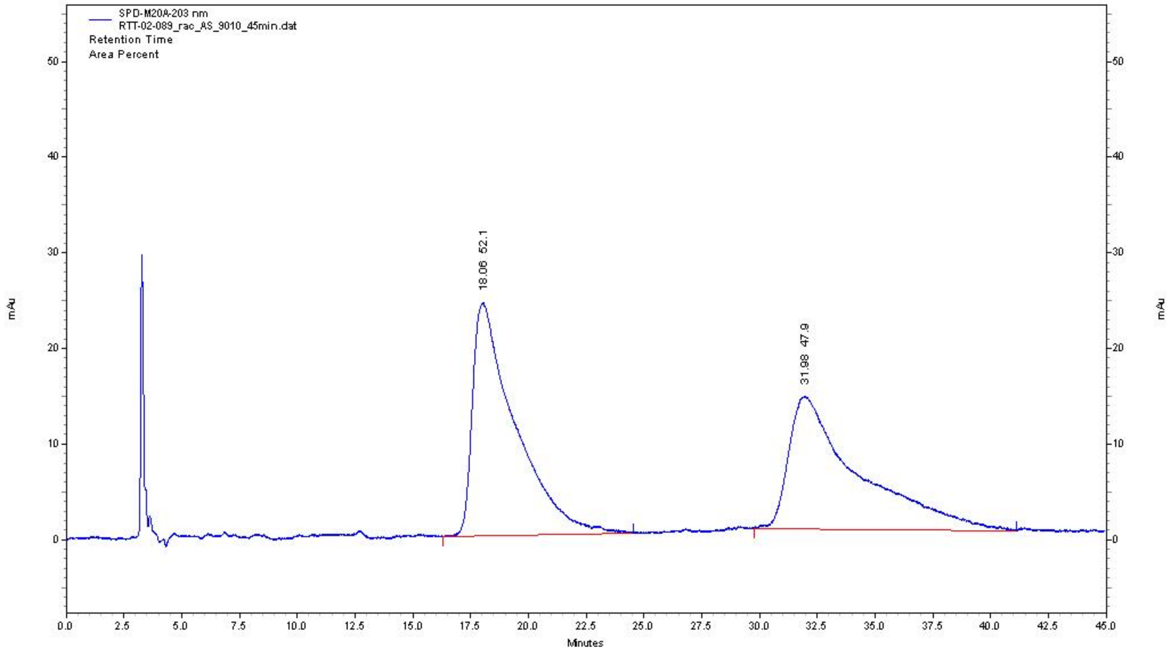

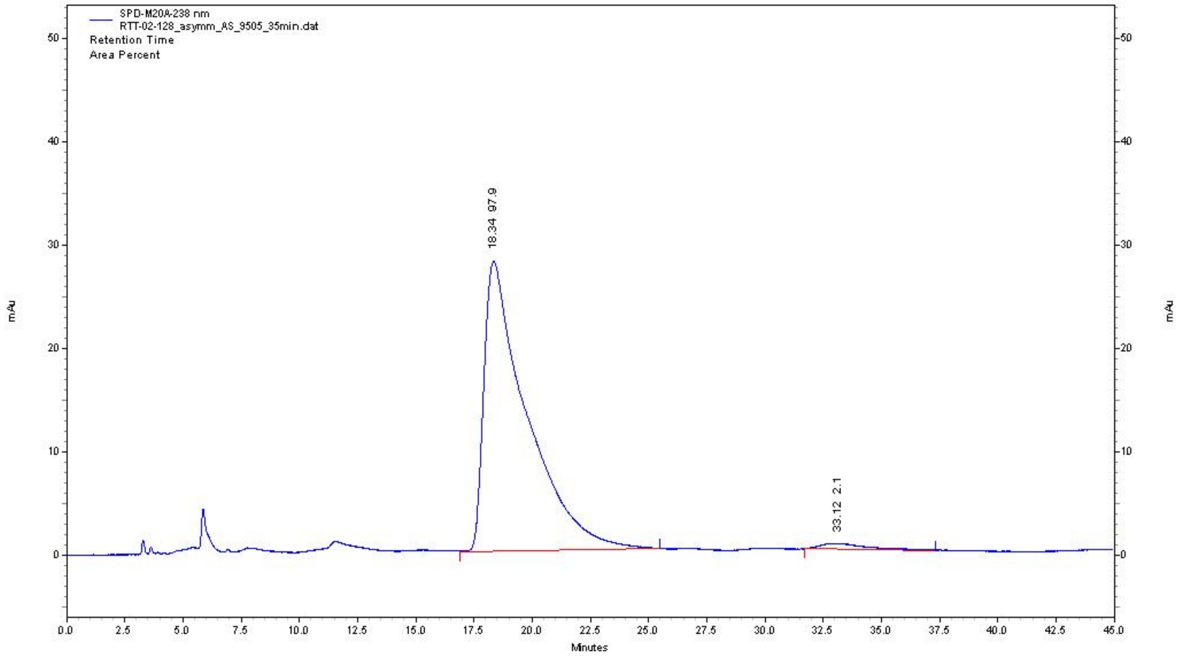

HPLC Analysis of **2f**

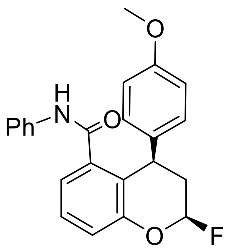

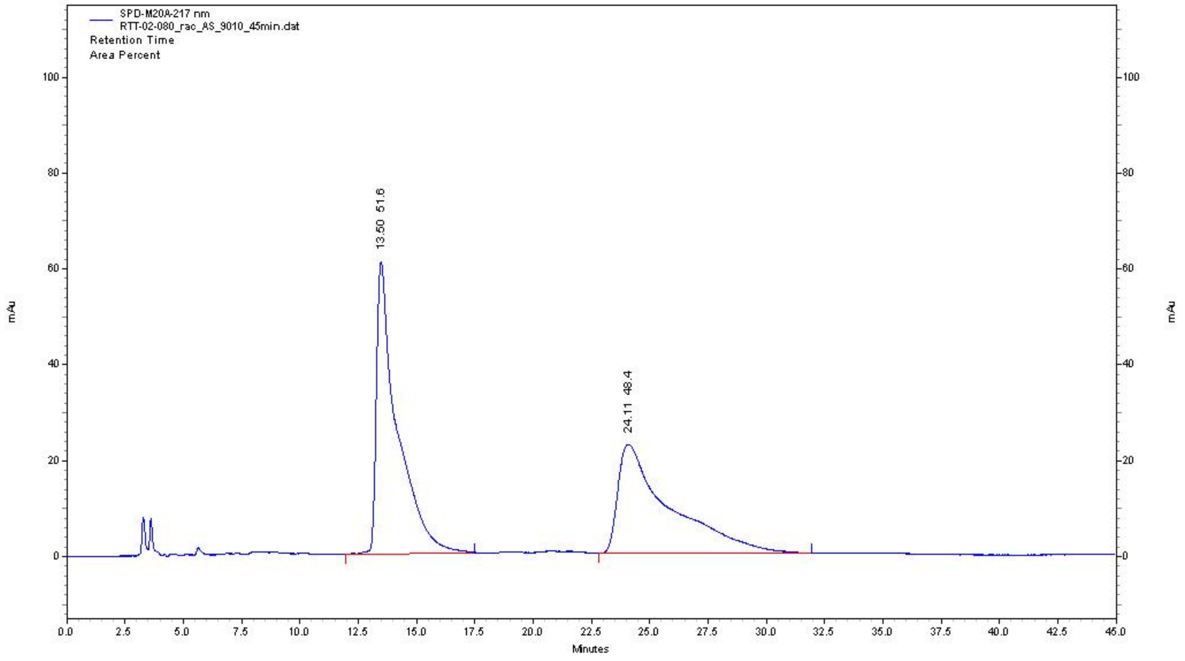

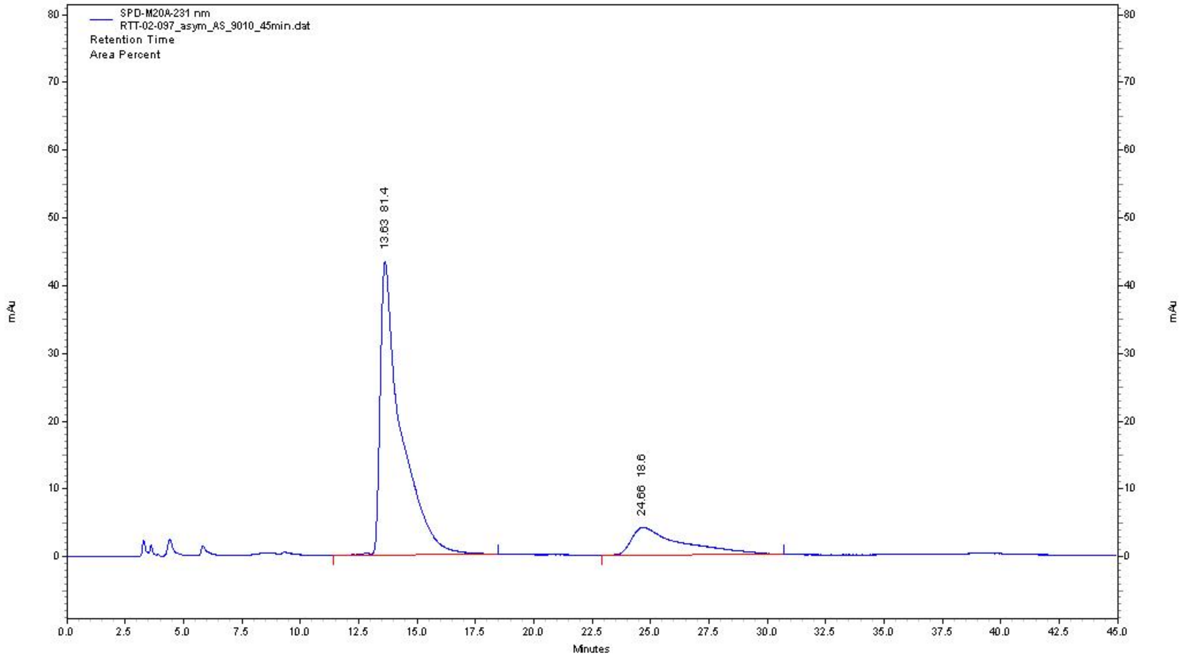

HPLC Analysis of **2g**

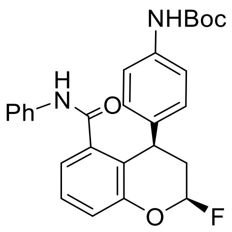

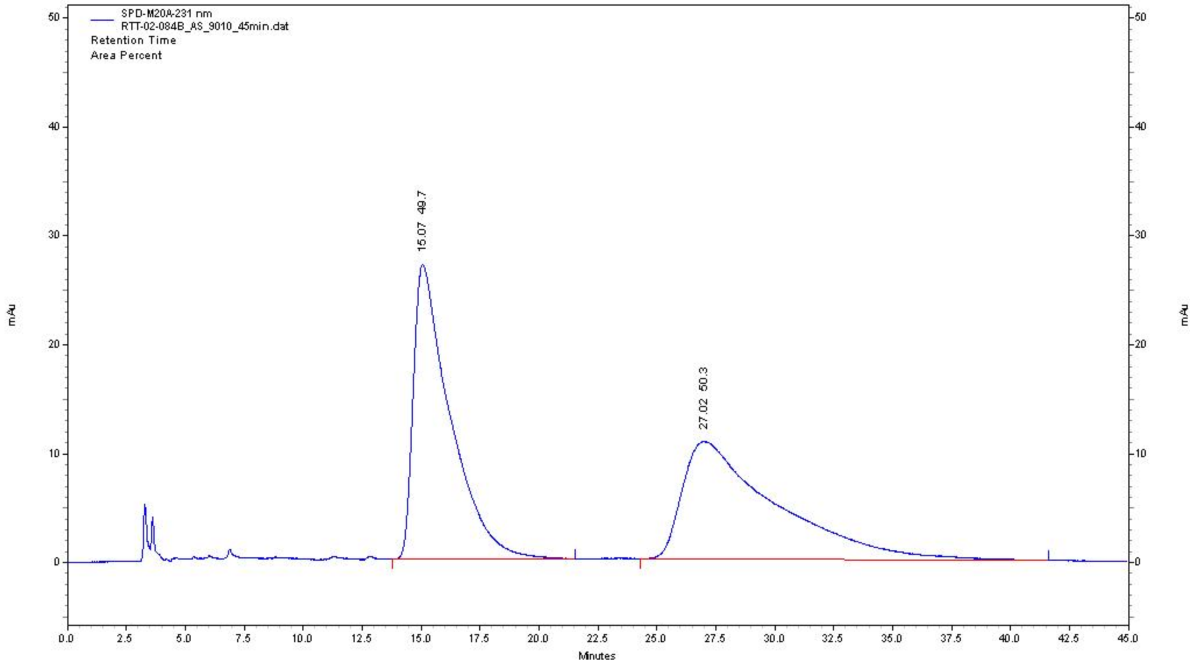

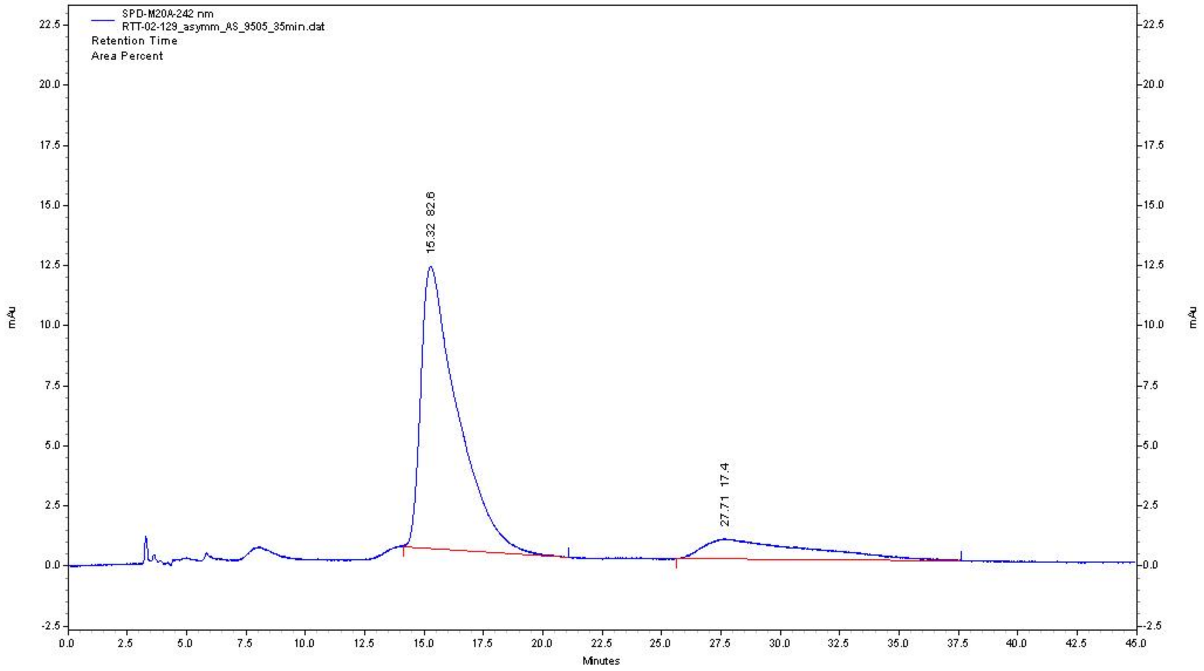

HPLC Analysis of **2h**

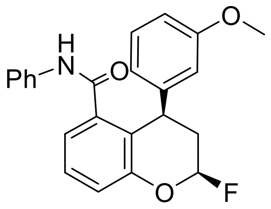

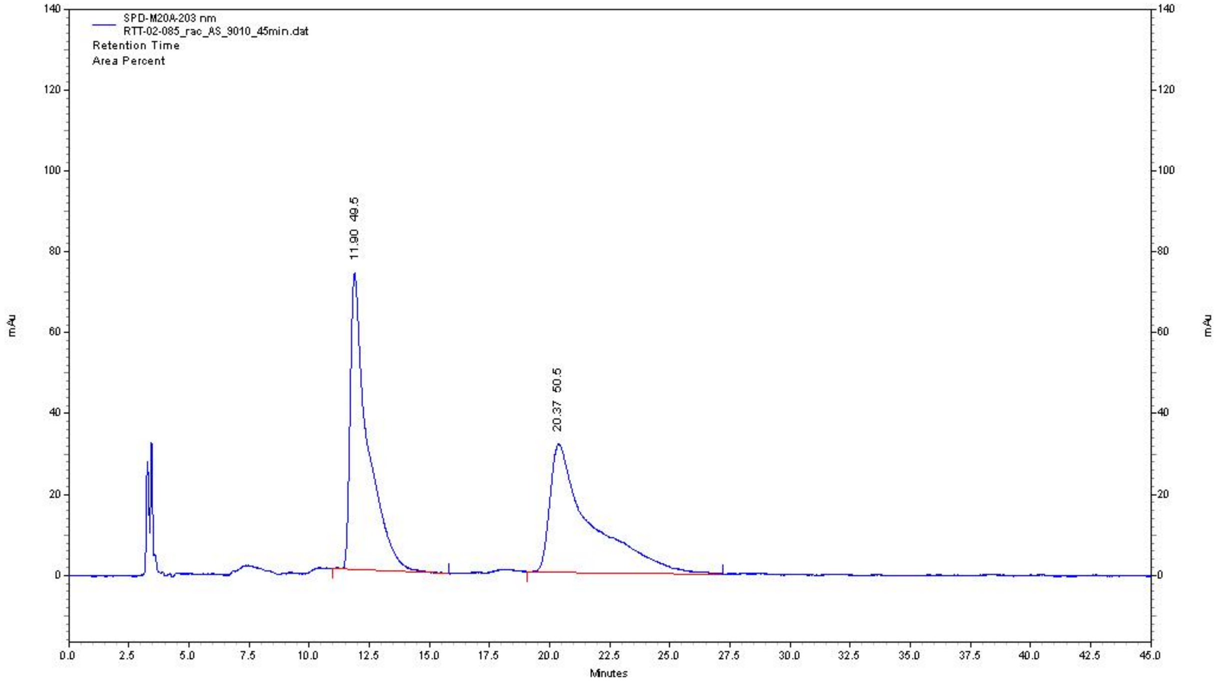

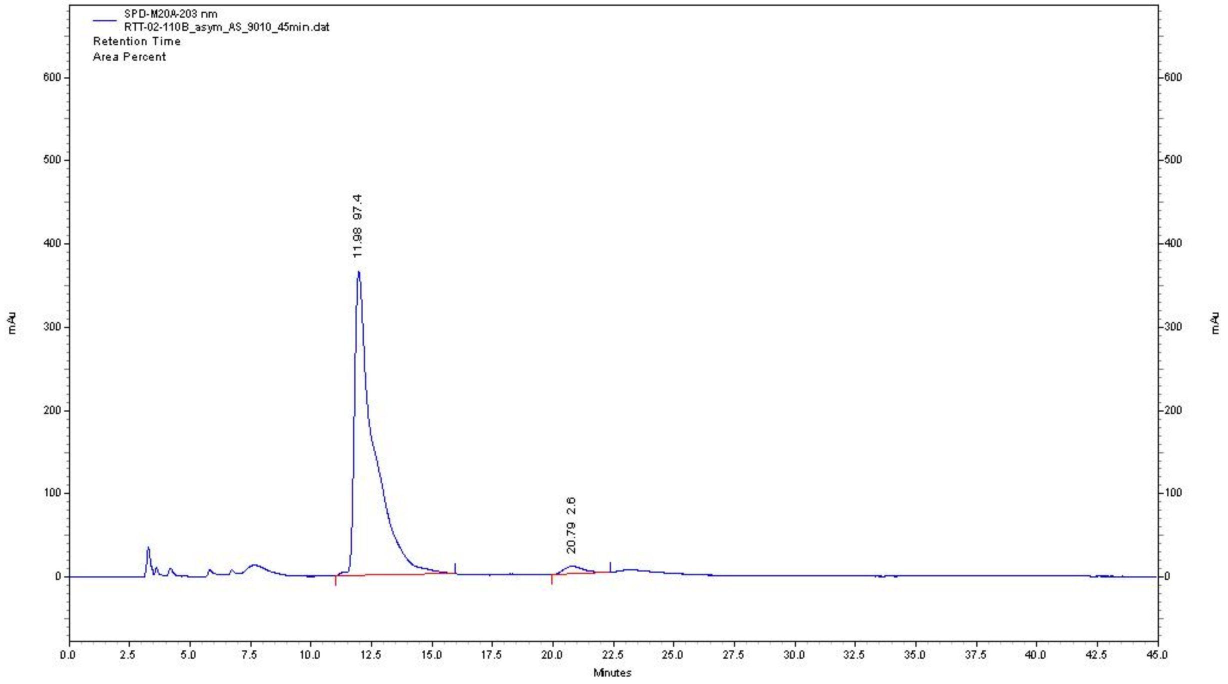

HPLC Analysis of **3**

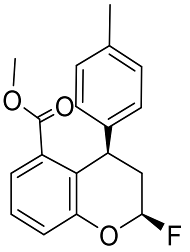

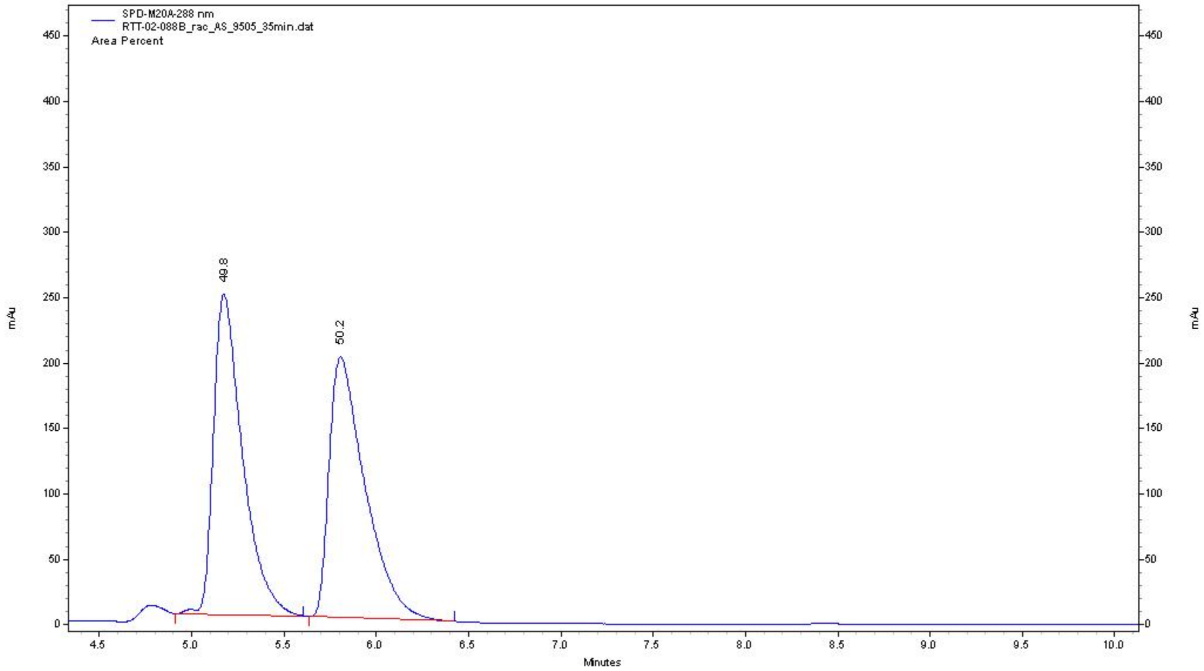

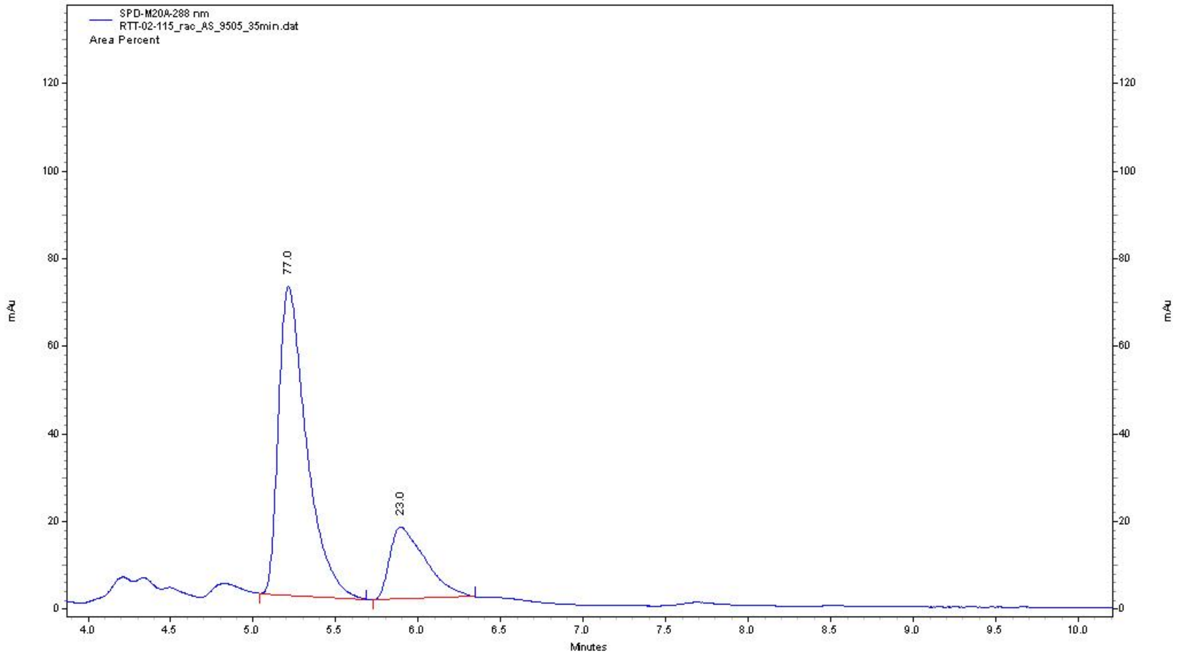

HPLC Analysis of **8**

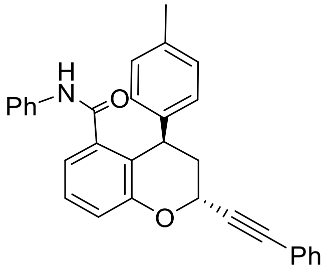

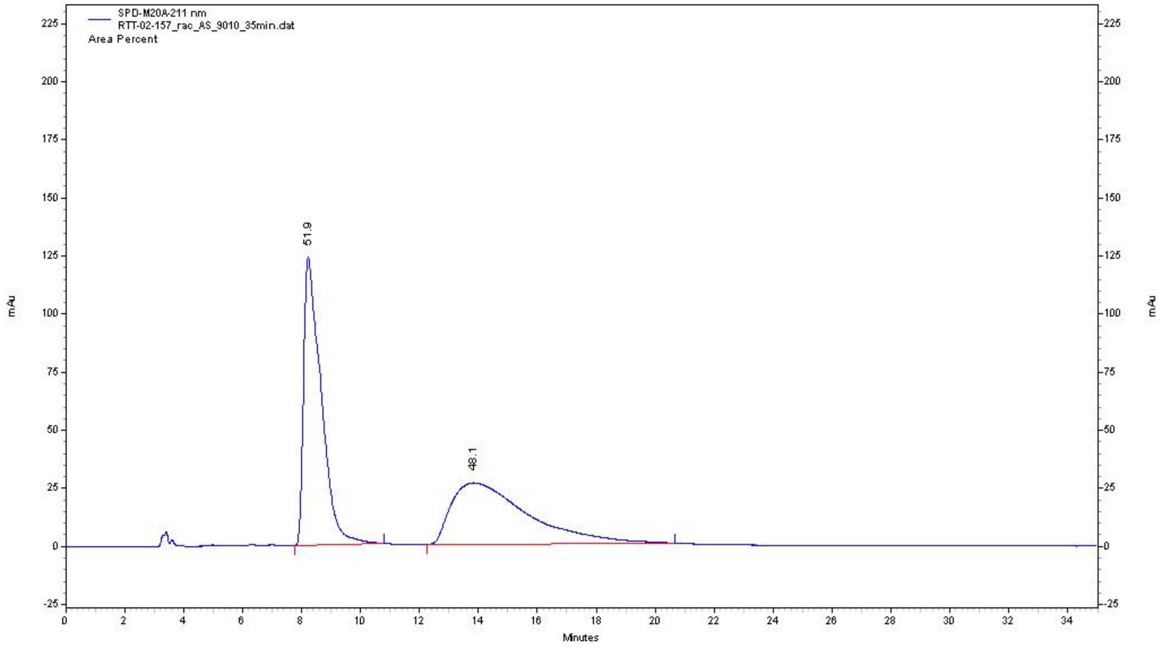

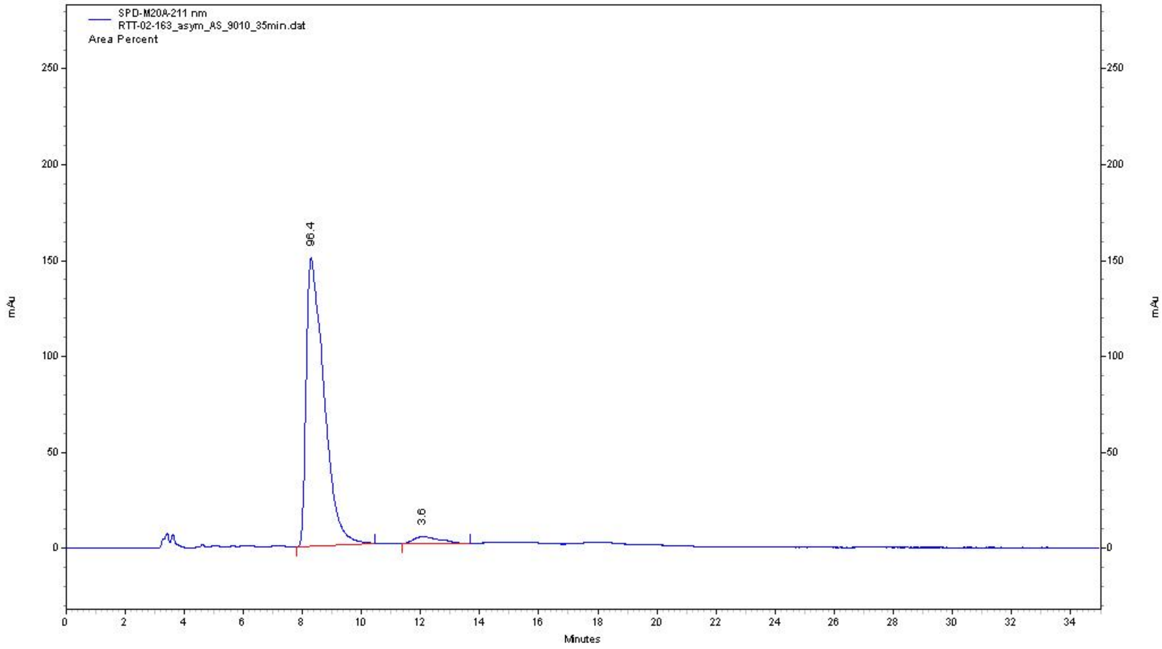

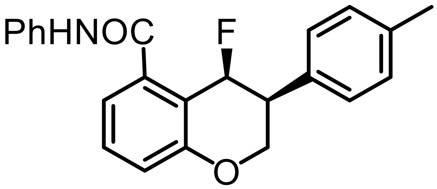
PhHNOC
F
O

Racemic

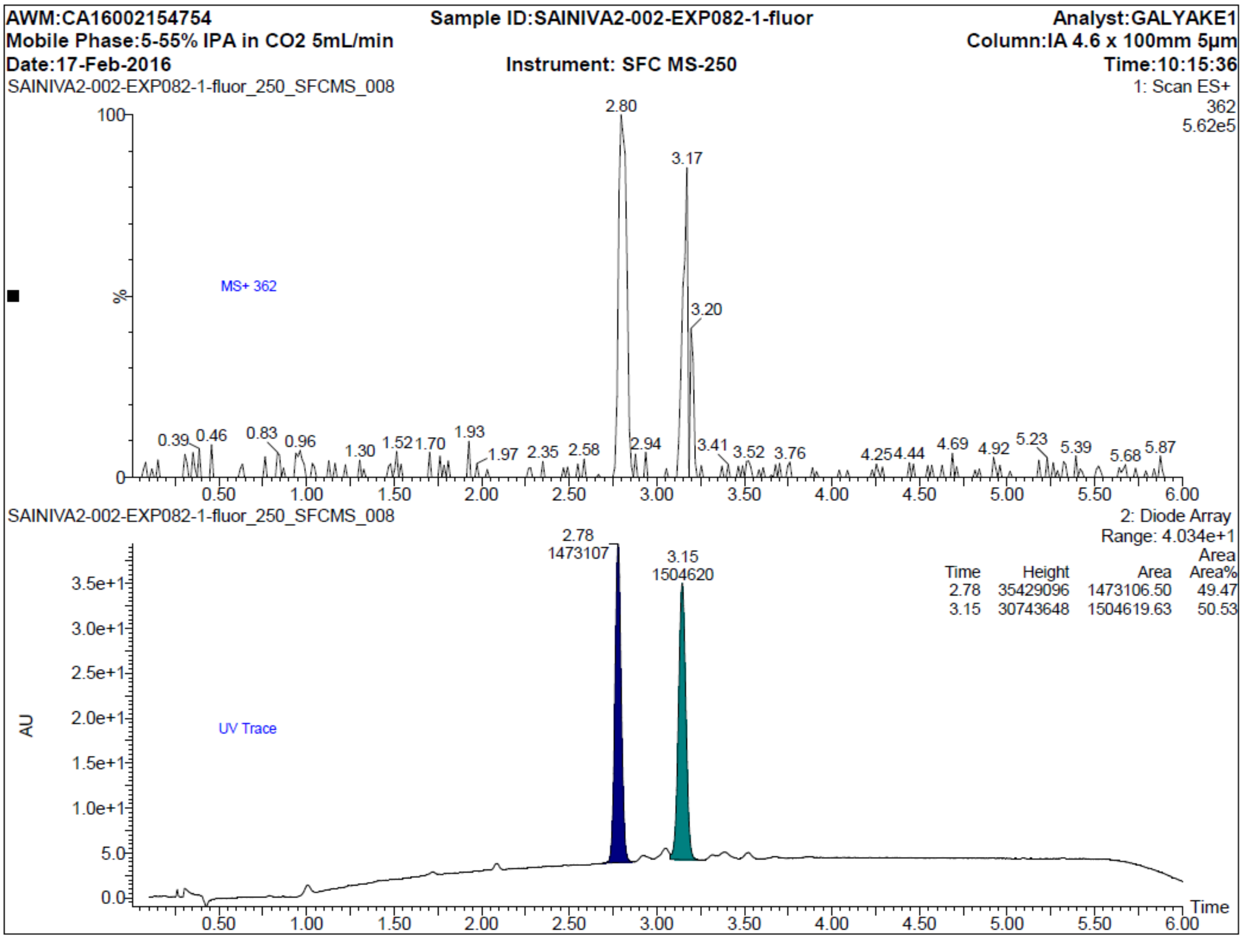
AWM:CA16002154754
Sample ID:SAINIVA2-002-EXP082-1-fluor
Analyst:GALYAKE1
Mobile Phase:5-55% IPA in CO2 5mL/min
Column:IA 4.6 x 100mm 5µm
Date:17-Feb-2016
Instrument: SFC MS-250
Time:10:15:36
SAINIVA2-002-EXP082-1-fluor_250_SFCMS_008
1: Scan ES+
362
5.62e5
MS+ 362
%
2.80
3.17
3.20
SAINIVA2-002-EXP082-1-fluor_250_SFCMS_008
2: Diode Array
Range: 4.034e+1
2.78
1473107
3.15
1504620
Time Height Area Area%
2.78 35429096 1473106.50 49.47
3.15 30743648 1504619.63 50.53
UV Trace
AU
Time

Chiral

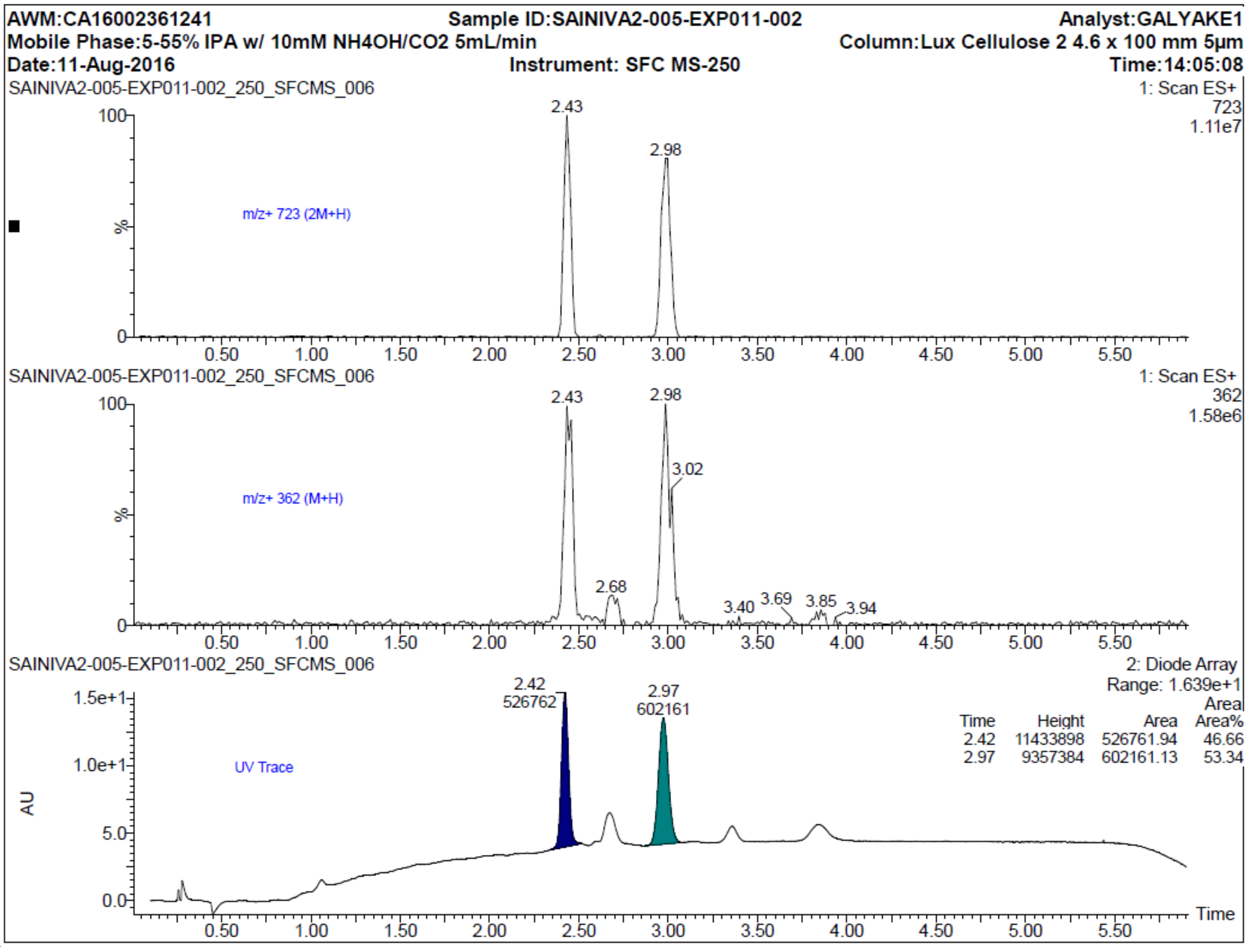
AWM:CA16002361241
Sample ID:SAINIVA2-005-EXP011-002
Analyst:GALYAKE1
Mobile Phase:5-55% IPA w/ 10mM NH4OH/CO2 5mL/min
Column:Lux Cellulose 2 4.6 x 100 mm 5µm
Date:11-Aug-2016
Instrument: SFC MS-250
Time:14:05:08
SAINIVA2-005-EXP011-002_250_SFCMS_006
1: Scan ES+
723
1.11e7
m/z+ 723 (2M+H)
2.43
2.98
SAINIVA2-005-EXP011-002_250_SFCMS_006
1: Scan ES+
362
1.58e6
m/z+ 362 (M+H)
2.43
2.98
3.02
2.68
3.40
3.69
3.85
3.94
SAINIVA2-005-EXP011-002_250_SFCMS_006
2: Diode Array
Range: 1.639e+1
UV Trace
2.42
526762
2.97
602161
Time Height Area Area%
2.42 11433898 526761.94 46.66
2.97 9357384 602161.13 53.34
AU
Time

9. X-ray crystallographic data for rac-2d :

X-ray crystallography quality crystals of **rac-2d** were grown by solvent layering from $CHCl_3$ and *n*-pentane. A colorless needle 0.01 x 0.02 x 0.06 mm in size was mounted on a Cryoloop with Paratone oil. Data were collected in a nitrogen gas stream at 100(2) K using phi and omega scans. Crystal-to-detector distance was 60 mm and exposure time was 5 seconds per frame using a scan width of 1.0°. Data collection was 99.7% complete to 67.00° in q. A total of 24648 reflections were collected covering the indices, -18<=h<=18, -14<=k<=14, -11<=l<=12. 3399 reflections were found to be symmetry independent, with an Rint of 0.0469. Indexing and unit cell refinement indicated a primitive, monoclinic lattice. The space group was found to be P2(1)/c (No. 14). The data were integrated using the Bruker SAINT software program and scaled using the SADABS software program. Solution by direct methods (SIR-2011) produced a complete heavy-atom phasing model consistent with the proposed structure. All non-hydrogen atoms were refined anisotropically by full-matrix least-squares (SHELXL-2014). All hydrogen atoms were placed using a riding model. Their positions were constrained relative to their parent atom using the appropriate HFIX command in SHELXL-2014.

Crystal data and structure refinement for ***rac*-2d**.

Identification code	shelx	
Empirical formula	C22 H17 Br F N O2	
Formula weight	426.27	
Temperature	100(2) K	
Wavelength	1.54178 Å	
Crystal system	Monoclinic	
Space group	P 21/c	
Unit cell dimensions	a = 15.3026(6) Å	α= 90°.
	b = 12.0184(4) Å	β= 90.552(3)°.
	c = 10.1214(4) Å	γ = 90°.
Volume	1861.37(12) Å^3	
Z	4	
Density (calculated)	1.521 Mg/m^3	
Absorption coefficient	3.238 mm^{-1}	
F(000)	864	
Crystal size	0.060 x 0.020 x 0.010 mm^3	
Theta range for data collection	2.888 to 68.325°.	
Index ranges	-18<=h<=18, -14<=k<=14, -11<=l<=12	
Reflections collected	24648	
Independent reflections	3399 [R(int) = 0.0469]	
Completeness to theta = 67.000°	99.7 %	
Absorption correction	Semi-empirical from equivalents	
Max. and min. transmission	0.7531 and 0.6139	
Refinement method	Full-matrix least-squares on F^2	
Data / restraints / parameters	3399 / 0 / 244	
Goodness-of-fit on F^2	1.038	
Final R indices [I>2sigma(I)]	R1 = 0.0372, wR2 = 0.0915	
R indices (all data)	R1 = 0.0447, wR2 = 0.0963	
Extinction coefficient	n/a	
Largest diff. peak and hole	0.870 and -0.325 e.Å^{-3}	

Table 7. Atomic coordinates (x 10^4) and equivalent isotropic displacement parameters ($Å^2$x 10^3) for ***rac*-2d**. U(eq) is defined as one third of the trace of the orthogonalized U^{ij} tensor.

	x	y	z	U(eq)
C(1)	1366(2)	-2975(2)	9430(3)	21(1)
C(2)	2218(2)	-3351(2)	9158(3)	23(1)
C(3)	2366(2)	-4394(2)	8630(3)	25(1)
C(4)	1664(2)	-5106(2)	8371(3)	26(1)
C(5)	823(2)	-4749(2)	8586(3)	25(1)
C(6)	680(2)	-3686(2)	9094(3)	23(1)
C(7)	-386(2)	-2307(2)	9520(3)	27(1)
C(8)	233(2)	-1759(2)	10480(3)	26(1)
C(9)	1195(2)	-1850(2)	10055(3)	22(1)
C(10)	2993(2)	-2631(2)	9515(3)	23(1)
C(11)	4292(2)	-1739(2)	8571(3)	28(1)
C(12)	4440(2)	-950(3)	7600(3)	34(1)
C(13)	5193(2)	-298(3)	7664(4)	42(1)
C(14)	5783(2)	-435(3)	8692(4)	42(1)
C(15)	5629(2)	-1215(3)	9650(4)	40(1)
C(16)	4888(2)	-1887(3)	9598(3)	33(1)
C(17)	1513(2)	-890(2)	9198(3)	21(1)
C(18)	1516(2)	-926(2)	7825(3)	26(1)
C(19)	1861(2)	-63(2)	7087(3)	28(1)
C(20)	2215(2)	845(2)	7741(3)	24(1)
C(21)	2200(2)	921(2)	9092(3)	24(1)
C(22)	1849(2)	54(2)	9813(3)	24(1)
N(1)	3519(2)	-2401(2)	8484(2)	26(1)
O(1)	-190(1)	-3413(2)	9261(2)	27(1)
O(2)	3128(1)	-2322(2)	10653(2)	27(1)
F(1)	-365(1)	-1730(1)	8314(2)	31(1)
Br(1)	2791(1)	1982(1)	6774(1)	33(1)

Table 8. Bond lengths [Å] and angles [°] for ***rac*-2d**.

C(1)-C(6)	1.393(4)
C(1)-C(2)	1.410(4)
C(1)-C(9)	1.516(4)
C(2)-C(3)	1.383(4)
C(2)-C(10)	1.509(4)
C(3)-C(4)	1.396(4)
C(3)-H(3)	0.9500
C(4)-C(5)	1.376(4)
C(4)-H(4)	0.9500
C(5)-C(6)	1.394(4)
C(5)-H(5)	0.9500
C(6)-O(1)	1.383(3)
C(7)-O(1)	1.389(3)
C(7)-F(1)	1.405(3)
C(7)-C(8)	1.502(4)
C(7)-H(7)	1.0000
C(8)-C(9)	1.542(4)
C(8)-H(8A)	0.9900
C(8)-H(8B)	0.9900
C(9)-C(17)	1.526(4)
C(9)-H(9)	1.0000
C(10)-O(2)	1.226(3)
C(10)-N(1)	1.353(4)
C(11)-C(12)	1.385(4)
C(11)-C(16)	1.389(4)
C(11)-N(1)	1.428(4)
C(12)-C(13)	1.395(4)
C(12)-H(12)	0.9500
C(13)-C(14)	1.381(5)
C(13)-H(13)	0.9500
C(14)-C(15)	1.371(5)
C(14)-H(14)	0.9500
C(15)-C(16)	1.392(4)
C(15)-H(15)	0.9500

C(16)-H(16)	0.9500
C(17)-C(18)	1.390(4)
C(17)-C(22)	1.391(4)
C(18)-C(19)	1.386(4)
C(18)-H(18)	0.9500
C(19)-C(20)	1.384(4)
C(19)-H(19)	0.9500
C(20)-C(21)	1.371(4)
C(20)-Br(1)	1.902(3)
C(21)-C(22)	1.383(4)
C(21)-H(21)	0.9500
C(22)-H(22)	0.9500
N(1)-H(1)	0.8800

C(6)-C(1)-C(2)	116.8(2)
C(6)-C(1)-C(9)	121.2(2)
C(2)-C(1)-C(9)	122.1(2)
C(3)-C(2)-C(1)	121.5(2)
C(3)-C(2)-C(10)	118.7(2)
C(1)-C(2)-C(10)	119.7(2)
C(2)-C(3)-C(4)	119.9(3)
C(2)-C(3)-H(3)	120.0
C(4)-C(3)-H(3)	120.0
C(5)-C(4)-C(3)	119.9(3)
C(5)-C(4)-H(4)	120.0
C(3)-C(4)-H(4)	120.0
C(4)-C(5)-C(6)	119.6(3)
C(4)-C(5)-H(5)	120.2
C(6)-C(5)-H(5)	120.2
O(1)-C(6)-C(1)	123.2(2)
O(1)-C(6)-C(5)	114.7(2)
C(1)-C(6)-C(5)	122.1(2)
O(1)-C(7)-F(1)	107.5(2)
O(1)-C(7)-C(8)	113.9(2)
F(1)-C(7)-C(8)	109.1(2)
O(1)-C(7)-H(7)	108.8

F(1)-C(7)-H(7)	108.8
C(8)-C(7)-H(7)	108.8
C(7)-C(8)-C(9)	112.8(2)
C(7)-C(8)-H(8A)	109.0
C(9)-C(8)-H(8A)	109.0
C(7)-C(8)-H(8B)	109.0
C(9)-C(8)-H(8B)	109.0
H(8A)-C(8)-H(8B)	107.8
C(1)-C(9)-C(17)	112.3(2)
C(1)-C(9)-C(8)	110.4(2)
C(17)-C(9)-C(8)	114.6(2)
C(1)-C(9)-H(9)	106.3
C(17)-C(9)-H(9)	106.3
C(8)-C(9)-H(9)	106.3
O(2)-C(10)-N(1)	124.5(3)
O(2)-C(10)-C(2)	121.6(2)
N(1)-C(10)-C(2)	113.9(2)
C(12)-C(11)-C(16)	120.5(3)
C(12)-C(11)-N(1)	118.6(3)
C(16)-C(11)-N(1)	120.9(3)
C(11)-C(12)-C(13)	119.5(3)
C(11)-C(12)-H(12)	120.2
C(13)-C(12)-H(12)	120.2
C(14)-C(13)-C(12)	120.1(3)
C(14)-C(13)-H(13)	119.9
C(12)-C(13)-H(13)	119.9
C(15)-C(14)-C(13)	119.9(3)
C(15)-C(14)-H(14)	120.1
C(13)-C(14)-H(14)	120.1
C(14)-C(15)-C(16)	121.1(3)
C(14)-C(15)-H(15)	119.5
C(16)-C(15)-H(15)	119.5
C(11)-C(16)-C(15)	118.9(3)
C(11)-C(16)-H(16)	120.6
C(15)-C(16)-H(16)	120.6
C(18)-C(17)-C(22)	117.9(2)

C(18)-C(17)-C(9)	123.3(2)
C(22)-C(17)-C(9)	118.8(2)
C(19)-C(18)-C(17)	121.4(3)
C(19)-C(18)-H(18)	119.3
C(17)-C(18)-H(18)	119.3
C(20)-C(19)-C(18)	118.8(3)
C(20)-C(19)-H(19)	120.6
C(18)-C(19)-H(19)	120.6
C(21)-C(20)-C(19)	121.3(3)
C(21)-C(20)-Br(1)	118.5(2)
C(19)-C(20)-Br(1)	120.1(2)
C(20)-C(21)-C(22)	119.1(2)
C(20)-C(21)-H(21)	120.5
C(22)-C(21)-H(21)	120.5
C(21)-C(22)-C(17)	121.5(3)
C(21)-C(22)-H(22)	119.3
C(17)-C(22)-H(22)	119.3
C(10)-N(1)-C(11)	124.4(2)
C(10)-N(1)-H(1)	117.8
C(11)-N(1)-H(1)	117.8
C(6)-O(1)-C(7)	117.4(2)

Symmetry transformations used to generate equivalent atoms:

Table 9. Anisotropic displacement parameters ($Å^2 x 10^3$) for ***rac*-2d**. The anisotropic displacement factor exponent takes the form: $-2\pi^2[h^2 a^{*2}U^{11} + ... + 2 h k a^* b^* U^{12}]$

	U^{11}	U^{22}	U^{33}	U^{23}	U^{13}	U^{12}
C(1)	29(1)	20(1)	14(1)	4(1)	1(1)	1(1)
C(2)	28(1)	24(1)	16(1)	4(1)	2(1)	0(1)
C(3)	29(1)	25(1)	20(1)	2(1)	3(1)	4(1)
C(4)	39(2)	20(1)	20(1)	0(1)	2(1)	2(1)
C(5)	34(1)	22(1)	19(1)	3(1)	0(1)	-4(1)
C(6)	28(1)	23(1)	17(1)	5(1)	0(1)	0(1)
C(7)	27(1)	27(1)	28(2)	5(1)	4(1)	3(1)
C(8)	29(1)	24(1)	24(1)	2(1)	5(1)	2(1)
C(9)	27(1)	21(1)	19(1)	2(1)	1(1)	0(1)
C(10)	25(1)	22(1)	21(2)	1(1)	1(1)	3(1)
C(11)	26(1)	31(1)	28(2)	-8(1)	6(1)	-3(1)
C(12)	37(2)	38(2)	28(2)	-2(1)	5(1)	-6(1)
C(13)	44(2)	40(2)	42(2)	-6(2)	16(2)	-13(1)
C(14)	31(2)	48(2)	48(2)	-17(2)	10(1)	-11(1)
C(15)	24(1)	53(2)	43(2)	-16(2)	3(1)	-1(1)
C(16)	30(1)	38(2)	30(2)	-4(1)	3(1)	1(1)
C(17)	22(1)	19(1)	21(1)	0(1)	0(1)	2(1)
C(18)	36(2)	19(1)	22(1)	-1(1)	-1(1)	-2(1)
C(19)	42(2)	24(1)	18(1)	1(1)	1(1)	-2(1)
C(20)	26(1)	21(1)	25(1)	3(1)	2(1)	2(1)
C(21)	26(1)	22(1)	23(1)	-5(1)	1(1)	-1(1)
C(22)	29(1)	25(1)	19(1)	-4(1)	2(1)	-1(1)
N(1)	27(1)	30(1)	19(1)	-2(1)	2(1)	-4(1)
O(1)	26(1)	23(1)	31(1)	2(1)	1(1)	-2(1)
O(2)	28(1)	33(1)	20(1)	-2(1)	0(1)	-1(1)
F(1)	35(1)	31(1)	26(1)	7(1)	-2(1)	4(1)
Br(1)	42(1)	26(1)	32(1)	8(1)	4(1)	-6(1)

Table 10. Hydrogen coordinates (x 10^4) and isotropic displacement parameters ($Å^2$x 10^3) for ***rac*-2d**.

	x	y	z	U(eq)
H(3)	2946	-4627	8443	30
H(4)	1767	-5836	8048	31
H(5)	343	-5223	8389	30
H(7)	-991	-2264	9881	33
H(8A)	167	-2109	11359	31
H(8B)	76	-964	10564	31
H(9)	1554	-1827	10886	27
H(12)	4032	-854	6896	41
H(13)	5301	241	6998	51
H(14)	6295	12	8737	51
H(15)	6034	-1300	10360	48
H(16)	4793	-2437	10255	39
H(18)	1276	-1555	7384	31
H(19)	1856	-95	6149	34
H(21)	2428	1560	9528	29
H(22)	1838	105	10749	29
H(1)	3375	-2677	7707	31

Table 11. Hydrogen bonds for ***rac*-2d** [Å and °].

D-H...A	d(D-H)	d(H...A)	d(D...A)	<(DHA)

10. DFT of *cis*- and *trans*- 1,3-arylfluorination Products

Density functional theory (DFT) calculations were performed with Gaussian 09 revision D01. Geometry optimizations were carried out at the B3LYP level of theory with the 6-31G(d) basis set. Optimized geometries were verified as a minima by frequency computations (zero imaginary frequencies). Single-point energy calculations on the optimized geometries were then evaluated using different density functionals and the triple-zeta valence quality def2-TZVPP basis set, within the SMD/IEF-PCM model (dichloromethane). The thermal corrections evaluated from the unscaled vibrational frequencies at the B3LYP/6-31G(d) level on the optimized geometries were then added to these electronic energies to obtain the free energies.

Level of theory	ΔG_{trans} (H)	ΔG_{cis} (H)	ΔΔG (kcal/mol)
B3LYP-D3/Def2TZVPP//B3LYP/6-31G(d)	-1193,784844	-1193,785210	0.2
M062X/Def2TZVPP//B3LYP/6-31G(d)	-1193,255521	-1193,256118	0.4
wB97XD/Def2TZVPP//B3LYP/6-31G(d)	-1193,338696	-1193,338928	0.1

Cartesian coordinates

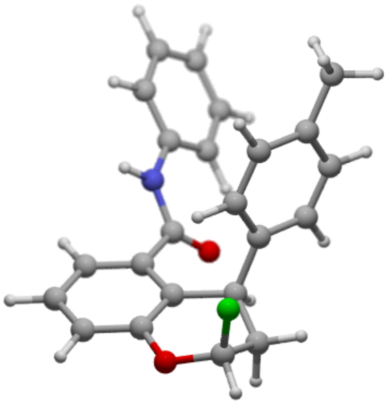

cis

C	2.063478000	-3.498878000	9.462858000
C	3.383647000	-3.921528000	9.188372000
C	3.627527000	-5.138860000	8.543560000
C	2.564865000	-5.962666000	8.165411000
C	1.260913000	-5.566418000	8.424425000
C	1.020531000	-4.344933000	9.063853000
C	-0.671707000	-2.772870000	9.674276000
C	0.303289000	-2.160004000	10.658846000
C	1.767114000	-2.174542000	10.154978000
C	4.563655000	-3.107398000	9.665544000
C	6.766021000	-2.250800000	8.793077000
C	7.517474000	-2.128112000	7.613282000
C	8.755738000	-1.493016000	7.628204000
C	9.264367000	-0.971008000	8.819092000
C	8.515427000	-1.094025000	9.989920000
C	7.272332000	-1.727235000	9.992764000
C	2.143215000	-0.950619000	9.314909000
C	1.925407000	-0.872766000	7.933760000
C	2.271420000	0.273260000	7.219439000
C	2.849776000	1.380849000	7.852350000
C	3.068179000	1.300325000	9.232746000
C	2.727642000	0.153360000	9.948594000
N	5.525946000	-2.918544000	8.699594000
O	-0.312647000	-4.070431000	9.295043000
O	4.654899000	-2.692870000	10.816394000
F	-0.759618000	-1.980774000	8.535201000
C	3.253307000	2.607289000	7.067239000
H	4.650571000	-5.461139000	8.371595000
H	2.757251000	-6.912937000	7.675755000
H	0.409424000	-6.178700000	8.145350000
H	-1.683984000	-2.861643000	10.077877000
H	0.232785000	-2.747454000	11.582811000
H	-0.013904000	-1.139076000	10.890364000
H	2.402673000	-2.136823000	11.043061000
H	7.126441000	-2.532621000	6.681470000
H	9.322568000	-1.407454000	6.704986000
H	10.230746000	-0.475227000	8.832793000
H	8.899407000	-0.691437000	10.923660000
H	6.690896000	-1.816573000	10.899136000
H	1.466154000	-1.705656000	7.411855000
H	2.084078000	0.308508000	6.148065000
H	3.521534000	2.140733000	9.754256000
H	2.928013000	0.111205000	11.016757000
H	5.272655000	-3.229550000	7.771171000
H	2.630678000	2.734583000	6.175120000
H	4.296347000	2.539965000	6.729311000
H	3.168156000	3.516858000	7.671781000

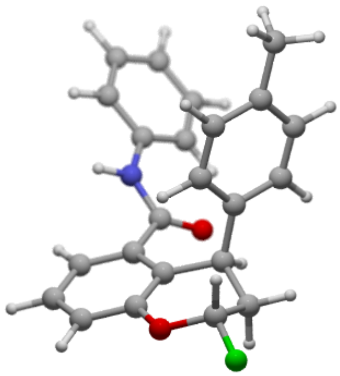

trans

C	2.147467000	-3.435812000	9.428133000
C	3.457745000	-3.906447000	9.189522000
C	3.670352000	-5.127200000	8.538224000
C	2.585702000	-5.898397000	8.113882000
C	1.291133000	-5.446375000	8.322280000
C	1.078532000	-4.221077000	8.968294000
C	-0.566919000	-2.605673000	9.666016000
F	-1.809000000	-2.772686000	10.228436000
C	0.427256000	-2.173687000	10.718509000
C	1.861852000	-2.126423000	10.147427000
C	4.654675000	-3.129700000	9.686473000
C	6.926267000	-2.405997000	8.870318000
C	7.725975000	-2.363540000	7.716784000
C	8.989005000	-1.780358000	7.754507000
C	9.474927000	-1.231138000	8.942631000
C	8.678377000	-1.274653000	10.087362000
C	7.409911000	-1.855090000	10.067172000
C	2.107456000	-0.886067000	9.286114000
C	1.850417000	-0.850608000	7.910182000
C	2.043601000	0.319333000	7.173028000
C	2.507979000	1.491414000	7.779422000
C	2.771414000	1.452366000	9.155719000
C	2.578684000	0.286731000	9.893088000
N	5.662294000	-3.023675000	8.753482000
O	-0.238542000	-3.862091000	9.103771000
O	4.722068000	-2.663113000	10.819190000
C	2.748537000	2.748899000	6.977097000
H	4.682699000	-5.495746000	8.399882000
H	2.754046000	-6.853043000	7.623738000
H	0.426028000	-6.016228000	7.998912000
H	-0.671856000	-1.874292000	8.855539000
H	0.374879000	-2.899829000	11.538591000
H	0.131023000	-1.196381000	11.111159000
H	2.545154000	-2.065779000	10.996527000
H	7.353053000	-2.789743000	6.787161000
H	9.592923000	-1.756816000	6.851379000
H	10.460554000	-0.775762000	8.974308000
H	9.044471000	-0.850434000	11.018682000
H	6.792511000	-1.882743000	10.953477000
H	1.505598000	-1.746364000	7.399753000
H	1.831873000	0.317197000	6.105819000
H	3.140704000	2.345479000	9.655530000
H	2.803103000	0.282440000	10.957369000
H	5.436375000	-3.370249000	7.830746000
H	2.209053000	2.725849000	6.024590000
H	3.814664000	2.877228000	6.747112000
H	2.428114000	3.642281000	7.524958000

11. DFT for Statistical Analysis

DFT Calculations

Compounds were geometrically optimized, with an ultrafine integration grid and ideal gas phase approximation using Gaussian 09 software.[9] DFT calculations of benzoic acid (as surrogates for arylboronic acid) ground state structures were performed using M06-2x functional and a triple zeta potential basis set (JUN-CC-PVTZ).[10] DFT calculations of $PdLCl_2$ complexes were performed using B3LYP functional and LANL2DZ basis set for the palladium atom and 6-31G(d,p) basis set for other atoms. NBO charges[11] and torsion angles were obtained from the geometry optimized structures. Hammett values were acquired from the compilation made by Hansch, Leo, and Taft.[12]

According to Curtin-Hammett principle,[13] the relative rate of formation of competing products (X and Y) is logarithmically related to the difference in transition state energies, represented by the measured $\Delta\Delta G^{\ddagger}$ (equation 1), where R is the gas constant and T is temperature. To derive measured $\Delta\Delta G^{\ddagger}$ values, product ratios resulting from differences in selectivity were obtained experimentally.

$$\text{measured } \Delta\Delta G^{\ddagger} = -RTln\left(\frac{X}{Y}\right) \qquad (1)$$

Table 12. Regioselectivity ratios (rr) of 1,3- vs 2,1-arylfluorinated product and corresponding measured $\Delta\Delta G^{\ddagger}$ from reactions run with various arylboronic acids.

R	*Trial 1 rr*	*Trial 2 rr*	*Average rr*	*Measured $\Delta\Delta G^{\ddagger}$*
p-Me	8.2	7.5	7.9	1.22
H	7.6	8.2	7.9	1.22
p-COMe	30.0	28.0	29.0	1.99
p-CO_2Me	17.7	20.8	19.3	1.75
p-OMe	15.2	13.4	14.3	1.58
p-F	10.8	11.7	11.3	1.43
p-CF_3	17.8	16.4	17.1	1.68
p-Br	14.0	12.0	13.0	1.52

m-OMe	16.2	16.5	16.4	1.66
m-CO_2Me	27.0	36.0	31.5	2.04
m-Cl	7.6	8.3	8.0	1.23

$\Delta\Delta G^{\ddagger}$ is reported in kcal/mol.

Table 13. Calculated parameters for benzoic acids as surrogates for arylboronic acids.

R	v_{COH}	$v_{C=O}$	NBO_C	$NBO_{=O}$	NBO_O	NBO_H
p-Me	1395.02	1844.66	0.78588	-0.60618	-0.70599	0.50033
H	1394.60	1847.85	0.80516	-0.60208	-0.70358	0.50087
p-COMe	1399.86	1851.01	0.80289	-0.59612	-0.69999	0.50263
p-CO_2Me	1397.72	1851.64	0.80291	-0.59567	-0.70074	0.50254
p-OMe	1396.68	1839.37	0.80480	-0.61182	-0.70667	0.49976
p-F	1396.19	1847.75	0.80562	-0.60242	-0.70414	0.50193
p-CF_3	1401.11	1854.66	0.78303	-0.59324	-0.70152	0.50390
p-Br	1395.33	1849.58	0.80491	-0.59850	-0.70255	0.50251
m-OMe	1396.67	1847.51	0.80599	-0.60040	-0.70218	0.50019
m-CO_2Me	1404.11	1850.19	0.80428	-0.59917	-0.70153	0.50178
m-Cl	1394.82	1852.53	0.80547	-0.59554	-0.70118	0.50252

Table 14. Regioselectivity ratios (rr) of 1,3- vs 2,1-arylfluorinated product and corresponding measured $\Delta\Delta G^{\ddagger}$ from reactions run with various ligands.

R	***Trial 1 rr***	***Trial 2 rr***	***Average rr***	***Measured*** $\Delta\Delta G^{\ddagger}$	NBO_{Pd}	***N-Pd-N angle***
bpyH	47	42	44.5	2.25	0.69727	79.18
bpytBu	16	19	17.5	1.70	0.69774	79.02
bpyMeO	9	7	8.0	1.23	0.69592	79.05
bpyCHO	26	28	27.0	1.95	0.70455	79.44
bpyBr	16	14	15.0	1.60	0.69882	79.09
phenH	17	20	18.5	1.73	0.69779	80.13
phenMe	4	4.5	4.25	0.86	0.69779	79.80
phenPh	15	17	16.0	1.64	0.69909	79.71
phenOMe	11	10	10.5	1.39	0.69662	79.93
dipyridylketone	0.16	0.21	0.19	-0.99	0.67595	88.93
diazafluorenone	0.83	0.71	0.77	-0.15	0.68843	81.95

$\Delta\Delta G^{\ddagger}$ is reported in kcal/mol.

Table 14. Calculated parameters of $PdLCl_2$ complexes.

R	NBO_{Pd}	***N-Pd-N angle***

bpyH	0.69727	79.18
bpytBu	0.69774	79.02
bpyMeO	0.69592	79.05
bpyCHO	0.70455	79.44
bpyBr	0.69882	79.09
phenH	0.69779	80.13
phenMe	0.69779	79.80
phenPh	0.69909	79.71
phenOMe	0.69662	79.93
dipyridylketone	0.67595	88.93
diazafluorenone	0.68843	81.95

Table 15. Regioselectivity ratios (rr) of 1,3- vs 2,1-arylfluorinated product and corresponding measured $\Delta\Delta G^{\ddagger}$ from reactions run with various chromene derivatives.

R	***Trial 1 rr***	***Trial 2 rr***	***Average rr***	***log(1,3 vs 2,1)***	***σ***
p-MeO	7.4	6.9	7.15	0.85	-0.268
p-Me	6.5	7.5	7	0.85	-0.170
H	8.2	7.5	7.85	0.89	0.000
p-F	9.4	9	9.2	0.96	0.062
p-Cl	13.8	12	12.9	1.11	0.227
p-Br	10	9.4	9.7	0.99	0.232
m-Cl	12.6	14	13.3	1.12	0.373
p-Ac	15	14.2	14.6	1.16	0.502

Cartesian Coordinates of Geometry Optimized Structures
benzoic acids
p-Me

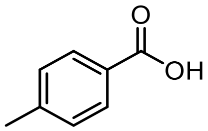

C	0.01585300	-1.18043900	-0.00046100
C	0.69479800	0.03528100	-0.00007300
C	-0.02119700	1.22592400	-0.00038400
C	-1.40566400	1.20074200	-0.00086500
C	-2.09820700	-0.00711500	-0.00092100
C	-1.36715300	-1.19470100	-0.00098400
C	2.17540700	0.10956600	0.00027900
O	2.81336200	1.12865700	0.00040300
H	0.52268400	2.16035600	-0.00047100
O	2.76678400	-1.10215000	0.00051500
H	3.72007700	-0.94452100	0.00078200
H	0.57432800	-2.10515500	-0.00062500
C	-3.60005100	-0.04406700	0.00137000
H	-3.97456200	-0.59589800	-0.86127400
H	-3.97199100	-0.54629100	0.89513400
H	-4.02137700	0.95861300	-0.02558100

H	-1.89366300	-2.14100400	-0.00165800
H	-1.95937100	2.13069400	-0.00141400

H

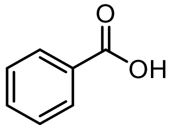

C	-0.44402200	-1.19534300	0.00000300
C	0.21414500	0.03015800	0.00000100
C	-0.51131500	1.21709800	-0.00000900
C	-1.89521100	1.17807200	-0.00001800
C	-2.55361800	-0.04512600	-0.00001800
C	-1.83002100	-1.22936000	-0.00000600
C	1.69655400	0.12263600	0.00001000
O	2.31942600	1.15033800	0.00001000
H	0.02644800	2.15487200	-0.00000900
O	2.30214000	-1.08088000	0.00002000
H	3.25377400	-0.91298100	0.00002600
H	0.12950800	-2.11060000	0.00001300
H	-2.34520100	-2.17929700	-0.00000400
H	-2.46171800	2.09828100	-0.00002400
H	-3.63440400	-0.07475400	-0.00002000

p-COMe

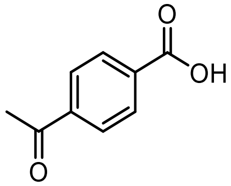

C	0.75387100	-1.19713600	-0.00010900
C	1.39803500	0.03767500	0.00005600
C	0.66028200	1.21495300	0.00016400
C	-0.72312400	1.15915000	0.00010600
C	-1.37448200	-0.07194600	-0.00005800
C	-0.62781100	-1.24769700	-0.00016200
C	2.88194100	0.14936200	0.00012300
O	3.48709000	1.18679100	0.00026300
H	1.18540900	2.15963000	0.00029300
O	3.49953700	-1.04561000	0.00001000
H	4.45023800	-0.87123500	0.00006400
H	1.33804100	-2.10549500	-0.00019100
H	-1.15363100	-2.19196200	-0.00028500
H	-1.29081500	2.07893000	0.00019200
C	-2.87314700	-0.18115500	-0.00012000
O	-3.40427000	-1.26515900	-0.00020300
C	-3.68588100	1.08881600	-0.00007700
H	-3.45333100	1.68978600	0.87979700
H	-3.45308200	1.69002800	-0.87971900
H	-4.73979800	0.83000300	-0.00025300

p-CO_2Me

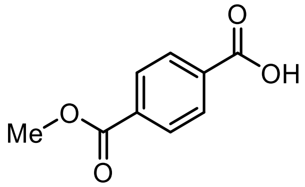

C	1.22816500	1.20896200	0.00018000
C	1.81363100	-0.05345800	-0.00001800
C	1.02324200	-1.19778400	-0.00019500
C	-0.35543300	-1.08354400	-0.00017500
C	-0.94165300	0.17882000	0.00002400
C	-0.15098200	1.32253100	0.00020100
C	3.29119900	-0.23094400	-0.00004700
O	3.85017900	-1.29384200	-0.00019900
H	1.50643900	-2.16452500	-0.00034800
O	3.96152600	0.93566000	0.00011500
H	4.90337700	0.71864000	0.00008300
H	1.85261600	2.08993700	0.00031700
H	-0.63432700	2.28913300	0.00035700
H	-0.98025000	-1.96402600	-0.00031200
C	-2.42295600	0.36026800	0.00006800
O	-2.97034900	1.43069500	0.00024500
O	-3.08436900	-0.80237400	-0.00011300
C	-4.50750000	-0.69047900	-0.00008100
H	-4.84232700	-0.15419300	-0.88556900
H	-4.88534000	-1.70712700	-0.00064300
H	-4.84235700	-0.15518100	0.88599300

p-OMe

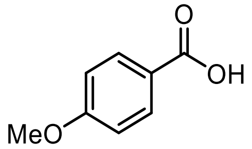

C	-0.54036400	-1.25921700	-0.00000100
C	-1.10322000	0.01844700	0.00001000
C	-0.27295300	1.13031900	0.00003700
C	1.10518200	0.98855300	0.00005900
C	1.65889000	-0.29081900	0.00006200
C	0.82810500	-1.41383700	0.00002300
C	-2.56500500	0.23625900	-0.00001300
O	-3.10388200	1.31210600	-0.00000200
H	-0.72332700	2.11335600	0.00003000
O	-3.27191500	-0.91314400	-0.00005100
H	-4.20495200	-0.66250400	-0.00006400
H	-1.18431500	-2.12656000	-0.00002100
H	1.28665600	-2.39234900	0.00002000
H	1.73158200	1.86698300	0.00006100
O	2.98595500	-0.54028400	0.00005700
C	3.86948900	0.56222200	-0.00011000
H	4.87193800	0.14617600	-0.00032700
H	3.73002700	1.17697800	-0.89167400
H	3.73038400	1.17693400	0.89154100

p-F

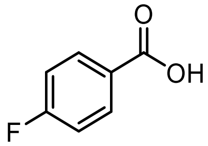

C	-0.02419300	-1.18621600	0.00000600
C	0.64653300	0.03313600	0.00000300
C	-0.07009400	1.22634300	-0.00000600
C	-1.45265600	1.20957700	-0.00001400
C	-2.09278900	-0.01675200	-0.00001100
C	-1.40848900	-1.21753200	-0.00000100
C	2.12719200	0.11305200	0.00001100
O	2.75798600	1.13593600	0.00000900
H	0.47468000	2.15990100	-0.00000800
O	2.72118000	-1.09608900	0.00002000
H	3.67462700	-0.93852200	0.00002400
H	0.53959700	-2.10729900	0.00001300
H	-1.96052200	-2.14590900	0.00000100
H	-2.03835900	2.11710000	-0.00002200
F	-3.43070800	-0.04152100	-0.00001800

p-CF_3

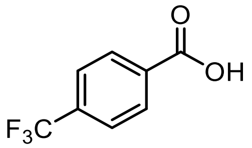

C	-1.01934400	-1.17122900	0.00003600
C	-1.70568300	0.03997600	0.00000800
C	-1.01221400	1.24296100	-0.00004600
C	0.37336700	1.24293100	-0.00008100
C	1.05146300	0.03417300	-0.00005300
C	0.36359800	-1.17285800	0.00001500
C	-3.19348700	0.09687500	0.00000800
O	-3.83476100	1.11153000	-0.00003500
H	-1.57173800	2.16757300	-0.00006800
O	-3.76480100	-1.12051700	0.00005700
H	-4.72177400	-0.98394300	0.00004900
H	-1.56941600	-2.10032100	0.00004400
H	0.91183300	-2.10523500	-0.00002900
H	0.92323900	2.17238600	-0.00013500
C	2.55436100	-0.00807700	-0.00001200
F	3.02531600	-0.65730200	-1.07343800
F	3.02540400	-0.65395300	1.07539900
F	3.09949900	1.21046800	-0.00188200

p-Br

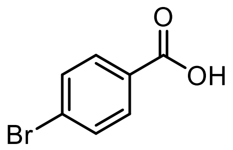

C	-1.08086200	-1.17814400	-0.00001000
C	-1.76098700	0.03467400	-0.00000600
C	-1.05313100	1.23171900	0.00000300
C	0.33033200	1.22383500	0.00000700

C	0.99439000	0.00511500	0.00000300
C	0.30449000	-1.19763200	-0.00000600
C	-3.24420100	0.10341400	-0.00001000
O	-3.88061600	1.12224900	-0.00000700
H	-1.60244700	2.16282000	0.00000600
O	-3.82770300	-1.10986500	-0.00001800
H	-4.78274700	-0.96167200	-0.00002000
H	-1.63533600	-2.10508200	-0.00001600
H	0.84429300	-2.13290900	-0.00000900
H	0.89042900	2.14708700	0.00001400
Br	2.88606200	-0.01563400	0.00000900

m-OMe

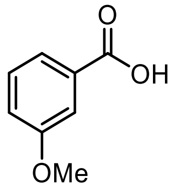

C	-0.06746300	-0.70787700	0.00020400
C	1.00403700	0.16787900	0.00006400
C	0.80094100	1.54713600	-0.00000100
C	-0.49090000	2.03256500	0.00005200
C	-1.58137300	1.16620200	0.00015500
C	-1.36808400	-0.20794700	0.00024700
C	2.40565400	-0.32859600	-0.00007100
O	3.37977700	0.37475100	-0.00023500
H	1.65728000	2.20519200	-0.00009600
O	2.49293100	-1.67245900	-0.00001500
H	3.43442600	-1.88994700	-0.00015300
H	0.08346900	-1.77713700	0.00025600
H	-0.66722400	3.09927400	0.00000600
H	-2.58171400	1.57183900	0.00018700
O	-2.35724400	-1.13503300	0.00044000
C	-3.69063600	-0.67415900	-0.00054100
H	-4.31807500	-1.55999000	-0.00025500
H	-3.90295100	-0.07866100	0.89039500
H	-3.90198100	-0.07985900	-0.89250800

m-CO_2Me

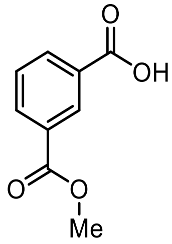

C	-0.77288200	0.59735200	0.00000000
C	0.26156100	-0.32947700	0.00000000
C	1.57677200	0.11741400	0.00000000
C	1.85726800	1.48035400	0.00000000
C	0.82173700	2.40068600	0.00000000
C	-0.49253500	1.95989400	0.00000000
H	2.89144100	1.79582200	0.00000000
H	1.03803100	3.45949600	0.00000000
H	-1.31937800	2.65668500	0.00000000

C	-2.20389200	0.18119700	0.00000000
O	-3.12893800	0.94968500	0.00000100
O	-2.35410200	-1.14796500	0.00000000
C	-3.70597000	-1.60591100	0.00000000
H	-4.22515000	-1.24568100	0.88574300
H	-4.22515100	-1.24567600	-0.88574000
H	-3.65230100	-2.68919200	-0.00000300
H	0.04487500	-1.38661700	0.00000000
C	2.72341100	-0.82831600	0.00000000
O	2.34239100	-2.11904200	0.00000000
O	3.87874600	-0.49944300	0.00000000
H	3.15004100	-2.64987900	0.00000000

m-Cl

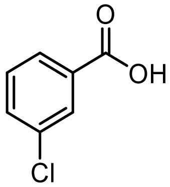

C	0.20187900	-0.61564900	-0.00000400
C	-0.93114000	0.19006800	-0.00000200
C	-0.81745600	1.57547200	0.00000800
C	0.43821200	2.15895900	0.00001700
C	1.57868000	1.36765200	0.00001500
C	1.44719800	-0.01240200	0.00000500
C	-2.29748900	-0.39842500	-0.00001100
O	-3.31385200	0.24088500	-0.00001000
H	-1.71822200	2.17228400	0.00001000
O	-2.29081700	-1.74370500	-0.00002000
H	-3.21387200	-2.03021200	-0.00002600
H	0.11426500	-1.69153500	-0.00001200
H	0.53684800	3.23526800	0.00002500
H	2.56435800	1.81042900	0.00002200
Cl	2.87262800	-1.00398400	0.00000300

ligands

bipyH

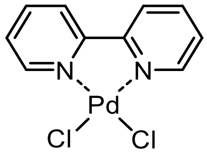

C	0.43838500	-2.66065300	0.00006800
C	1.77380600	-0.73776300	-0.00002300
C	2.93260100	-1.51628600	-0.00004300
C	2.82397000	-2.90443500	-0.00000100
C	1.55889600	-3.48659300	0.00006200
H	-0.58130000	-3.03328600	0.00010200
H	3.90828500	-1.04694600	-0.00008600
H	3.71775900	-3.51954100	-0.00001400
H	1.43015200	-4.56273900	0.00010200
C	1.77380100	0.73777400	-0.00002800
C	2.93259000	1.51630500	-0.00007300
C	2.82394900	2.90445400	-0.00003700

H	3.90827800	1.04697300	-0.00013500
C	0.43836600	2.66065500	0.00007500
C	1.55887000	3.48660200	0.00004600
H	3.71773400	3.51956700	-0.00007200
H	-0.58132200	3.03328000	0.00012300
H	1.43011900	4.56274800	0.00008300
N	0.54985300	-1.32530700	0.00002400
N	0.54984300	1.32530900	0.00003400
Pd	-1.05274600	-0.00000400	0.00000800
Cl	-2.66331900	1.67456000	-0.00001000
Cl	-2.66330900	-1.67457600	-0.00005700

bipy*t*Bu

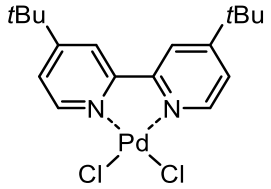

C	-2.65822100	-0.82469600	-0.00055600
C	-0.73816000	0.49431600	-0.00017700
C	-1.51348200	1.65521800	0.00010000
C	-2.90884200	1.58383300	0.00003100
C	-3.47126100	0.29921600	-0.00040100
H	-3.04348200	-1.83939900	-0.00076200
H	-1.02054200	2.61642900	0.00045600
H	-4.54565600	0.15861100	-0.00055000
C	0.73814800	0.49432500	-0.00010500
C	1.51345800	1.65523600	-0.00045700
C	2.90881800	1.58386500	-0.00017100
H	1.02050600	2.61644100	-0.00100600
C	2.65822300	-0.82466700	0.00065600
C	3.47125000	0.29925300	0.00051400
H	3.04349400	-1.83936600	0.00100900
H	4.54564600	0.15866000	0.00088200
N	-1.32070500	-0.72869200	-0.00040100
N	1.32070700	-0.72867600	0.00031500
C	-3.80486800	2.82400200	0.00030400
C	3.80484300	2.82403600	-0.00027400
C	4.69249200	2.79821400	1.26386100
H	5.34570200	3.67631500	1.27864000
H	4.08195500	2.81060100	2.17191700
H	5.32730200	1.90879300	1.29685800
C	4.69637800	2.79566400	-1.26159900
H	4.08865300	2.80594900	-2.17156400
H	5.34940100	3.67390100	-1.27624100
H	5.33158800	1.90640500	-1.29076600
C	-4.69536400	2.79608600	1.26237700
H	-4.08688800	2.80673300	2.17183600
H	-5.34840900	3.67430400	1.27723100
H	-5.33049900	1.90679900	1.29239300
C	-4.69355900	2.79773500	-1.26308300
H	-5.34671800	3.67587600	-1.27765900
H	-4.08377800	2.80973000	-2.17165200
H	-5.32847800	1.90836300	-1.29521800
C	-2.99234300	4.12882500	0.00178400

H	-2.35847100	4.21468300	-0.88676700
H	-3.67472300	4.98325600	0.00211000
H	-2.35940900	4.21320900	0.89114600
C	2.99232500	4.12886000	-0.00292300
H	2.35995000	4.21278100	-0.89272700
H	2.35789500	4.21518500	0.88518300
H	3.67470800	4.98328900	-0.00324700
Pd	0.00001100	-2.33033100	-0.00001500
Cl	-1.68092200	-3.93873800	-0.00057300
Cl	1.68096600	-3.93871400	0.00060100

bipyMeO

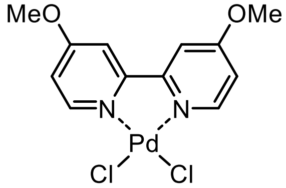

C	-2.65681900	-0.21049800	-0.00000800
C	-0.73898800	1.11688200	-0.00011800
C	-1.50570200	2.27223700	-0.00014200
C	-2.90503800	2.17399100	-0.00006900
C	-3.48960900	0.90199600	-0.00000800
H	-3.03888700	-1.22690600	0.00004500
H	-1.05730800	3.25695000	-0.00019900
H	-4.56140800	0.75726500	0.00004300
C	0.73899400	1.11688000	-0.00013200
C	1.50571200	2.27223000	-0.00004800
C	2.90504800	2.17398100	-0.00001300
H	1.05732100	3.25694500	0.00003000
C	2.65682000	-0.21050600	-0.00019600
C	3.48961600	0.90198500	-0.00011400
H	3.03888600	-1.22691500	-0.00024600
H	4.56141300	0.75724500	-0.00011000
N	-1.32237500	-0.11223800	-0.00005400
N	1.32237700	-0.11224300	-0.00018700
O	3.57872100	3.33870200	0.00013100
O	-3.57871900	3.33870700	-0.00005200
C	-5.00594600	3.29423900	0.00011200
H	-5.38446200	2.78963500	0.89576500
H	-5.33384000	4.33312300	0.00016300
H	-5.38467400	2.78965700	-0.89546300
C	5.00594500	3.29423900	0.00032100
H	5.38469600	2.78975800	-0.89530200
H	5.33382900	4.33312600	0.00049400
H	5.38444400	2.78953900	0.89592800
Pd	-0.00000200	-1.71491300	-0.00002700
Cl	-1.68470900	-3.32197600	0.00026300
Cl	1.68470000	-3.32198100	-0.00004800

bipyCHO

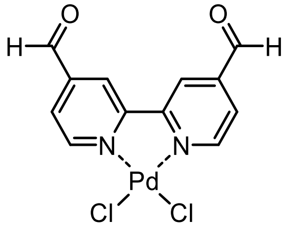

C	0.26055400	-2.66200300	0.00016600
C	-1.07991400	-0.73742300	0.00013800
C	-2.23689100	-1.50911000	0.00006000
C	-2.12815200	-2.90173400	0.00000500
C	-0.86184200	-3.48590300	0.00009400
H	1.28017100	-3.03488100	0.00020400
H	-3.22577900	-1.06711900	-0.00001000
H	-0.74036200	-4.56438300	0.00008000
C	-1.07981600	0.73749500	0.00010300
C	-2.23669400	1.50932800	0.00013600
C	-2.12778000	2.90193700	-0.00000300
H	-3.22563600	1.06745900	0.00026500
C	0.26089900	2.66190700	-0.00017400
C	-0.86139500	3.48594600	-0.00020400
H	1.28056500	3.03465200	-0.00029500
H	-0.73977900	4.56441000	-0.00034900
N	0.14678600	-1.32744100	0.00013400
N	0.14695700	1.32735800	0.00000200
C	-3.36317700	-3.73669100	-0.00019800
C	-3.36270200	3.73704700	0.00006400
O	-4.48024200	-3.26540700	-0.00032500
O	-4.47982500	3.26590000	0.00029200
H	-3.19095600	-4.83241000	-0.00024200
H	-3.19034700	4.83274500	-0.00010000
Pd	1.74447200	-0.00010800	-0.00005100
Cl	3.35387100	-1.66958700	0.00015300
Cl	3.35390600	1.66937200	-0.00009400

bipyBr

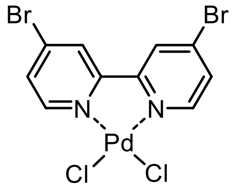

C	2.66050200	0.85806100	0.00018900
C	0.73840400	-0.47152600	-0.00000700
C	1.50775000	-1.63456800	-0.00005700
C	2.89537000	-1.51785400	0.00003200
C	3.48952200	-0.25869400	0.00017500
H	3.03973100	1.87584100	0.00026100
H	1.04734000	-2.61287100	-0.00015600
H	4.56554900	-0.14093500	0.00026300
C	-0.73839100	-0.47154100	-0.00000300
C	-1.50771300	-1.63460000	-0.00006700
C	-2.89533500	-1.51791500	0.00003000
H	-1.04728200	-2.61289300	-0.00018600
C	-2.66051700	0.85800500	0.00022200

C	-3.48951300	-0.25876700	0.00019600
H	-3.03976700	1.87577700	0.00031000
H	-4.56554200	-0.14103200	0.00029100
N	1.32468100	0.75198400	0.00009200
N	-1.32469400	0.75195600	0.00011500
Br	3.96571300	-3.07869500	-0.00003200
Br	-3.96564500	-3.07877900	-0.00005600
Pd	-0.00002600	2.35634400	0.00001600
Cl	-1.67621900	3.96011600	-0.00008600
Cl	1.67612500	3.96016000	-0.00015900

phenH

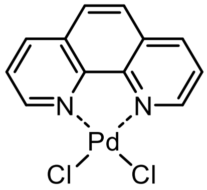

C	1.33559800	3.45201000	0.00034100
C	2.57163100	2.83587700	0.00017800
C	2.64605100	1.42484600	0.00002500
C	1.42470900	0.71548600	0.00016800
C	0.16455400	2.67145200	0.00025700
C	3.87441200	0.68213000	-0.00030800
C	1.42472000	-0.71548000	0.00011400
C	2.64606900	-1.42482900	-0.00012700
C	3.87442100	-0.68210000	-0.00039500
C	2.57165600	-2.83586000	-0.00011600
H	3.48656400	-3.42064200	-0.00022300
C	1.33562600	-3.45200000	0.00007600
C	0.16457500	-2.67145100	0.00016200
H	4.81008500	1.23241300	-0.00046700
H	1.24739100	4.53236400	0.00045400
H	3.48653800	3.42066200	0.00018700
H	-0.83473100	3.09625000	0.00016000
H	4.81010100	-1.23237200	-0.00062600
H	1.24742500	-4.53235500	0.00009900
H	-0.83468700	-3.09628500	0.00015900
N	0.21568700	1.34363700	0.00024000
N	0.21570400	-1.34363300	0.00020500
Pd	-1.38198900	-0.00000400	-0.00000100
Cl	-2.97266300	-1.69066100	0.00015500
Cl	-2.97269400	1.69063900	-0.00045300

phenMe

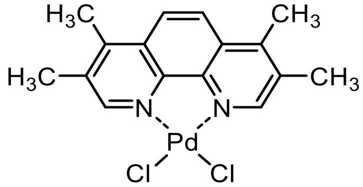

C	3.47196900	0.92685900	-0.00001000
C	2.86123100	2.18143600	0.00000700
C	1.43792900	2.24080800	0.00001100
C	0.71567000	1.02995900	0.00000000

C	2.66109200	-0.22824000	-0.00001500
C	0.68287100	3.46215200	0.00002500
C	-0.71567600	1.02995500	-0.00000200
C	-1.43794200	2.24079900	-0.00000200
C	-0.68289100	3.46214800	0.00001800
C	-2.86124200	2.18141800	-0.00001500
C	-3.47197300	0.92683700	-0.00000800
C	-2.66109000	-0.22825700	-0.00000400
H	1.21311900	4.40713300	0.00004000
H	3.08385200	-1.22887700	-0.00002200
H	-1.21314500	4.40712500	0.00003500
H	-3.08384300	-1.22889700	-0.00000100
N	1.33642200	-0.18117700	-0.00000900
N	-1.33642000	-0.18118600	-0.00000500
C	3.66002000	3.45766100	0.00001700
H	3.42669000	4.06473300	-0.88157700
H	3.42664100	4.06475100	0.88158600
H	4.73235500	3.26865600	0.00004900
C	-3.66004700	3.45763100	-0.00002200
H	-3.42700200	4.06452100	0.88177600
H	-3.42640300	4.06491000	-0.88138700
H	-4.73238000	3.26860800	-0.00042600
C	4.96930400	0.74264600	-0.00002600
H	5.43091900	1.19655900	-0.88318200
H	5.43094900	1.19662700	0.88307900
H	5.22478400	-0.31883400	0.00001000
C	-4.96930700	0.74262600	-0.00000300
H	-5.43093900	1.19662600	0.88309900
H	-5.43093300	1.19652800	-0.88316000
H	-5.22479200	-0.31885300	0.00005300
Pd	0.00000600	-1.77962300	-0.00000100
Cl	-1.69527800	-3.37237900	0.00001500
Cl	1.69529900	-3.37236900	0.00000300

phenPh

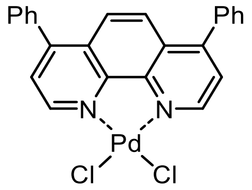

C	-3.44492900	0.25288500	-0.05854300
C	-2.86169800	-1.00698400	0.00274200
C	-1.43545400	-1.07820300	0.00722600
C	-0.71730600	0.13928200	-0.00645500
C	-2.65954300	1.41348200	-0.07325100
C	-0.68262800	-2.29963800	0.00068600
C	0.71733200	0.13910000	0.00660500
C	1.43517200	-1.07856800	-0.00700900
C	0.68203400	-2.29981100	-0.00029500
C	2.86143500	-1.00770900	-0.00266200
C	3.44498700	0.25202000	0.05849200
C	2.65989400	1.41281400	0.07319800
H	-1.22044200	-3.23919300	-0.00912100
H	-4.52458300	0.34983800	-0.05881800
H	-3.07981800	2.41426200	-0.09826500

H	1.21961000	-3.23950100	0.00963600
H	4.52466400	0.34870600	0.05865000
H	3.08042300	2.41349000	0.09812600
N	-1.33267300	1.35534000	-0.03492300
N	1.33300800	1.35500500	0.03495600
C	3.71786800	-2.21484700	-0.06072500
C	4.74577200	-2.38807700	0.87833700
C	3.55178300	-3.17410500	-1.07247500
C	5.57911000	-3.50322100	0.81637000
H	4.87485000	-1.65382200	1.66752400
C	4.39151500	-4.28421000	-1.13654100
H	2.78158700	-3.03210400	-1.82382000
C	5.40377100	-4.45394700	-0.19031400
H	6.36392800	-3.63013900	1.55542600
H	4.25995100	-5.01262400	-1.93056000
H	6.05502100	-5.32092800	-0.23978100
C	-3.71843200	-2.21391300	0.06074300
C	-3.55280500	-3.17310400	1.07263000
C	-4.74614100	-2.38700800	-0.87855600
C	-4.39280100	-4.28301400	1.13660300
H	-2.78275300	-3.03119900	1.82414300
C	-5.57974000	-3.50196300	-0.81668300
H	-4.87486100	-1.65280200	-1.66784700
C	-5.40486000	-4.45262500	0.19014200
H	-4.26159500	-5.01137800	1.93072800
H	-6.36440100	-3.62878400	-1.55592200
H	-6.05631300	-5.31945700	0.23953500
Pd	0.00036800	2.95235900	-0.00000800
Cl	-1.68976800	4.54765300	-0.05244000
Cl	1.69091100	4.54722400	0.05237700

phenOMe

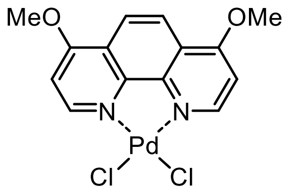

C	-0.74440700	3.45526800	0.00016300
C	-1.99106700	2.84217200	0.00002400
C	-2.06218100	1.41624800	-0.00003300
C	-0.84122600	0.71558400	0.00004200
C	0.41527300	2.66547400	0.00020300
C	-3.29042800	0.68374400	-0.00015800
C	-0.84129600	-0.71550400	0.00003300
C	-2.06231900	-1.41604800	-0.00005100
C	-3.29049500	-0.68342500	-0.00016600
C	-1.99134600	-2.84197900	-0.00000500
C	-0.74474500	-3.45519800	0.00011900
C	0.41501200	-2.66551800	0.00016500
H	-4.22049400	1.23864800	-0.00024000
H	-0.63711700	4.53145900	0.00022700
H	1.41206500	3.09650900	0.00027100
H	-4.22061300	-1.23824000	-0.00025400
H	-0.63756100	-4.53139900	0.00016300
H	1.41176200	-3.09664800	0.00022400

N	0.37337600	1.33894800	0.00013500
N	0.37324500	-1.33898700	0.00011800
O	-3.17159500	3.48519700	-0.00006200
O	-3.17194000	-3.48488000	-0.00008700
C	-3.16818900	-4.91287400	0.00003500
H	-4.21583700	-5.21128600	-0.00015300
H	-2.67300900	-5.30313100	-0.89554400
H	-2.67337200	-5.30297100	0.89588300
C	-3.16766600	4.91318900	-0.00003800
H	-4.21527600	5.21172900	-0.00018900
H	-2.67274900	5.30328900	0.89575500
H	-2.67248300	5.30331800	-0.89567200
Pd	1.97113600	-0.00009500	0.00003200
Cl	3.56052300	1.69967500	-0.00013200
Cl	3.56034900	-1.70002700	-0.00013500

dipyridylketone

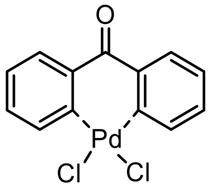

C	-3.70963500	0.90845900	0.93719400
C	-2.63814000	0.02238900	0.86410500
C	-1.31412500	1.61193600	-0.19407500
C	-2.36312100	2.53430800	-0.19089300
C	-3.57790500	2.18044800	0.38759200
H	-4.63233100	0.58532300	1.40541900
H	-2.71014900	-1.00068600	1.20876600
H	-2.19582300	3.50482800	-0.64037900
H	-4.40308300	2.88467000	0.40837300
C	0.00000300	2.09432400	-0.74656200
C	1.31412600	1.61193900	-0.19406000
C	2.36310900	2.53432600	-0.19082800
C	2.63815400	0.02236500	0.86406200
C	3.57789200	2.18045800	0.38765500
H	2.19580400	3.50486300	-0.64027700
C	3.70963700	0.90844400	0.93719600
H	2.71017200	-1.00072600	1.20867800
H	4.40305900	2.88469200	0.40847800
H	4.63233500	0.58530000	1.40541100
O	0.00000800	3.02543100	-1.53576600
N	1.46385500	0.37075100	0.30993700
N	-1.46384300	0.37076300	0.30996700
Pd	0.00000000	-1.08623700	-0.00860300
Cl	-1.65386200	-2.67741100	-0.39968000
Cl	1.65385600	-2.67740000	-0.39974800

diazafluorenone

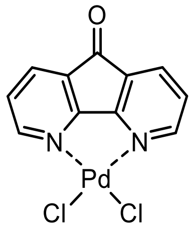

C	0.28908100	-2.76295500	-0.00010900
C	1.55521100	-3.36317200	-0.00013300
C	2.73399000	-2.59354100	-0.00006200
C	2.58104500	-1.21174100	0.00010700
C	1.27987700	-0.72178700	0.00015400
H	-0.63719200	-3.32794500	-0.00021400
H	1.61416100	-4.44567100	-0.00024000
H	3.71248100	-3.06242700	-0.00014700
C	2.58102000	1.21177000	0.00011100
C	2.73393700	2.59357300	-0.00005200
C	1.55514200	3.36318000	-0.00012300
C	0.28902500	2.76293800	-0.00010600
C	1.27986200	0.72179000	0.00015500
H	3.71241900	3.06247900	-0.00013300
H	1.61407100	4.44568000	-0.00022500
H	-0.63726000	3.32790900	-0.00021300
C	3.51035400	0.00002400	0.00008600
O	4.71916000	0.00003700	0.00003500
N	0.15027400	1.42186100	-0.00002500
N	0.15030300	-1.42188100	-0.00002300
Pd	-1.48695700	-0.00000800	0.00000700
Cl	-3.03433200	1.71358600	0.00001200
Cl	-3.03432900	-1.71360400	0.00003100

12. References

1) Ishikawa,T.; Nagai, K.; Ohkubo, N.; Ishii, H. *Heterocycles* **1994**, *39*, 9119-9123.
2) Aissoui, H.; Boss, C.; Gude, M.; Koberstein, R.; Lehmann, D.; Sifferlen, T.; Traschel, D. 3-Aza-Bicylco[3.1.0]Hexane Derivatives, U.S. patent IB 2007/053497, Sept 28, 2007.
3) Efe, C.; Lykakis, I. N.; Stratakis, M. *Chem. Commun.* **2011**, *47*, 803.
4) Lykakis, I. N.; Efe, C.; Gryparis, C.; Stratakis, M. *Eur. J. Org. Chem.* **2011**, 2334-2338.
5) Yoo, K. S.; Park, C. P.; Yoon, C. H.; Sakaguchi, S.; O'Neill, J.; Junk, K. W. *Org. Lett.* **2007**, *9*, 3933-3935.
6) Mezzailles, N.; Ricard, L.; Gagosz, F. *Org. Lett.* **2005**, *7*, 4133-4136.
7) Nag, S.; Lehnman, L.; Kettschau, G.; Toth, M.; Heinrich, T.; Thiele, A.; Varrone, A.; Halldin, C. *Bioorg. Med. Chem.* **2013**, *21*, 6634-6641.
8) Zeng, J.; Vedachalam, S.; Xiang, S.; Liu, X. *Org. Lett.* **2011**, *13*, 42-45.
9) Frisch, M. J. T., G. W.; Schlegel, H. B.; Scuseria, G. E.; Robb, M. A.; Cheeseman, J. R.; Scalmani, G.; Barone, V.; Mennucci, B.; Petersson, G. A.; Nakatsuji, H.; Caricato, M.; Li, X.; Hratchian, H. P.; Izmaylov, A. F.; Bloino, J.; Zheng, G.; Sonnenberg, J. L.; Hada, M.; Ehara, M.; Toyota, K.; Fukuda, R.; Hasegawa, J.; Ishida, M.; Nakajima, T.; Honda, Y.; Kitao, O.; Nakai, H.; Vreven, T.; Montgomery, J. A., Jr.; Peralta, J. E.; Ogliaro, F.; Bearpark, M.; Heyd, J. J.; Brothers, E.; Kudin, K. N.; Staroverov, V. N.; Kobayashi, R.; Normand, J.; Raghavachari, K.; Rendell, A.; Burant, J. C.; Iyengar, S. S.; Tomasi, J.; Cossi, M.; Rega, N.; Millam, M. J.; Klene, M.; Knox, J. E.; Cross, J. B.; Bakken, V.; Adamo, C.; Jaramillo, J.; Gomperts, R.; Stratmann, R. E.; Yazyev, O.; Austin, A. J.; Cammi, R.; Pomelli, C.; Ochterski, J. W.; Martin, R. L.; Morokuma, K.; Zakrzewski, V. G.; Voth, G. A.; Salvador, P.; Dannenberg, J. J.; Dapprich, S.; Daniels, A. D.; Farkas, Ö.; Foresman, J. B.; Ortiz, J. V.; Cioslowski, J.; Fox, D. J. *Gaussian 09*, Gaussian, Inc.: Wallingford CT, 2009
10) Schäfer, A.; Huber, C.; Ahlrichs, R., *J. Chem. Phys.* **1994,** *100*, 5829.
11) E. D. Glendening, J., K. Badenhoop, A. E. Reed, J. E. Carpenter, J. A. Bohmann, C. M. Morales, C. R. Landis, and F. Weinhold *NBO 6.0*, Theoretical Chemistry Institute, University of Wisconsin, Madison 2013.
12) Hansch, C.; Leo, A.; Taft, R. W., *Chem. Rev.* **1991,** *91*, 165.
13) Seeman, J. I., *J. Chem. Edu.* **1986,** *63*, 42.
